# Generating extended foldamer dye stacks and unravelling their evolving exciton dynamics

**DOI:** 10.1038/s41557-026-02082-0

**Published:** 2026-03-23

**Authors:** Leander Ernst, Yongseok Hong, Hongwei Song, Wei Zhang, Elisabeth Lass, Dongho Kim, Frank Würthner

**Affiliations:** 1 Universität Würzburg, Institut für Organische Chemie, Am Hubland, Würzburg, Germany; 2 Spectroscopy Laboratory for Functional π-Electronic Systems and Department of Chemistry, Yonsei University, Seoul, Republic of Korea; 3 Universität Würzburg, Center for Nanosystems Chemistry, Theodor-Boveri-Weg, Würzburg, Germany

**Keywords:** Light harvesting, Supramolecular polymers

## Abstract

In biomacromolecules, many amino acids or nucleotides are needed to obtain defined secondary structures and concomitant advanced functionalities. However, when researchers generate synthetic model analogues composed of dyes to predict the (photo-)functional properties of the respective solid-state aggregates they often use dimer models. Here we introduce a foldamer series of closely *π*-stacked dyes from dimer to 14-mer, obtained by an iterative block-based coupling protocol, that enable the study of oligomer-length effects on their photophysical properties. Spectroscopic techniques identify a distinct change of fluorescence properties at around four to six dye units—affording narrowed fluorescence bands and an increase of the total quantum yield from 47% for the dimer up to 75% for the 14-mer—that is accompanied by the development of a multiexciton state. These results highlight the limitation of the dimer model and motivate future research on well-defined *π*-stacked foldamers, both as models for solid-state materials and as supramolecular wires for future electronic and photonic applications.

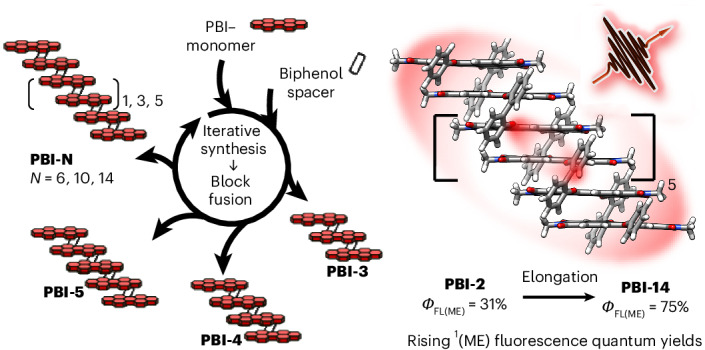

## Abstract (web summary)

Synthetic dye-based analogues of biomacromolecules often rely on simple dimers to model solid-state photofunctional behaviour. Now an iterative block-based coupling protocol has been developed that generates tightly *π*-stacked perylene bisimide foldamers up to the 14-mer. Optical spectroscopy revealed a distinct change of fluorescence properties at around four to six dye units, accompanied by the formation of a multiexciton state.

## Main

The generation of highly ordered secondary structures by non-covalent interaction-driven folding processes is the basic principle observed in protein-based and RNA-based biomacromolecules for the generation of desired functional properties. Accordingly, more than half a century ago, chemists established synthetic methods for the synthesis of oligopeptides^1^ and oligonucleotides^2^ of structures found in nature as well as synthetic variants to gain insight into structure–function relationships and establish the flourishing fields of peptide and oligonucleotide therapeutics. However, the chemical space is much larger than the one used by nature. Particularly useful structures are provided by *π* scaffolds that enable properties such as absorption, emission or charge carrier transport, which during the last two decades established the research field of organic electronics. Highlights from the perspective of structural space are the elaboration of synthetic protocols for extended one-dimensional *π*‑conjugated chains up to lengths of 40 nm (and up to 30 kDa mass)^3–6^, the realization of carbon nanobelts^7^ or expanded two-dimensional nanographene^8^ or nanocone^9^ structures. However, going into the third dimension, which requires control of packing arrangements by intermolecular interactions, still had a few exceptions^10,11^ mostly limited to bi-chromophoric systems that we call here folda-dimers. Considering that inspired from the natural counterparts already a number of well-designed extended foldamer architectures have been accomplished for other synthetic oligomers^12–14^, it is indeed surprising that knowledge about dye–dye interaction in ground and excited states (excitonic coupling) so far has mainly originated from dimer model systems^15,16^ even though it is known that technologically important processes such as energy transfer and singlet fission require larger ensembles. Besides theoretical work^17^, such larger ensembles have so far been either limited to solid-state materials^18,19^ or self-assembled dye aggregates^20,21^ that do not compare with the well-defined discrete and monodisperse nanostructures provided by proteins and oligonucleotides. Also, due to the lack of directionality of dispersion interactions, self-assembled structures composed of *π* scaffolds are prone to dynamic processes, internal structural disorder and dissociation on dilution, solvent or temperature change and other environmental stimuli such as illumination by light. For this reason, defined secondary structures formed by covalent bonds between the respective *π* systems seem to be better suited than their self-assembled counterparts to gaining deep insights into structure–function relationships in the context of photofunctional applications.^22^

In recent years we and others have started to address the challenge of synthesizing well-folded dye stacks beyond dimers and to explore their photofunctional properties^23,24^. Similar to previous research on self-assembly^25^, perylene bis(dicarboximide) (PBI) dyes with their exceptional fluorescence and n-type charge transport properties have proved to be most suitable photofunctional building blocks. Most recently, an extension towards a tetramer stack composed of different PBIs has been accomplished^26^. In this article we go an important step further by introducing a pioneering synthetic protocol that enabled us to accomplish a stack size of up to 14 PBI units. Our structural investigations confirm the presence of highly ordered structures for the whole series up to a molecular weight of 14.3 kDa, which is comparable to small proteins. By use of steady-state and time-resolved optical spectroscopy, we demonstrate a pronounced change of the excited-state dynamics leading to improved fluorescence properties on the expansion of the *π*-stack size at around 4–6 PBI dyes with further saturation on extension to the 14-mer. These insights pinpoint the limitations of the common ‘dimer approach’ and illustrate the opportunities provided by research devoted to larger foldamer *π* stacks for a more accurate prediction of functional properties in related solid-state materials.

## Results

### Synthesis and structural characterization

**Fig. 1:**
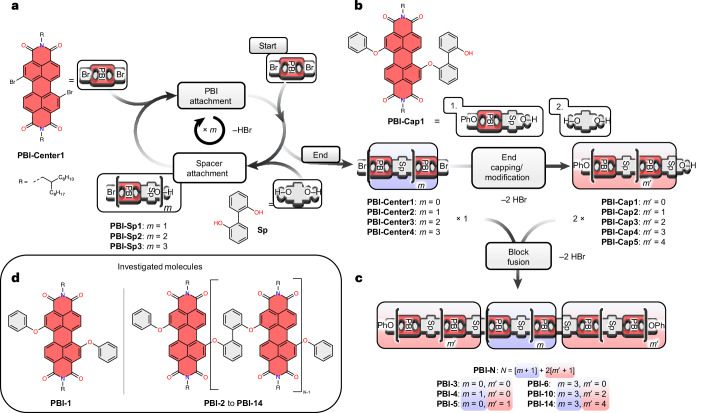
Block-based approach for the synthesis of extended PBI oligomers. **a**, Repetitive synthesis of central building blocks (**PBI‑Center2** to **PBI‑Center4**). **b**, Synthesis of large end-capping fragments (**PBI‑Cap2** to **PBI‑Cap5**). **c**, Final block fusion (**PBI‑3** to **PBI‑14**). **d**, Molecular structure of investigated molecules. The 2,2′-biphenol spacer is abbreviated to **Sp** and the number in each molecules name gives the number of PBI units. As solubilizing substituents branched, 2-hexyldecyl imide substituents R were applied. **PBI-1**, **PBI-2** and **PBI-Cap1** have been synthesized following alternative routes (Supplementary Fig. 1).

The herein introduced block-based approach towards PBI foldamers starts with a stepwise protocol similar to that applied in peptide synthesis but subsequently relies on the fusion of larger blocks towards the desired more extended foldamers (Fig. 1). In this way larger oligomers become more easily accessible and purification via size-exclusion chromatography is simplified. As illustrated in Fig. 1, the synthesis of PBI arrays **PBI‑3** to **PBI-14** starts with the repetitive generation of central building blocks ranging from two (**PBI-Center2**) up to four (**PBI‑Center4**) PBI units. This can be accomplished starting from a 1,7‑dibromo-substituted PBI precursor (**PBI‑Center1**) that is substituted by a 2,2′-biphenol spacer unit (**Sp**, spacer attachment) in one bay position (Fig. 1, left). By repeating the cycle (PBI attachment), central building blocks are prepared. The versatility of the herein proposed central units is based on two bromine substitution sites in the bay position that renders them suitable as central units as well as further substituted end-groups to finalize the generation of large PBI arrays (Fig. 1, right). To subsequently yield large end-capping fragments (**PBI‑Cap2** to **PBI‑Cap5**), the central building blocks of required length (*m* = 0–3) are first substituted once by a monomeric end-unit (1., **PBI-Cap1**) followed by a spacer moiety (2., **Sp**) (top right, end capping). Afterwards, an excess of end-capping fragments (**PBI‑Cap2** to **PBI‑Cap5**) is reacted with one equivalent of the required central building block (**PBI‑Center1** to **PBI-Center4**) to yield the desired symmetrical foldamers (**PBI-3** to **PBI-14**) (bottom right, block fusion). The block-based sequence ensures a fast and versatile synthesis of large PBI oligomers while offering the possibility to vary end-capping units and further extend foldamer sizes. The shorter oligomers **PBI-1** and **PBI-2** have been synthesized following established routes (Supplementary Fig. 1).

**Fig. 2:**
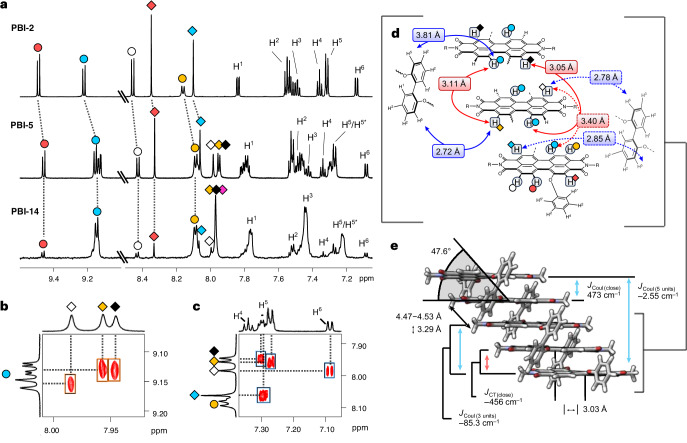
Structural characterization by NMR and DFT. **a**, Excerpts of the ^1^H NMR spectra (600 MHz, TCE‑*d*_2_, 384 K) of **PBI-2** (top), **PBI-5** (middle) and **PBI‑14** (bottom) with PBI core protons marked in colours (singlet, rhomb; multiplet, circle) and each spacer proton number labelled. **b**,**c**, Excerpts of the ^1^H–^1^H rotating frame nuclear Overhauser effect spectroscopy/homonuclear correlation spectroscopy (600 MHz, TCE‑*d*_2_, 384 K) superposition of **PBI-5** with relevant cross coupling signals between core protons (**b**, orange) and core or spacer protons (**c**, blue). **d**, Excerpt of the molecular structure of **PBI-5** to visualize the assignment of all protons, important couplings indicated by arrows with corresponding distances. **e**, Molecular structure of **PBI‑5** obtained from DFT geometry optimization (ωB97X-D/def2‑SVP) with imide substituents replaced by methyl groups including the long-range (*J*_Coul_, blue) and short-range (*J*_CT_, red) couplings obtained from TD‑DFT calculations. Source data

To gain insight into the packing of the chromophores, 600-MHz ^1^H NMR spectroscopy was performed in deuterated 1,1,2,2-tetrachloroethane (TCE-*d*_2_, 384 K). The limited number of ^1^H NMR signals (Fig. 2a) verify the high symmetry within the stack. When increasing the molecular size from **PBI-2** to **PBI-14** an additional singlet signal per chromophore unit is observed, representing two chemically equivalent protons located in the centre of the stack. ^1^H NMR signals can only be resolved fully until **PBI‑5**. Excerpts from the ^1^H NMR spectra of **PBI‑2**, **PBI‑5** and **PBI‑14** illustrate the general upfield shift of the PBI core protons (9.5–7.9 ppm), indicative of the increased shielding effect upon intramolecular stacking (Fig. 2a). Within the stack the protons highlighted in red and blue (circle) exhibit a substantial downfield shift due to their location opposite to the oxygen atoms of the phenoxy units^27^. In contrast, a pronounced upfield shift is observed for the core protons highlighted in blue (rhomb) (**PBI‑2**) and black (rhomb) (**PBI-5**) centred within each stack, exposed to the ring current and magnetic shielding effect of the phenoxy moieties. For **PBI‑10** and **PBI‑14** a saturation of the chemical shift of the core protons is observed.

Two-dimensional rotating frame nuclear Overhauser effect spectroscopy and homonuclear correlation spectroscopy spectra were recorded in TCE-*d*_2_ at 384 K to further investigate the structural orientation within the stack and to obtain a detailed assignment for all observed proton signals (Fig. 2b,c). The vicinity of the chromophores is exemplary demonstrated for **PBI-5** (for the other oligomers, see Supplementary Figs. 5‒9) by intense cross signals between the protons marked in blue (circle) and white, yellow and black (rhomb) (Fig. 2b). The through-space coupling between the spacer (H^5^, H^6^) and the core protons (blue, white, yellow and black rhomb) supports the tightly stacked geometry of the chromophores for **PBI-5** (Fig. 2c). For better interpretation, the couplings are visualized in the section of the molecular structure of **PBI-5** (Fig. 2d), including their respective H–H distances. Finally, diffusion ordered spectroscopy was performed, yielding increasing hydrodynamic radii from **PBI‑2** (11.8 Å) to **PBI‑14** (26.4 Å) (Supplementary Figs. 10 and 11). Further structural elucidation is based on geometry optimized structures, which are in good agreement with the NMR data and a single crystal X-ray structure obtained in our earlier work for a related PBI trimer^24^. With a longitudinal slip of 3.03–3.06 Å between the *π‑*stacked neighbouring PBI moieties at the van der Waals distances of 3.2–3.3 Å (Fig. 2e) the oligomer series features a centre-to-centre offset of ~4.5 Å and a longitudinal slip angle of 46.0–‍47.6°, accompanied by a rotational offset of 6.6–‍11.8° and ~0 Å in the transversal shift. The tight stacking of the PBI *π* scaffolds affords a pronounced overlap of *π* orbitals. For the given geometry, a pronounced charge-transfer (CT) coupling is expected that according to time-dependent-density functional theory (TD‑DFT) calculations for **PBI‑5** establishes long-range Coulomb couplings of *J*_Coul_ = 473 cm^−1^ and short-range CT couplings of *J*_CT_ = −456 cm^−1^ between two proximate chromophores (Fig. 2e and Supplementary Table. 1). The overall Coulomb coupling is augmented by weaker couplings between PBI dyes at a distance of three units (−85.3 cm^−1^) and five units (−2.55 cm^−1^). These quantum chemical simulations suggest a near destructive quantum interference of Coulomb and CT couplings leading to null-like coupling situation as already known for previously studied structurally related null-type PBI dimers and trimers^24,26,27,28^ (Fig. 2e and Supplementary Table 1).

### Steady-state and time-resolved optical spectroscopy

**Fig. 3:**
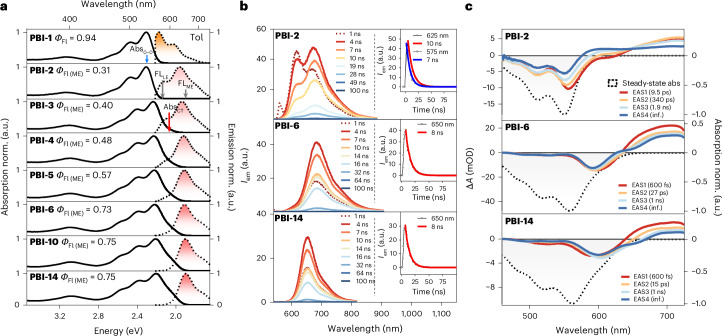
Optical characterization by steady-state and time-resolved spectroscopy. **a**, Steady-state absorption and emission (coloured) spectra of **PBI‑1** to **PBI‑14** recorded in Tol with their relative fluorescence quantum yields of the ME state (*Φ*_FL (ME)_), (total fluorescence quantum yield can be higher in Tol, Supplementary Table 3). Important absorption (Abs_0–0_, blue; Abs_CT_, red) and emission (FL_LE_, FL_ME_, grey) bands are marked with arrows. **b**, ns-TF spectra of **PBI-2** (top), **PBI-6** (middle) and **PBI‑14** (bottom) in Tol with temporal traces as insets, using a 520 nm pump pulse. The decay lines were fitted by a single exponential. The spectra at 1 ns are within instrumental response function (~3 ns). **c**, fs‑TA EAS of **PBI-2** (top), **PBI-6** (middle) and **PBI‑14** (bottom) in Tol, using a 520 nm pump pulse. Corresponding steady-state spectra are inverted with filled curves (grey). mOD, milli-optical density. Source data

The electronic coupling among slip-stacked PBI chromophores was investigated by steady-state absorption spectroscopy in toluene (Tol), tetrahydrofuran (THF) and benzonitrile (BCN) (Fig. 3a for Tol and Supplementary Figs. 17 and 18). The monomer-like spectral profiles observed for all PBI stacks support the predicted negligible (null-type)^28^ exciton coupling. Nevertheless, the spectra exhibit size-dependent features as a function of the number of stacked chromophores. Specifically, the progressive redshift and broadening of the absorption spectra are observed for **PBI-2** to **PBI-14**, attributable to enhanced CT interactions, and extended exciton delocalization. Notably, the increasing CT absorption band at ~600 nm, supplemented by corresponding fluorescence spectra, reinforce this trend, showing clear signatures of increasing CT contributions (Fig. 3a). In line with previous findings on a PBI trimer, the fluorescence spectra comprise two distinct bands at ~575 nm and ~625 nm, assigned to locally excited (LE) and multiexciton ^1^(ME) states, respectively^24^. On the basis of solvent-dependent transient absorption analyses across literature known oligomer series^24,29^, we assign the ^1^(ME) state as a multiconfigurational state involving contributions from LE, CT and triplet-pair configurations^23,30^. While a pure triplet-pair state is optically dark, the presence of CT character within the ME state enables an efficient ME generation (MEG) process through a CT-mediated pathway. Conversely, the triplet-like component of the ME state can facilitate the formation of two free triplets (^1^(ME) → ^5^(ME) → ^3^T + ^3^T)^31^. As the stack length increases, the emission spectra narrow while the ^1^(ME) fluorescence becomes increasingly dominant (Fig. 3a), a trend that will be discussed in greater detail below. To gain further insight into excited-state dynamics, nanosecond time-resolved fluorescence (ns-TF) spectroscopy was used (Fig. 3b). All time-resolved measurements were conducted in Tol to examine the influence of intramolecular CT interactions on excited-state dynamics. The evolution of the ns-TF emission spectrum for **PBI-2** (Fig. 3b, top) reveals two emission peaks centred at ~575 nm (LE) and ~625 nm ^1^(ME). The LE/ME peak ratio decreases from 1.34 at 1 ns to 0.4 at 28 ns, indicating a dynamic LE-to-ME conversion. Fluorescence decay kinetics reveal a shorter lifetime for the LE (7 ns) compared with the ME state (10 ns), consistent with this interconversion process. Similar dynamics were observed for **PBI-3** to **PBI-5** (Supplementary Figs. 46–48), indicating a consistent excited-state behaviour across short oligomers. In contrast, for longer stacks such as **PBI-6** (Fig. 3b, middle), **PBI-10** (Supplementary Fig. 49) and **PBI-14** (Fig. 3b, bottom), a single emission peak near 650 nm emerges within 1 ns, with a lifetime of approximately 8 ns. This behaviour suggests that beyond five chromophore units, the ME state dominates the fluorescence emission.

To further investigate the excited-state dynamics of null-type coupled PBI stacks, femtosecond transient absorption (fs-TA) and nanosecond transient absorption (ns-TA) spectroscopy (Fig. 3c and Supplementary Figs. 28–41) was performed in Tol. In particular, fs-TA spectroscopy was used to probe the ultrafast formation of the ^1^(ME) state (Fig. 3c and Supplementary Figs. 28–34), which is further analysed by global analysis based on the evolution-associated decay spectrum (EAS) model^32^. For **PBI-2**, the fs-TA spectrum reveals a rise of the excited-state absorption (ESA) near 720 nm within 9.5 ps, corresponding to the formation of an LE–CT mixed state, facilitated by solvation and structural relaxation (EAS2). This is followed by the appearance of an ESA band at ~600 nm (EAS3), signalling the MEG process on a timescale of ~340 ps, in agreement with previous reports on similar PBI dimers^16^. The deviation of ground-state bleach (GSB) features from the steady-state absorption spectrum below 600 nm is attributed to the ^1^(ME) state and exciton delocalization effects, leading to an ESA evolution around 540 nm^24^.

In contrast, **PBI-6** exhibits distinct spectral features representative of larger stacks in the series. The initial TA spectrum at 500 fs reveals GSB contributions below 650 nm that deviate from the steady-state absorption spectrum, suggesting a unique evolution of ESA around 580 nm due to enhanced electronic coupling. Furthermore, the PBI anion band near 720 nm detected within 500 fs indicates that LE and CT contributions are already superimposed in the Franck–Condon state. The MEG process in **PBI-6** (Fig. 3c, middle) proceeds via biphasic kinetics (0.7 ps and 27 ps), with a clear isosbestic point at ~640 nm, supporting the conversion from mixed LE–CT to ^1^(ME) states. The broader ESA features observed suggest enhanced LE–CT mixing in the ^1^(ME) state, consistent with the hybridized nature typically found in singlet fission systems^15,24,31,33,34^. Moreover, an early-time ESA narrowing indicates an exciton localization process, probably reflecting a geometry-specific relaxation pathway for the bright exciton^35–37^. For **PBI-14**, the MEG process also follows biphasic kinetics, accompanied by a spectral shift of ESA bands. In addition to the biphasic MEG process, a redshift in the ESA without isosbestic point between ME states (EAS2 and EAS3, Fig. 3c) and pump-power-independent kinetic traces (Supplementary Fig. 33) point to the presence of exciton diffusion while excluding exciton–exciton annihilation. Overall, the exciton localization mechanism and strong CT configuration play a pivotal role in facilitating the ultrafast MEG process by enhancing non-adiabatic couplings between the S_1_ and ME states, which is further supported by fs-TA in THF (Supplementary Fig. 34).

The singlet fission process is commonly described as a sequential two-step mechanism, in which a singlet exciton evolves into a ME state that subsequently dissociates into two free triplet excitons. As illustrated in Supplementary Figs. 35–41, we performed ns-TA spectroscopy to understand the full singlet fission process in null-type PBI stacks. The GSB signal around 600 nm and the ESA signal below 600 nm exhibit rapid decay, consistent with the time-resolved fluorescence spectra shown in Fig. 3b. Following the rapid recovery of the GSB at 600 nm and the decay of ESA above 600 nm, a residual GSB and a new ESA signal around 500 nm emerge in **PBI-2**, **PBI-6** and **PBI-14**, which are attributed to free triplet excitons with the lifetime of ~70 μs. To quantify the relative triplet quantum yield, we used a singlet oxygen generation method using 9,10-dimethylanthracene (DMA) as singlet oxygen probe^38,39^. The relative singlet oxygen efficiency obtained in this way directly reflects the triplet quantum yield (Supplementary Figs. 43–45). Despite the formation of free triplets, the high fluorescence yield suggests that the substantial binding energy of the ME state probably promotes ME fusion over dissociation into free triplets. As the stack lengthens, the free triplet quantum yield (~50% for **PBI-2** to ~15% for **PBI-6**) decreases while the fluorescence quantum yield increases, driven by fusion from the ^1^(ME) state into the singlet state. Two factors probably contribute to this behaviour: (1) enhanced structural rigidity in longer stacks suppresses fluctuations required for ^1^(ME) → ^5^(ME) conversion^40–42^, and (2) stronger S_1_–^1^(ME) coupling promotes the reverse fusion process into the singlet state. Consequently, the free triplet yield diminishes with elongation, while the fluorescence quantum yield increases and eventually saturates at **PBI-6**. We note that the TA analysis of **PBI-10** and **PBI-14** is complicated by severe spectral overlaps among GSB, SE and ESA signals, which limits detailed kinetic resolution at this stage.

## Discussion and conclusion

Steady-state absorption and fluorescence spectra of the extended null-type coupled PBI arrays (Fig. 3a) provide critical information on the evolving electronic interactions. Earlier studies on smaller covalent PBI assemblies established key photophysical insights such as excimer^35–37^ formation and suppression, electronic state mixing^23,24,29,43^, CT and/or charge separation^44,45^ and singlet fission^24,46^. Nevertheless, the functional relationship between molecular aggregates (as hitherto disclosed for dimers and trimers) and solid-state properties such as exciton delocalization^29,47,48^, migration^36,49^ and localization has yet to be fully resolved. Extending to larger, covalently linked arrays allows us to approach solid-state regimes while maintaining molecular precision.

The presented null-coupling case can only be roughly described with a nearest-neighbour model because in closely packed *π* stacks (~3.3 Å) long-range dipole–dipole and short-range orbital-overlap interactions remain substantial beyond the first neighbour, as predicted by extended Frenkel–Holstein models^18,50–52^. Incorporating these couplings broadens exciton bandwidths, increases coherence lengths, redistributes oscillator strength and alters vibronic 0–0/0–1 intensity ratios, which was observed in conjugated polymers and PBI aggregates^18,50,52^. In particular, nearest-neighbour models underestimate spectroscopic and transport behaviour, as dipole–dipole interactions and transition density overlap. Consistently, our arrays show progressive absorption broadening, emission narrowing and higher fluorescence yields with elongation, underscoring the inadequacy of dimer-based descriptions for solid-state PBI systems.

**Fig. 4:**
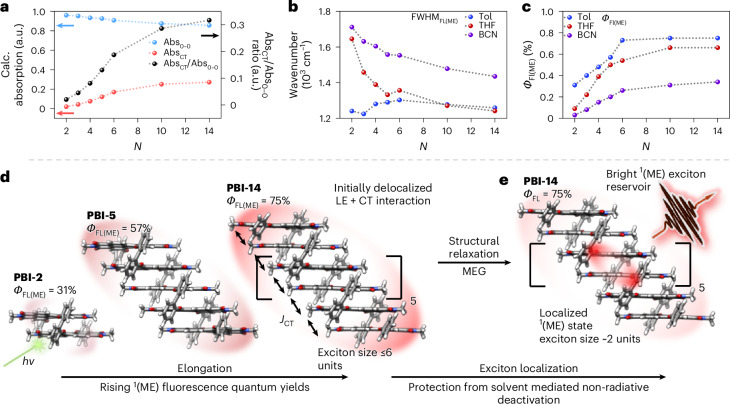
Interpretation of exciton dynamics. **a**, Absorption maxima of the 0–0 transition (Abs_0–0_, blue), CT band (Abs_CT_, red) and intensity ratio of Abs_CT_ (600 nm) over Abs_0–0_ (550 nm) (black) in Tol as a function of molecular size. **b**, FWHM of the ^1^(ME) emission in Tol (blue), THF (red) and BCN (purple). **c**, Fluorescence quantum yield of the ^1^(ME) state (*Φ*_FL(ME)_) in Tol (blue), THF (red) and BCN (purple). The parameters were estimated by fitting of absorption and fluorescence spectra using multiple Gaussian functions (Supplementary Figs. 16–21). **d**, Schematic illustration of increasing fluorescence quantum yields on stack elongation with fluorescence quantum yields of the ^1^(ME) state (*Φ*_FL(ME)_) of **PBI-2** (left), **PBI-5** (middle) and **PBI-14** (right) in Tol). **e**, Schematic illustration of the exciton localization process on structural relaxation for **PBI-14** with corresponding fluorescence quantum yields of the ^1^(ME) state (*Φ*_FL(ME)_) in Tol. Source data

While the 0–0/0–1 intensity ratio in PBI absorption spectra is conventionally used to infer H-type or J-type excitonic coupling^11,23^, another metric used in this study signifies effectively enhanced intramolecular PBI–PBI CT interactions. The Abs_CT_ (~600 nm) to Abs_0–0_ (~550 nm) intensity ratio increases from 0.02 (**PBI-2**) to 0.32 (**PBI-14**) in Tol (Fig. 4a for Tol, Supplementary Fig. 25), reflecting a progressive enhancement of intramolecular electronic interactions between LE and CT states, comparable to literature known and theoretically investigated systems^24,52^. Non-nearest-neighbour long-range Coulomb and short-range orbital-overlap couplings increase the exciton manifold size and bandwidth, generating more distinct eigenstates that can be optically populated. In addition, a pronounced CT admixture, which rises on elongation and constructive interference of multiple coupling terms, introduces additional transitions, further broadening the absorption spectra. Correspondingly, the fluorescence intensity ratio Fl_(ME)_/Fl_(LE)_ in Tol increases markedly with stack size, rising from ~2 in **PBI-2** to ~6 in **PBI-5** and reaching ~67 in **PBI-14**, indicating an enhancement of CT-mediated coupling across the *π* stack (Supplementary Fig. 26). Collectively, these findings reveal that an enhanced CT coupling is of utmost importance in mediating an ultrafast MEG via strong non-adiabatic coupling between the S_1_ and ^1^(ME) states^36^.

Elongation of the stack furthermore induces a narrowing of the full-width at half-maximum of the ^1^(ME) fluorescence (FWHM_FL (ME)_), along with increased ^1^(ME) fluorescence quantum yields (*Φ*_FL (ME)_) (Fig. 4b,c and Supplementary Tables 3–5). These observations suggest a reduction in the density of accessible emissive states and an exciton localization process by the increasing structural rigidity of the central chromophores within the extended stacks. Solvent-dependent trends further support this interpretation. In Tol, FWHM_FL (ME)_ remains relatively constant; however, in more polar solvents such as THF and BCN, a pronounced narrowing is observed with increasing stack length, consistent with a localization of the exciton at the centre of the array^6^ (Fig. 4b). In addition, as predicted by the Shuai group, upon aggregation within the weak to intermediate coupling regime non-radiative deactivation can be suppressed. It was theoretically shown that larger exciton delocalization, reduced reorganization energies, small energy gap changes and increasing excitonic couplings on elongation promote high quantum yields, counteracting the energy gap law^53^. These observations hold true for systems with an exciton coupling/monomer reorganization ratio smaller than 0.5, which is the case for our PBI null-type aggregates.

For **PBI‑2**, *Φ*_FL (ME)_ decreases sharply in polar environments, from 0.31 in Tol to 0.09 in THF and 0.03 in BCN, due to accelerated CT-mediated non-radiative decay processes such as symmetry-breaking charge separation effects. In stark contrast, *Φ*_FL (ME)_ values for longer arrays increase proportionally more pronounced in polar solvents, reaching 0.66 in THF and 0.34 in BCN for **PBI‑14**, representing enhancements of 7.3-fold and 11.3-fold over **PBI-2**, respectively (Fig. 4c). In addition, radiative (*k*_r_) and non-radiative (*k*_nr_) decay rate constants indicate that solvent-mediated CT interactions facilitate non-radiative deactivation, particularly in shorter oligomers (Supplementary Fig. 27). In contrast, a marked suppression of non-radiative decay pathways is observed in longer arrays, especially beyond **PBI-6**. These observations support the emergence of a spatially confined ^1^(ME) state localized in the central chromophore segments.

These trends plateau beyond **PBI‑6**, as evidenced by the saturation in both FWHM_FL (ME)_ and *Φ*_FL (ME)_, indicating that six chromophores are sufficient to form a central, solvent-shielded ^1^(ME) domain. Collectively, these findings point to a progressive enhancement in LE–CT mixing with stack length, leading to the formation of localized, emissive ^1^(ME) states with increasingly high quantum yields (Fig. 4d,e). This confinement is explained by an effective shielding of the emissive state from non-radiative solvent-induced quenching, comparable to bright, localized excitons^35,49,54,55^ or sterically shielded systems^56^. In shorter oligomers, by contrast, the ^1^(ME) state remains more exposed to the environment, facilitating non-radiative deactivation pathways. In addition, shorter oligomers exhibit more conformational flexibility that may enable relaxation processes into symmetry-breaking charge separation or singlet fission pathways^15,24^. It is noteworthy that this improvement in fluorescence efficiency arises without any spectroscopic signatures of J-type excitonic coupling and contrasts with substantial suppression of fluorescence typically observed in H-type coupled systems (for example, excimers), emphasizing the importance of non-nearest-neighbour interactions and a distinct structure–function relationship that emerges only in the extended null-coupled *π*-stacked foldamers. Accordingly, a 14-mer PBI stack could be regarded as ideal experimental example for extended excitonic coupling and exciton delocalization, consistent with theoretical calculations, providing the opportunity for quantitative comparison via Frenkel–Holstein simulations in a future study.

In conclusion, by exploiting the foldamer concept^12–14^ we have accomplished a pioneering example for an iterative synthesis of extended configurationally and conformationally well-defined *π*-stacked structures composed of tightly stacked PBI dyes containing up to 14 subunits. Comparable to natural counterparts such as peptides and oligonucleotides for which stable secondary structures only evolve on sufficient elongation (~15 for α‑helices of alanine-rich peptides and 7 for DNA double strands)^57,58^ our comprehensive investigation by optical spectroscopy highlights the necessity of having at least six PBI dyes tightly stacked for the light-induced fast generation of a highly luminescent ^1^(ME) state. For such elongated PBI oligomer stacks, the exciton can localize in the more rigid central part of the stack that is similar to the α‑helical peptide counterparts^58^, and is less prone to conformational disorder, temperature-induced motions and interaction with the solvent molecules. These localization and rigidification effects obviously hinder triplet–triplet-pair dissociation, resulting in decreasing yields of free triplet species on elongation. Accordingly, our work reveals the limitation of the common dimer approach and the necessity for the synthesis of larger oligomers than dimers for the prediction of electronic properties of molecular solid-state materials. Further, our insights for PBI foldamers hold promise for making use of similar supramolecular arrangements in PBI-based organic semiconductors in optoelectronic devices^59,60^. With their increased brightness and prolonged lifetime, extended dye stacks might also have potential in future nanoelectronic and nanophotonic devices.

## Methods

### Synthesis

The necessary nucleophilic aromatic substitution reactions with phenol and 2,2′‑biphenol were carried out in high boiling solvents (for example, DMF, for details see Supplementary Information). To prevent the common challenge of low solubility of extended *π*-conjugated systems, even in low polar solvents, branched 2‑hexyldecyl alkyl chains were introduced as imide substituents. The shorter oligomers **PBI-1** and **PBI-2** have been synthesized following established routes (Supplementary Fig. 1). Detailed synthetic procedures and characterization of all molecules are provided in the Supplementary Information (Supplementary Figs. 1–‍4).

### Quantum chemical calculations

Structural elucidation is based on geometry optimized structures on the level of long‑range corrected DFT for **PBI-2** to **PBI-5** (Supplementary Figs. 12 and 13) and the Merck molecular force field (MMFF94) for **PBI-6** to **PBI-14** to reduce computational costs. To scrutinize the electronic coupling between PBI chromophores in their slip-stacked arrangement, TD-DFT (ωB97X-D/def2-SVP) calculations have been conducted applying a polarizable continuum solvation model in Tol. The effective electron and hole transfer integrals have been derived applying the Amsterdam Density Functional (PW91/TZP) program. It must be noted that structural fluctuations substantially influence coupling values, especially those derived from orbital overlap^61^. As the couplings are simply estimated to account for the optically observed null-coupling behaviour, any fluctuation-dependence has been neglected.

### Relative free triplet yield determination

The singlet oxygen generation efficiencies were measured with DMA as the singlet oxygen scavenger. On photoexcitation at 520 nm on **PBI-N** (*N* = 2–14) oligomers, the DMA absorption was monitored. The singlet oxygen generation efficiency can be calculated by using the following equation^38,39^.

1 $${\varPhi }_{\mathrm{sam}}={\varPhi }_{\mathrm{std}}\left(\frac{1-{10}^{-{A}_{\mathrm{std}}}}{1-{10}^{-{A}_{\mathrm{sam}}}}\right)\left(\frac{{m}_{\mathrm{sam}}}{{m}_{\mathrm{std}}}\right){\left(\frac{{\eta }_{\mathrm{sam}}}{{\eta }_{\mathrm{std}}}\right)}^{2}$$

where sam stands for the target sample and std for the standard sample; and *η*, *A*, *Φ* and *m* denote the refractive index of the solvent, absorbance at excitation wavelength, singlet oxygen quantum yield and slope of the absorbance of DMA changing with time, respectively. Rose Bengal in methanol was used as the standard with singlet oxygen efficiency of 80%. For the singlet oxygen generation measurements, the concentrations of Rose Bengal and **PBI-N** oligomers were set as (2–3) × 10^−5^ M while the concentrations of DMA was set as (7–9) × 10^−5^ M.

## Online content

Any methods, additional references, Nature Portfolio reporting summaries, source data, extended data, supplementary information, acknowledgements, peer review information; details of author contributions and competing interests; and statements of data and code availability are available at 10.1038/s41557-026-02082-0.

## Supplementary information

Supplementary Information Supplementary Figs. 1–109, Text and Discussion and Tables 1–5.

Supplementary Data Source data for Supplementary figures.

## Source data

Source Data Fig. 2 Source Data Fig. 2.

Source Data Fig. 3 Source Data Fig. 3.

Source Data Fig. 4 Source Data Fig. 4.

## Data Availability

All experimental procedures, analytical data and computational data supporting the findings of this study are available within the paper and its Supplementary Information. Data are available via Zenodo at 10.5281/zenodo.15690640 (ref. ^62^). Source data are provided with this paper.
