# Supplementary material for: Generating extended foldamer dye stacks and unravelling their evolving exciton dynamics
Source: Nat Chem. 2026 Mar 23;18(5):923–30. doi: 10.1038/s41557-026-02082-0 (PMC13149034; doi:10.1038/s41557-026-02082-0)
Supplement: Supplementary file 1 — Supplementary Figs. 1–109, Text and Discussion and Tables 1–5. [file 41557_2026_2082_MOESM1_ESM.pdf]

nature chemistry

Supplementary information

https://doi.org/10.1038/s41557-026-02082-0

Generating extended foldamer dye stacks and unravelling their evolving exciton dynamics

In the format provided by the authors and unedited

Table of the contents

1. Materials and Methods 2

2. Synthesis 5

3. 2D NMR Spectroscopy 30

4. Quantum Chemical Calculations 37

5. Steady-State Absorption and Fluorescence Spectroscopy 44

6. Time-Resolved Spectroscopy 58

7. NMR Spectroscopy 74

8. Mass Spectrometry 114

9. References 134

1. Materials and Methods

Chemicals

All commercial chemicals and reagents, unless otherwise stated, were used without further purification. All air or moisture sensitive reactions were carried out under nitrogen atmosphere by standard Schlenk techniques. The solubilizing 2-hexyldecylamine (**A-1**),[1] 1,7-dibromoperylene-3,4:9,10-bis(dicarboximide) (**PBI-Center1**),[2] *N,N´*-di(2-hexyldecyl)-1-bromo-7-(2,2´-biphenol)perylene-3,4:9,10-bis(dicarboximide) (**1**) and **PBI-Center2**[3] were synthesized according to literature known procedures. **PBI-1** has been characterized previously.[3]

Purification

Column chromatography was performed on silica-gel (particle size 0.040-0.063 mm) with freshly distilled solvents as eluents. Thin layer chromatography (TLC) was performed on pre-coated TLC-sheets (ALUGRAM Xtra SIL G/UV254, MACHERY-NAGEL). Recycling gel permeation chromatography (GPC) was performed on a Shimadzu Prominence CBM (LC-20AD Prominence Pump; SPDMA20A Prominence Diode Array Detector) with three preparative columns as stationary phase (Japan Analytical Industries Co., Ltd.; JAIGEL-1H, JAIGEL-2H and JAIGEL-2.5H) and chloroform (HPLC grade, stabilized with 0.1 % ethanol) as eluent with a flow rate of 5.0 mL min^{-1}.

NMR Spectroscopy

^{1}H, ^{13}C and 2D NMR spectra were recorded on Bruker Avance DMX 600 and Avance III HD 400 spectrometers. Chemical shifts δ are given in parts per million (ppm) and J (coupling constants) in Hertz (Hz). For all multiplicities, the following abbreviations were used: s = singlet, d = doublet, dd = doublet of doublets, t = triplet, m = multiplet, br = broad. Solvent signals used for calibration of ^{1}H NMR: in deuterated chloroform ($CDCl_3$) δ = 7.26 ppm, in deuterated 1,1,2,2-tetrachloroethane (TCE-d_2) δ = 6.00 ppm. Solvent signals used for ^{13}C NMR: in $CDCl_3$ δ = 77.2 ppm, in TCE-d_2 δ = 73.8 ppm.

For DOSY experiments the kinematic viscosity of 1,1,2,2-tetrachloroethane was determined with a Schott AVS 360 Micro-Ubbelohde viscosimeter (501 00, Xylem Analytics Germany GmbH), a transparent thermostat (CT 52) and a Schott heating unit (CK 300). Corrected by the Hagenbach-Couette correction. The density of 1,1,2,2-tetrachloroethane at 384 K was extracted from literature and extrapolated.[4, 5] DOSY measurements were not referenced against any standard.

<u>Mass Spectrometry</u>

MALDI-TOF mass spectrometry was performed with a Burker Daltonics ultrafleXtreme mass spectrometer in positive-ion mode using *trans*-2-[3-(4-*tert*-butylphenyl)-2-methyl-2-propenylidene]malononitrile (DCTB) as matrix. The monoisotopic signal was chosen for characterization, except for **PBI-10** and **PBI-14**.

<u>Steady-state absorption and fluorescence spectroscopy</u>

Steady state absorption and fluorescence measurements were conducted with spectroscopy grade solvents. UV/Vis absorption spectra in toluene (Tol), tetrahydrofuran (THF), TCE (1,1,2,2-tetrachloroethane) and benzonitrile (BCN) were measured with a V-770 spectrophotometer equipped with a JASCO PAC-743R Auto Peltier 6/8-cell changer system for temperature control. Standard Hellma quartz glass cuvettes of different path lengths were used. Fluorescence studies (TCE) were carried out on a FLS980 spectrometer from *Edinburgh Instruments*. Relative fluorescence quantum yields were measured by the optical dilution method ($OD < 0.05$) using *N*,*N*-bis(2,6-diisopropylphenyl)-1,6,7,12-tetraphenoxyperylene-3,4:9,10-bis(dicarboximide) (Φ_{fl} = 96 % in chloroform) as standard. Absolut fluorescence quantum yields were measured using a Hamamatsu Ulbricht sphere A9924-01with a continuous Xe lamp and a Hamamatsu photonic multi-channel analyzer C10027 and are reabsorption corrected. Steady-state absorption spectra in toluene (Tol), tetrahydrofuran (THF) and benzonitrile (BCN) were measured on a UV/Vis/NIR spectrometer (Varian, Cary5000). Steady state fluorescence spectra in Tol, THF and BCN were measured on a fluorescence spectrophotometer (Hitachi, F-7000). Fluorescence spectra are spectrally corrected by using correction factor of the fluorescence spectrophotometer.

Time-resolved absorption spectroscopy (fs-TA)

The femtosecond transient absorption spectra were measured with pump-probe spectrometer, which an Optical Parametric Amplifiers (TOPAS-C, Spectra-Physics) pumped by a Ti:sapphire regenerative amplifier system (Spitfire Pro, Spectra-Physics) operating at 10 kHz repetition rate and an optical detection system. The generated OPA pulses had a pulse width of ~ 100 fs and an average power of 80 mW in the range 280–2700 nm which were used as pump pulses. White light continuum (WLC) probe pulses were generated using a sapphire window (8 mm of thickness) by focusing of small portion of the fundamental 800 nm pulses which was picked off by a quartz plate before entering to the OPA. After the measurements, we carefully checked absorption spectra of all compounds to detect if there were artifacts due to degradation and photo-oxidation of samples. HPLC grade solvents were used in all measurements. The three dimensional data sets of ΔA versus time and wavelength were subjected to singular value decomposition and global fitting to obtain the kinetic time constants and their associated spectra using Surface Xplorer software (Ultrafast Systems).

Time-resolved absorption spectroscopy (ns-TA)

Nanosecond transient absorption spectroscopy (ns-TA) experiments were performed using a commercial spectrometer (EOS, Ultrafast Systems). The excitation pulses were generated by a commercial collinear optical parametric amplifier (ORPHEUS, Light Conversion) combined with a second-harmonic generation stage (LYRA-SH, Light Conversion). The a Yb:KGW regenerative amplifier (PHAROS-SP-1.5mJ, Light Conversion, 1030 nm, 600 μJ, 10 kHz, 176 fs) was used as the main source for ns-TA. 2 mm quartz cuvettes were used to carry the samples. Since the triplet-state dynamics of molecules in solution are strongly dependent on the concentration of oxygen molecules dissolved in solution, oxygen was partially removed by degassing with argon gas for 1 hour. All measurements were carried out at room temperature.

Femtosecond broadband fluorescence upconversion spectroscopy (FLUPS, transient fluorescence)

Femtosecond broadband fluorescence up-conversion apparatus[6-10] was used for obtaining the transient fluorescence spectra. A Ti:sapphire laser system (Spectra-Physics, Spitfire) provides 35 fs, 380 μJ pulses at 800 nm with 10 kHz repetition rate. The output beam is divided by beam splitter with the equivalent ratio. A pulse of190 μJ is used to pump a commercial collinear optical parametric amplifier (TOPAS, Light Conversion) which delivers 50 fs, ~ 20 μJ gate pulses (~ 1300 nm) with the vertical polarization. The gate beam passes through a periscope,

which adjusts the height and rotates the vertically polarized pulse to be horizontally polarized. Then, the gate pulse passes a sequence of SF50 prism (55.5°) compressor with optimal separation of 12 cm between the apexes of each prism. Finally, the gate beam is relayed onto the nonlinear crystal by a lens (f = 100 mm, Tc = 2 mm). The pulse energy of the gate beam is attenuated by the neutral density filter to keep a level below 6 μJ. The rest of the fundamental light is used as a source for the tunable homemade optical parametric amplifiers (OPA)system. This homemade OPA system is based on noncollinear phase-matching geometry in a nonlinear crystal(EKSMA, BBO, θ = 32°, φ = 0°, d = 2 mm), which is easily color-tuned by controlling optical delay between white light continuum seed pulses (450−1400 nm) produced by using a sapphire window (d = 2 mm) and visible pump pulses (400 nm) generated by a frequency doubling nonlinear crystal (EKSMA, BBO, θ = 29.2°). The generated visible OPA pulses had a pulse width of ~20 fs and an average power of 20 mW at 10 kHz repetition rate in the range of 480−700 nm with vertical polarization. The visible OPA is compressed by chirped mirror pairs. In order to prevent polarization-dependent signals, the pulse polarization is controlled with a half wave plate to be a magic angle (54.7º) and finally the beam is focused on to a 500 μm thick quartz cuvette containing sample with a lens (f = 300 mm, Tc = 2 mm). The pulse energy is attenuated by the ND filter to keep a level below 60 nJ. Moreover, the cuvette is mounted on a motor-driven stage and continuously moved back and forth to avoid photo-degradation and the thermal lens effect. Collection of the fluorescence is achieved by a reflecting microscope objective lens (Newport). Finally, the collected fluorescence is relayed onto the nonlinear crystal by the off-axis parabolic mirror (Newport, f = 50 mm). The horizontally polarized extraordinary upconverted signal is emitted from the BBO crystal (EKSMA, θ = 40°, φ = 0°, d = 0.5 mm) with type II interaction between fluorescence (o) and gate (e) pulses based on noncollinear geometry (an angle between fluorescence and gate pulses, α = ~ 25º). Unwanted light of horizontal polarization, stemming from the original fluorescence and the pump pulse (or Rayleigh scattered light), is mostly ejected by a wire-gridpolarizer (Moxtec PPL04C). Moreover, the upconverted signals pass a Glan-Taylor calcite polarizer (Thorlabs, GT10-A) in order to eliminate unwanted light of vertical polarization, originating from the remaining original fluorescence. The upconverted signals are imaged dispersion-free onto the entrance slit of a spectrograph (Princeton Instrument, Isoplane® SCT 320) and then the upconverted spectrum is finally registered with a CCD camera (Andor Technology, DV420 BU). The FWHM of the cross-correlation functions between the scattered pump pulse (i.e., 520 nm) and the gate pulse (i.e., 1300 nm) is measured to be 250 fs. The transient fluorescence spectrum at each time delay was recorded

with 1 s CCD exposure time and was averaged by using 10 successively recorded spectra. The chirp due to group velocity dispersion (GVD) was measured based on the instantaneous response of DCM dye in toluene. Photometric correction (from 420 to 850 nm) was applied by using standard dye solutions (1) DCM in toluene, 2) DCM in methanol, and 3) LDS751 in methanol as described in the literature.[10]

Area normalized transient fluorescence spectra

For ANTF, the following equation is used:

$$I(v,t) = \frac{I_{\mathrm{SS}}(v)\sum_{\mathrm{j}}\alpha_{\mathrm{j}}(v)e^{-t/\tau_{\mathrm{j}}(v)}}{\sum_{\mathrm{j}}\alpha_{\mathrm{j}}(v)\tau_{\mathrm{j}}(v)}. \qquad (1)$$

$I_{\mathrm{SS}}(v)$: the steady-state fluorescence (or transient spectra at 500 ps) intensity at v, which is correction factor. $\alpha_{\mathrm{j}}(v)$ and $\tau_{\mathrm{j}}(v)$: the values of the fit parameters.[11]

Time-resolved fluorescence spectroscopy (ns-TF)

Time-resolved fluorescence spectra (ns-TF) were measured with an ICCD detector (PI-MAX4, Princeton Instruments) after photoexcitation using 2.25 eV pump (170 fs) generated through an optical parametric amplifier (OPA, ORPHEUS, Light Conversion) and its second harmonic (SH, LYRA-SH, Light Conversion) module, pumped by a Yb:KGW amplifier (PHAROS-SP-1.5mJ, Light Conversion, 1030 nm, 600 μJ, 10 kHz, 176 fs).

Quantum Chemical Calculations

Geometry optimizations were performed for **PBI-1** to **PBI-5** at the density functional theory (DFT) level, as implemented in the Gaussian 16 program package,[12] using the long-range corrected hybrid density ωB97X-D[13] functional including dispersion correction and the def2-SVP basis set.[14] An implicit solvent simulation for Tol using the polarizable continuum model (PCM) implemented in Gaussian16 was applied. The structures were geometry-optimized followed by frequency calculations to prove the existence of true minima. The solubilizing alkyl chains were replaced by methyl groups to reduce computational effort.

2. Synthesis

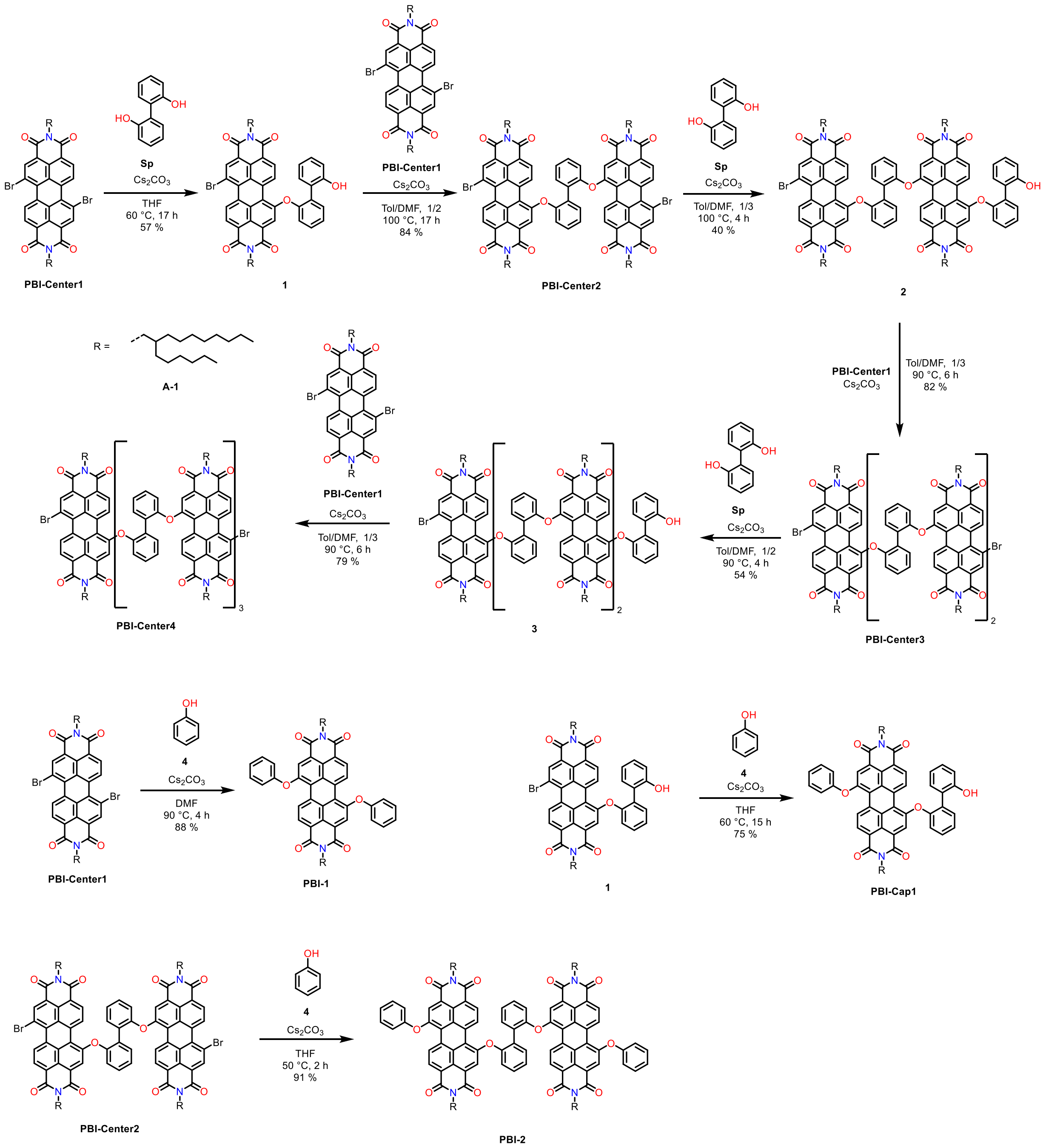

Supplementary Figure 1. Synthesis of central building blocks (**PBI-Center2** to **PBI-Center4**) as well as **PBI-1** and **PBI-2**.

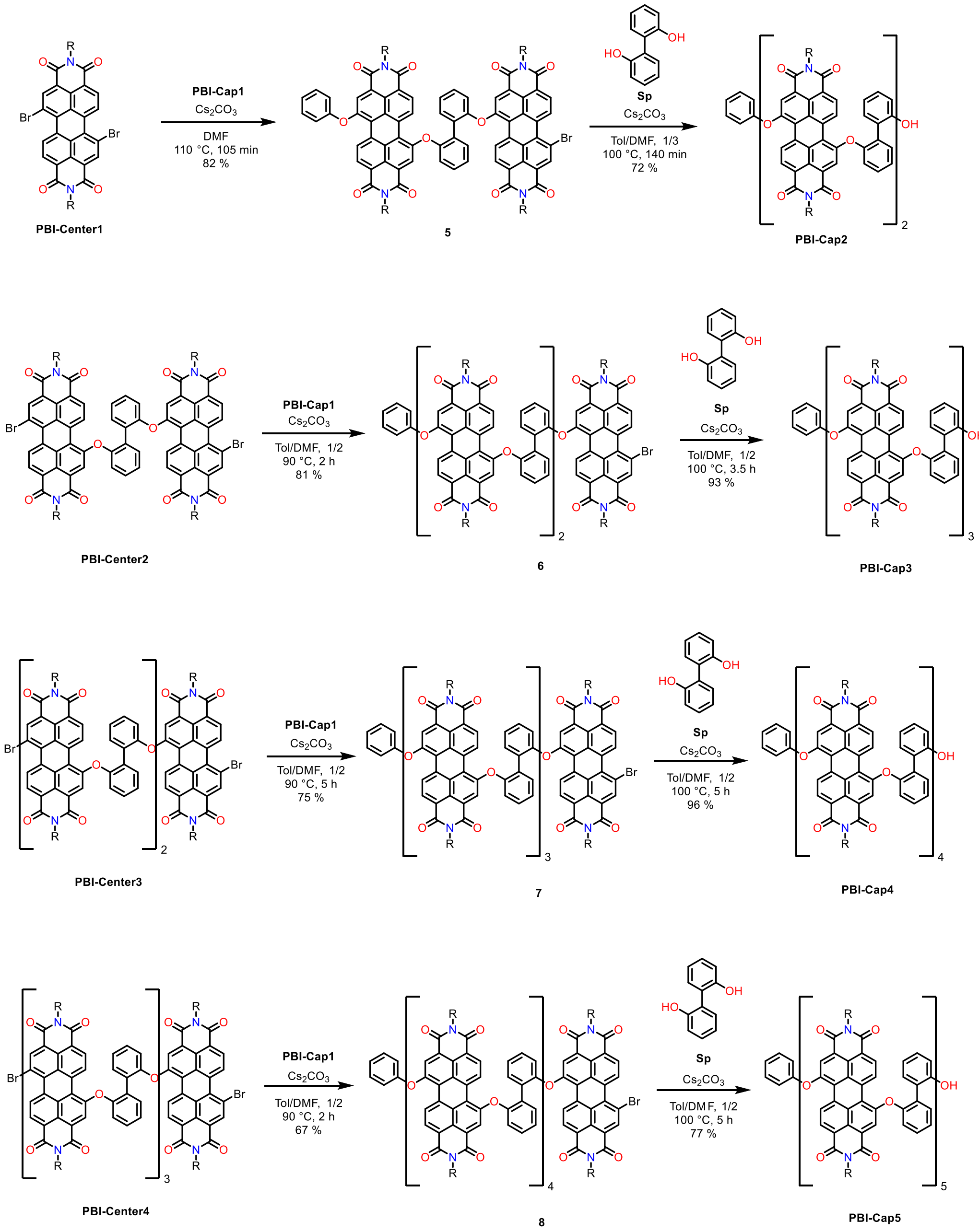

Supplementary Figure 2. Synthesis of end fragments **PBI-Cap2** to **PBI-Cap5**.

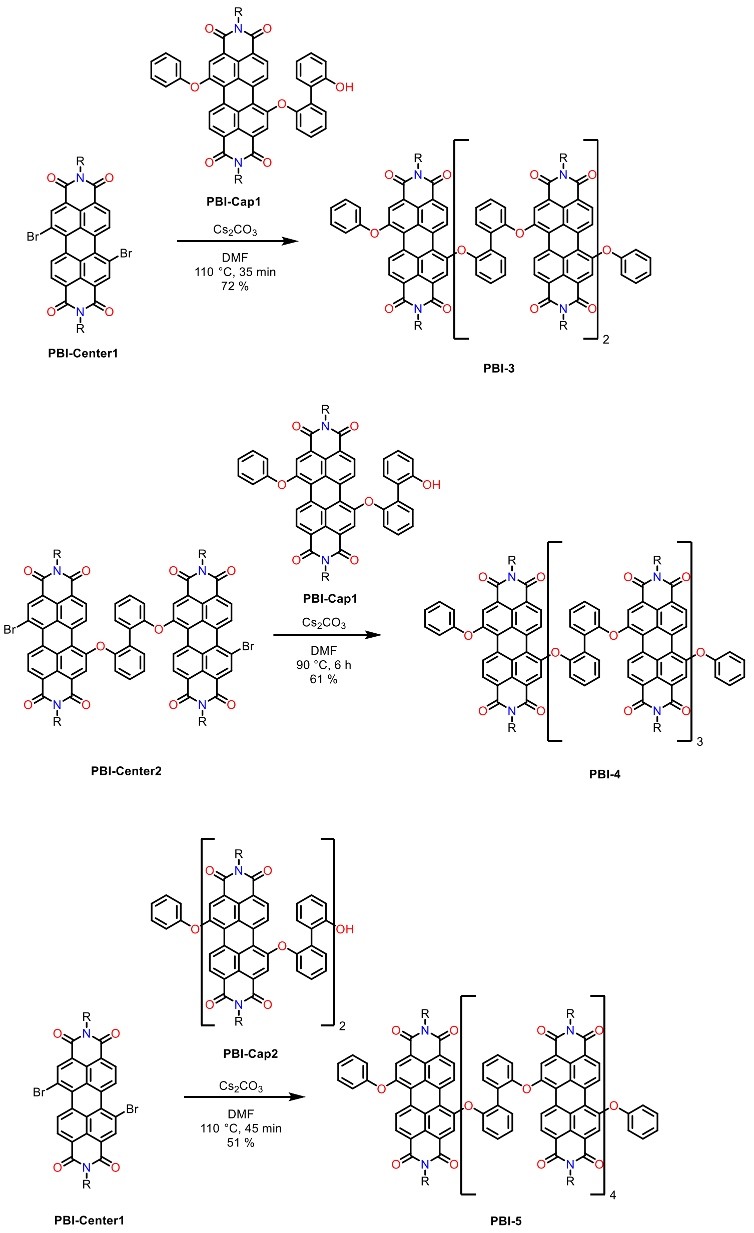

Supplementary Figure 3. Synthesis of bay-linked **PBI-3**, **PBI-4** and **PBI-5**.

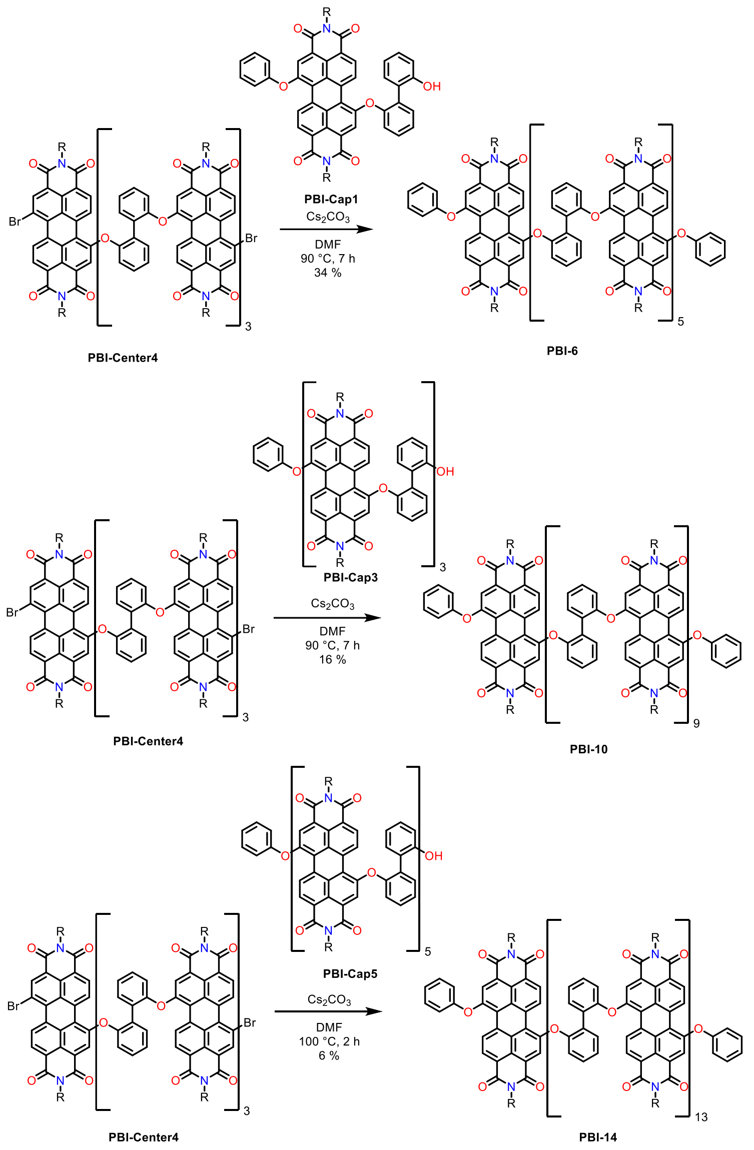

Supplementary Figure 4. Synthesis of bay-linked **PBI-6**, **PBI-10** and **PBI-14**.

Synthesis of **PBI-Center3**

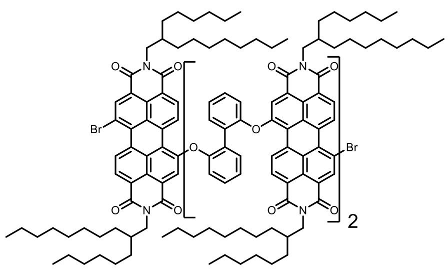

2,2´-Biphenol-bromo-substituted dimer (**2**) (10.0 mg, 4.71 µmol), Cs_2CO_3 (28.1 mg, 86.2 µmol) and *N,N´*-di(2-hexyldecyl)-1,7-dibromoperylene-3,4:9,10-bis(dicarboximide) (**PBI-Center1**) (42.3 mg, 42.4 µmol) were dissolved in dry toluene (10 mL) and stirred at room temperature for 5 min under nitrogen atmosphere. Afterwards, DMF (30 mL) was added and the reaction mixture was stirred at 90 °C for 6 h. After the reaction mixture was cooled to room temperature, 10 % HCl (aq.) (30 mL) was added and the separated aqueous layer was extracted with toluene (3 × 30 mL). The combined organic layers were washed with H_2O (50 mL) and dried over anhydrous $MgSO_4$. After removal of the solvents under reduced pressure, the crude product was purified by column chromatography on silica (CH_2Cl_2) followed by GPC ($CHCl_3$) to give compound **PBI-Center3** (11.7 mg, 3.85 µmol, 82 %) as a dark red solid. ^{1}H NMR (600 MHz, TCE-d_2, 384 K): δ/ppm = 9.60 (d, ^3J = 8.1 Hz, 2H), 9.14 (d, ^3J = 8.3 Hz, 2H), 8.97 (d, ^3J = 8.2 Hz, 2H), 8.93 (s, 2H), 8.52 (d, ^3J = 8.1 Hz, 2H), 8.11 (d, ^3J = 8.3 Hz, 2H), 8.07 – 8.05 (m, 4H), 8.04 – 8.00 (m, 2H), 7.86 – 7.82 (m, 4H), 7.60 – 7.56 (m, 2H), 7.54 – 7.48 (m, 6H), 7.28 – 7.25 (m, 2H), 7.17 – 7.14 (m, 2H), 4.00 (d, ^3J = 7.3 Hz, 4H), 3.78 (d, ^3J = 6.8 Hz, 4H), 3.60 (d, ^3J = 7.1 Hz, 4H), 1.99 – 1.93 (m, 2H), 1.90 – 1.84 (m, 2H), 1.73 – 1.67 (m, 2H), 1.35 – 1.20 (m, 144H), 0.93 – 0.87 (m, 36H). ^{13}C NMR (151 MHz, TCE-d_2, 384 K): δ/ppm = 162.7, 162.4, 162.2, 162.2, 161.9, 155.1, 154.5, 152.5, 152.5, 137.8, 133.5, 133.1, 132.9, 132.8, 132.6, 132.4, 130.8, 130.5, 130.3, 130.2, 129.9, 129.2, 129.0, 128.9, 128.3, 128.2, 128.0, 127.3, 126.8, 125.9, 125.4, 124.3, 124.2, 123.9, 123.1, 123.0, 122.7, 122.6, 122.3, 122.3, 121.6, 121.4, 120.2, 119.6, 119.3, 119.1, 44.7, 44.6, 44.6, 36.6, 36.6, 36.5, 31.8, 31.8, 31.8, 31.7, 31.6, 31.6, 31.6, 31.6, 31.5, 31.5, 31.5, 31.4, 29.8, 29.7, 29.4, 29.4, 29.3, 29.2, 29.2, 29.2, 28.9, 28.9, 28.9, 26.4, 26.3, 26.3, 26.2, 26.2, 26.1, 22.3, 22.3, 22.3, 22.2, 22.2, 13.7, 13.6, 13.6, 13.6. HRMS (MALDI-TOF, positive mode, DCTB in chloroform): m/z calcd. for $C_{192}H_{232}Br_2N_6O_{16}$ [M]$^+$, 3035.5886, found: 3035.5890.

Synthesis of **PBI-Center4**

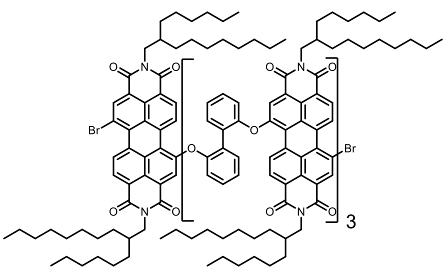

2,2´-Biphenol-bromo-substituted trimer (**3**) (4.50 mg, 1.43 µmol), Cs_2CO_3 (14.7 mg, 21.5 µmol) and *N,N´*-di(2-hexyldecyl)-1,7-dibromoperylene-3,4:9,10-bis(dicarboximide) (**PBI-Center1**) (12.6 mg, 12.6 µmol) were dissolved in dry toluene (5 mL) and stirred at room temperature for 5 min under nitrogen atmosphere. Afterwards, DMF (15 mL) was added and the reaction mixture was stirred at 90 °C for 6 h. After the reaction mixture was cooled to room temperature, 10 % HCl (aq.) (30 mL) was added and the separated aqueous layer was extracted with toluene (3 × 20 mL). The combined organic layers were washed with H_2O (50 mL) and dried over anhydrous $MgSO_4$. After removal of the solvents under reduced pressure, the crude product was purified by column chromatography on silica (CH_2Cl_2) followed by GPC ($CHCl_3$) to give compound **PBI-Center4** (4.59 mg, 1.13 µmol, 79 %) as a dark red solid. ^{1}H NMR (600 MHz, TCE-d_2, 384 K): *δ*/ppm = 9.58 (d, ^3J = 8.2 Hz, 2H), 9.17 (d, ^3J = 8.2 Hz, 2H), 9.11 (d, ^3J = 8.3 Hz, 2H), 8.95 (d, ^3J = 8.1 Hz, 2H), 8.93 (s, 2H), 8.50 (d, ^3J = 8.1 Hz, 2H), 8.12 (d, ^3J = 8.1 Hz, 2H), 8.06 (s, 2H), 8.05 – 8.00 (m, 6H), 7.99 (s, 2H), 7.86 – 7.83 (m, 2H), 7.82 – 7.79 (m, 4H), 7.56 – 7.49 (m, 10H), 7.47 – 7.43 (m, 2H), 7.38 – 7.34 (m, 2H), 7.27 – 7.24 (m, 2H), 7.10 – 7.07 (m, 2H), 4.03 (d, ^3J = 7.3 Hz, 4H), 3.73 (d, ^3J = 6.8 Hz, 4H), 3.61 (d, ^3J = 6.8 Hz, 4H), 3.50 (d, ^3J = 6.0 Hz, 4H), 2.02 – 1.96 (m, 2H), 1.87 – 1.81 (m, 2H), 1.73 – 1.64 (m, 4H), 1.35 – 1.17 (m, 192H), 0.94 – 0.85 (m, 48H). ^{13}C NMR (151 MHz, TCE-d_2, 384 K): *δ*/ppm = 162.7, 162.5, 162.5, 162.5, 162.2, 162.1, 161.9, 161.8, 155.0, 154.6, 154.3, 152.5, 152.4, 152.1, 137.8, 133.4, 133.2, 132.8, 132.7, 132.7, 132.4, 130.6, 130.5, 130.2, 130.1, 130.0, 129.3, 129.2, 129.1, 129.0, 128.9, 128.2, 128.2, 127.9, 127.8, 127.5, 126.9, 125.7, 125.5, 125.4, 124.3, 124.3, 124.2, 123.7, 123.1, 123.0, 122.8, 122.8, 122.7, 122.4, 122.4, 122.3, 122.2, 121.7, 121.6, 121.4, 120.2, 119.7, 119.6, 119.2, 119.1, 44.7, 44.6, 44.5, 44.3, 36.5, 36.5, 31.9, 31.8, 31.7, 31.6, 31.6, 31.6, 31.6, 31.5, 31.5, 31.5, 31.5, 31.4, 31.4, 29.8, 29.8, 29.7, 29.7, 29.4, 29.4, 29.3, 29.3, 29.2, 29.2, 29.2, 29.1, 28.9, 28.9, 28.9, 26.4, 26.4, 26.3, 26.2, 26.2, 26.1, 26.1, 22.3, 22.3, 22.3, 22.2, 22.2, 13.7, 13.6, 13.6, 13.6. HRMS (MALDI-TOF, positive mode, DCTB in chloroform): m/z calcd. for $C_{260}H_{313}Br_2N_8O_{22}$ $[M]^+$, 4057.1981, found: 4057.1936.

Synthesis of 2,2´-biphenol-bromo-substituted dimer **(2)**

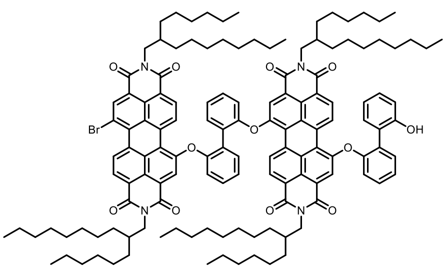

PBI-Center2 (50.0 mg, 24.8 µmol), 2,2´-biphenol (**Sp**) (138 mg, 743 µmol) and Cs_2CO_3 (140 mg, 430 µmol) were dissolved in dry toluene (36 mL) and stirred at room temperature for 5 min under nitrogen atmosphere. Dry DMF (110 mL) was added and the reaction mixture was stirred at 100 °C for 4 h. Afterwards, the reaction mixture was cooled to room temperature, 10 % HCl (aq.) (100 mL) was added and the separated aqueous layer was extracted with toluene (3 × 50 mL). The combined organic layers were washed with H_2O (100 mL) and dried over anhydrous $MgSO_4$. After removal of the solvents under reduced pressure, the crude product was purified by column chromatography on silica (CH_2Cl_2) and GPC ($CHCl_3$). The product was precipitated from CH_2Cl_2 in MeOH to give compound **2** (21.1 mg, 9.94 µmol, 40 %) as a dark red solid. ^{1}H NMR (600 MHz, TCE-d_2, 384 K): δ/ppm = 9.63 (d, ^3J = 8.2 Hz, 1H), 9.39 (d, ^3J = 8.3 Hz, 1H), 9.16 (d, ^3J = 8.3 Hz, 1H), 9.01 (d, ^3J = 8.2 Hz, 1H), 8.95 (s, 1H), 8.55 (d, ^3J = 8.2 Hz, 1H), 8.42 (d, ^3J = 8.3 Hz, 1H), 8.32 (s, 1H), 8.14 (d, ^3J = 8.1 Hz, 1H), 8.10 (s, 1H), 8.06 (s, 1H), 8.03 (d, ^3J = 8.2 Hz, 1H), 7.84 – 7.79 (m, 2H), 7.61 – 7.58 (m, 1H), 7.57 – 7.43 (m, 6H), 7.35 – 7.33 (m, 1H), 7.32 – 7.29 (m, 1H), 7.15 – 7.13 (m, 1H), 7.07 – 7.03 (m, 2H), 6.84 – 6.79 (m, 2H), 5.44 (s, 1H), 4.08 (d, ^3J = 7.3 Hz, 2H), 4.05 (d, ^3J = 7.3 Hz, 2H), 3.78 (d, ^3J = 7.2 Hz, 2H), 3.68 (d, ^3J = 7.3 Hz, 2H), 2.05 – 1.96 (m, 2H), 1.89 – 1.82 (m, 1H), 1.81 – 1.75 (m, 1H), 1.39 – 1.21 (m, 96H), 0.93 – 0.89 (m, 24H). ^{13}C NMR (151 MHz, TCE-d_2, 384 K): δ/ppm = 163.1, 162.9, 162.8, 162.7, 162.2, 162.2, 162.1, 155.0, 154.8, 154.1, 153.3, 152.7, 152.6, 152.5, 138.0, 133.5, 133.1, 132.9, 132.7, 132.7, 132.5, 130.9, 130.5, 130.5, 130.2, 130.1, 129.9, 129.9, 129.8, 129.7, 129.3, 129.2, 129.1, 128.9, 128.3, 127.9, 127.7, 127.0, 125.7, 125.5, 125.3, 124.6, 124.4, 124.4, 124.3, 123.8, 123.4, 123.1, 123.0, 123.0, 122.9, 122.6, 122.6, 122.4, 122.3, 122.0, 121.7, 121.5, 120.3, 120.2, 119.9, 119.7, 119.1, 118.6, 115.8, 44.8, 44.8, 44.6, 36.6, 36.6, 36.5, 36.4, 31.9, 31.8, 31.7, 31.6, 31.6, 31.5, 31.5, 31.5, 31.5, 31.4, 29.8, 29.8, 29.7, 29.4, 29.4, 29.3, 29.3, 29.2, 29.2, 29.2, 29.2, 28.9, 28.9, 26.4, 26.4, 26.3, 26.3, 26.3, 26.2, 26.2, 22.3, 22.3, 22.3, 22.2, 13.6, 13.6, 13.6, 13.6. HRMS (MALDI-TOF, positive mode, DCTB in chloroform): m/z calcd. for $C_{136}H_{161}BrN_4O_{12}$ $[M]^+$, 2121.1289, found: 2121.1317.

Synthesis of 2,2´-biphenol-bromo-substituted trimer **(3)**

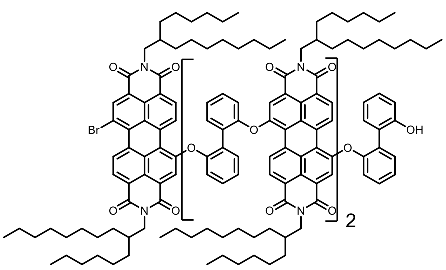

PBI-Center3 (8.00 mg, 2.63 µmol), 2,2´-biphenol (**Sp**) (10.4 mg, 55.9 µmol) and Cs_2CO_3 (10.1 mg, 31.0 µmol) were dissolved in dry toluene (7 mL) and stirred at room temperature for 5 min under nitrogen atmosphere. Dry DMF (15 mL) was added and the reaction mixture was stirred at 90 °C for 4 h. Afterwards, the reaction mixture was cooled to room temperature, 10 % HCl (aq.) (50 mL) was added and the separated aqueous layer was extracted with toluene (3 × 40 mL). The combined organic layers were washed with H_2O (50 mL) and dried over anhydrous $MgSO_4$. After removal of the solvents under reduced pressure, the crude product was purified by column chromatography on silica (CH_2Cl_2) and GPC ($CHCl_3$). The product was precipitated from CH_2Cl_2 in MeOH to give compound **3** (4.50 mg, 1.43 µmol, 54 %) as a dark red solid. ^{1}H NMR (600 MHz, TCE-d_2, 384 K): δ/ppm = 9.60 (d, ^3J = 8.1 Hz, 1H), 9.31 (d, ^3J = 8.3 Hz, 1H), 9.18 (d, ^3J = 8.3 Hz, 1H), 9.16 (d, ^3J = 8.2 Hz, 1H), 9.12 (d, ^3J = 8.3 Hz, 1H), 8.97 (d, ^3J = 8.2 Hz, 1H), 8.93 (s, 1H), 8.52 (d, ^3J = 8.2 Hz, 1H), 8.37 (d, ^3J = 8.3 Hz, 1H), 8.31 (s, 1H), 8.14 – 8.09 (m, 2H), 8.08 – 8.00 (m, 6H), 7.84 – 7.80 (m, 4H), 7.59 – 7.56 (m, 1H), 7.54 – 7.49 (m, 7H), 7.48 – 7.43 (m, 3H), 7.34 – 7.29 (m, 2H), 7.28 – 7.24 (m, 2H), 7.14 – 7.09 (m, 2H), 7.02 – 6.98 (m, 1H), 6.78 – 6.74 (m, 2H), 5.30 (s, 1H), 4.05 – 4.00 (m, 4H), 3.78 – 3.73 (m, 2H), 3.65 – 3.60 (m, 4H), 3.57 – 3.52 (m, 2H), 2.02 – 1.95 (m, 2H), 1.88 – 1.82 (m, 1H), 1.81 – 1.76 (m, 1H), 1.72 – 1.66 (m, 2H), 1.33 – 1.19 (m, 144H), 0.92 – 0.85 (m, 36H). ^{13}C NMR (151 MHz, TCE-d_2, 384 K): δ/ppm = 163.0, 162.9, 162.8, 162.7, 162.6, 162.6, 162.5, 162.2, 162.1, 162.0, 161.9, 155.1, 155.0, 154.7, 154.6, 154.5, 154.4, 153.2, 152.8, 152.5, 152.4, 152.3, 152.3, 148.8, 139.0, 137.8, 133.4, 133.2, 132.9, 132.9, 132.9, 132.8, 132.7, 132.4, 130.8, 130.7, 130.7, 130.6, 130.5, 130.5, 130.2, 130.2, 130.1, 130.0, 129.9, 129.8, 129.6, 129.3, 129.3, 129.2, 129.2, 129.1, 129.0, 128.9, 128.9, 128.8, 128.7, 128.3, 128.2, 128.1, 127.9, 127.7, 127.5, 126.9, 125.8, 125.5, 125.5, 125.4, 125.4, 124.6, 124.4, 124.3, 124.3, 124.3, 123.8, 123.6, 123.4, 123.2, 123.1, 123.1, 123.0, 122.9, 122.8, 122.7, 122.4, 122.4, 122.4, 122.3, 122.3, 121.8, 121.7, 121.6, 121.6, 121.4, 120.3, 120.2, 119.9, 119.6, 119.5, 119.3, 119.2, 119.1, 115.8, 44.7, 44.7, 44.7, 44.6, 44.6, 44.4, 44.4, 44.4, 44.4, 36.6, 36.6, 36.5, 36.5, 36.4, 31.8, 31.8, 31.8, 31.7, 31.7, 31.6, 31.6, 31.6, 31.6, 31.6, 31.6, 31.5, 31.5, 31.5, 31.5, 31.5, 31.4, 31.4, 29.8, 29.8, 29.7, 29.7, 29.4, 29.4, 29.4, 29.3, 29.3, 29.3, 29.2, 29.2, 29.2, 29.2, 29.1, 28.9, 28.9, 28.9, 26.4, 26.4, 26.3, 26.3, 26.2, 26.2, 26.2, 26.1, 26.1, 22.3, 22.3, 22.3, 22.2, 22.2, 22.2, 13.6, 13.6, 13.6, 13.6, 13.6. HRMS (MALDI-TOF, positive mode, DCTB in chloroform): m/z calcd. for $C_{204}H_{241}BrN_6O_{18}$ $[M]^+$, 3141.7305, found: 3141.7402.

Synthesis of bromo-phenoxy-substituted dimer **(5)**

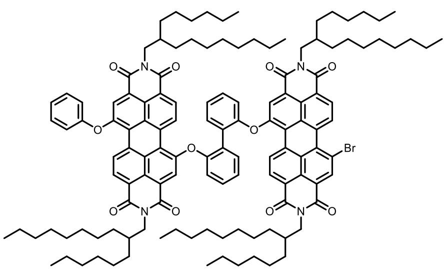

N,N´-Di(2-hexyldecyl)-1-phenoxy-7-(2,2´-biphenol)perylene-3,4:9,10-bis(dicarboximide) (**PBI-Cap1**) (31.3 mg, 28.1 µmol), *N,N´*-di(2-hexyldecyl)-1,7-dibromoperylene-3,4:9,10-bis(dicarboximide) (**PBI-Center1**) (60.9 mg, 61.1 µmol) and Cs_2CO_3 (53.0 mg, 163 µmol) were dissolved in dry DMF (20 mL) and stirred at 110 °C for 105 min under nitrogen atmosphere. After cooling to room temperature, 10 % HCl (aq.) (10 mL) was added and the separated aqueous layer was extracted with toluene (3 × 10 mL). The combined organic layers were washed with H_2O (3 × 10 mL) and dried over anhydrous $MgSO_4$ afterwards. After removal of the solvents under reduced pressure, the crude product was purified by column chromatography on silica gel (CH_2Cl_2/cyclohexane, *v*/*v*, 9/1) and GPC to give compound **5** (46.5 mg, 22.9 µmol, 82 %) as a dark purple solid. ^{1}H NMR (600 MHz, TCE-d_2, 384 K): δ/ppm = 9.64 (d, ^3J = 8.2 Hz, 1H), 9.53 (d, ^3J = 8.3 Hz, 1H), 9.20 (d, ^3J = 8.3 Hz, 1H), 9.01 (d, ^3J = 8.2 Hz, 1H), 8.95 (s, 1H), 8.54 (d, ^3J = 8.2 Hz, 1H), 8.49 (d, ^3J = 8.3 Hz, 1H), 8.37 (s, 1H), 8.15 (d, ^3J = 8.3 Hz, 1H), 8.12 (s, 1H), 8.09 – 8.08 (m, 2H), 7.85 – 7.81 (m, 2H), 7.58 – 7.44 (m, 6H), 7.39 – 7.35 (m, 1H), 7.34 – 7.32 (m, 2H), 7.14 (dd, ^3J = 8.0 Hz, ^4J = 1.2 Hz, 1H), 7.09 (dd, ^3J = 8.0 Hz, ^4J = 1.2 Hz, 1H), 4.08 – 4.06 (m, 4H), 3.79 (d, ^3J = 7.2 Hz, 2H), 3.72 (d, ^3J = 7.3 Hz, 2H), 2.03 – 2.00 (m, 2H), 1.87 – 1.85 (m, 1H), 1.80 – 1.79 (m, 1H), 1.46 – 1.24 (m, 96H), 0.92 – 0.87 (m, 24H). ^{13}C NMR (151 MHz, TCE-d_2, 384 K): δ/ppm = 163.0, 162.8, 162.7, 162.6, 162.6, 162.2, 162.1, 162.1, 155.1, 155.0, 154.9, 154.2, 152.6, 152.5, 137.9, 133.4, 133.1, 132.9, 132.9, 132.8, 132.7, 132.4, 130.5, 130.4, 130.2, 130.1, 130.0, 129.8, 129.4, 129.1, 129.1, 129.1, 129.0, 128.3, 128.2, 128.0, 127.7, 127.0, 125.6, 125.3, 125.0, 124.9, 124.6, 124.3, 123.9, 123.7, 123.6, 123.5, 123.0, 122.9, 122.8, 122.6, 122.5, 122.4, 122.1, 121.8, 119.7, 119.4, 118.9, 118.6, 44.8, 44.8, 44.6, 36.6, 36.6, 36.5, 36.4, 31.9, 31.9, 31.9, 31.8, 31.7, 31.6, 31.6, 31.5, 31.5, 31.5, 31.5, 31.4, 31.4, 29.8, 29.8, 29.7, 29.7, 29.4, 29.3, 29.3, 29.2, 29.2, 29.1, 28.9, 28.9, 28.9, 26.4, 26.4, 26.3, 26.3, 26.3, 26.2, 26.2, 26.2, 22.3, 26.3, 22.2, 22.2, 22.2, 13.6, 13.6, 13.6. HRMS (MALDI-TOF, positive mode, DCTB in chloroform): m/z calcd. for $C_{130}H_{157}BrN_4O_{11}$ $[M]^+$: 2029.1027; found: 2029.1023.

Synthesis of bromo-phenoxy-substituted trimer **(6)**

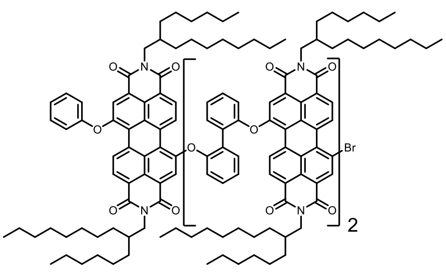

N,N´-Di(2-hexyldecyl)-1-phenoxy-7-(2,2´-biphenol)perylene-3,4:9,10-bis(dicarboximide) (**PBI-Cap1**) (3.00 mg, 2.69 µmol), **PBI-Center2** (17.0 mg, 8.42 µmol) and Cs_2CO_3 (18.5 mg, 56.8 µmol) were dissolved in dry toluene (5 mL) and stirred at room temperature for 5 min under nitrogen atmosphere. Dry DMF (10 mL) was added and the reaction mixture was stirred at 90 °C for 2 h. After cooling to room temperature, 10 % HCl (aq.) (10 mL) was added and the separated aqueous layer was extracted with toluene (3 × 10 mL). The combined organic layers were washed with H_2O (3 × 10 mL) and dried over anhydrous $MgSO_4$. After removal of the solvents under reduced pressure, the crude product was purified by column chromatography on silica gel (CH_2Cl_2) and GPC ($CHCl_3$) to give compound **6** (6.65 mg, 2.18 µmol, 81 %) as a dark purple solid. ^{1}H NMR (600 MHz, TCE-d_2, 384 K): δ/ppm = 9.60 (d, ^3J = 8.2 Hz, 1H), 9.47 (d, ^3J = 8.3 Hz, 1H), 9.20 – 9.17 (m, 2H), 9.12 (d, ^3J = 8.3 Hz, 1H), 8.96 (d, ^3J = 8.2 Hz, 1H), 8.93 (s, 1H), 8.51 (d, ^3J = 8.2 Hz, 1H), 8.43 (d, ^3J = 8.3 Hz, 1H), 8.34 (s, 1H), 8.14 (d, ^3J = 8.3 Hz, 1H), 8.11 (d, ^3J = 7.8 Hz, 1H), 8.09 – 8.05 (m, 3H), 8.04 – 8.00 (m, 3H), 7.86 – 7.80 (m, 4H), 7.57 – 7.46 (m, 10H), 7.37 – 7.32 (m, 2H), 7.31 – 7.27 (m, 3H), 7.17 – 7.15 (m, 1H), 7.13 – 7.10 (m, 1H), 4.01 (d, ^3J = 7.0 Hz, 4H), 3.76 (d, ^3J = 7.0 Hz, 2H), 3.67 (d, ^3J = 6.3 Hz, 2H), 3.62 – 3.56 (m, 4H), 2.01 – 1.93 (m, 2H), 1.88 – 1.82 (m, 1H), 1.81 – 1.76 (m, 1H), 1.75 – 1.66 (m, 2H), 1.34 – 1.18 (m, 144H), 0.93 – 0.85 (m, 36H). ^{13}C NMR (151 MHz, TCE-d_2, 384 K): δ/ppm = 162.9, 162.8, 162.7, 162.7, 162.5, 162.5, 162.2, 162.1, 161.9, 155.1, 155.0, 154.8, 154.6, 154.6, 154.4, 152.5, 152.4, 152.4, 152.3, 137.8, 133.4, 133.2, 133.1, 133.0, 132.9, 132.9, 132.8, 132.7, 132.4, 130.8, 130.7, 130.5, 130.4, 130.2, 130.0, 129.7, 129.5, 129.3, 129.1, 129.1, 129.0, 128.9, 128.2, 128.2, 128.0, 127.9, 127.4, 126.9, 125.8, 125.7, 125.5, 125.4, 125.0, 124.8, 124.5, 124.3, 123.8, 123.7, 123.5, 123.2, 123.1, 123.1, 123.0, 122.9, 122.8, 122.7, 122.5, 122.3, 122.2, 122.1, 121.9, 121.9, 121.7, 121.5, 121.4, 119.6, 119.5, 119.3, 119.2, 44.7, 44.6, 44.5, 44.4, 36.6, 36.5, 36.4, 31.8, 31.7, 31.7, 31.6, 31.6, 31.6, 31.6, 31.5, 31.5, 31.5, 31.5, 31.4, 31.4, 29.8, 29.8, 29.7, 29.7, 29.4, 29.4, 29.4, 29.3, 29.3, 29.2, 29.2, 29.2, 29.1, 28.9, 28.9, 28.9, 26.4, 26.3, 26.3, 26.3, 26.3, 26.2, 26.2, 26.1, 26.1, 26.1, 22.3, 22.3, 13.6, 13.6, 13.6, 13.6. HRMS (MALDI-TOF, positive mode, DCTB in chloroform): m/z calcd. for $C_{198}H_{237}BrN_6O_{17}$ $[M]^+$, 3049.7043, found: 3049.7092.

Synthesis of bromo-phenoxy-substituted tetramer **(7)**

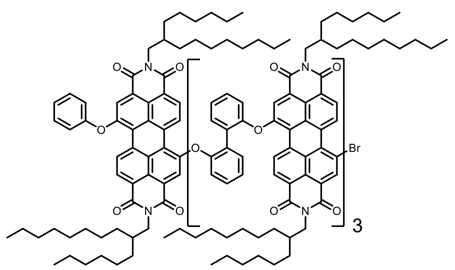

N,N´-Di(2-hexyldecyl)-1-phenoxy-7-(2,2´-biphenol)perylene-3,4:9,10-bis(dicarboximide) (**PBI-Cap1**) (2.50 mg, 2.24 µmol), **PBI-Center3** (20.4 mg, 6.72 µmol) and Cs_2CO_3 (21.2 mg, 65.1 µmol) were dissolved in dry toluene (5 mL) and stirred at room temperature for 5 min under nitrogen atmosphere. Dry DMF (10 mL) was added and the reaction mixture was stirred at 90 °C for 5 h. After cooling to room temperature, 10 % HCl (aq.) (15 mL) was added and the separated aqueous layer was extracted with toluene (3 × 15 mL). The combined organic layers were washed with H_2O (3 × 10 mL) and dried over anhydrous $MgSO_4$. After removal of the solvents under reduced pressure, the crude product was purified by column chromatography on silica gel (CH_2Cl_2) and GPC ($CHCl_3$) to give compound **7** (6.84 mg, 1.68 µmol, 75 %) as a dark purple solid. ^{1}H NMR (600 MHz, TCE-d_2, 384 K): δ/ppm = 9.59 (d, ^3J = 8.1 Hz, 1H), 9.47 (d, ^3J = 8.3 Hz, 1H), 9.18 – 9.14 (m, 4H), 9.12 (d, ^3J = 8.3 Hz, 1H), 8.95 (d, ^3J = 8.2 Hz, 1H), 8.93 (s, 1H), 8.50 (d, ^3J = 8.1 Hz, 1H), 8.43 (d, ^3J = 8.3 Hz, 1H), 8.34 (s, 1H), 8.15 – 7.95 (m, 12H), 7.85 – 7.78 (m, 6H), 7.56 – 7.41 (m, 14H), 7.38 – 7.25 (m, 7H), 7.11 – 7.07 (m, 2H), 4.05 – 4.01 (m, 4H), 3.76 – 3.45 (m, 12H), 2.03 – 1.95 (m, 2H), 1.86 – 1.65 (m, 6H), 1.45 – 1.06 (m, 192H), 0.95 – 0.82 (m, 48H). ^{13}C NMR: (151 MHz, TCE-d_2, 384 K): δ/ppm = 163.1, 163.0, 163.0, 162.9, 162.8, 162.8, 162.7, 162.7, 162.6, 162.6, 162.1, 162.1, 162.0, 162.0, 161.9, 161.9, 155.0, 154.8, 154.8, 154.7, 154.6, 154.5, 154.5, 154.5, 154.4, 153.2, 152.8, 152.4, 152.3, 152.1, 152.1, 133.1, 133.0, 133.0, 133.0, 132.9, 132.9, 132.8, 132.8, 132.7, 132.7, 130.8, 130.5, 130.4, 130.4, 130.4, 130.3, 130.2, 130.2, 130.1, 130.1, 130.0, 130.0, 129.9, 129.9, 129.9, 129.8, 129.8, 129.7, 129.6, 129.6, 129.5, 129.4, 129.3, 129.3, 129.2, 129.1, 129.1, 129.0, 128.9, 128.8, 128.8, 128.8, 128.2, 127.9, 127.9, 127.8, 127.8, 125.6, 125.5, 125.5, 125.4, 125.4, 125.0, 124.9, 124.6, 124.5, 124.5, 124.5, 124.4, 124.4, 124.4, 124.3, 124.3, 123.8, 123.7, 123.7, 123.6, 123.5, 123.4, 123.2, 123.1, 123.1, 123.0, 123.0, 123.0, 122.9, 122.8, 122.3, 122.2, 122.2, 122.2, 122.1, 122.1, 122.1, 121.9, 121.8, 121.8, 121.6, 121.6, 121.5, 121.5, 120.2, 120.2, 119.9, 119.9, 119.6, 119.5, 119.5, 119.4, 119.4, 119.3, 119.3, 119.2, 119.1, 119.1, 115.7, 44.8, 44.7, 44.4, 44.4, 44.3, 44.3, 44.3, 36.6, 36.6, 36.5, 36.5, 36.4, 31.9, 31.8, 31.7, 31.7, 31.7, 31.6, 31.6, 31.6, 31.5, 31.5, 31.5, 31.5, 31.5, 31.4, 31.4, 31.4, 31.4, 29.8, 29.8, 29.8, 29.8, 29.7, 29.7, 29.4, 29.4, 29.4, 29.4, 29.3, 29.3, 29.2, 29.2, 29.2, 29.1, 29.0, 28.9, 28.9, 28.9, 28.9, 28.9, 26.4, 26.4, 26.3, 26.3, 26.3, 26.3, 26.2, 26.2, 26.1, 26.1, 26.1, 22.3, 22.3, 22.2, 22.2, 22.2, 13.7, 13.6, 13.6, 13.6, 13.6. HRMS (MALDI-TOF, positive mode, DCTB in chloroform): m/z calcd. for $C_{266}H_{317}BrN_8O_{23}$ $[M]^+$, 4070.2943 found: 4070.3060.

Synthesis of bromo-phenoxy-substituted pentamer **(8)**

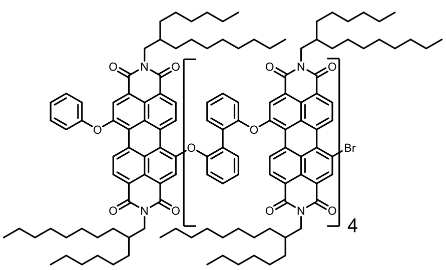

N,N´-Di(2-hexyldecyl)-1-phenoxy-7-(2,2´-biphenol)perylene-3,4:9,10-bis(dicarboximide) (**PBI-Cap1**) (1.30 mg, 1.17 µmol), **PBI-Center4** (14.2 mg, 3.50 µmol) and Cs_2CO_3 (3.80 mg, 11.7 µmol) were dissolved in dry toluene (5 mL) and stirred at room temperature for 5 min under nitrogen atmosphere. Dry DMF (10 mL) was added and the reaction mixture was stirred at 90 °C for 2 h. After cooling to room temperature, 10 % HCl (aq.) (10 mL) was added and the separated aqueous layer was extracted with toluene (3 × 10 mL). The combined organic layers were washed with H_2O (3 × 10 mL) and dried over anhydrous $MgSO_4$. After removal of the solvents under reduced pressure, the crude product was purified by column chromatography on silica gel (CH_2Cl_2) and GPC ($CHCl_3$) to give compound **8** (4.00 mg, 0.78 µmol, 67 %) as a dark purple solid. ^{1}H NMR (600 MHz, TCE-d_2, 384 K): δ/ppm = 9.59 (d, ^3J = 8.1 Hz, 1H), 9.47 (d, ^3J = 8.3 Hz, 1H), 9.18 – 9.10 (m, 7H), 8.95 (d, ^3J = 8.2 Hz, 1H), 8.93 (s, 1H), 8.50 (d, ^3J = 8.1 Hz, 1H), 8.43 (d, ^3J = 8.3 Hz, 1H), 8.34 (s, 1H), 8.14 – 7.94 (m, 9H), 8.03 – 7.94 (m, 7H), 7.84 – 7.76 (m, 8H), 7.55 – 7.41 (m, 18H), 7.36 – 7.33 (m, 1H), 7.32 – 7.23 (m, 8H), 7.11 – 7.07 (m, 2H), 4.05 – 4.00 (m, 4H), 3.76 – 3.63 (m, 4H), 3.62 – 3.57 (m, 4H), 3.55 – 3.45 (m, 8H), 2.02 – 1.95 (m, 2H), 1.86 – 1.64 (m, 8H), 1.36 – 1.06 (m, 240H), 0.95 – 0.81 (m, 60H). ^{13}C NMR (151 MHz, TCE-d_2,384 K): δ/ppm = 163.0, 162.8, 162.7, 162.7, 162.5, 162.5, 162.2, 162.1, 162.1, 162.0, 161.9, 161.9, 161.9, 161.8, 155.0, 155.0, 154.8, 154.5, 154.5, 154.5, 154.4, 154.4, 154.3, 152.5, 152.4, 152.4, 152.3, 152.1, 152.1, 152.0, 137.8, 133.4, 133.2, 133.1, 133.0, 132.9, 132.8, 132.8, 132.8, 132.8, 132.7, 132.7, 132.4, 130.6, 130.5, 130.4, 130.4, 130.3, 130.2, 130.1, 130.1, 130.0, 129.7, 129.5, 129.3, 129.2, 129.1, 129.0, 128.9, 128.2, 128.2, 128.0, 127.9, 127.5, 126.9, 125.7, 125.5, 125.5, 125.4, 125.4, 125.0, 124.9, 124.5, 124.3, 124.3, 124.3, 123.8, 123.7, 123.7, 123.5, 123.2, 123.0, 123.0, 122.9, 122.7, 122.5, 122.4, 122.3, 122.2, 121.9, 121.8, 121.8, 121.7, 121.6, 121.6, 121.6, 121.5, 121.4, 120.2, 119.6, 119.5, 119.4, 119.3, 119.1, 119.1, 44.7, 44.6, 44.5, 44.4, 44.4, 44.3, 36.5, 36.5, 36.4, 31.8, 31.8, 31.7, 31.7, 31.7, 31.6, 31.6, 31.5, 31.5, 31.5, 31.4, 31.4, 31.4, 29.8, 29.8, 29.8, 29.7, 29.7, 29.7, 29.7, 29.4, 29.4, 29.4, 29.3, 29.3, 29.2, 29.2, 29.2, 29.2, 29.2, 29.1, 28.9, 28.9, 28.9, 28.9, 28.9, 28.9, 26.4, 26.4, 26.4, 26.3, 26.3, 26.3, 26.2, 26.2, 26.1, 26.1, 22.3, 22.3, 22.3, 22.3, 22.2, 22.2, 22.2, 13.7, 13.6, 13.6, 13.6, 13.6, 13.6. HRMS (MALDI-TOF, positive mode, DCTB in chloroform): m/z calcd. for $C_{334}H_{397}BrN_{10}O_{29}$ [M]$^+$, 5090.9075, found: 5090.9158.

Synthesis of *N,N´*-di(2-hexyldecyl)-1-phenoxy-7-(2,2´-biphenol)perylene-3,4:9,10-bis(dicarboximide) **(PBI-Cap1)**

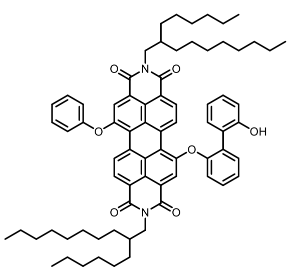

N,N´-Di(2-hexyldecyl)-1-bromo-7-(2,2´-biphenol)perylene-3,4:9,10-bis(dicarboximide) (**1**) (410 mg, 0.37 mmol), Cs_2CO_3 (1.22 g, 3.74 mmol) and phenol (**4**) (351 mg, 3.73 mmol) were dissolved in dry THF (10 mL) and stirred at 60 °C for 15 h under nitrogen atmosphere. After cooling to room temperature, H_2O (30 mL) was added and the separated aqueous layer was extracted with CH_2Cl_2 (3 × 20 mL). The combined organic layers were washed with H_2O (3 × 30 mL), dried over anhydrous $MgSO_4$ and the solvents were removed under reduced pressure. The crude product was purified by column chromatography on silica gel (CH_2Cl_2) and precipitation from CH_2Cl_2 in MeOH to give the compound **PBI-Cap1** (313 mg, 0.28 mmol, 75 %) as a dark red solid. ^{1}H NMR (400 MHz, $CDCl_3$, 295 K): *δ*/ppm = 9.25 (d, ^3J = 8.4 Hz, 1H), 9.20 (d, ^3J = 8.4 Hz, 1H), 8.40 (d, ^3J = 8.4 Hz, 1H), 8.39 (d, ^3J = 8.4 Hz, 1H), 8.23 (s, 1H), 8.09 (s, 1H), 7.52 – 7.48 (m, 1H), 7.45 – 7.41 (m, 2H), 7.36 – 7.30 (m, 3H), 7.25 – 7.22 (m, 1H), 7.18 – 7.12 (m, 1H), 7.08 – 7.04 (m, 2H), 6.94 – 6.83 (m, 3H), 5.95 (br, 1H), 4.04 (d, ^3J = 7.3 Hz, 2H), 4.01 (d, ^3J = 7.3 Hz, 2H), 1.96 – 1.88 (m, 2H), 1.36 – 1.22 (m, 48H), 0.85 – 0.81 (m, 12H). ^{13}C NMR (101 MHz, $CDCl_3$, 295 K): *δ*/ppm = 160.6, 160.5, 159.8, 159.8, 151.7, 151.6, 151.4, 150.5, 149.9, 129.6, 129.5, 129.4, 127.9, 127.3, 127.0, 126.8, 126.7, 126.5, 126.3, 125.9, 125.4, 125.3, 122.0, 121.8, 121.4, 121.3, 120.9, 120.5, 120.4, 120.2, 120.2, 119.9, 118.6, 118.5, 117.2, 116.2, 115.6, 112.5, 47.6, 41.5, 41.4, 33.4, 33.4, 28.6, 28.6, 28.5, 26.8, 26.5, 26.3, 26.1, 26.1, 23.2, 19.4, 10.9, 10.9. HRMS (MALDI-TOF, positive mode, DCTB in chloroform): m/z calcd. for $C_{74}H_{86}N_2O_7$ $[M]^+$: 1114.6430; found: 1114.6406.

Synthesis of 2,2´-biphenol-phenoxy-substituted dimer **(PBI-Cap2)**

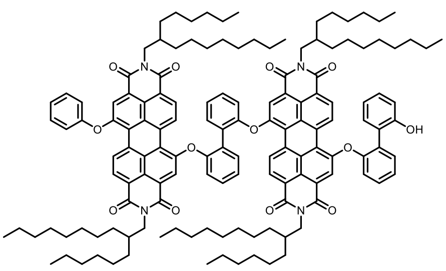

Bromo-phenoxy-substituted dimer (**5**) (16.9 mg, 8.32 µmol), 2,2´-biphenol (**Sp**) (68.7 mg, 369 µmol) and Cs_2CO_3 (60.2 mg, 185 µmol) were dissolved in dry toluene (5 mL) and stirred at room temperature for 20 min under nitrogen atmosphere. Dry DMF (15 mL) was added and the reaction mixture was stirred at 100 °C for 140 min. Afterwards, the reaction mixture was cooled to room temperature, 10 % HCl (aq.) (10 mL) was added and the separated aqueous layer was extracted with toluene (3 × 15 mL). The combined organic layers were washed with H_2O (10 mL) and dried over anhydrous $MgSO_4$. After removal of the solvent under reduced pressure, the crude product was purified by column chromatography on silica (CH_2Cl_2) and GPC ($CHCl_3$). The product was precipitated from CH_2Cl_2 in MeOH to give compound **PBI-Cap2** (12.8 mg, 5.99 µmol, 72 %) as a dark red solid. ^{1}H NMR (600 MHz, TCE-d_2, 384 K): δ/ppm = 9.46 (d, ^3J = 8.4 Hz, 1H), 9.34 (d ^3J = 8.3 Hz, 1H), 9.22 (d, ^3J = 8.3 Hz, 1H), 9.19 (d, ^3J = 8.3 Hz, 1H), 8.43 (d, ^3J = 8.3 Hz, 1H), 8.38 (d, ^3J = 8.3 Hz, 1H), 8.35 (s, 1H), 8.32 (s, 1H), 8.16 (d, ^3J = 8.3 Hz, 1H), 8.12 (d, ^3J = 8.2 Hz, 1H), 8.08 (s, 1H), 8.07 (s, 1H), 7.83 – 7.81 (m, 2H), 7.59 – 7.43 (m, 9H), 7.38 – 7.35 (m, 1H), 7.33 – 7.28 (m, 4H), 7.16 – 7.10 (m, 2H), 7.05 – 7.00 (m, 1H), 6.80 – 6.78 (m, 2H), 4.07 (d, ^3J = 7.4 Hz, 2H), 4.05 (d, ^3J = 7.3 Hz, 2H), 3.67 – 3.65 (m, 4H), 2.03 – 1.99 (m, 2H), 1.82 – 1.77 (m, 2H), 1.40 – 1.23 (m, 96H), 0.92 – 0.86 (m, 24H). ^{13}C NMR (151 MHz, TCE-d_2, 384 K): δ/ppm = 163.1, 162.9, 162.9, 162.8, 162.8, 162.2, 162.1, 155.0, 154.9, 154.7, 154.6, 154.3, 153.3, 152.7, 152.5, 152.4, 133.1, 133.0, 132.9, 132.9, 132.9, 132.8, 132.7, 130.9, 130.4, 130.4, 130.2, 130.1, 129.9, 129.8, 129.8, 129.3, 129.2, 129.1, 129.0, 128.9, 128.8, 128.8, 128.2, 128.0, 127.9, 125.5, 125.4, 125.0, 124.9, 124.6, 124.5, 124.5, 124.4, 123.8, 123.7, 123.6, 123.4, 123.2, 123.1, 123.0, 123.0, 122.5, 122.4, 121.9, 121.9, 121.6, 120.3, 120.2, 119.8, 119.4, 119.3, 119.1, 119.0, 115.8, 53.2, 44.8, 44.7, 44.4, 36.6, 36.4, 36.4, 31.9, 31.8, 31.8, 31.7, 31.6, 31.6, 31.5, 31.5, 31.5, 31.4, 31.4, 29.8, 29.8, 29.7, 29.7, 29.4, 29.4, 29.3, 29.3, 29.2, 29.2, 29.1, 28.9, 28.9, 26.4, 26.3, 26.3, 26.3, 26.2, 26.2, 26.2, 22.3, 22.3, 22.2, 13.6, 13.6, 13.6. HRMS (MALDI-TOF, positive mode, DCTB in chloroform): m/z calcd. for $C_{142}H_{166}N_4O_{13}$ $[M]^+$: 2135.2446; found: 2133.2418.

Synthesis of 2,2´-biphenol-phenoxy-substituted trimer **(PBI-Cap3)**

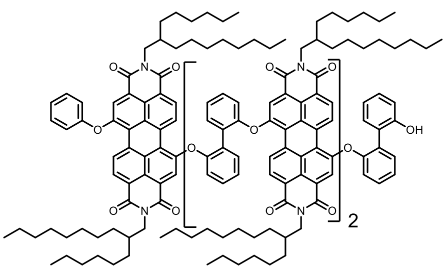

Bromo-phenoxy-substituted trimer (**6**) (196 mg, 64.2 µmol), 2,2'-biphenol (**Sp**) (540 mg, 2.90 mmol) und Cs_2CO_3 (400 mg, 1.23 mmol) were dissolved in dry toluene (100 mL) and stirred at room temperature for 20 min under nitrogen atmosphere. Dry DMF (200 mL) was added and the reaction mixture was stirred at 100 °C for 3.5 h. After cooling to room temperature, 10 % HCl (aq.) (300 mL) was added and the separated aqueous layer was extracted with toluene (3 × 70 mL). The combined organic layers were washed with H_2O (3 × 100 mL) and dried over anhydrous $MgSO_4$. After removal of the solvent under reduced pressure the crude product was purified by column chromatography on silica gel (CH_2Cl_2), GPC ($CHCl_3$ and precipitation from CH_2Cl_2 in MeOH to give compound **PBI-Cap3** (189 mg, 59.8 µmol, 93 %) as a dark purple solid. ^{1}H NMR (600 MHz, TCE-d_2, 384 K): δ/ppm = 9.47 (d, ^3J = 8.3 Hz, 1H), 9.33 (d, ^3J = 8.3 Hz, 1H), 9.21 – 9.15 (m, 4H), 8.45 (d, ^3J = 8.3 Hz, 1H), 8.37 (d, ^3J = 8.3 Hz, 1H), 8.35 (s, 1H), 8.32 (s, 1H), 8.14 – 8.07 (m, 5H), 8.06 (s, 1H), 8.02 (s, 1H), 7.99 (s, 1H), 7.85 – 7.80 (m, 4H), 7.58 – 7.48 (m, 10H), 7.48 – 7.43 (m, 3H), 7.37 – 7.30 (m, 4H), 7.30 – 7.27 (m, 2H), 7.27 – 7.25 (m, 1H), 7.13 – 7.09 (m, 2H), 7.02 – 6.98 (m, 1H), 6.79 – 6.74 (m, 2H), 5.36 (s, 1H), 4.08 – 4.00 (m, 4H), 3.70 – 3.53 (m, 8H), 2.04 – 1.95 (m, 2H), 1.83 – 1.75 (m, 2H), 1.75 – 1.67 (m, 2H), 1.36 – 1.17 (m, 144H), 0.93 – 0.84 (m, 36H). ^{13}C NMR (151 MHz, TCE-d_2, 384 K): δ/ppm = 163.1, 163.0, 162.9, 162.8, 162.8, 162.7, 162.6, 162.1, 162.0, 161.9, 155.0, 154.8, 154.7, 154.6, 154.5, 154.4, 153.2, 152.8, 152.4, 152.3, 152.3, 152.3, 133.1, 133.0, 132.9, 132.8, 132.8, 132.8, 132.7, 130.8, 130.5, 130.5, 130.4, 130.2, 130.2, 130.1, 130.1, 130.1, 129.9, 129.8, 129.7, 129.6, 129.5, 129.4, 129.3, 129.2, 129.0, 128.9, 128.8, 128.7, 128.2, 128.0, 127.9, 127.9, 127.8, 125.6, 125.5, 125.4, 125.0, 124.9, 124.6, 124.5, 124.4, 124.4, 124.3, 123.8, 123.7, 123.5, 123.4, 123.2, 123.1, 123.0, 123.0, 122.9, 122.9, 122.8, 122.3, 122.2, 122.0, 121.9, 121.8, 121.6, 121.5, 120.3, 119.9, 119.6, 119.5, 119.3, 119.2, 115.8, 44.7, 44.7, 44.5, 44.4, 44.3, 36.6, 36.6, 36.5, 36.4, 31.9, 31.8, 31.8, 31.7, 31.7, 31.6, 31.6, 31.6, 31.6, 31.5, 31.5, 31.5, 31.5, 31.4, 29.8, 29.8, 29.8, 29.7, 29.4, 29.4, 29.4, 29.4, 29.3, 29.2, 29.2, 29.2, 29.1, 28.9, 28.9, 28.9, 26.4, 26.4, 26.3, 26.3, 26.3, 26.3, 26.2, 26.2, 26.2, 26.2, 26.1, 26.1, 22.3, 22.3, 22.3, 22.3, 22.3, 22.2, 22.2, 13.7, 13.6, 13.6, 13.6. HRMS (MALDI-TOF, positive mode, DCTB in chloroform): m/z calcd. for $C_{210}H_{246}N_6O_{19}$, $[M]^+$ 3155.8463, found: 3155.8534.

Synthesis of 2,2´-biphenol-phenoxy-substituted tetramer **(PBI-Cap4)**

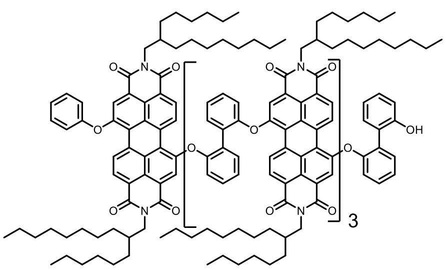

Bromo-phenoxy-substituted tetramer (**7**) (139 mg, 34.1 µmol), 2,2‘-biphenol (**Sp**) (286 mg, 1.54 mmol) und Cs_2CO_3 (216 mg, 663 µmol) were dissolved in dry toluene (75 mL) and stirred at room temperature for 20 min under nitrogen atmosphere. Afterwards, dry DMF (150 mL) was added and the reaction mixture was stirred at 100 °C for 5 h After cooling to room temperature, 10 % HCl (aq.) (225 mL) was added and the separated aqueous layer was extracted with toluene (3 × 70 mL). The combined organic layers were washed with H_2O (3 × 100 mL) and dried over anhydrous $MgSO_4$. After removal of the solvents under reduced pressure, the crude product was purified by column chromatography on silica gel (CH_2Cl_2), GPC ($CHCl_3$) and precipitation from CH_2Cl_2 in MeOH to give compound **PBI-Cap4** (137 mg, 32.8 µmol, 96 %) as a dark purple solid. ^{1}H NMR (600 MHz, TCE-d_2, 384 K): δ/ppm = 9.47 (d, ^3J = 8.3 Hz, 1H), 9.33 (d, ^3J = 8.2 Hz, 1H), 9.19 – 9.13 (m, 6H), 8.44 (d, ^3J = 8.3 Hz, 1H), 8.37 (d, ^3J = 8.2 Hz, 1H), 8.34 (s, 1H), 8.31 (s, 1H), 8.13 – 8.03 (m, 8H), 8.01 – 7.96 (m, 4H), 7.84 – 7.76 (m, 6H), 7.58 – 7.39 (m 17H), 7.37 – 7.23 (m, 9H), 7.11 – 7.04 (m, 2H), 7.01 – 6.96 (m, 1H), 6.77 – 6.73 (m, 2H), 5.37 (s, 1H), 4.08 – 4.00 (m, 4H), 3.69 – 3.46 (m, 12H), 2.04 – 1.96 (m, 2H), 1.81 – 1.65 (m, 6H), 1.38 – 1.07 (m, 192H), 0.95 – 0.81 (m, 48H). ^{13}C NMR (151 MHz, TCE-d_2, 384 K): δ/ppm = 163.1, 163.0, 162.9, 162.8, 162.8, 162.7, 162.7, 162.6, 162.5, 162.1, 162.0, 162.0, 161.9, 155.0, 154.8, 154.7, 154.5, 154.5, 154.4, 153.2, 152.8, 152.4, 152.3, 152.1, 152.1, 133.1, 133.0, 133.0, 132.9, 132.9, 132.8, 132.7, 130.8, 130.5, 130.4, 130.4, 130.3, 130.2, 130.2, 130.1, 130.0, 129.9, 129.8, 129.7, 129.6, 129.5, 129.4, 129.3, 129.1, 129.0, 128.9, 128.8, 128.8, 128.2, 127.9, 127.9, 127.8, 125.5, 125.4, 125.4, 125.0, 124.9, 124.6, 124.5, 124.4, 124.4, 124.3, 124.3, 123.8, 123.7, 123.5, 123.4, 123.2, 123.1, 123.1, 123.0, 122.9, 122.9, 122.2, 122.0, 121.9, 121.8, 121.6, 121.6, 121.5, 120.2, 119.9, 119.6, 119.5, 119.4, 119.3, 119.1, 115.7, 44.7, 44.4, 44.4, 44.3, 44.3, 44.2, 36.6, 36.5, 36.5, 36.4, 31.9, 31.8, 31.7, 31.7, 31.6, 31.6, 31.5, 31.5, 31.5, 31.5, 31.5, 31.4, 31.4, 31.4, 31.4, 29.8, 29.8, 29.8, 29.7, 29.7, 29.4, 29.4, 29.4, 29.4, 29.3, 29.3, 29.2, 29.2, 29.2, 29.1, 28.9, 28.9, 28.9, 28.9, 28.9, 28.9, 26.4, 26.4, 26.3, 26.3, 26.3, 26.3, 26.2, 26.2, 26.1, 26.1, 22.3, 22.3, 22.3, 22.2, 22.2, 22.2, 13.7, 13.6, 13.6, 13.6, 13.6. HRMS (MALDI-TOF, positive mode, DCTB in chloroform): m/z calcd. for $C_{278}H_{326}N_8O_{25}$ [M]$^+$, 4176.4479, found: 4176.4356.

Synthesis of 2,2´-biphenol-phenoxy-substituted pentamer **(PBI-Cap5)**

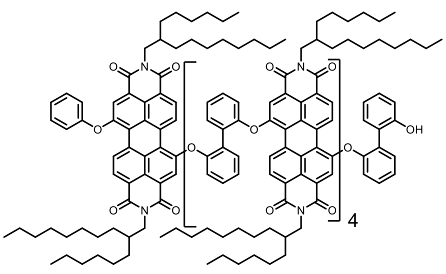

Bromo-phenoxy-substituted pentamer (**8**) (26.0 mg, 5.10 µmol), 2,2´-biphenol (**Sp**) (61.1 mg, 328 µmol) and Cs_2CO_3 (45.5 mg, 140 µmol) were dissolved in dry toluene (14 mL) and stirred at room temperature for 10 min under nitrogen atmosphere. Dry DMF (28 mL) was added and the reaction mixture was stirred at 100 °C for 5 h. After cooling to room temperature, 10 % HCl (aq.) (225 mL) was added and the separated aqueous layer was extracted with toluene (3 × 70 mL). The combined organic layers were washed with H_2O (3 × 100 mL) and dried over anhydrous $MgSO_4$. After removal of the solvents under reduced pressure, the crude product was purified by column chromatography on silica gel (CH_2Cl_2), GPC ($CHCl_3$) and precipitation from CH_2Cl_2 in MeOH to give compound **PBI-Cap5** (20.0 mg, 3.85 µmol, 77 %) as a dark purple solid. ^{1}H NMR (600 MHz, TCE-d_2, 384 K): δ/ppm = 9.47 (d, ^3J = 8.2 Hz, 1H), 9.32 (d ^3J = 8.3 Hz, 1H), 9.18 – 9.13 (m, 8H), 9.43 (d, ^3J = 8.2 Hz, 1H), 8.38 (d, ^3J = 8.3 Hz, 1H), 8.34 (s, 1H), 8.31 (s, 1H), 8.12 – 8.04 (m, 10H), 8.00 – 7.96 (m, 6H), 7.83 – 7.76 (m, 8H), 7.58 – 7.55 (m, 1H), 7.54 – 7.39 (m, 21H), 7.35 – 7.33 (m, 1H), 7.32 – 7.24 (m, 9H), 7.10 – 7.06 (m, 1H), 7.00 – 6.96 (m, 1H), 6.76 – 6.73 (m, 2H), 5.33 (s, 1H), 4.07 – 4.00 (m, 4H), 3.66 – 3.56 (m, 8H), 3.55 – 3.48 (m, 8H), 2.03 – 1.96 (m, 2H), 1.81 – 1.75 (m, 2H), 1.74 – 1.66 (m, 6H), 1.33 – 1.23 (m, 240H), 0.92 – 0.82 (m, 60H). ^{13}C NMR (151 MHz, TCE-d_2, 384 K): δ/ppm = 163.1, 163.0, 162.9, 162.8, 162.8, 162.7, 162.7, 162.7, 162.5, 162.1, 162.1, 162.0, 162.0, 161.9, 161.9, 161.9, 155.0, 154.8, 154.7, 154.5, 154.5, 154.5, 154.4, 154.4, 154.4, 153.2, 152.8, 152.4, 152.3, 152.1, 152.1, 133.1, 133.0, 133.0, 132.9, 132.9, 132.8, 132.8, 132.8, 132.7, 132.7, 130.8, 130.5, 130.4, 130.3, 130.2, 130.2, 130.1, 130.0, 129.9, 129.8, 129.7, 129.6, 129.5, 129.4, 129.3, 129.3, 129.1, 129.0, 128.9, 128.8, 128.8, 128.7, 127.9, 127.9, 127.9, 127.8, 125.5, 125.4, 125.4, 125.0, 124.9, 124.7, 124.6, 124.5, 124.4, 124.3, 124.3, 124.2, 123.8, 123.7, 123.4, 123.2, 123.1, 123.1, 123.0, 122.9, 122.9, 122.9, 122.2, 122.1, 121.9, 121.8, 121.7, 121.6, 121.6, 121.5, 120.2, 119.9, 119.6, 119.5, 119.4, 119.4, 119.3, 119.1, 116.4, 116.2, 116.0, 115.7, 44.7, 44.7, 44.4, 44.4, 44.3, 44.3, 36.6, 36.6, 36.5, 36.5, 36.4, 31.8, 31.7, 31.7, 31.6, 31.6, 31.5, 31.5, 31.5, 31.5, 31.4, 31.4, 31.4, 29.8, 29.8, 29.8, 29.8, 29.7, 29.7, 29.7, 29.4, 29.4, 29.4, 29.3, 29.3, 29.3, 29.2, 29.2, 29.2, 29.2, 29.1, 28.9, 28.9, 28.9, 28.9, 28.9, 28.9, 26.4, 26.4, 26.3, 26.3, 26.3, 26.2, 26.2, 26.2, 26.2, 26.1, 26.1, 22.3, 22.3, 22.3, 22.2, 22.2, 22.2, 22.2, 13.6, 13.6, 13.6, 13.6, 13.6, 13.6. HRMS (MALDI-TOF, positive mode, DCTB in chloroform): m/z calcd. for $C_{346}H_{406}N_{10}O_{31}$ $[M]^+$: 5197.0495; found: 5197.0501.

Synthesis of **PBI-1**

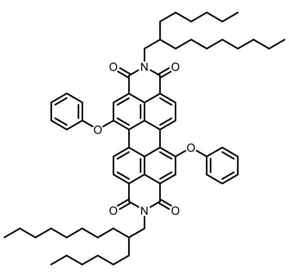

N,N´-di(2-hexyldecyl)-1,7-dibromoperylene-3,4:9,10-bis(dicarboximide) (**PBI-Center1**) (9.60 mg, 9.63 µmol), Cs_2CO_3 (64.9 mg, 199 µmol) and phenol (**4**) (14.5 mg, 154 µmol) were suspended in dry DMF (10 mL) and the reaction mixture was stirred at 90 °C overnight under nitrogen atmosphere. After cooling to room temperature, 10 % HCl (aq.) (30 mL) was added, the precipitate was filtrated and washed with H_2O (100 mL). The solid residue was dissolved in CH_2Cl_2 (30 mL) and dried over anhydrous $MgSO_4$. After removal of the solvents under reduced pressure, the crude product was purified by column chromatography on silica gel (CH_2Cl_2), GPC ($CHCl_3$) and precipitation from CH_2Cl_2 in MeOH to give **PBI-1** (9.00 mg, 8.79 µmol, 88 %) as a dark purple solid. ^1H NMR (400 MHz, TCE-d_2, 295 K): δ/ppm = 9.55 (d, ^3J = 8.4 Hz, 2H), 8.56 (d, ^3J = 8.4 Hz, 2H), 8.26 (s, 2H), 7.54 – 7.48 (m, 4H), 7.35 – 7.30 (m, 2H), 7.24 – 7.20 (m, 4H), 4.05 (d, ^3J = 7.1 Hz, 4H), 1.98 – 1.92 (m, 2H), 1.39 – 1.22 (m, 48H), 0.85 – 0.82 (m, 12H).

^1H NMR data is consistent with that reported in literature.[3]

Synthesis of **PBI-2**

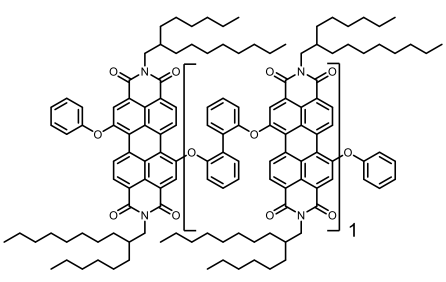

PBI-Center2 (10.9 mg, 5.39 µmol), Cs_2CO_3 (39.1 mg, 120 µmol) and phenol (**4**) (9.50 mg, 101 µmol) were suspended in dry DMF (10 mL) and the reaction mixture was stirred at 90 °C overnight under nitrogen atmosphere. After cooling to room temperature, 10 % HCl (aq.) (30 mL) was added, the precipitate was filtrated and washed with H_2O (100 mL). The solid residue was dissolved in CH_2Cl_2 (30 mL) and dried over anhydrous $MgSO_4$. After removal of the solvent under reduced pressure the crude product was purified by column chromatography on silica gel (CH_2Cl_2), GPC ($CHCl_3$) and precipitation from CH_2Cl_2 in MeOH to give **PBI-2** (8.71 mg, 4.26 µmol, 86 %) as a dark purple solid. ^{1}H NMR (600 MHz, TCE-d_2, 384 K): δ/ppm = 9.49 (d, ^3J = 8.3 Hz, 2H), 9.22 (d, ^3J = 8.3 Hz, 2H), 8.46 (d, ^3J = 8.3 Hz, 2H), 8.35 (s, 2H), 8.16 (d, ^3J = 8.3 Hz, 2H), 8.10 (s, 2H), 7.84 (dd, ^3J = 7.7 Hz, ^4J = 1.9 Hz, 2H), 7.57 – 7.47 (m, 8H), 7.38 – 7.34 (m, 2H), 7.33 – 7.30 (m, 4H), 7.14 (dd, ^3J = 8.0 Hz, ^4J = 1.2 Hz, 2H), 4.06 (d, ^3J = 7.3 Hz, 4H), 3.70 (d, ^3J = 7.2 Hz, 4H), 2.04 – 1.98 (m, 2H), 1.84 – 1.77 (m, 2H), 1.44 – 1.19 (m, 96H), 0.92 – 0.86 (m, 24H). ^{13}C NMR (151 MHz, TCE-d_2, 384 K): δ/ppm = 163.0, 162.8, 162.7, 162.1, 155.0, 154.9, 154.4, 152.5, 133.0, 132.9, 130.4, 130.3, 130.1, 129.8, 129.5, 129.1, 129.0, 128.2, 128.0, 125.5, 125.0, 124.9, 124.5, 123.8, 123.6, 123.5, 123.3, 123.1, 122.6, 122.0, 121.8, 119.4, 118.9, 44.8, 44.5, 36.6, 36.4, 31.9, 31.8, 31.6, 31.5, 31.5, 31.4, 29.8, 29.7, 29.4, 29.3, 29.2, 29.1, 28.9, 28.9, 26.4, 26.3, 26.3, 26.2, 22.3, 22.3, 22.3, 22.2, 13.6, 13.6, 13.6. HRMS (MALDI-TOF, positive mode, DCTB in chloroform): m/z calcd. for $C_{136}H_{162}N_4O_{12}$ $[M]^+$: 2043.2184; found: 2043.2181.

Synthesis of **PBI-3**

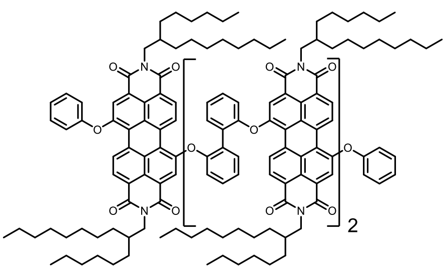

N,N´-Di(2-hexyldecyl)-1-phenoxy-7-(2,2´-biphenol)-perylene-3,4:9,10-bis(dicarboximide) (**PBI-Cap1**) (52.0 mg, 46.6 µmol), *N,N´*-di(2-hexyldecyl)-1,7-dibromoperylene-3,4:9,10-bis(dicarboximide) (**PBI-Center1**) (18.0 mg, 18.1 µmol) and Cs_2CO_3 (131 mg, 401 µmol) were dissolved in dry DMF (3 mL) and stirred at 110 °C for 35 min. under nitrogen atmosphere. After the reaction mixture was cooled to room temperature, 10 % HCl (aq.) (20 mL) was added and the separated aqueous layer was extracted with toluene (3 × 15 mL). The combined organic layers were washed with H_2O (10 mL) and dried over anhydrous $MgSO_4$. After removal of the solvents under reduced pressure, the crude product was purified by column chromatography on silica (CH_2Cl_2/cyclohexane, *v*/*v*, 8/2), GPC ($CHCl_3$) and precipitation from CH_2Cl_2 in MeOH to give **PBI-3** (40.1 mg, 13.1 µmol, 72 %) as a dark purple solid. ^{1}H NMR (600 MHz, TCE-d_2, 384 K): *δ*/ppm = 9.46 (d, ^3J = 8.3 Hz, 2H), 9.18 – 9.15 (m, 4H), 8.43 (d, ^3J = 8.3 Hz, 2H), 8.34 (s, 2H), 8.11 – 8.10 (m, 4H), 8.07 (s, 2H), 8.01 (s, 2H), 7.84 – 7.81 (m, 4H), 7.55 – 7.50 (m, 10H), 7.48 – 7.45 (m, 2H), 7.36 – 7.34 (m, 4H), 7.29 – 7.27 (m, 4H), 7.12 (d, ^3J = 7.4 Hz, 2H), 4.02 (d, ^3J = 7.2 Hz, 4H), 3.65 (d, ^3J = 7.0 Hz, 4H), 3.58 (d, ^3J = 7.0 Hz, 4H), 2.01 – 1.96 (m, 2H), 1.80 – 1.76 (m, 2H), 1.74 – 1.70 (m, 2H), 1.41 – 1.20 (m, 144H), 0.96 – 0.84 (m, 36H). ^{13}C NMR (151 MHz, TCE-d_2, 384 K): *δ*/ppm = 162.9, 162.7, 162.5, 162.1, 161.9, 155.0, 154.8, 154.5, 154.5, 152.3, 152.3, 133.1, 133.0, 133.0, 132.8, 132.8, 130.6, 130.4, 130.2, 130.1, 130.1, 129.7, 129.5, 129.2, 129.0, 129.0, 129.0, 128.9, 128.1, 127.9, 127.8, 125.6, 125.4, 125.0, 124.8, 124.5, 124.3, 123.8, 123.6, 123.5, 123.1, 123.1, 122.9, 122.8, 122.2, 122.1, 121.9, 121.9, 121.5, 120.2, 119.5, 119.3, 119.2, 44.7, 44.4, 44.4, 36.5, 36.5, 36.4, 31.8, 31.7, 31.7, 31.6, 31.5, 31.5, 31.5, 31.4, 29.8, 29.7, 29.4, 29.4, 29.3, 29.2, 29.2, 29.1, 28.9, 28.9, 28.9, 26.3, 26.3, 26.2, 26.2, 26.1, 22.3, 22.3, 22.2, 22.2, 13.6, 13.6, 13.6, 13.6. HRMS (MALDI-TOF, positive mode, DCTB in chloroform): m/z calcd. for $C_{204}H_{242}N_6O_{18}$ $[M]^+$: 3063.8200; found: 3063.8213.

Synthesis of **PBI-4**

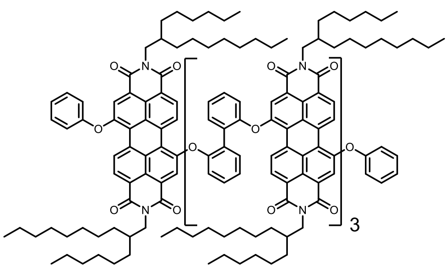

N,N´-Di(2-hexyldecyl)-1-phenoxy-7-(2,2´-biphenol)perylene-3,4:9,10-bis(dicarboximide) (**PBI-Cap1**) (16.5 mg, 14.8 µmol), **PBI-Center2** (10.0 mg, 4.95 µmol) and Cs_2CO_3 (48.2 mg, 148 µmol) were suspended in dry DMF (10 mL) and stirred at 90 °C for 6 h under nitrogen atmosphere. After cooling to room temperature, 10 % HCl (aq.) (20 mL) was added and the separated aqueous layer was extracted with toluene (3 × 10 mL). The combined organic layers were washed with H_2O (10 mL) and dried over anhydrous $MgSO_4$. The solvents were removed under reduced pressure, and the crude product was purified by column chromatography on silica gel (CH_2Cl_2), GPC ($CHCl_3$) and precipitation from CH_2Cl_2 in MeOH to give compound **PBI-4** (12.4 mg, 3.03 µmol, 61 %) as a dark purple solid. ^{1}H NMR (600 MHz, TCE-d_2, 384 K): δ/ppm = 9.46 (d, ^3J = 8.3 Hz, 2H), 9.18 – 9.13 (m, 6H), 8.43 (d, ^3J = 8.3 Hz, 2H), 8.33 (s, 2H), 8.10 – 8.07 (m, 8H), 7.99 (s, 2H), 7.97 (s, 2H), 7.83 – 7.79 (m, 6H), 7.55 – 7.46 (m, 14H), 7.44 – 7.41 (m, 2H), 7.36 – 7.26 (m, 10H), 7.09 (dd, ^3J = 8.0 Hz, ^4J = 0.8 Hz, 2H), 4.03 (d, ^3J = 7.2 Hz, 4H), 3.64 – 3.63 (m, 4H), 3.59 – 3.58 (m, 4H), 3.50 – 3.49 (m, 4H), 2.02 – 1.95 (m, 2H), 1.79 – 1.67 (m, 6H), 1.41 – 1.13 (m, 192H), 0.93 – 0.82 (m, 48H). ^{13}C NMR (151 MHz, TCE-d_2, 384 K): δ/ppm = 163.0, 162.7, 162.7, 162.5, 162.0, 161.9, 161.8, 155.0, 154.8, 154.5, 154.4, 152.3, 152.3, 152.0, 133.0, 133.0, 132.9, 132.9, 132.8, 132.7, 130.5, 130.4, 130.4, 130.2, 130.1, 130.1, 130.0, 129.7, 129.5, 129.3, 129.3, 129.0, 129.0, 128.8, 128.2, 127.8, 127.8, 127.8, 125.5, 125.5, 125.5, 125.4, 125.0, 124.8, 124.5, 124.3, 124.2, 123.8, 123.6, 123.5, 123.1, 123.1, 122.9, 122.8, 122.8, 122.7, 122.1, 121.9, 121.6, 121.5, 121.4, 119.7, 119.4, 119.3, 119.1, 44.7, 44.4, 44.3, 44.2, 36.5, 36.5, 36.4, 31.8, 31.7, 31.6, 31.6, 31.6, 31.6, 31.5, 31.5, 31.5, 31.4, 31.4, 29.8, 29.8, 29.7, 29.6, 29.4, 29.4, 29.3, 29.3, 29.2, 29.2, 29.2, 29.1, 28.9, 28.9, 28.9, 28.9, 26.3, 26.3, 26.2, 26.2, 26.2, 26.1, 26.0, 22.3, 22.3, 22.3, 22.2, 22.2, 22.2, 13.7, 13.6, 13.6, 13.6, 13.6, 13.6. HRMS (MALDI-TOF, positive mode, DCTB in chloroform): m/z calcd. for $C_{272}H_{322}N_8O_{24}$ $[M]^+$: 4084.4217; found: 4087.4266.

Synthesis of **PBI-5**

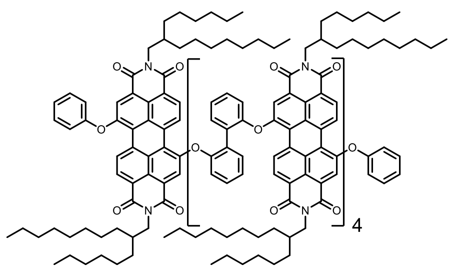

2,2′-Biphenol-phenoxy-substituted dimer (**PBI-Cap2**) (12.2 mg, 5.72 µmol), *N,N′*-di(2-hexyldecyl)-1,7-dibromoperylene-3,4:9,10-bis(dicarboximide) (**PBI-Center1**) (2.82 mg, 2.83 µmol), and Cs_2CO_3 (11.0 mg, 33.8 µmol) were suspended in dry DMF (2.5 mL) and stirred at 110 °C for 45 min under nitrogen atmosphere. After cooling to room temperature, 10 % HCl (aq.) (20 mL) was added and the separated aqueous layer was extracted with toluene (3 × 10 mL). The combined organic layers were washed with H_2O (10 mL) and dried over anhydrous $MgSO_4$. The solvents were removed under reduced pressure and the crude product was purified by column chromatography on silica gel (CH_2Cl_2), GPC ($CHCl_3$) and precipitation from CH_2Cl_2 in MeOH to give compound **PBI-5** (7.31 mg, 1.43 µmol, 51 %) as a dark purple solid. ^{1}H NMR (600 MHz, TCE-d_2, 384 K): δ/ppm = 9.46 (d, ^3J = 8.2 Hz, 2H), 9.18 – 9.12 (m, 8H), 8.43 (d, ^3J = 8.2 Hz, 2H), 8.33 (s, 2H), 8.10 – 8.07 (m, 10H), 8.00 (s, 2H), 7.97 (s, 2H), 7.96 (s, 2H), 7.83 – 7.77 (m, 8H), 7.54 – 7.45 (m, 18H), 7.44 – 7.42 (m, 2H), 7.36 – 7.32 (m, 2H), 7.31 – 7.26 (m, 10H), 7.09 (d, ^3J = 7.3 Hz, 2H), 4.02 (d, ^3J = 7.0 Hz, 4H), 3.64 – 3.63 (m, 4H), 3.59 – 3.58 (m, 4H), 3.51 – 3.49 (m, 8H), 2.00 – 1.96 (m, 2H), 1.76 – 1.65 (m, 8H), 1.40 – 1.12 (m, 240H), 0.97 – 0.82 (m, 60H). ^{13}C NMR (151 MHz, TCE-d_2, 384 K): δ/ppm = 163.0, 162.7, 162.7, 162.5, 162.5, 162.1, 161.9, 161.9, 161.8, 155.0, 154.8, 154.5, 154.4, 154.4, 154.4, 152.4, 152.3, 152.3, 152.0, 133.0, 133.0, 133.0, 132.9, 132.8, 132.8, 132.7, 132.6, 130.5, 130.4, 130.3, 130.2, 130.1, 130.1, 130.0, 129.7, 129.5, 129.3, 129.3, 129.0, 128.9, 128.2, 127.9, 127.8, 125.5, 125.4, 125.3, 125.0, 124.8, 124.5, 124.3, 124.3, 123.8, 123.6, 123.6, 123.5, 123.1, 123.0, 122.8, 122.2, 121.9, 121.7, 121.7, 121.7, 121.7, 121.6, 121.5, 121.5, 120.2, 119.6, 119.6, 119.5, 119.5, 119.4, 119.4, 119.3, 119.1, 44.7, 44.4, 44.3, 44.2, 36.5, 36.5, 36.5, 36.4, 31.8, 31.7, 31.6, 31.6, 31.5, 31.5, 31.5, 31.5, 31.4, 31.4, 31.4, 29.8, 29.8, 29.7, 29.7, 29.6, 29.4, 29.4, 29.3, 29.3, 29.3, 29.2, 29.2, 29.2, 29.1, 28.9, 28.9, 28.9, 28.9, 28.8, 26.4, 26.3, 26.2, 26.2, 26.1, 26.1, 26.1, 26.0, 22.3, 22.3, 22.2, 22.2, 22.2, 22.2, 22.2, 13.6, 13.6, 13.6, 13.6, 13.6, 13.6, 13.6, 13.5. HRMS (MALDI-TOF, positive mode, DCTB in chloroform): m/z calcd. for $C_{340}H_{402}N_{10}O_{30}$ $[M]^+$: 5105.0233; found: 5105.0241.

Synthesis of **PBI-6**

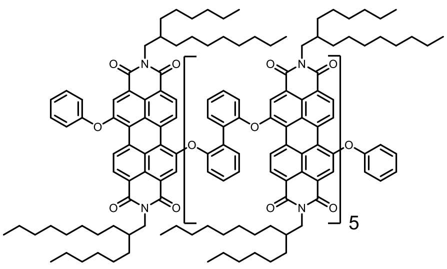

N,N´-Di(2-hexyldecyl)-1-phenoxy-7-(2,2´-biphenol)perylene-3,4:9,10-bis(dicarboximide) (**PBI-Cap1**) (8.30 mg, 7.44 µmol), **PBI-Center4** (10.0 mg, 2.46 µmol) and Cs_2CO_3 (26.4 mg, 81.0 µmol) were suspended in dry DMF (10 mL) and stirred at 90 °C for 7 h under nitrogen atmosphere. After cooling to room temperature, 10 % HCl (aq.) (20 mL) was added and the separated aqueous layer was extracted with toluene (3 × 10 mL). The combined organic layers were washed with H_2O (10 mL) and dried over anhydrous $MgSO_4$. The solvents were removed under reduced pressure and the crude product was purified by column chromatography on silica gel (CH_2Cl_2), GPC ($CHCl_3$) and precipitation from CH_2Cl_2 in MeOH to give compound **PBI-6** (5.20 mg, 848 nmol, 34 %) as a dark purple solid. ^{1}H NMR (600 MHz, TCE-d_2, 384 K): δ/ppm = 9.47 (d, ^3J = 8.3 Hz, 2H), 9.19 – 9.13 (m, 10H), 8.44 (d, ^3J = 8.2 Hz, 2H), 8.34 (s, 2H), 8.12 – 8.06 (m, 12H), 8.00 (s, 2H), 7.99 – 7.96 (m, 6H), 7.82 – 7.76 (m, 10H), 7.55 – 7.50 (m, 8H), 7.49 – 7.41 (m, 16H), 7.36 – 7.32 (m, 2H), 7.31 – 7.22 (m, 12H), 7.11 – 7.08 (m, 2H), 4.05 – 4.00 (m, 4H), 3.68 – 3.63 (m, 4H), 3.63 – 3.57 (m, 4H), 3.57 – 3.49 (m, 12H), 2.02 – 1.96 (m, 2H), 1.82 – 1.75 (m, 2H), 1.73 – 1.67 (m, 8H), 1.32 – 1.12 (m, 288H), 0.93 – 0.82 (m, 72H). ^{13}C NMR (151 MHz, TCE-d_2, 384 K): δ/ppm = 163.0, 162.8, 162.7, 162.5, 162.1, 162.0, 161.9, 161.9, 161.9, 155.0, 154.8, 154.5, 154.5, 154.4, 154.4, 154.4, 152.4, 152.3, 152.1, 152.1, 133.1, 133.0, 133.0, 132.9, 132.8, 132.8, 132.7, 132.7, 132.6, 130.5, 130.4, 130.3, 130.3, 130.2, 130.1, 130.0, 129.7, 129.5, 129.3, 129.3, 129.0, 128.9, 128.2, 127.9, 127.9, 127.9, 125.5, 125.4, 125.3, 125.3, 125.3, 125.0, 124.9, 124.5, 124.3, 124.3, 123.8, 123.7, 123.6, 123.2, 123.0, 122.9, 122.9, 122.9, 122.2, 121.9, 121.8, 121.8, 121.8, 121.6, 121.6, 121.5, 121.5, 120.2, 119.6, 119.5, 119.4, 119.4, 119.3, 119.1, 44.8, 44.4, 44.4, 44.3, 36.6, 36.5, 36.5, 36.4, 31.8, 31.7, 31.7, 31.6, 31.6, 31.5, 31.5, 31.5, 31.5, 31.5, 31.4, 31.4, 29.8, 29.8, 29.7, 29.6, 29.4, 29.4, 29.3, 29.3, 29.2, 29.2, 29.2, 29.1, 29.1, 28.9, 28.9, 28.9, 28.9, 28.9, 28.9, 26.4, 26.3, 26.3, 26.2, 26.2, 26.1, 26.1, 26.1, 22.3, 22.3, 22.3, 22.2, 22.2, 22.2, 22.2, 13.6, 13.6, 13.6, 13.6, 13.6. HRMS (MALDI-TOF, positive mode, DCTB in chloroform): m/z calcd. for $C_{408}H_{482}N_{12}O_{36}$ $[M]^+$, 6125.6249, found: 6125.6189.

Synthesis of **PBI-10**

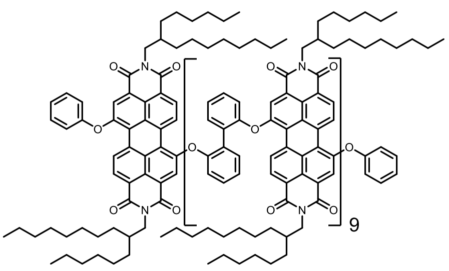

2,2´-Biphenol-phenoxy-substituted trimer (**PBI-Cap3**) (23.0 mg, 7.28 µmol), **PBI-Center4** (10.0 mg, 2.46 µmol), and Cs_2CO_3 (8.02 mg, 24.6 µmol) were suspended in dry DMF (10 mL) and stirred at 90 °C for 7 h under nitrogen atmosphere. After cooling to room temperature, 10 % HCl (aq.) (40 mL) was added and the separated aqueous layer was extracted with toluene (3 × 15 mL). The combined organic layers were washed with H_2O (40 mL) and dried over anhydrous $MgSO_4$. The solvents were removed under reduced pressure and the crude product was purified by column chromatography on silica gel (CH_2Cl_2), GPC ($CHCl_3$) and precipitation from CH_2Cl_2 in MeOH to give compound **PBI-10** (4.00 mg, 392 nmol, 16 %) as a dark purple solid. ^{1}H NMR (600 MHz, TCE-d_2, 384 K): δ/ppm = 9.47 (d, ^3J = 8.2 Hz, 2H), 9.19 – 9.12 (m, 18H), 8.43 (d, ^3J = 8.2 Hz, 2H), 8.33 (s, 2H), 8.12 – 8.07 (m, 16H), 8.07 – 8.06 (m, 3H), 8.00 (s, 2H), 7.98 – 7.95 (m, 13H), 7.83 – 7.74 (m, 19H), 7.55 – 7.50 (m, 9H), 7.49 – 7.41 (m, 32H), 7.35 – 7.33 (m, 2H), 7.31 – 7.21 (m, 20H), 7.11 – 7.07 (m, 2H), 4.05 – 4.00 (m, 4H), 3.67 – 3.62 (m, 4H), 3.61 – 3.58 (m, 4H), 3.58 – 3.45 (m, 28H), 2.02 – 1.95 (m, 2H), 1.80 – 1.76 (m, 2H), 1.73 – 1.66 (m, 16H), 1.32 – 1.12 (m, 480H), 0.92 – 0.82 (m, 120H). ^{13}C NMR (151 MHz, TCE-d_2, 384 K): δ/ppm = 163.0, 162.8, 162.7, 162.6, 162.1, 162.0, 161.9, 161.9, 155.0, 155.0, 154.8, 154.5, 154.5, 154.4, 154.4, 154.4, 152.4, 152.3, 152.2, 152.1, 152.1, 133.1, 133.0, 133.0, 132.8, 132.8, 132.7, 132.7, 130.5, 130.4, 130.3, 130.3, 130.2, 130.1, 130.0, 129.3, 129.3, 129.0, 128.9, 128.9, 127.9, 127.9, 125.4, 125.4, 125.3, 125.3, 125.0, 124.5, 124.4, 124.3, 123.8, 123.7, 123.6, 123.6, 123.5, 123.2, 123.0, 123.0, 122.9, 122.9, 121.9, 121.9, 121.8, 121.8, 121.8, 121.6, 121.6, 121.5, 120.2, 119.4, 119.4, 119.3, 44.7, 44.4, 44.3, 36.5, 36.5, 36.5, 36.4, 31.8, 31.7, 31.7, 31.6, 31.6, 31.5, 31.5, 31.5, 31.4, 29.8, 29.8, 29.7, 29.6, 29.4, 29.4, 29.3, 29.3, 29.2, 29.2, 29.1, 29.1, 28.9, 28.9, 28.9, 28.9, 28.9, 26.4, 26.3, 26.3, 26.2, 26.1, 26.1, 22.3, 22.3, 22.2, 22.2, 22.2, 22.2, 13.6, 13.6, 13.6, 13.6, 13.6, 13.6. HRMS (MALDI-TOF, positive mode, DCTB in chloroform, most abundant): m/z calcd. for $C_{680}H_{802}N_{20}O_{60}$ $[M]^+$: 10215.0546; found: 10215.0621.

Synthesis of **PBI-14**

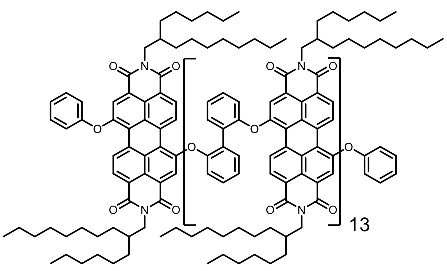

2,2´-Biphenol-phenoxy-substituted pentamer (**PBI-Cap5**) (10.0 mg, 1.92 µmol), **PBI-Center4** (4.00 mg, 0.98 µmol), and Cs_2CO_3 (24.4 mg, 76.0 µmol) were suspended in dry DMF (4 mL) and stirred at 100 °C for 2 h under nitrogen atmosphere. After cooling to room temperature, 10 % HCl (aq.) (20 mL) was added and the separated aqueous layer was extracted with toluene (3 × 10 mL). The combined organic layers were washed with H_2O (20 mL) and dried over anhydrous $MgSO_4$. Afterwards, the crude product was purified by column chromatography on silica gel (CH_2Cl_2/cyclohexane, *v/v*, 3/1), GPC ($CHCl_3$) and precipitation from CH_2Cl_2 in MeOH to give compound **PBI-14** (0.84 mg, 58.7 nmol, 6 %) as a dark purple solid. ^{1}H NMR (600 MHz, TCE-d_2, 384 K): δ/ppm = 9.46 (d, ^3J = 8.2 Hz, 2H), 9.17 – 9.13 (m, 26H), 8.43 (d, ^3J = 8.3 Hz, 2H), 8.33 (s, 2H), 8.11 – 8.07 (m, 28H), 8.00 (s, 2H), 7.99 – 7.96 (m, 22H), 7.80 – 7.75 (m, 24H), 7.51 – 7.43 (m, 56H), 7.35 – 7.33 (m, 2H), 7.29 – 7.21 (m, 30H), 7.10 – 7.08 (m, 2H), 4.04 – 4.00 (m, 4H), 3.66 – 3.63 (m, 4H), 3.59 – 3.50 (m, 48H), 2.01 – 2.00 (m, 2H), 1.78 – 1.75 (m, 2H), 1.72 – 1.67 (m, 24H), 1.27 – 1.13 (m, 672H), 0.90 – 0.83 (m, 168H). ^{13}C NMR (151 MHz, TCE-d_2, 384 K): δ/ppm = 162.8, 162.7, 162.6, 162.6, 162.5, 162.0, 162.0, 161.9, 161.6, 161.6, 154.5, 154.5, 154.4, 154.4, 154.4, 154.4, 152.2, 152.2, 152.1, 133.3, 133.1, 133.1, 133.0, 133.0, 132.8, 132.8, 132.8, 132.7, 132.7, 132.6, 130.4, 130.4, 130.3, 130.3, 130.3, 130.2, 130.1, 130.0, 130.0, 129.3, 129.3, 129.3, 129.0, 129.0, 129.0, 128.9, 128.0, 128.0, 128.0, 127.9, 127.9, 127.9, 125.3, 125.3, 124.9, 124.4, 124.4, 123.2, 123.0, 123.0, 123.0, 122.9, 121.9, 121.9, 121.9, 121.6, 120.2, 119.4, 119.3, 44.3, 44.2, 36.5, 36.5, 36.4, 31.9, 31.8, 31.7, 31.7, 31.6, 31.6, 31.5, 31.5, 31.5, 31.5, 31.4, 29.8, 29.8, 29.7, 29.6, 29.4, 29.4, 29.3, 29.2, 29.2, 29.1, 29.1, 28.9, 28.9, 28.9, 28.9, 28.4, 26.4, 26.3, 26.3, 26.2, 26.1, 26.1, 26.1, 22.3, 22.3, 22.2, 22.2, 13.6, 13.6, 13.6, 13.5. HRMS (MALDI-TOF, positive mode, DCTB in chloroform, most abundant): m/z calcd. for $C_{952}H_{1122}N_{28}O_{84}$ $[M]^+$: 14300.4710; found: 14300.9520.

3. 2D NMR Spectroscopy

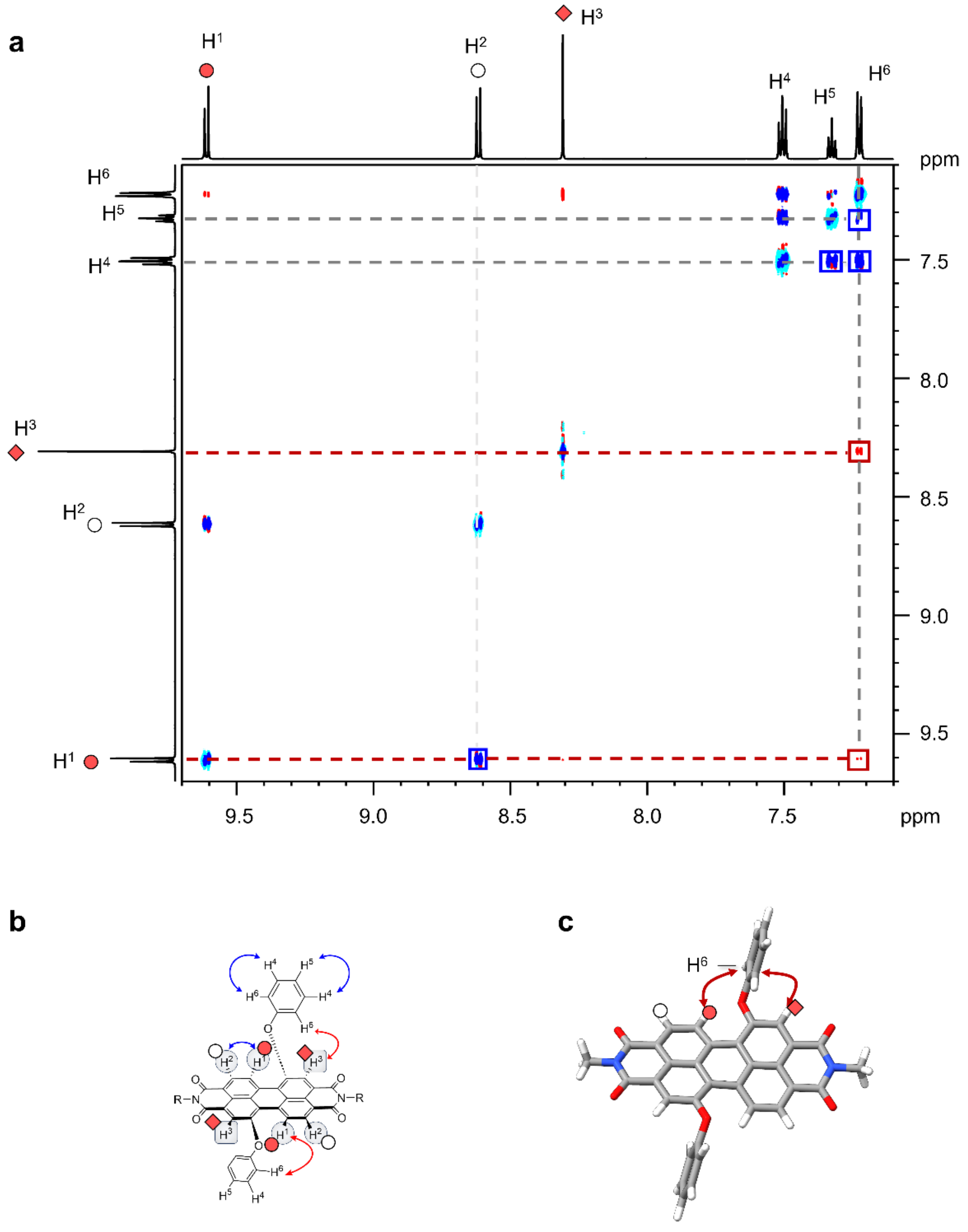

Supplementary Figure 5. 2D NMR spectroscopic characterization. a Superposition of COSY (blue) and ROESY (red: positive signal, cyan: negative signal) spectra (600 MHz, 295 K, TCE-d_2). **b** Chemical structure of **PBI-1** with the significant protons highlighted in color. **c** Side-view of the energy-minimized structure of **PBI-1** obtained by DFT calculations (ωB97X-D/def2-SVP). Red boxes / arrows indicate important ROESY cross-signals, blue boxes / arrows indicate important COSY signals.

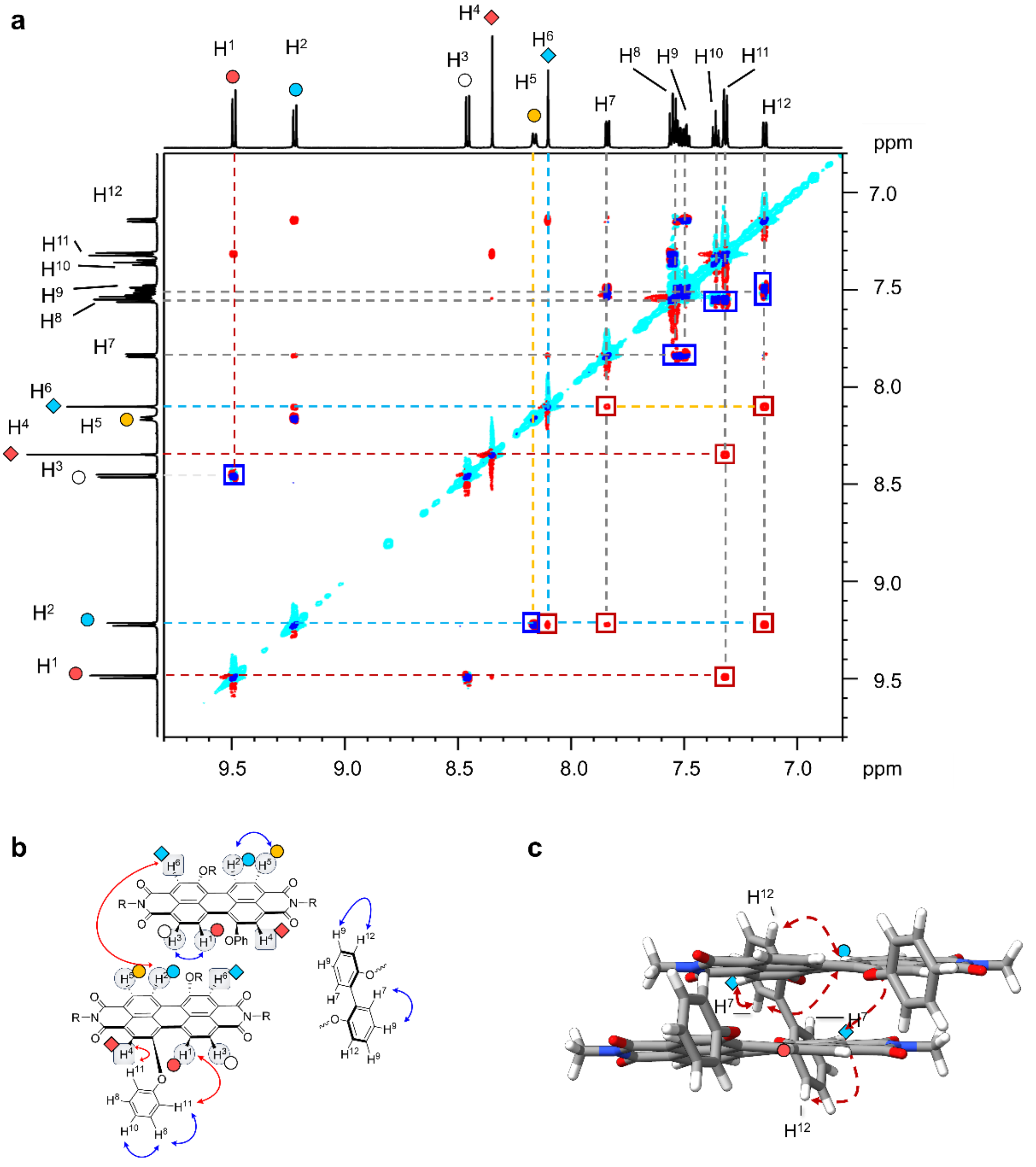

Supplementary Figure 6. 2D NMR spectroscopic characterization. a Superposition of COSY (blue) and ROESY (red: positive signal, cyan: negative signal) spectra (600 MHz, 384 K, TCE-d_2). **b** Chemical structure of **PBI-2** with the significant protons highlighted in color, spacer moieties are omitted for clarity. **c** Side-view of the energy-minimized structure of **PBI-2** obtained by density functional theory (DFT) calculations (wB97XD/def2-SVP). Red boxes / arrows indicate important ROESY cross-signals, blue boxes / arrows indicate important COSY signals. 2-Hexyldecyl groups are replaced by methyl groups.

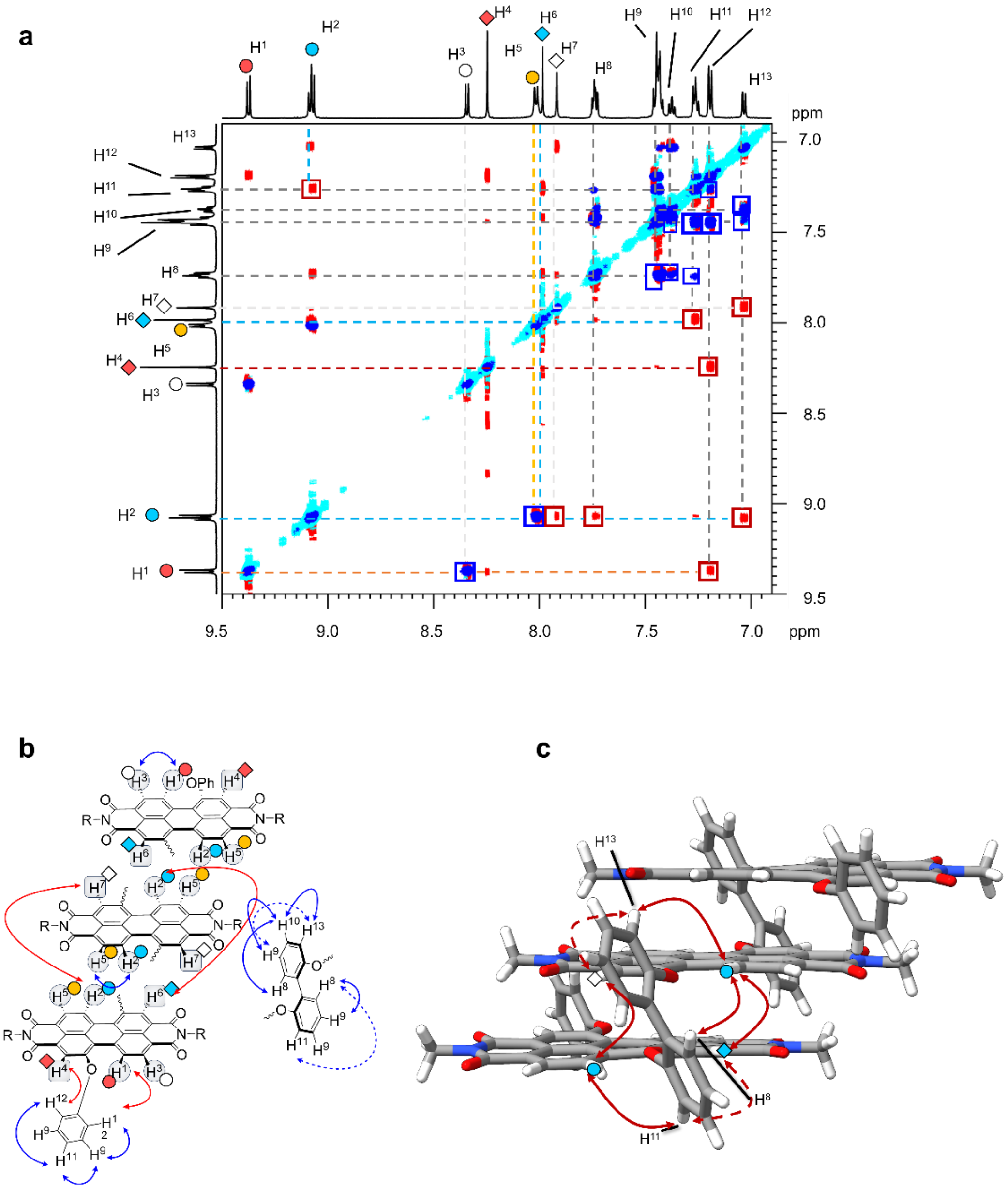

Supplementary Figure 7. 2D NMR spectroscopic characterization. a Superposition of COSY (blue) and ROESY (red: positive signal/ cyan: negative signal) spectra (600 MHz, 384 K, TCE-d_2), **b** Chemical structure of **PBI-3** with the significant protons highlighted in color, spacer moieties are omitted for clarity. **c** Side-view of the energy-minimized structure of **PBI-3** obtained by DFT calculations (ωB97X-D/def2-SVP). Red boxes / arrows indicate important ROESY cross-signals, blue boxes / arrows indicate important COSY signals. 2-Hexyldecyl groups are replaced by methyl groups.

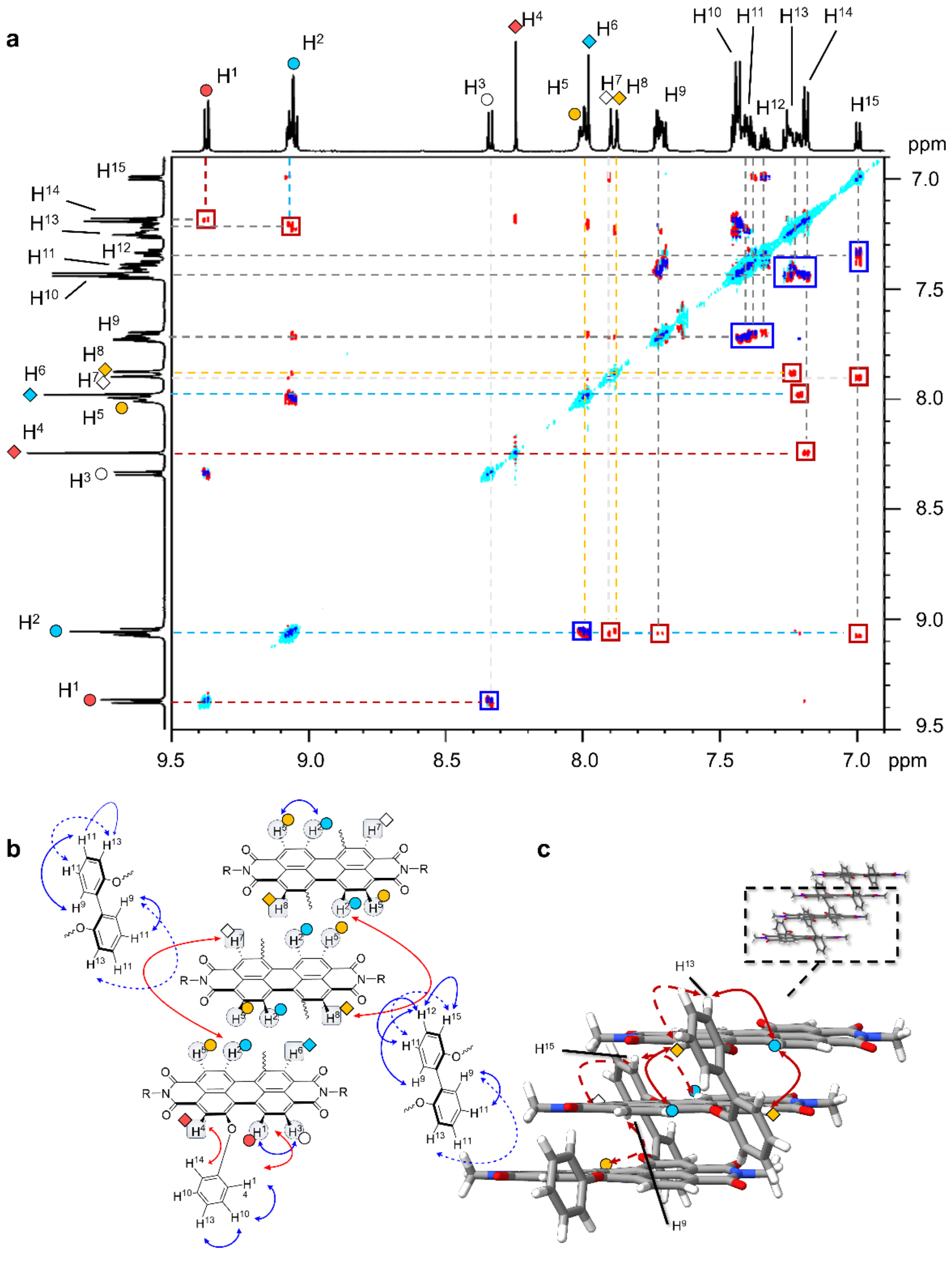

Supplementary Figure 8. 2D NMR spectroscopic characterization. a Superposition of COSY (blue) and ROESY (red: positive signal/ cyan: negative signal) spectra (600 MHz, 384 K, TCE-d_2). **b** Excerpt of the chemical structure of **PBI-4** with the significant protons highlighted in color, spacer moieties and one PBI chromophore are omitted for clarity. **c** Side-view of the energy-minimized structure of **PBI-4** obtained by DFT calculations (ωB97X-D/def2-SVP). Red boxes / arrows indicate important ROESY cross-signals, blue boxes / arrows indicate important COSY signals. 2-Hexyldecyl groups are replaced by methyl groups.

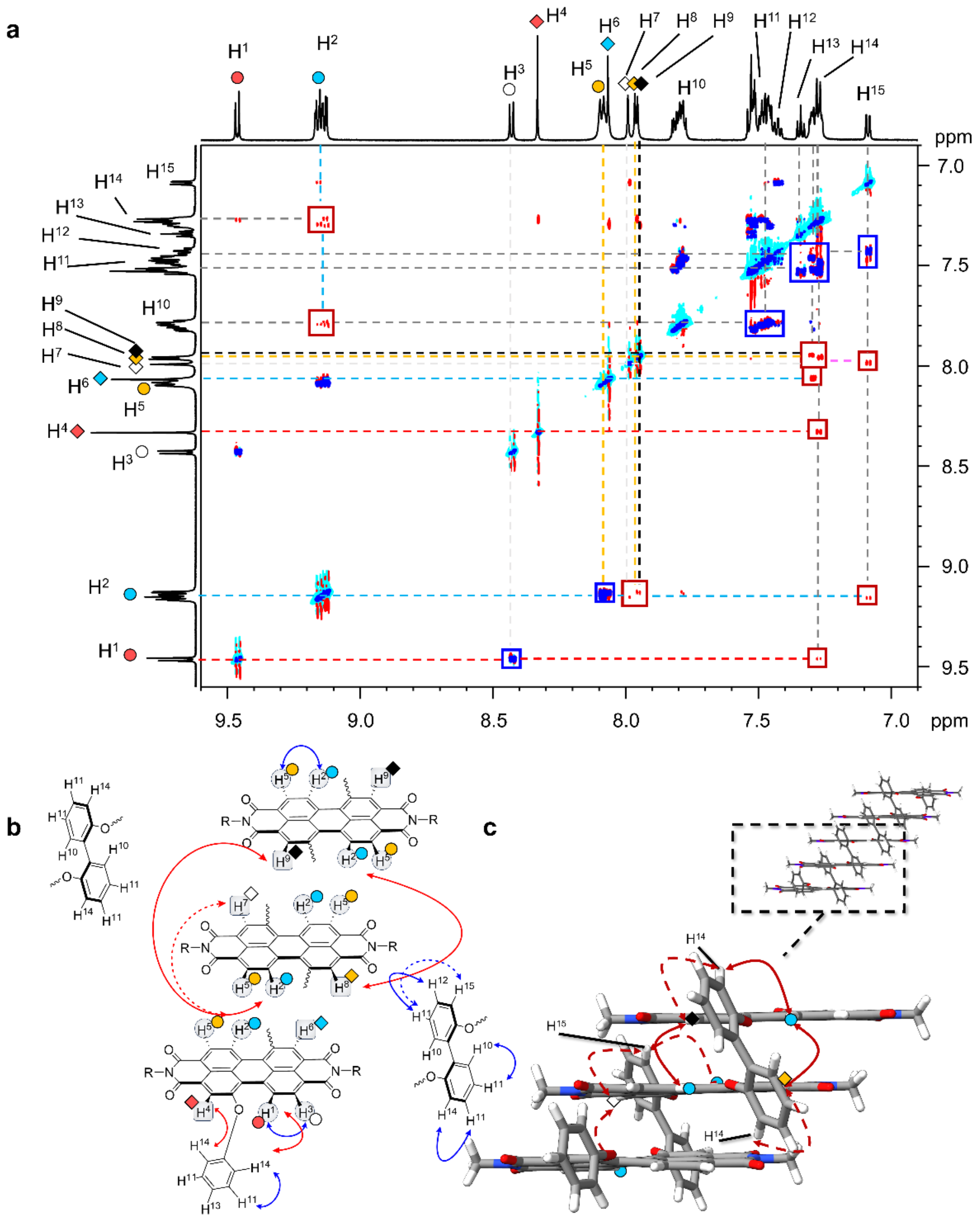

Supplementary Figure 9. 2D NMR spectroscopic characterization. a Superposition of COSY (blue) and ROESY (red: positive signal, cyan: negative signal) spectra (600 MHz, 384 K, TCE-*d*2). **b** Excerpt of the chemical structure of **PBI-5** with the significant protons highlighted in color, spacer moieties and two PBI chromophores are omitted for clarity. **c** Side-view of the energy-minimized structure of **PBI-5** obtained by DFT calculations (ωB97X-D/def2-SVP). Red boxes / arrows indicate important ROESY cross-signals, blue boxes / arrows indicate important COSY signals. 2-Hexyldecyl groups are replaced by methyl groups.

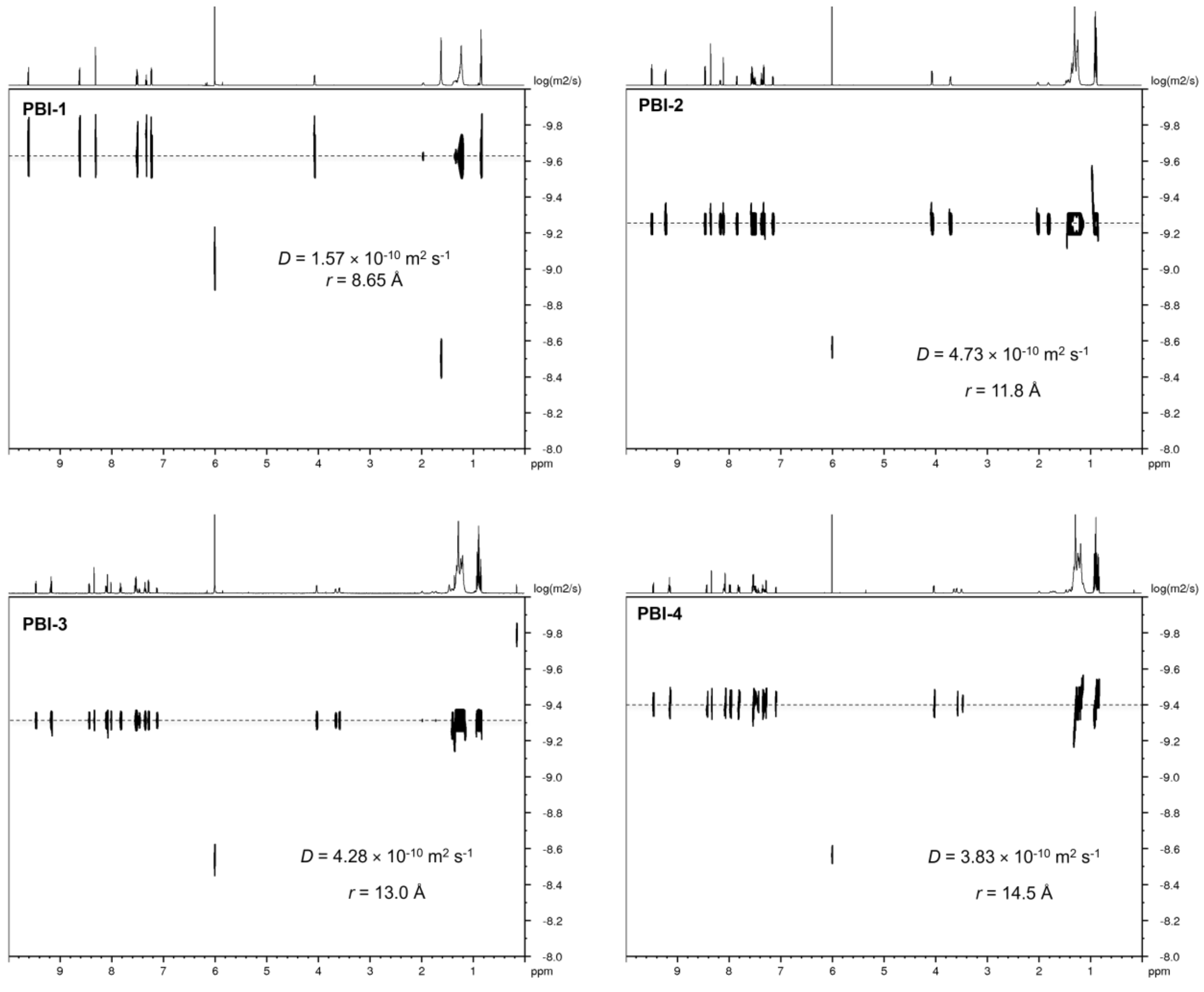

Supplementary Figure 10. NMR spectroscopic characterization. 2D plot of DOSY NMR (600 MHz, 295 K (**PBI-1**), 384 K (**PBI-2, PBI-3**, **PBI-4**), TCE-d_2) spectra of **PBI-1** (top, left), **PBI-2** (top, right), **PBI-3** (bottom, left) and **PBI-4** (bottom, right) with the hydrodynamic radii as received from the Stokes-Einstein equation.[15]

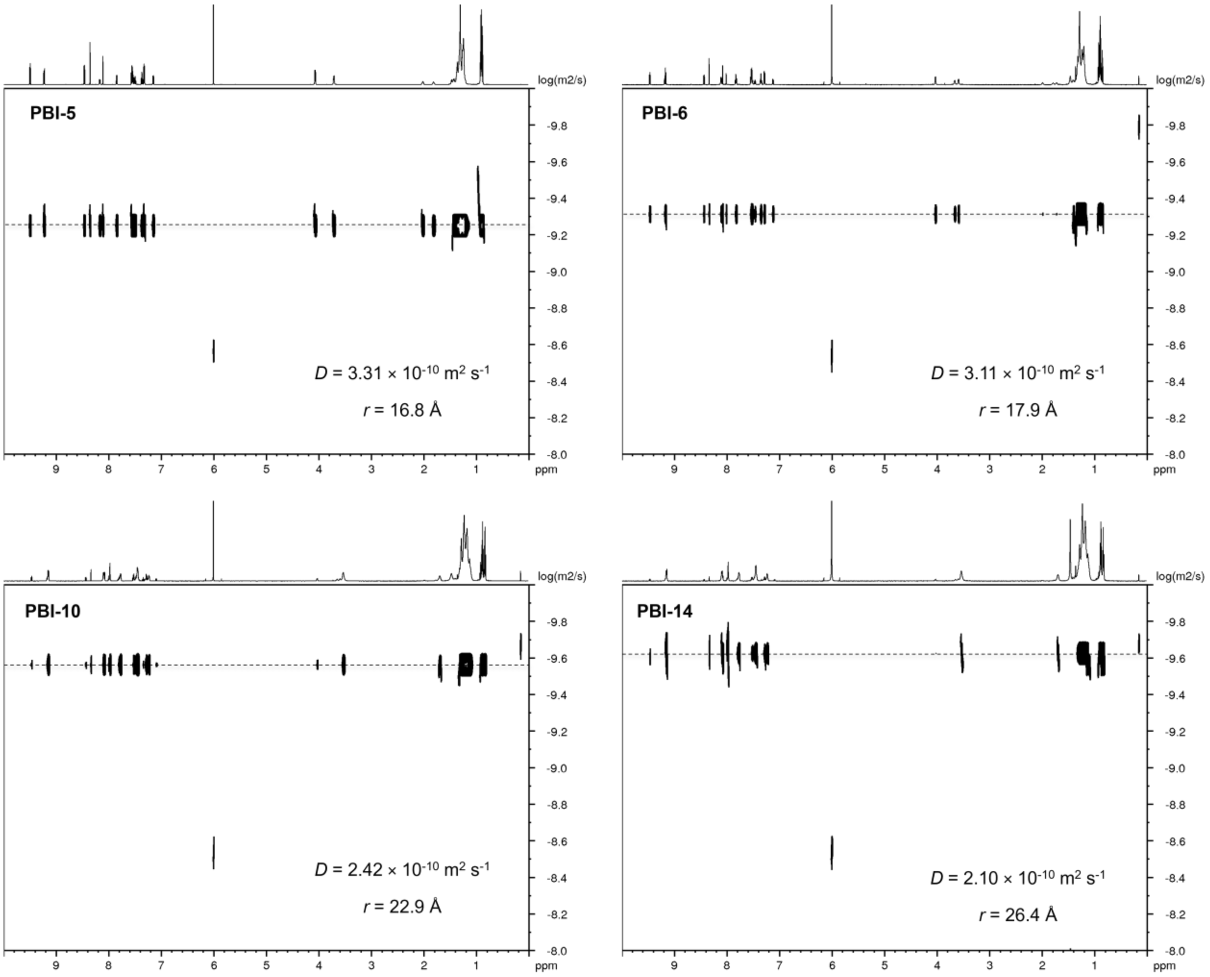

Supplementary Figure 11. NMR spectroscopic characterization. 2D plot of DOSY NMR (600 MHz, 384 K, TCE-d_2) spectra of **PBI-5** (top, left), **PBI-6** (top, right), **PBI-10** (bottom, left) and **PBI-14** (bottom, right) with the hydrodynamic radii as received from the Stokes-Einstein equation.[15]

4. Quantum Chemical Calculations

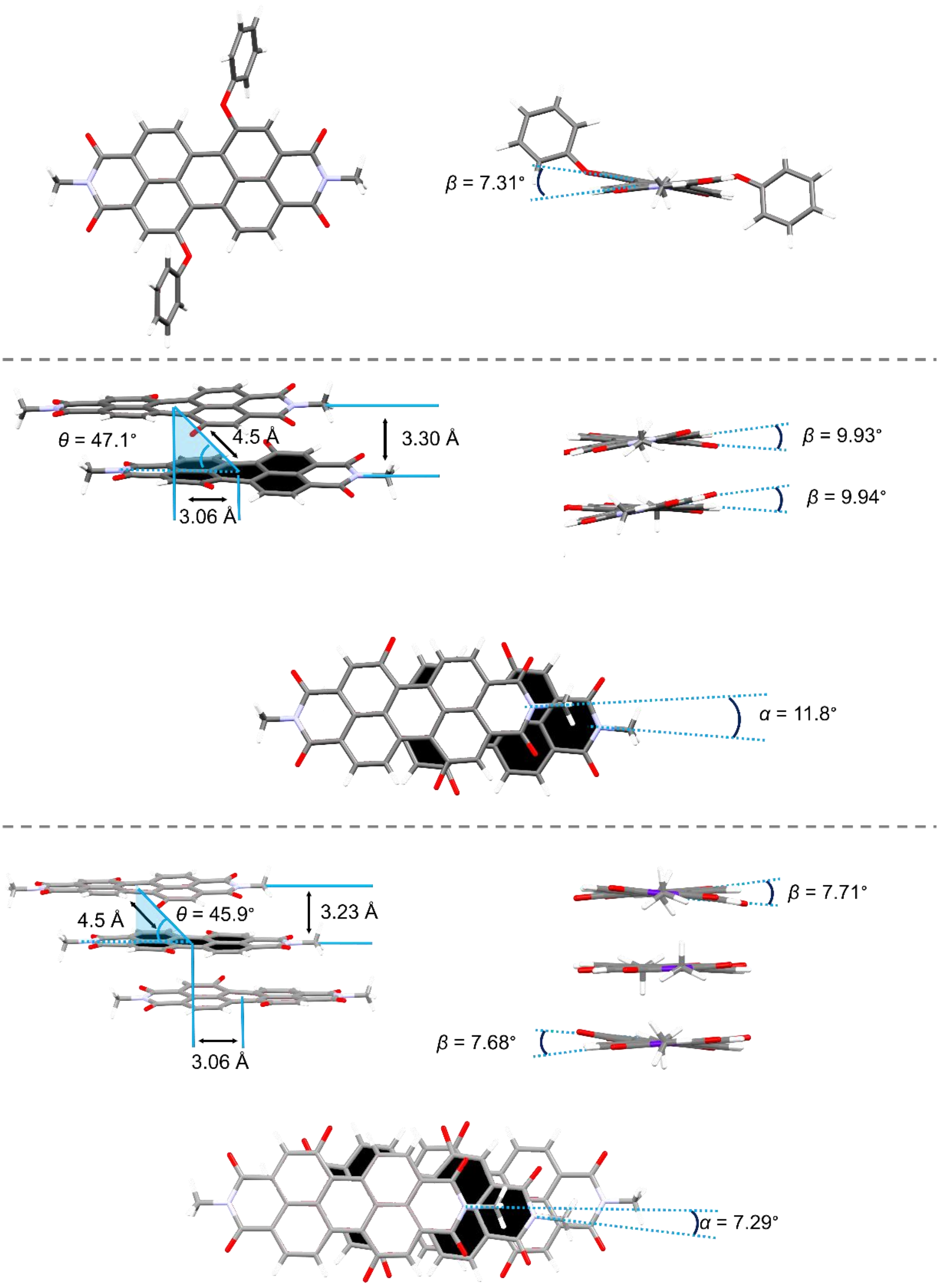

Supplementary Figure 12. Top side and front view on the geometry-optimized structure of **PBI-1** (top), **PBI-2** (middle) and **PBI-3** (bottom) (ωB97X-D/def2-SVP). The spacer moieties were removed for improved clarity.

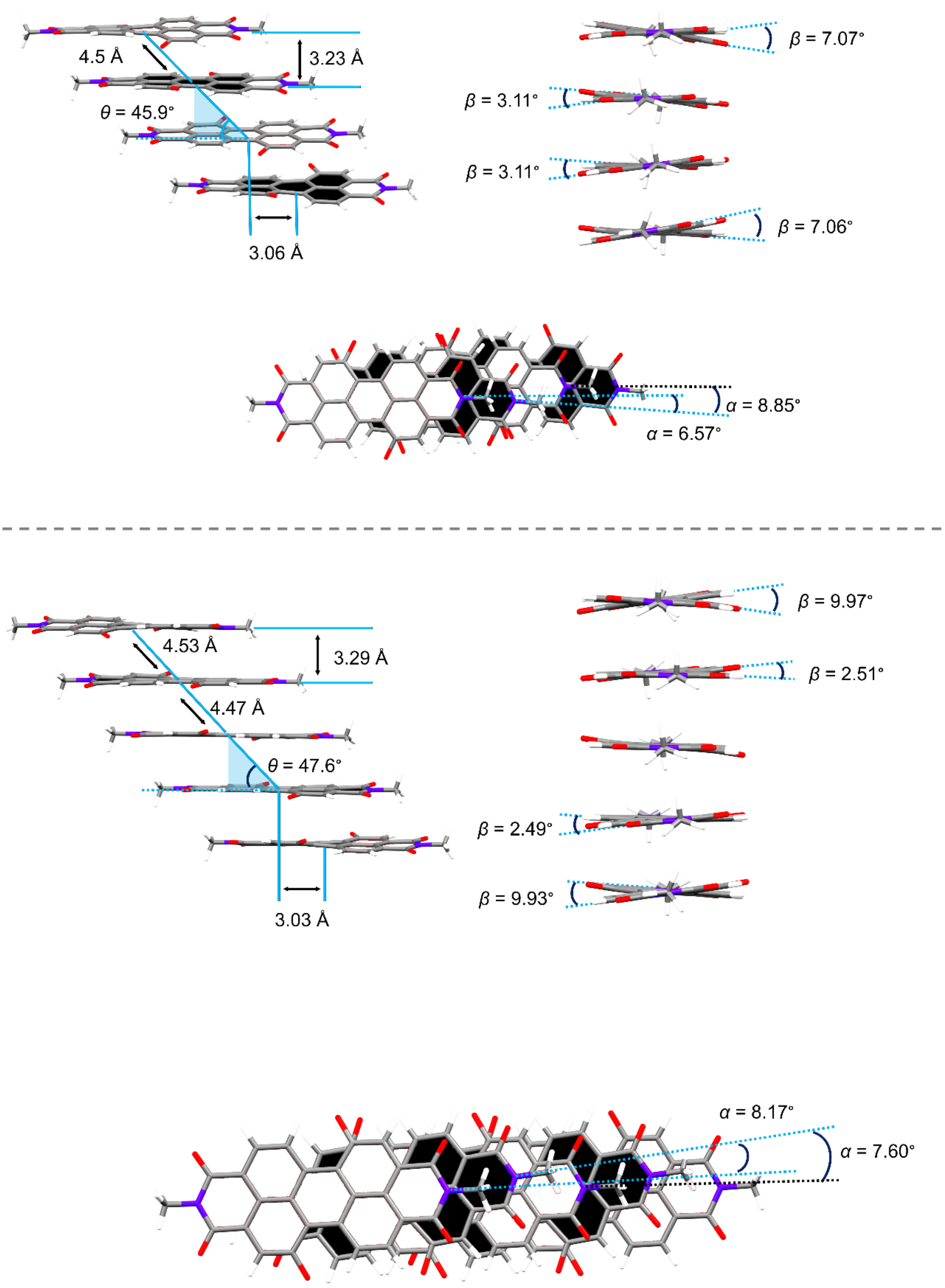

Supplementary Figure 13. Top-, side- and front-view of the geometry-optimized structure of **PBI-4** (top), and **PBI-5** (bottom) (ωB97X-D/def2-SVP). The spacer moieties were removed for improved clarity.

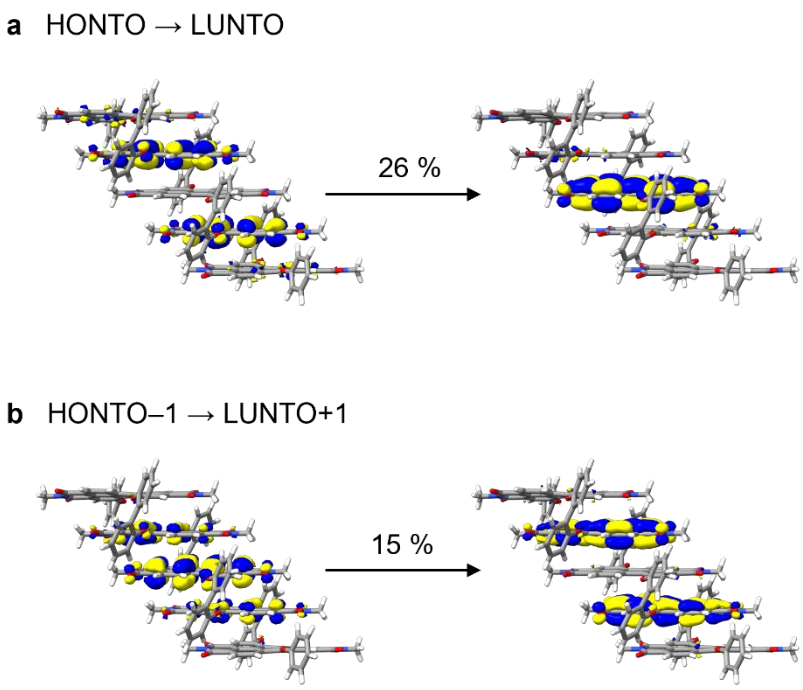

Supplementary Figure 14. a HONTO and LUNTO and **b** HONTO–1 and LUNTO+1 orbital plots for the important transitions to the S6 state of **PBI-5** with their contributions (%) in Tol (isovalue 0.02) derived from TD-DFT calculations (ωB97X-D/def2-SVP).

NTOs were generated using the MultiWFN 3.8 software package.[16] These NTO plots, specifically the highest occupied (HONTO) and lowest unoccupied (LUNTO) orbitals (Fig. 3d, Supplementary Fig. 14–16), were obtained from TD-DFT calculations to qualitatively interpret the excited state character. Due to the involvement of numerous molecular orbital (MO) transitions, excited state interpretation based solely on MO pairs is insufficient.

Coupling Calculations

The quantum chemical calculations for determination of the different types of couplings were conducted following a procedure of the previously reported **Bis-PBI2**,[17] **Tris-PBI**[18] and **DA-PBI2–4**[3] molecules using the geometry optimized structures to perform time-dependent DFT (TD-DFT) calculations (Gaussian 16 program package, ωB97X-D functional and def2-SVP basis set in Tol (PCM)).

Initially the transition dipole moment μ_{eg} of the **PBI-1** reference was calculated from the absorption spectrum measured in Tol at 295 K. It was determined as the integral of the reduced absorption band by[19]

$$|\mu_{\mathrm{eg}}|^2 = \frac{3hc\varepsilon_0 ln10}{2\pi^2 N_{\mathrm{A}}} \cdot \int_{\tilde{\nu}_1}^{\tilde{\nu}_2} \frac{\varepsilon(\tilde{\nu})}{\tilde{\nu}} d\tilde{\nu}. \quad (2)$$

The molar extinction coefficient is given by $\varepsilon(\tilde{\nu})$, the speed of light by c and the Planck constant by h. Furthermore, ε_0 represents the vacuum permittivity and N_A gives the Avogadro constant. A value of μ_{eg} = 7.52 D was obtained and further applied to appropriately scale the transition charges determined by the Mulliken electron excitation analysis.

The long-range Coulomb coupling J_{Coul} was calculated applying TD-DFT. The resulting transition density was projected onto atomic transition charges by a Mulliken style electron excitation analysis for the first excited state using the Multiwfn software package.[20] The Coulomb coupling J_{Coul} was calculated using the transition charge method.[21]

The exciton coupling energy was calculated according to eq. 3:

$$J = \frac{1}{4\pi\varepsilon_0} \sum_i \sum_j \frac{q_i^{(\mathrm{a})} \cdot q_j^{(\mathrm{b})}}{\left|r_i^{(\mathrm{a})} - r_j^{(\mathrm{b})}\right|}. \quad (3)$$

The transition charge on atom i of chromophore a is defined by $q_i^{(a)}$. The position vector of the respective transition charge is given by $r_i^{(a)}$ and ε_0 gives the vacuum permittivity. As reported in literature, the transition dipole moments of the respective monomer layers were overestimated by the TD-DFT calculations ($\mu_{eg\ (TD\text{-}DFT)}$ = 9.85 D (**PBI-2**) and 9.96 D (**PBI-5**)).[17, 22]

In comparison to the transition dipole values obtained for the integral of the reduced absorption band $\mu_{eg\,(TD\text{-}DFT)}$ is overestimated by a factor of 1.31 and 1.32 for **PBI-2** and **PBI-5**, respectively. Therefore, the transition charges were scaled by a factor of 0.76 (**PBI-2**) and 0.75 (**PBI-5**).

The transfer integrals were determined within the unique fragment approach using the Amsterdam Density Functional (ADF) program[23, 24] applying the PW91 functional[25] and a TZP basis set.[26]

Given the overlap integrals S_e / S_h of the LUMO / HOMO, the electron / hole site energies $E_{e,1}$ / $E_{h,1}$ and $E_{e,2}$ / $E_{h,2}$ of the respective fragments 1 and 2 the effective electron (t_e) and hole (t_h) transfer integrals can be calculated by[17, 27]

$$t_{\mathrm{e}} = \frac{t_{\mathrm{e}}' - \frac{1}{2}\left(E_{\mathrm{e},1} + E_{\mathrm{e},2}\right)S_{\mathrm{e}}}{1 - S_{\mathrm{e}}^2} \tag{4}$$

$$t_{\mathrm{h}} = \frac{t_{\mathrm{h}}' - \frac{1}{2}\left(E_{\mathrm{h},1} + E_{\mathrm{h},2}\right)S_{\mathrm{h}}}{1 - S_{\mathrm{h}}^2}. \tag{5}$$

The short-range charge-transfer coupling J_{CT} was calculated at the perturbative limit[28, 29] (charge-transfer state and local Frenkel exciton state should be well separated) with its effective electron (t_e) and hole (t_h) transfer integrals and the energy difference E_{CT}–E_{S1} = 3200 cm^{-1} for **PBI-2** and 4654 cm^{-1} for **PBI-5** (Supplementary Table 1). Energy values for the first excited virtual state (S_1) and the first virtual CT state have been derived from TD-DFT calculations (ωB97X-D/def2-SVP) in Tol (PCM).

Information of the CT character of the calculated excited state has been derived from qualitative interpretation of the NTO plots for **PBI-5** (Supplementary Figure 14). Due to difficulties to identify the first virtual CT state for **PBI-2**, literature values of E_{CT}–E_{S1} = 3200 cm^{-1} had to be used. These values were in good accordance with those derived from monomeric PBI crystals[30] and reported to be suitable for the calculations of short-range coupling energies.[17, 18, 31]

The short-range charge-transfer coupling J_{CT} was calculated by[28, 29]

$$J_{\mathrm{CT}} = -2\frac{t_{\mathrm{e}}t_{\mathrm{h}}}{E_{\mathrm{CT}} - E_{\mathrm{S_1}}}. \tag{6}$$

The resulting total coupling J_{Total} was determined as the sum of J_{Coul} and J_{CT}. The molecular structures obtained from DFT geometry optimizations were used. The calculations were

performed by extracting the monomeric layers from **PBI-2** and **PBI-5** and replacing the spacer moieties by phenoxy units. The solubilizing alkyl chains were replaced by methyl groups.

The transition to an electronic state is not predominated by one molecular orbital (MO) pair. Therefore, the natural transition orbital (NTO) method[16] has been chosen, to separately perform unitary transformations for occupied MOs and virtual MOs as implemented in the Multiwfn 3.8 program package (Supplementary Figure 14). To achieve acceptable accuracy, for the TD-DFT calculations (wB97X-D/def2-SVP) in Tol (PCM) all configuration coefficients whose magnitude is larger than 0.001 have been taken into account.[20]

Supplementary Table 2 summarizes the excited state energies and oscillator strengths of the six lowest excited states obtained by TD-DFT calculations (wB97X-D/def2-SVP) for **PBI-2** and **PBI-5** in Tol (PCM).

Supplementary Table 1. Quantum chemical calculation of coupling values. Calculated Exciton Coupling Energies and Hole/Electron Integrals for **PBI-2** and **PBI-5** in Tol.

Couplings [cm^{-1}] in Tol	J_{Total}	J_{Coul}[a]	J_{CT}[b]	t_e	t_h
**PBI-2** (close)	111	479	–368	513	1147
**PBI-5** (close)	17	473	–456	837	1264
**PBI-5** (far, 3 units)	–85.3	–85.3	–		
**PBI-5** (far, 5 units)	–2.55	–2.55	–		

[a] Calculated by the transition charge method (averaged). [b] Determined based on eq. 6 (averaged).

Supplementary Table 2. Quantum chemical calculation of excited states. Summary of the energy and oscillator strength of the lowest six calculated excited states applying TD-DFT calculations (ωB97X-D/def2-SVP, PCM) in Tol for **PBI-2** to **PBI-5**.

	State	Tol<br>Energy [eV]	Oscillator Strength
**PBI-2**	S_1	2.4890	1.2572
	S_2	2.4934	0.0225
	S_3	2.9869	0.0055
	S_4	3.1363	0.3496
	S_5	3.5723	0.0415
	S_6	3.6236	0.0124
**PBI-5**	S_1	2.2332	1.5147
	S_2	2.3380	0.0000
	S_3	2.4146	0.6284
	S_4	2.4707	0.0000
	S_5	2.4857	0.4299
	S_6	2.8078	0.0000

5. Steady-State Absorption and Fluorescence Spectroscopy

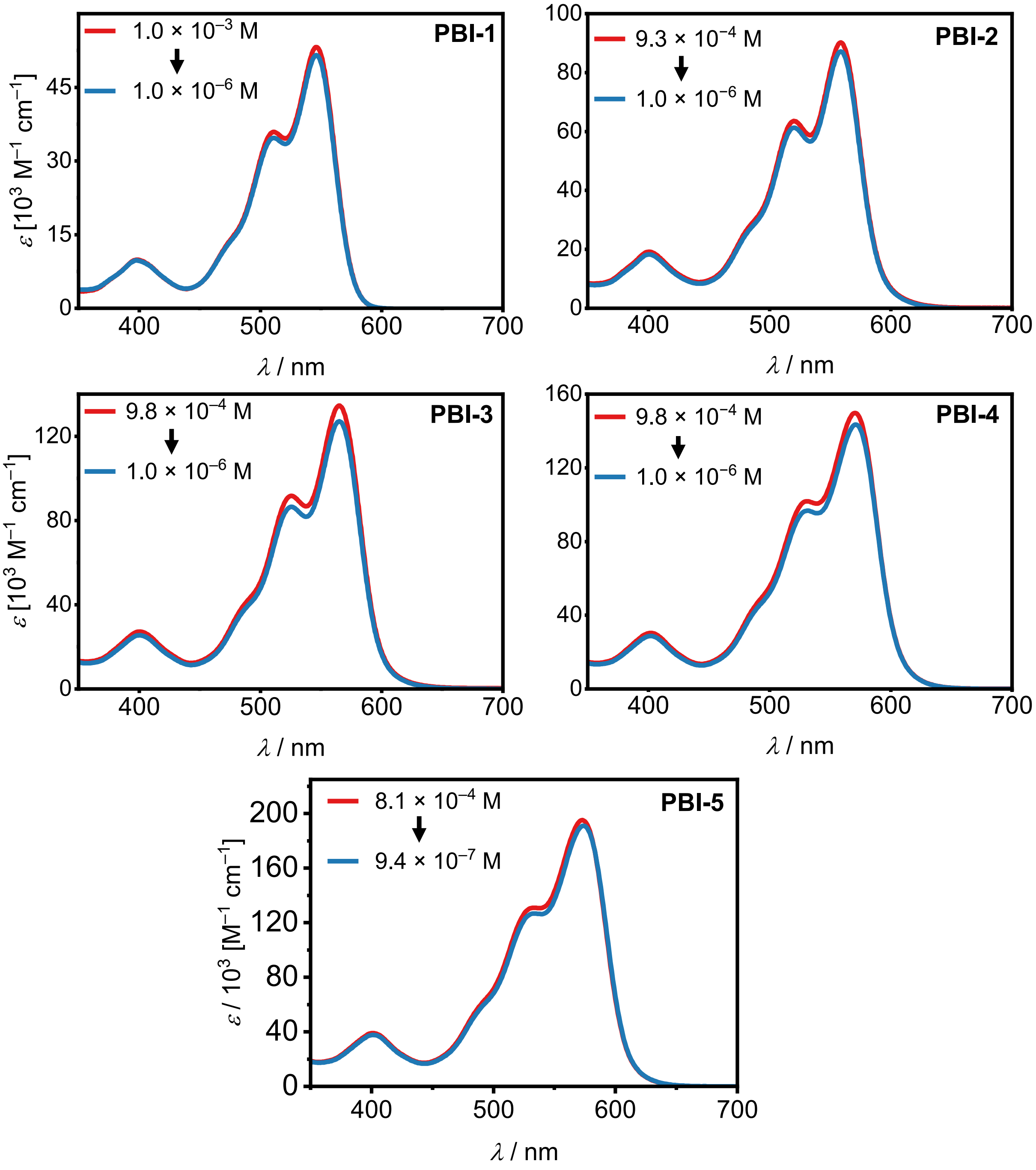

Supplementary Figure 15. Optical characterization by steady-state absorption spectroscopy. Concentration-dependent UV/Vis absorption spectra of **PBI-1** (top left, $c_0 = 9.3 \times 10^{-4}$ M to $c_0 = 1.0 \times 10^{-6}$ M), **PBI-2** (top right, $c_0 = 9.3 \times 10^{-4}$ M to $c_0 = 1.0 \times 10^{-6}$ M), **PBI-3** (middle left, $c_0 = 9.8 \times 10^{-4}$ M to $c_0 = 1.0 \times 10^{-6}$ M) and **PBI-4** (middle right, $c_0 = 9.8 \times 10^{-4}$ M to $c_0 = 1.0 \times 10^{-6}$ M) and **PBI-5** (bottom, $c_0 = 8.1 \times 10^{-4}$ M to $c_0 = 9.4 \times 10^{-7}$ M) in TCE at 298 K.

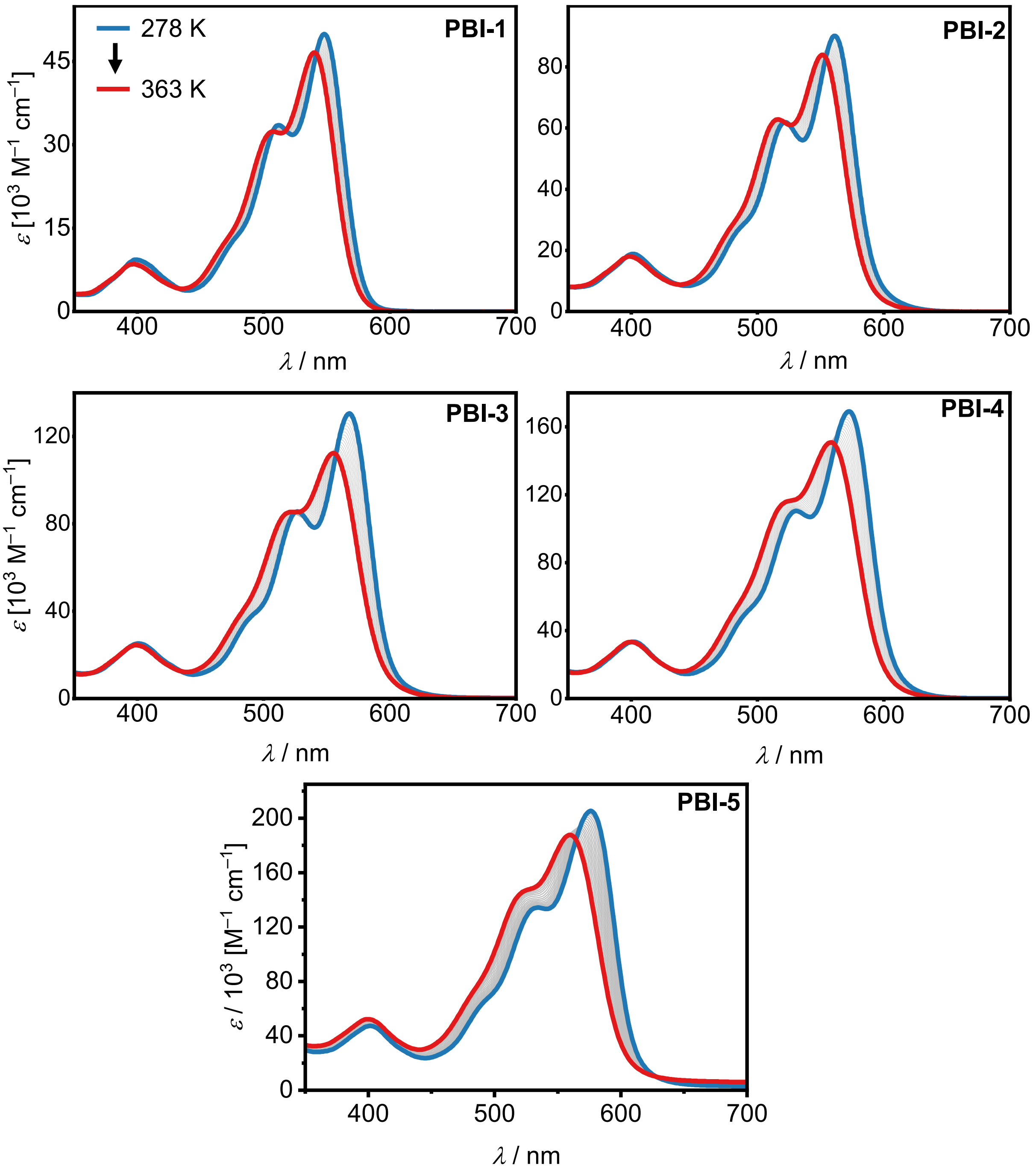

Supplementary Figure 16. Optical characterization by steady-state absorption spectroscopy. Temperature-dependent UV/Vis absorption spectra of **PBI-1** (top left), **PBI-2** (top right), **PBI-3** (middle left), **PBI-4** (middle right) and **PBI-5** (bottom) in TCE (c_0 = 1.0 × 10^{-5} M) in steps of 5 K between 278 K (blue) and 363 K (red), density corrected.

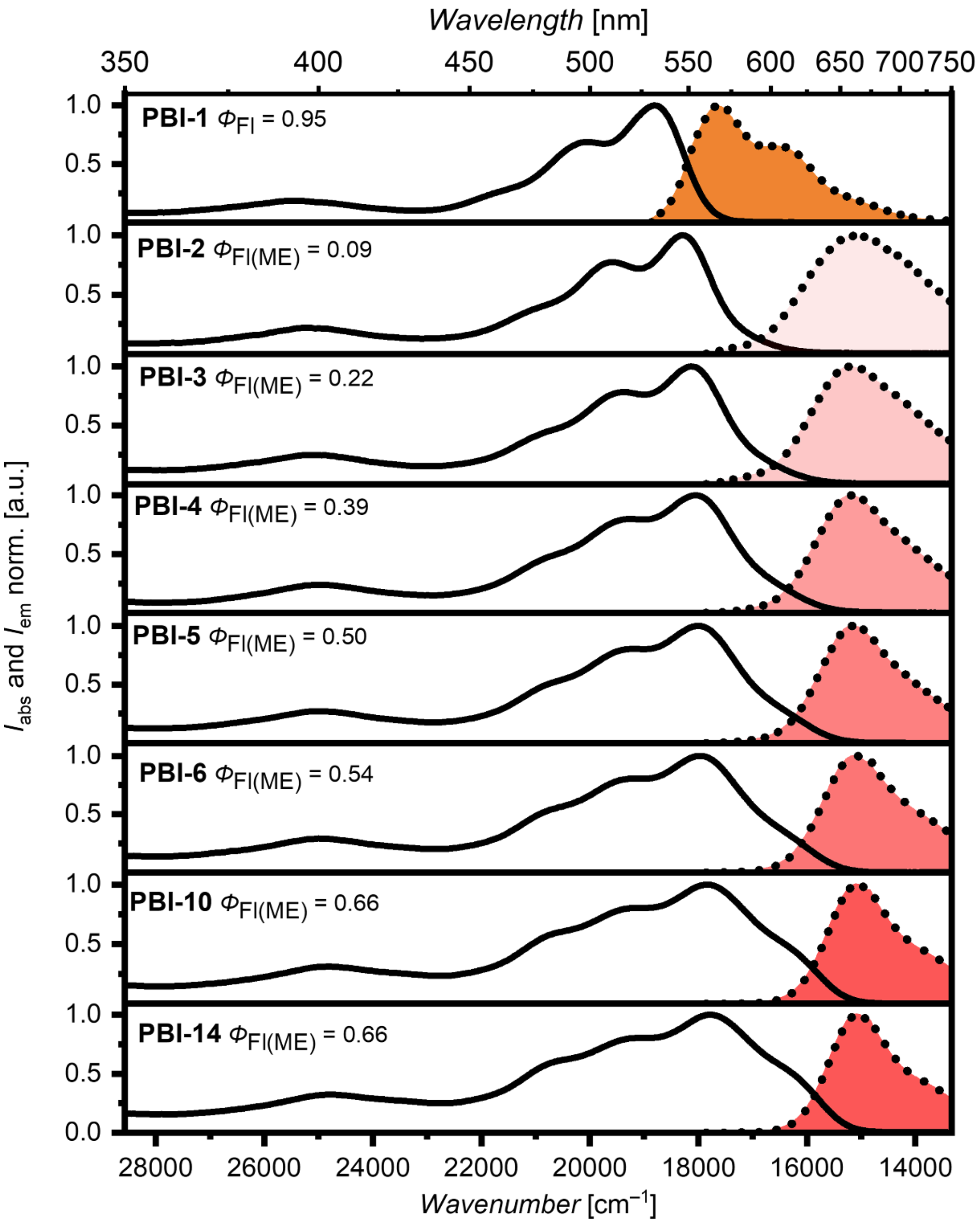

Supplementary Figure 17. Optical characterization by steady-state spectroscopy. Normalized UV/Vis absorption (c_0 = 1.0 × 10^{-5} M) (black) and fluorescence (*OD* < 0.05) (colored) spectra of **PBI-N** (**N** = **1**, **2**, **3**, **4**, **5**, **6**, **10** and **14**) in THF with their relative fluorescence quantum yields of the LE (Φ_{FL}) or ME state ($\Phi_{FL(ME)}$).

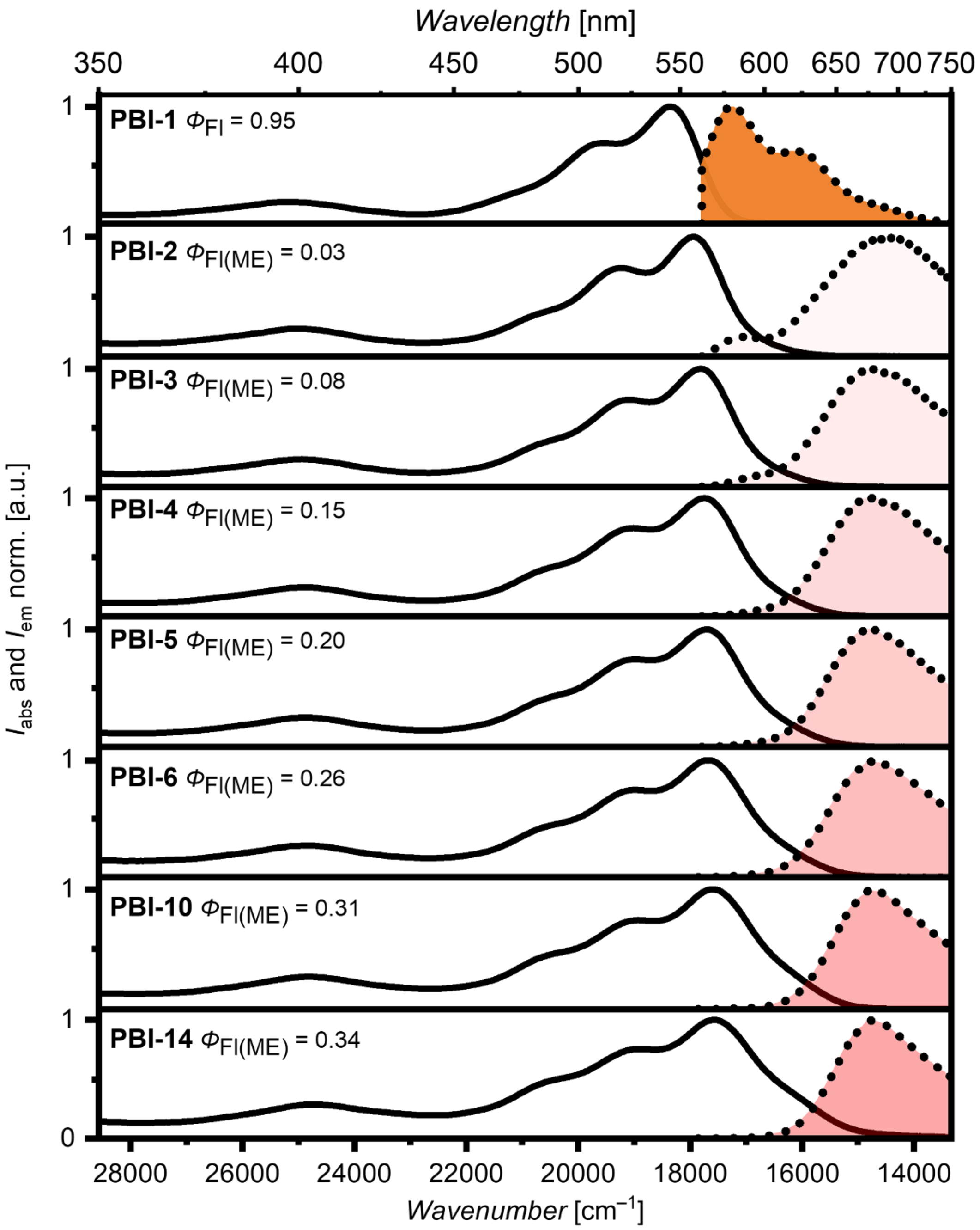

Supplementary Figure 18. Optical characterization by steady-state spectroscopy. Normalized UV/Vis absorption (c_0 = 1.0 × 10^{-5} M) (black) and fluorescence (*OD* < 0.05) (colored) spectra of **PBI-N** (**N**=**1**, **2**, **3**, **4**, **5**, **6**, **10** and **14**) in BCN with their relative fluorescence quantum yields of the LE (Φ_{FL}) or the ME state ($\Phi_{FL(ME)}$).

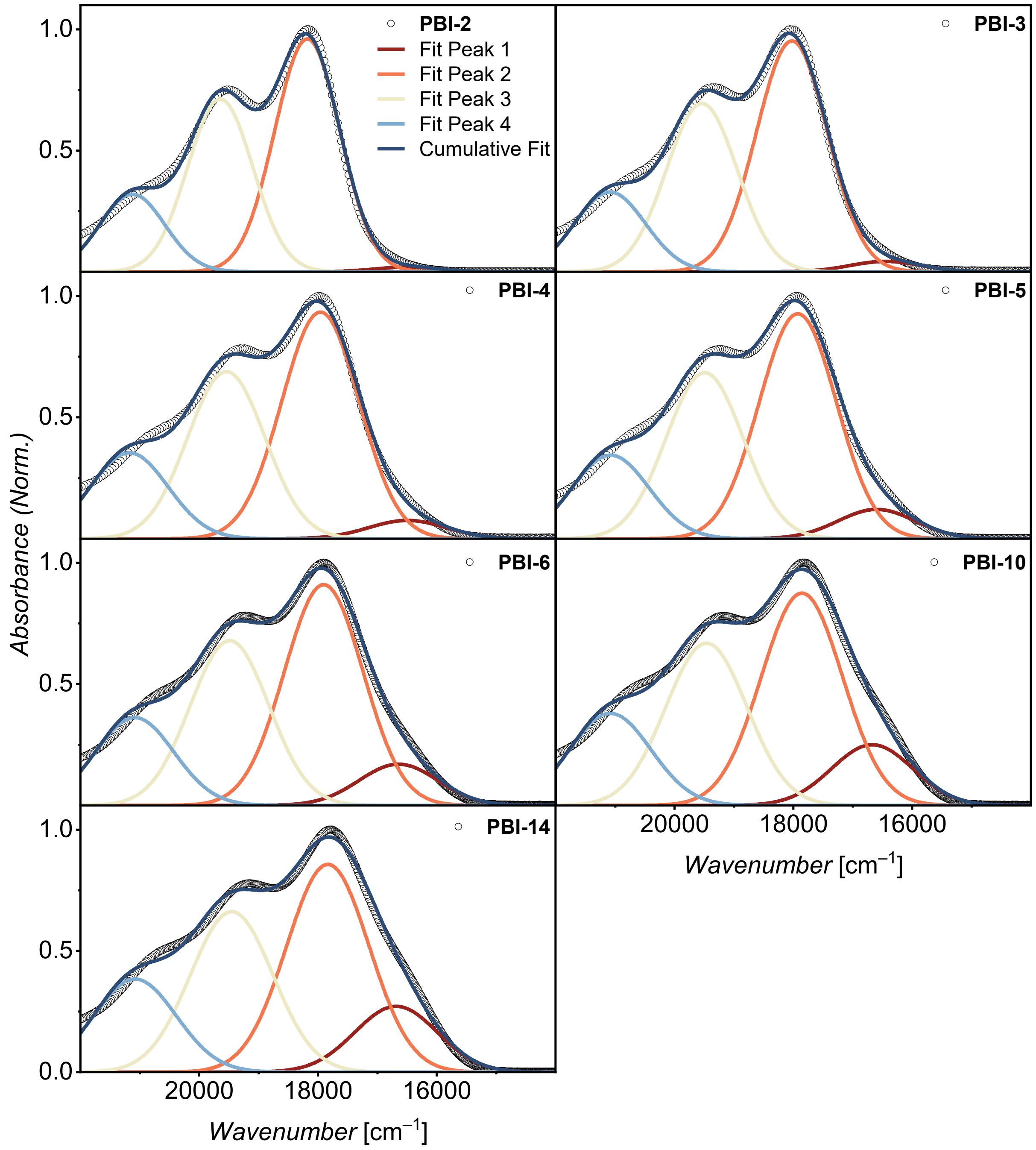

Supplementary Figure 19. Optical characterization by steady-state absorption spectroscopy. UV/Vis absorption spectra (black dots) of **PBI-2** (top left), **PBI-3** (top right), **PBI-4** (middle top left), **PBI-5** (middle top right), **PBI-6** (middle bottom left), **PBI-10** (middle bottom right) and **PBI-14** (bottom left) in Tol (c_0 = 1.0 × 10^{-5} M). The cumulative fit of the absorption spectra (gray) as the sum of the four Gaussian functions (red, orange, white and blue solid lines).

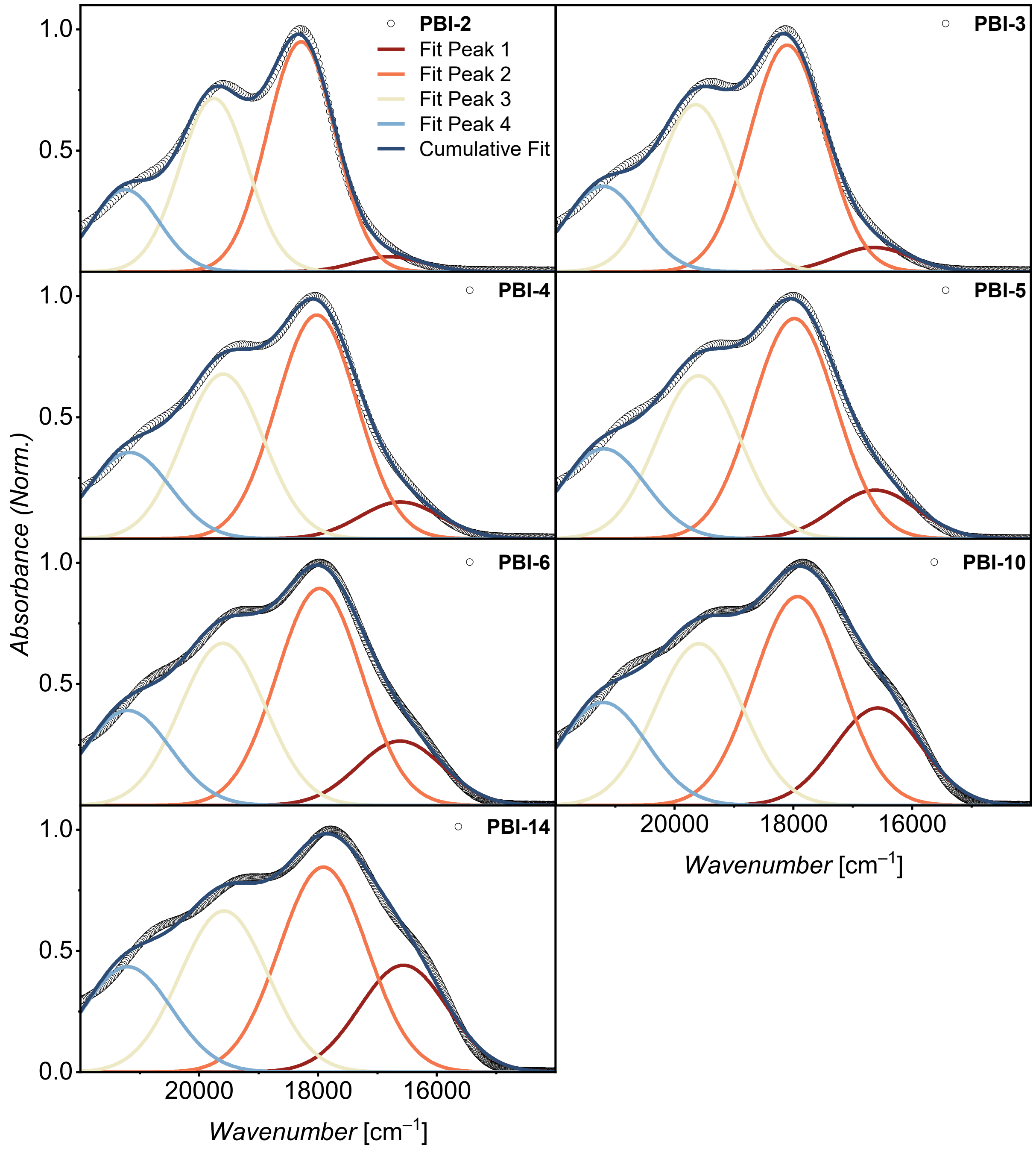

Supplementary Figure 20. Optical characterization by steady-state absorption spectroscopy. UV/Vis absorption spectra (black dots) of **PBI-2** (top left), **PBI-3** (top right), **PBI-4** (middle top left), **PBI-5** (middle top right), **PBI-6** (middle bottom left), **PBI-10** (middle bottom right) and **PBI-14** (bottom left) in THF (c_0 = 1.0 × 10^{-5} M). The cumulative fit of the absorption spectra (gray) as the sum of the four Gaussian functions (red, orange, white and blue solid lines).

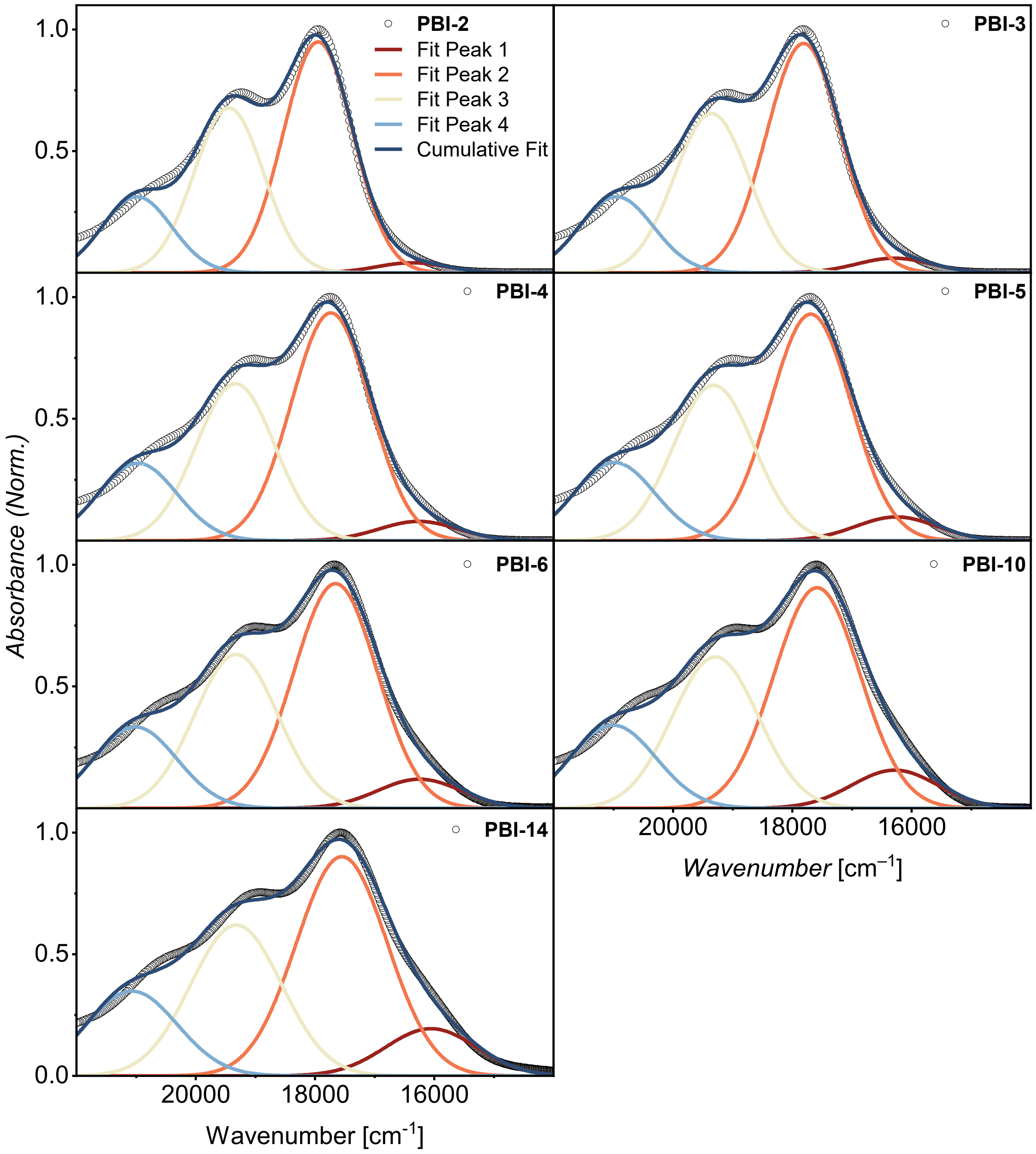

Supplementary Figure 21. Optical characterization by steady-state absorption spectroscopy. UV/Vis absorption spectra (black dots) of **PBI-2** (top left), **PBI-3** (top right), **PBI-4** (middle top left), **PBI-5** (middle top right), **PBI-6** (middle bottom left), **PBI-10** (middle bottom right) and **PBI-14** (bottom left) in BCN (c_0 = 1.0 × 10^{-5} M). The cumulative fit of the absorption spectra (gray) as the sum of the four Gaussian functions (red, orange, white and blue solid lines).

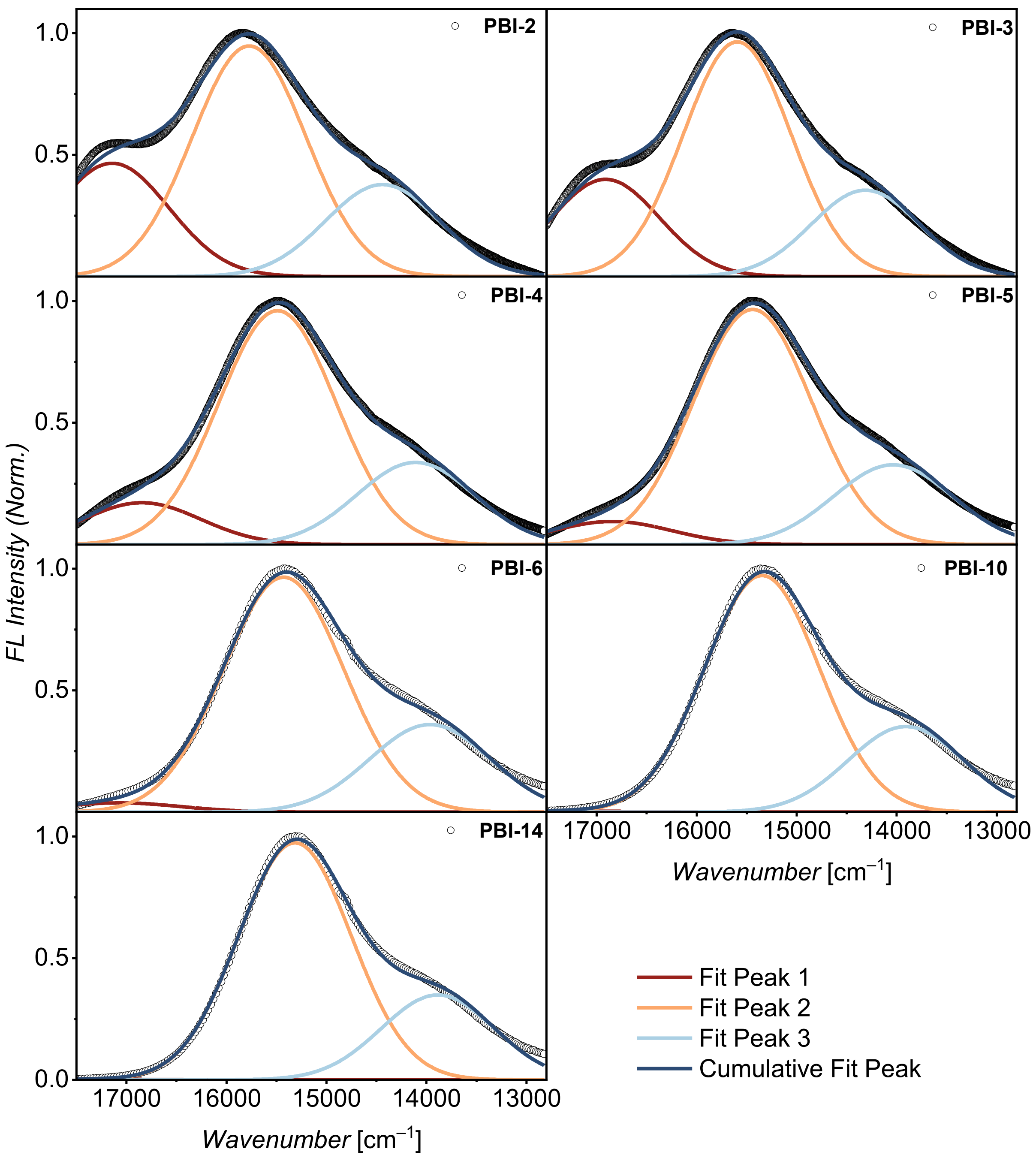

Supplementary Figure 22. Optical characterization by steady-state fluorescence spectroscopy. Fluorescence spectra (black dots) of **PBI-2** (top left), **PBI-3** (top right), **PBI-4** (middle top left), **PBI-5** (middle top right), **PBI-6** (middle bottom left), **PBI-10** (middle bottom right) and **PBI-14** (bottom left) in Tol (*OD* < 0.05). The cumulative fit of the absorption spectra (gray) as the sum of the three Gaussian functions (red, orange, white and blue solid lines).

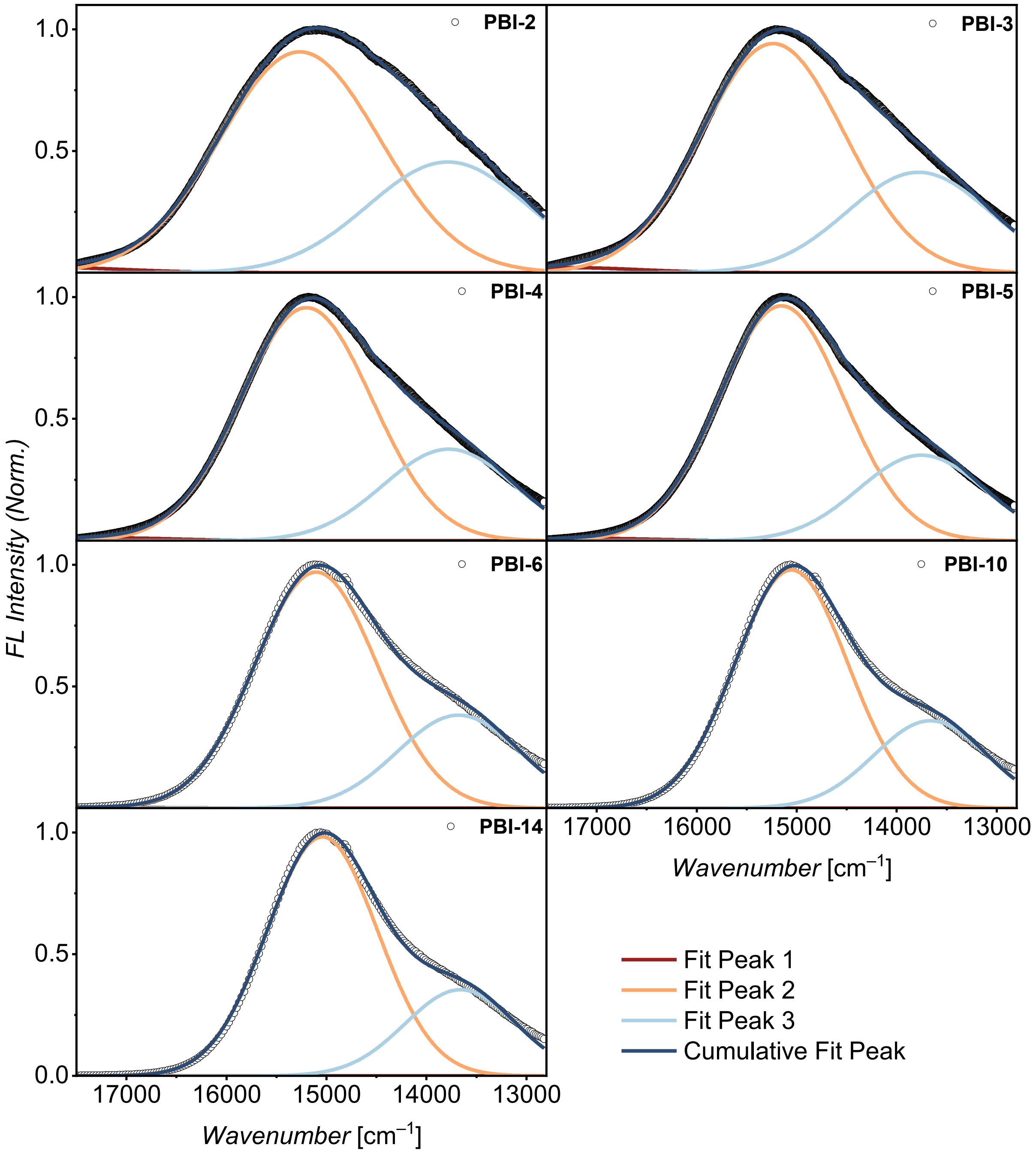

Supplementary Figure 23. Optical characterization by steady-state fluorescence spectroscopy. Fluorescence spectra (black dots) of **PBI-2** (top left), **PBI-3** (top right), **PBI-4** (middle top left), **PBI-5** (middle top right), **PBI-6** (middle bottom left), **PBI-10** (middle bottom right) and **PBI-14** (bottom left) in THF (*OD* < 0.05). The cumulative fit of the absorption spectra (gray) as the sum of the three Gaussian functions (red, orange, white and blue solid lines).

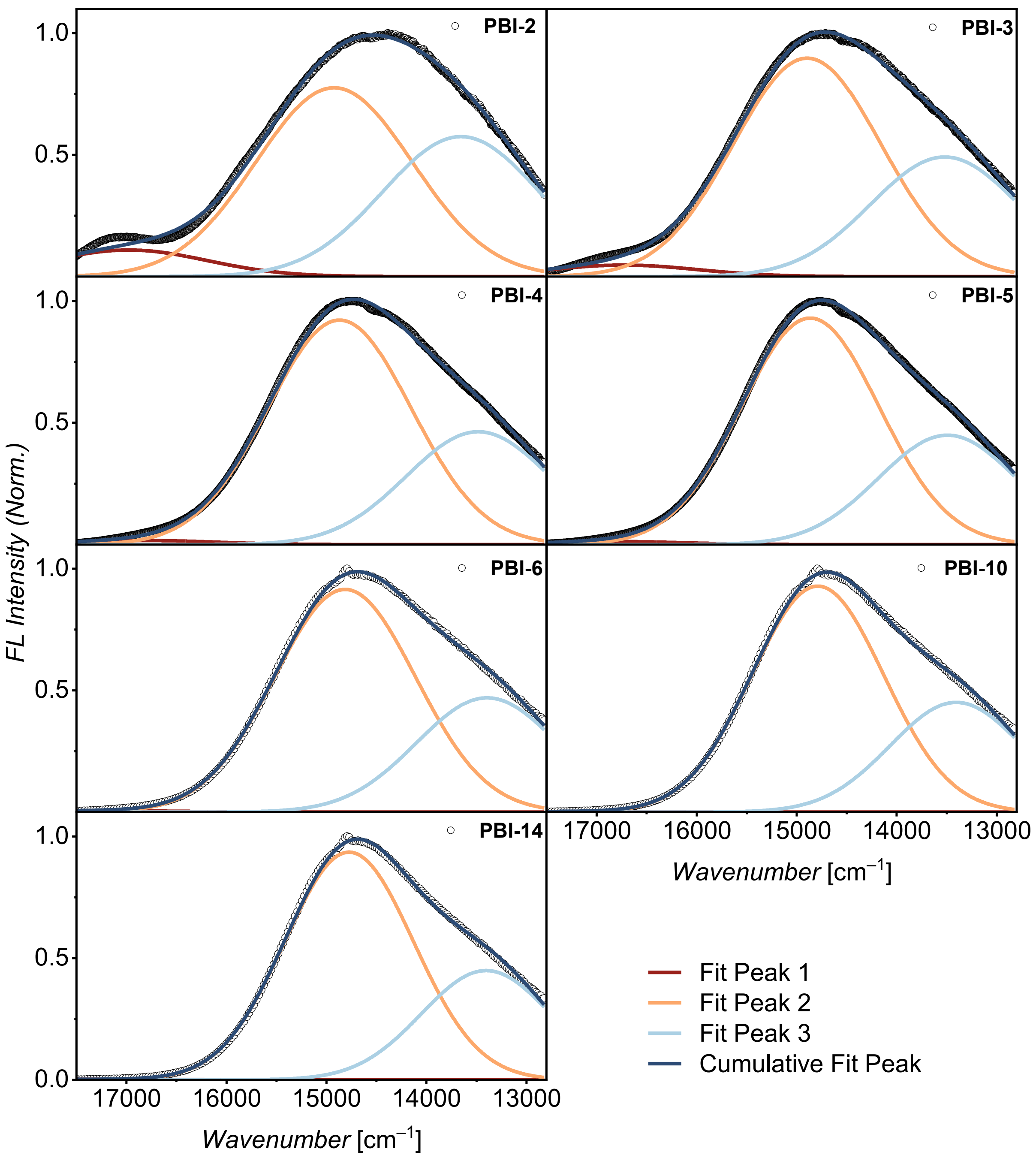

Supplementary Figure 24. Optical characterization by steady-state fluorescence spectroscopy. Fluorescence spectra (black dots) of **PBI-2** (top left), **PBI-3** (top right), **PBI-4** (middle top left), **PBI-5** (middle top right), **PBI-6** (middle bottom left), **PBI-10** (middle bottom right) and **PBI-14** (bottom left) in BCN (*OD* < 0.05). The cumulative fit of the absorption spectra (gray) as the sum of the three Gaussian functions (red, orange, white and blue solid lines).

Supplementary Table 3. Optical characterization in Tol. Summary of spectroscopic data obtained from UV/Vis and fluorescence measurements of **PBI-2** to **PBI-14** in toluene at 298 K.

	**PBI-2**	**PBI-3**	**PBI-4**	**PBI-5**	**PBI-6**	**PBI-10**	**PBI-14**
$Abs_{max}{}^{a}$ / eV	2.25	2.23	2.23	2.22	2.22	2.21	2.20
FWHM(Abs.)^{a,d} / cm^{-1}	1293.1	1407	1535.2	1531.4	1560.4	1612.5	1636.7
$[Abs_{CT} / Abs_{0\text{–}0}]^{a,\,d}$	0.02	0.04	0.08	0.13	0.19	0.29	0.32
$Em_{max}{}^{b}$ / eV	1.96	1.93	1.92	1.91	1.91	1.90	1.90
$FWHM_{FL(ME)}{}^{b,d}$ / cm^{-1}	1240.5	1224.4	1280.2	1289.8	1302.7	1277.2	1258.6
$\Phi_{Fl}{}^{b}$ / %	0.47	0.56	0.57	0.63	0.73	0.75	0.75
$\tau_{Fl(ME)}$./ ns	10.0	9.6	9.6	9.1	8.0	7.3	8.0
$k_r{}^{c}$ / s^{-1}	4.74×10^7	5.79×10^7	5.96×10^7	6.96×10^7	9.12×10^7	10.0×10^7	9.37×10^7
$k_{nr}{}^{c}$ / s^{-1}	5.26×10^7	4.63×10^7	4.46×10^7	4.03×10^7	3.37×10^7	3.37×10^7	3.12×10^7

^{a} UV/Vis $c_0 = 1.00 \times 10^{-5}$ M. ^{b} Fluorescence under high diluted conditions $OD < 0.05$. ^{c} Determined according to $k_r = \Phi_{Fl(ME)}\ \tau^{-1}$ and $k_{nr} = \tau^{-1} - k_r$. ^{d} The parameters were estimated by fitting the absorption and fluorescence spectra using multiple Gaussian functions.

Supplementary Table 4. Optical characterization in THF. Summary of spectroscopic data obtained from UV/Vis and fluorescence measurements of **PBI-2** to **PBI-14** in THF at 298 K.

	**PBI-2**	**PBI-3**	**PBI-4**	**PBI-5**	**PBI-6**	**PBI-10**	**PBI-14**
$Abs_{max}{}^{a}$ / eV	2.27	2.25	2.24	2.23	2.23	2.21	2.21
FWHM(Abs.)^{a,d} / cm^{-1}	1348.1	1494.5	1599.1	1657.8	1689.1	1730.3	1745.6
$[Abs_{CT} / Abs_{0\text{–}0}]^{a,\,d}$	0.07	0.11	0.16	0.22	0.30	0.47	0.52
$Em_{max}{}^{b,d}$ / eV	1.89	1.89	1.88	1.88	1.87	1.87	1.86
$FWHM_{FL(ME)}{}^{b}$ / cm^{-1}	1645.7	1457.6	1389.0	1332.0	1356.7	1270.6	1241.1
$\Phi_{Fl}{}^{b}$ / %	0.09	0.22	0.39	0.50	0.54	0.66	0.66
$\tau_{Fl(ME)}$./ ns	6.4	8.5	9.3	9.2	7.8	7.2	8.3
$k_r{}^{c}$ / s^{-1}	1.45×10^7	2.60×10^7	4.20×10^7	5.42×10^7	6.90×10^7	9.16×10^7	7.95×10^7
$k_{nr}{}^{c}$ / s^{-1}	1.42×10^8	9.17×10^7	6.55×10^7	5.45×10^7	5.88×10^7	4.72×10^7	4.09×10^7

^{a} UV/Vis $c_0 = 1.00 \times 10^{-5}$ M. ^{b} Fluorescence under high diluted conditions $OD < 0.05$. ^{c} Determined according to $k_r = \Phi_{Fl(ME)}\ \tau^{-1}$ and $k_{nr} = \tau^{-1} - k_r$. ^{d} The parameters were estimated by fitting the absorption and fluorescence spectra using multiple Gaussian functions.

Supplementary Table 5. Optical characterization in BCN. Summary of spectroscopic data obtained from UV/Vis and fluorescence measurements of **PBI-2** to **PBI-14** in BCN at 298 K.

	**PBI-2**	**PBI-3**	**PBI-4**	**PBI-5**	**PBI-6**	**PBI-10**	**PBI-14**
Abs_{max}[a] / eV	2.23	2.21	2.20	2.19	2.19	2.18	2.18
FWHM(Abs.)[a,d] / cm^{-1}	1374.2	1465.1	1556.6	1598.7	1649.6	1702.0	1795.1
[Abs_{CT} / Abs_{0-0}][a, d]	0.04	0.06	0.08	0.10	0.13	0.17	0.22
Em_{max}[b] / eV	1.85	1.85	1.84	1.84	1.84	1.83	1.83
$FWHM_{FL(ME)}$[b,d] / cm^{-1}	1711.7	1631.2	1604.7	1557.6	1553.1	1478.5	1435.2
Φ_{Fl}[b] / %	0.03	0.08	0.15	0.20	0.26	0.31	0.34
$\tau_{Fl(ME)}$./ ns	3.3	4.7	5.9	6.2	5.2	-	-
k_r[c] / s^{-1}	7.99×10^6	1.71×10^7	2.51×10^7	3.30×10^7	5×10^7	-	-
k_{nr}[c] / s^{-1}	2.95×10^8	1.96×10^8	1.44×10^8	1.28×10^8	14.2×10^7	-	-

[a] UV/Vis $c_0 = 1.00 \times 10^{-5}$ M. [b] Fluorescence under high diluted conditions $OD < 0.05$. [c] Determined according to $k_r = \Phi_{Fl(ME)}\ \tau^{-1}$ and $k_{nr} = \tau^{-1} - k_r$. [d] The parameters were estimated by fitting the absorption and fluorescence spectra using multiple Gaussian functions.

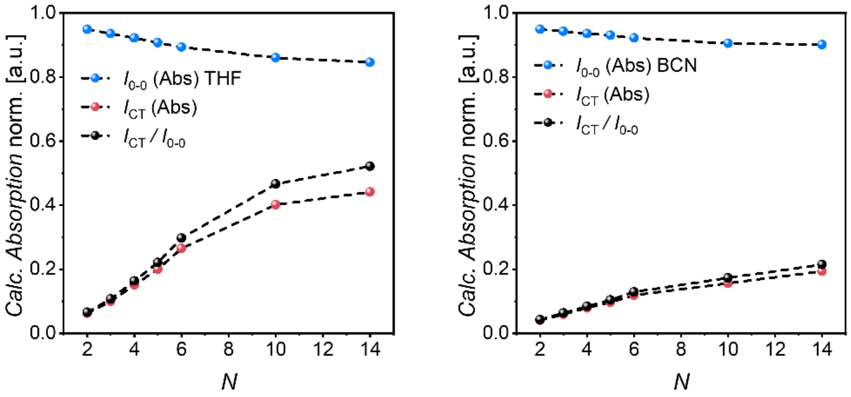

Supplementary Figure 25. Optical characterization by steady-state absorption spectroscopy. Absorption maxima of the 0–0 transition ($Abs_{0–0}$, blue), CT band (Abs_{CT}, red) and intensity ratio of Abs_{CT} (600 nm) over $Abs_{0–0}$ (550 nm) (black) in THF (left) and BCN (right) as a function of molecular size. The parameters were estimated by fitting of absorption and fluorescence spectra using multiple Gaussian functions.

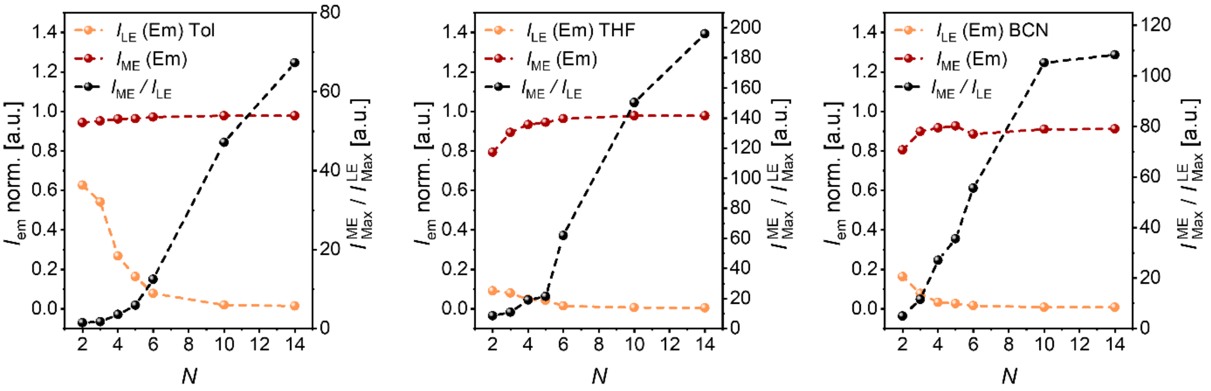

Supplementary Figure 26. Optical characterization by steady-state fluorescence spectroscopy. Emission maxima of the LE transition (I_{LE}, orange), ME band (I_{ME}, red) and intensity ratio of I_{ME} over I_{LE} (black) in Tol (left), THF (middle) and BCN (right) as a function of molecular size. The parameters were estimated by fitting of absorption and fluorescence spectra using multiple Gaussian functions.

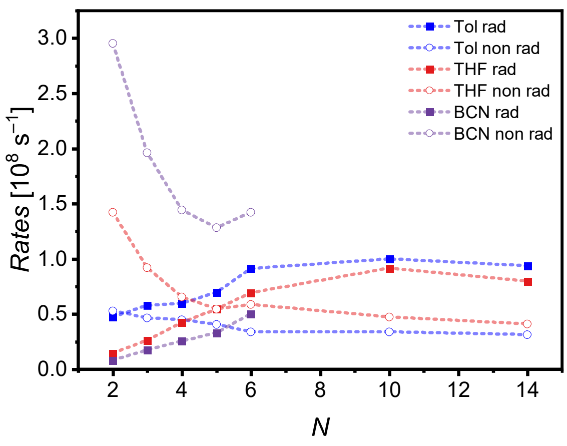

Supplementary Figure 27. Optical characterization by steady-state fluorescence spectroscopy. Radiative (square) and nonradiative (circle) rate constants in Tol (blue), THF (red) and BCN (purple) as a function of molecular size.

6. Time-Resolved Spectroscopy

fs-Transient Absorption Spectroscopy in Toluene

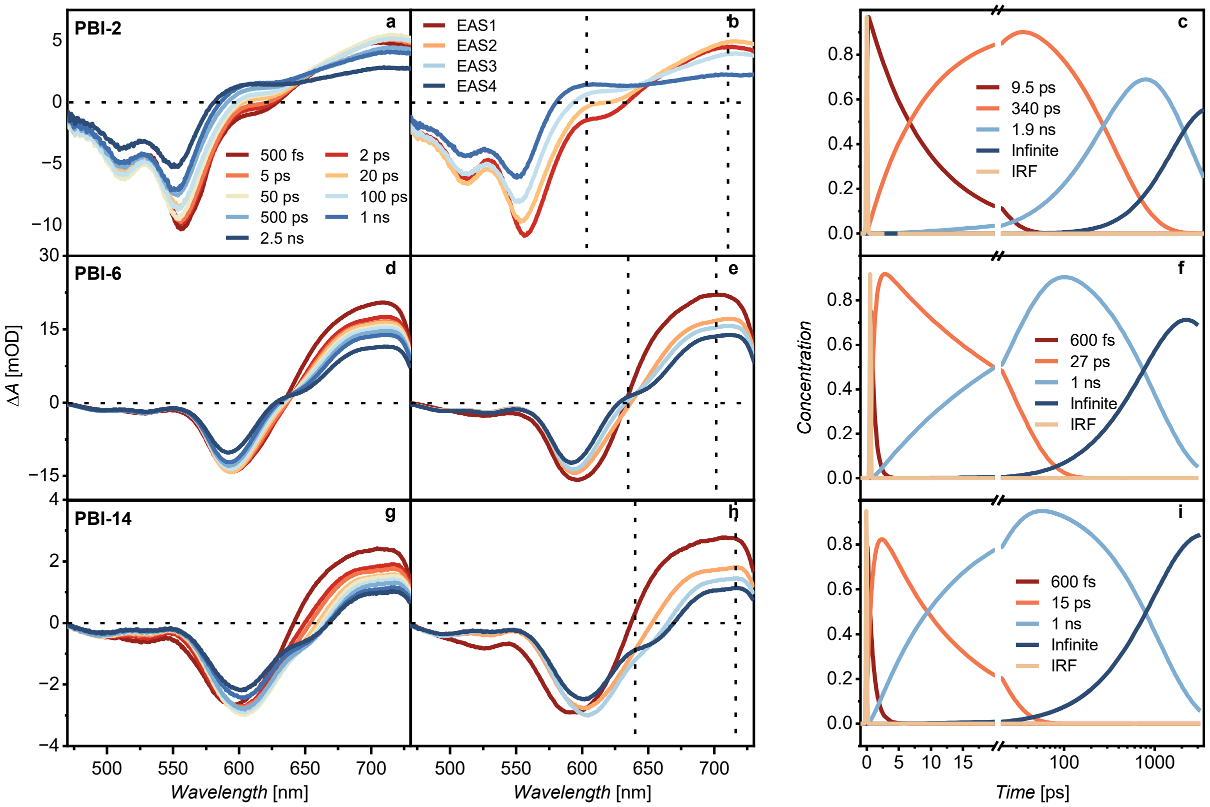

Supplementary Figure 28. Optical characterization by time-resolved spectroscopy. Transient absorption results of **PBI-2**, **PBI-6** and **PBI-14** in Tol using an excitation pump of 520 nm. **a** TA results of **PBI-2** in Tol. **b**, **c** Evolution associated spectra (EAS) and the corresponding concentration fitted by using the glotaran program. The respective EAS correspond to the excited-state species (EAS1, LE state, 9.5 ps; EAS2, LE-CT mixed state, 340 ps; EAS3, ME state,1.9 ns; EAS4, relaxed ME state; infinite). **d** TA results of **PBI-6** in Tol. **e**, **f** EAS and the corresponding concentration fitted by using the glotaran program, related to the excited-state species (EAS1, LE-CT mixed state, 600 fs; EAS2, ME state, 27 ps; EAS3, relaxed ME state, 1 ns; EAS4, relaxed ME state, infinite). **g** TA results of **PBI-14** in Tol. **h**, **i** The respective EAS and the corresponding concentration fitted by using the glotaran program, related to the excited-state species (EAS1, LE-CT mixed state, 600 fs; EAS2, ME state, 15 ps; EAS3, relaxed ME state, 1 ns; EAS4, relaxed ME state, infinite).

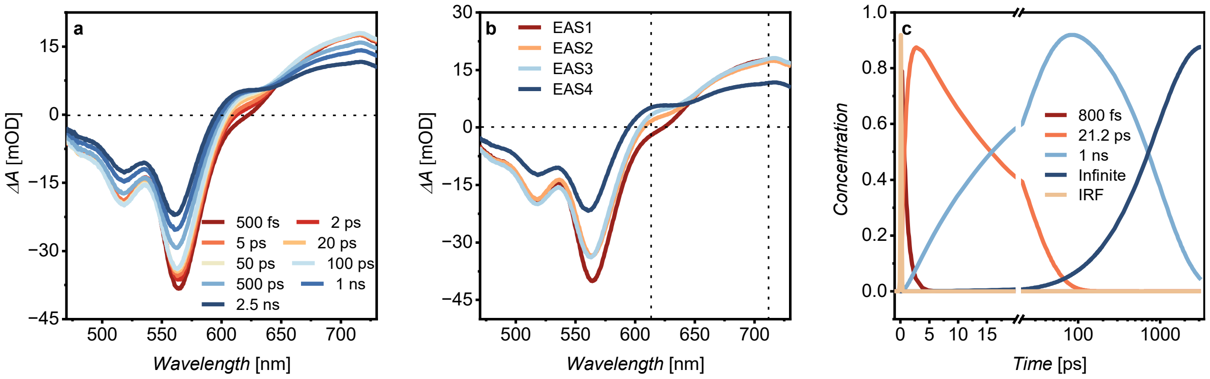

Supplementary Figure 29. Optical characterization by time-resolved spectroscopy. Transient absorption results of **PBI-3** in Tol using an excitation pump of 520 nm. **a** TA results of **PBI-3** in Tol. **b**, **c** Evolution associated spectra and the corresponding concentration fitted by using the glotaran program. The respective EAS corresponds to the excited-state species (EAS1, initial S1, 800 fs; EAS2, LE-CT mixed state, 21.2 ps; EAS3, ME state, 1 ns; EAS4, relaxed ME state; infinite).

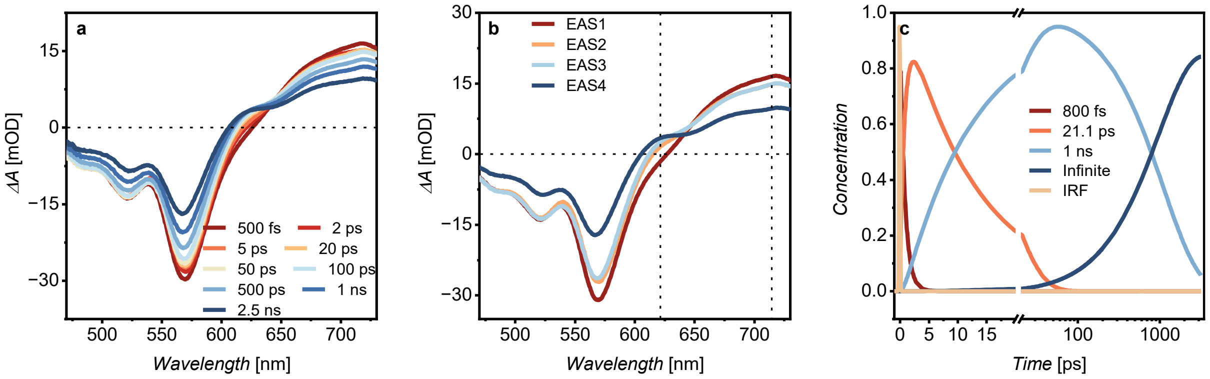

Supplementary Figure 30. Optical characterization by time-resolved spectroscopy. Transient absorption results of **PBI-4** in Tol using an excitation pump of 520 nm. **a** TA results of **PBI-4** in Tol. **b**, **c** Evolution associated spectra and the corresponding concentration fitted by using the glotaran program. The respective EAS corresponds to the excited-state species (EAS1, initial S1, 800 fs; EAS2, LE-CT mixed state, 21.1 ps; EAS3, ME state, 1 ns; EAS4, relaxed ME state; infinite).

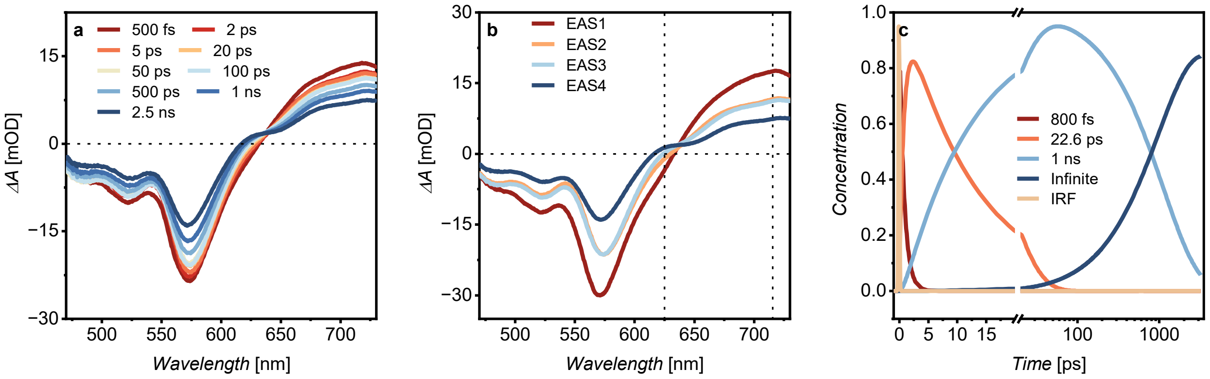

Supplementary Figure 31. Optical characterization by time-resolved spectroscopy. Transient absorption results of **PBI-5** in Tol using an excitation pump of 520 nm. **a** TA results of **PBI-5** in Tol. **b**, **c** The respective EAS and the corresponding concentration fitted by using the glotaran program, related to the excited-state species (EAS1, LE-CT mixed state, 800 fs; EAS2, ME state, 22.6 ps; EAS3, relaxed ME state, 1 ns; EAS4, relaxed ME state, infinite).

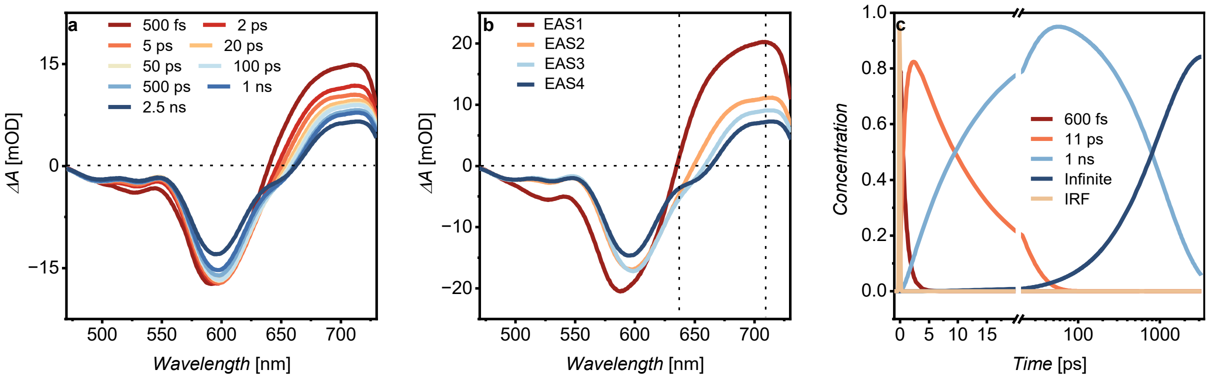

Supplementary Figure 32. Optical characterization by time-resolved spectroscopy. Transient absorption results of **PBI-10** in Tol using an excitation pump of 520 nm. **a** TA results of **PBI-10** in Tol. **b**, **c** The respective EAS and the corresponding concentration fitted by using the glotaran program, related to the excited-state species (EAS1, LE-CT mixed state, 600 fs; EAS2, ME state, 11 ps; EAS3, relaxed ME state, 1 ns; EAS4, relaxed ME state, infinite).

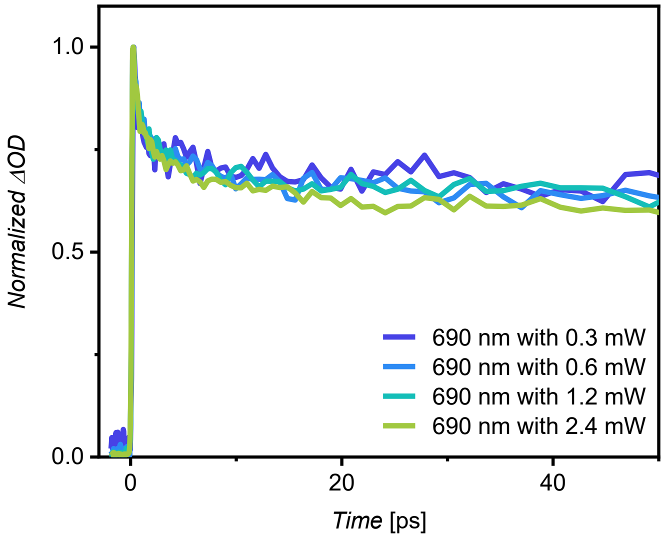

Supplementary Figure 33 Optical characterization by time-resolved spectroscopy. Pump-power independent kinetic traces of **PBI-14** in Tol.

fs-Transient Absorption Spectroscopy in THF

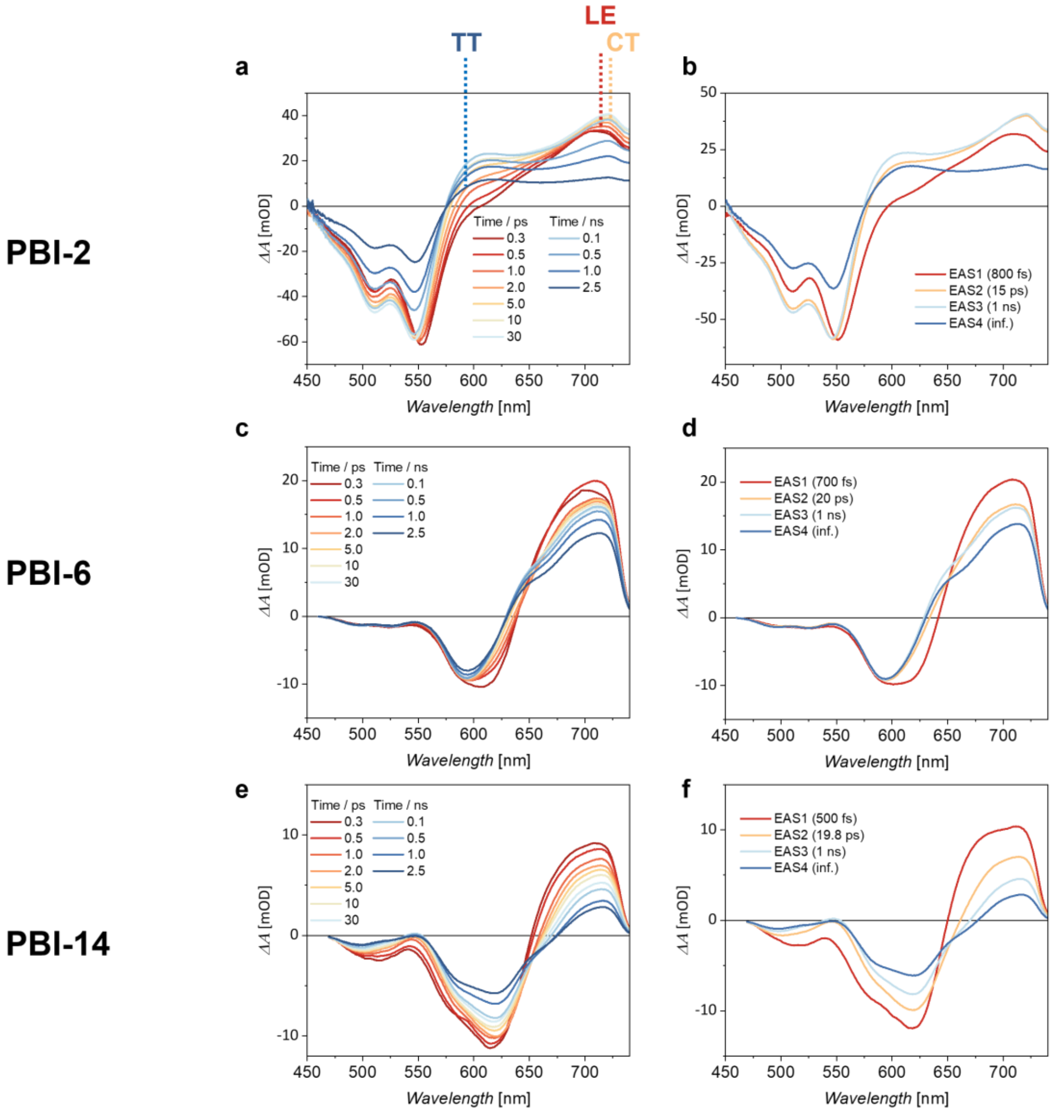

Supplementary Figure 34. Optical characterization by time-resolved spectroscopy. fs-Transient absorption results **PBI-2, PBI-6,** and **PBI-14** in THF using an excitation pump of 520 nm. **a** The representative TA spectra of **PBI-2**. **b** Evolution associated spectra (EAS) of **PBI-2** fitted by using the glotaran program, corresponding to the excited-state species (EAS1, initial S_1, 800 fs; EAS2, LE-CT mixed state, 15 ps; EAS3, ^{1}(ME)state, 1 ns; EAS4, relaxed ^{1}(ME)state; infinite). **c** The representative TA spectra of **PBI-6**. **d** Evolution associated spectra (EAS) of **PBI-6** fitted by using the glotaran program, corresponding to the excited-state species (EAS1, initial LE-CT mixed state, 800 fs; EAS2, ^{1}(ME) state, 20 ps; EAS3, relaxed ^{1}(ME) state, 1 ns; EAS4, relaxed ^{1}(ME) state; infinite). **e** The representative TA spectra of **PBI-14**. **f** Evolution associated spectra (EAS) of **PBI-14** fitted by using the glotaran program, corresponding to the excited-state species (EAS1, initial LE-CT mixed state, 500 fs; EAS2, ^{1}(ME) state, 19.8 ps; EAS3, relaxed ^{1}(ME) state, 1 ns; EAS4, relaxed ^{1}(ME) state; infinite. The dashed lines indicate the contributions of the LE (blue), CT (red), and triplet-pair (TT, purple) states.

We assign the ^{1}(ME) state as a multiconfigurational excited state involving contributions from locally excited (LE), charge-transfer (CT), and triplet-pair (TT) configurations. This assignment is further supported by fs-transient absorption measurements in a more polar solvent (THF), where CT-related features become more pronounced. For **PBI-2**, the excited-state absorption (ESA) at ~720 nm rises within 0.8 ps, marking the formation of an LE–CT mixed state facilitated by solvation and structural relaxation. This is followed by the growth of an ESA band near ~580 nm, characteristic of multiexciton (ME) generation (Supplementary Figure 34), consistent with previous reports on PBI dimers. The ME generation process is strongly influenced by solvent polarity, accelerating dramatically from ~330 ps in Tol to ~15 ps in THF. The appearance of a PBI anion-like absorption at ~720 nm in THF further corroborates a CT-assisted MEG mechanism.

In contrast, elongated stacks exhibit distinct excited-state dynamics. At ~300 fs, the TA spectra display ground-state bleaching (GSB) below 700 nm, deviating from the steady-state absorption profile and revealing new ESA features around ~580 nm, indicative of enhanced electronic coupling. The absence of any pump-fluence dependence, together with consistent excitation-wavelength-dependent fluorescence spectra, rules out the involvement of exciton–exciton annihilation or structural heterogeneity. Moreover, the immediate appearance (within ~100 fs) of the ~720 nm CT band demonstrates that LE and CT contributions are already mixed in the Franck–Condon (FC) state. In **PBI-6**, a biphasic MEG process is observed with components on sub-picosecond and ~5 ps timescales, accompanied by a distinct isosbestic point at ~720 nm. The broad ESA features observed here reflect efficient mixing of LE, CR, and TT adiabats in the ME state, in sharp contrast to the more localized behavior in **PBI-2**. These findings clearly highlight enhanced electronic communication within extended PBI stacks. Together with the results obtained in toluene, these observations elucidate the evolution of excitonic states in null-type PBI aggregates. With increasing CT admixture in the initial S_1 state, the rate of ME generation accelerates dramatically, from 15–330 ps in **PBI-2** to 500–800 fs in **PBI-6**, reflecting enhanced nonadiabatic coupling between the S_1 and ME states. This acceleration reaches saturation at **PBI-6**. While stronger CT coupling expedites ME formation, relaxation within the ME manifold induces two correlated effects:

(i) an increase in emission quantum yield (Fig. 3c, main text), and (ii) a concomitant decrease in free triplet yield (Supplementary Figure 42–44).

ns-Transient Absorption Spectroscopy

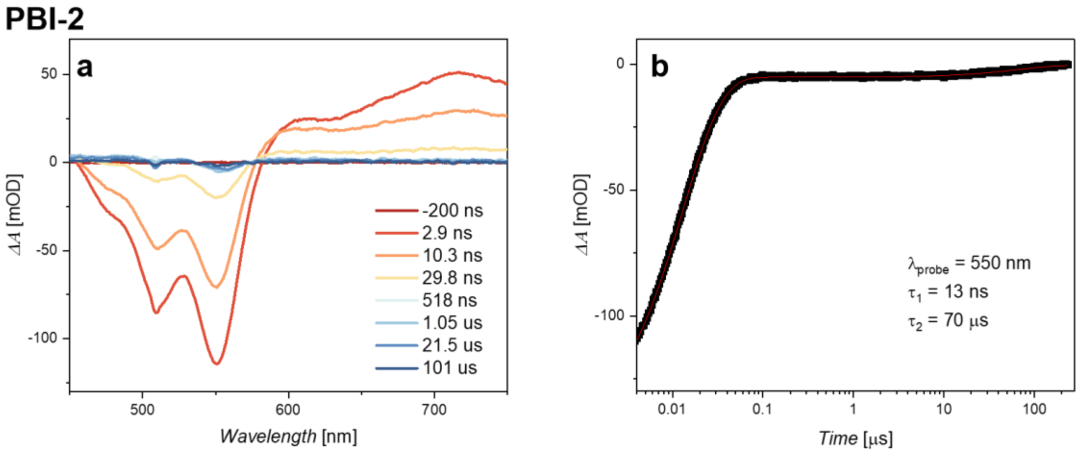

Supplementary Figure 35. Optical characterization by time-resolved spectroscopy. a ns-Transient absorption results of **PBI-2** in Tol using an excitation pump of 520 nm. **b** Temporal traces of **PBI-2** in Tol at 550 nm. The decay lines were fitted by a biexponential fit.

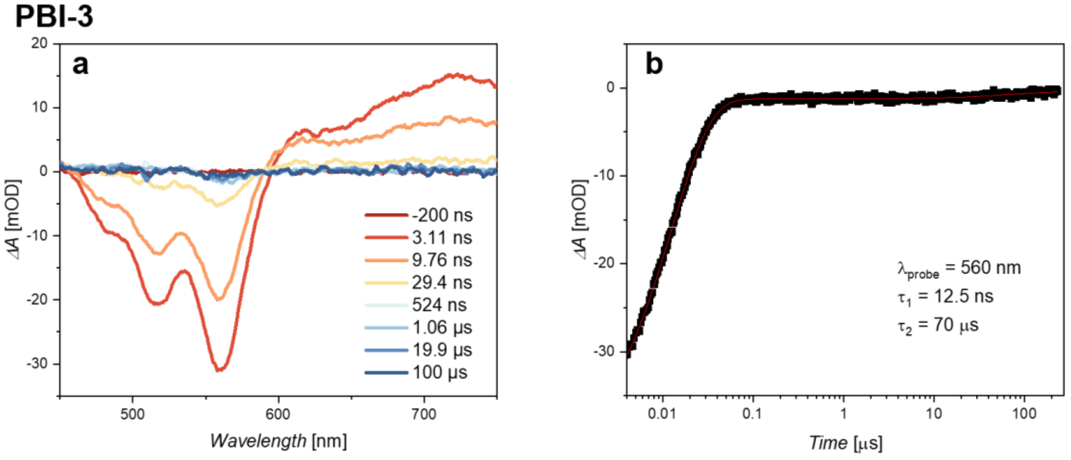

Supplementary Figure 36. Optical characterization by time-resolved spectroscopy. a ns-Transient absorption results of **PBI-3** in Tol using an excitation pump of 520 nm. **b** Temporal traces of **PBI-3** in Tol at 560 nm. The decay lines were fitted by a biexponential fit.

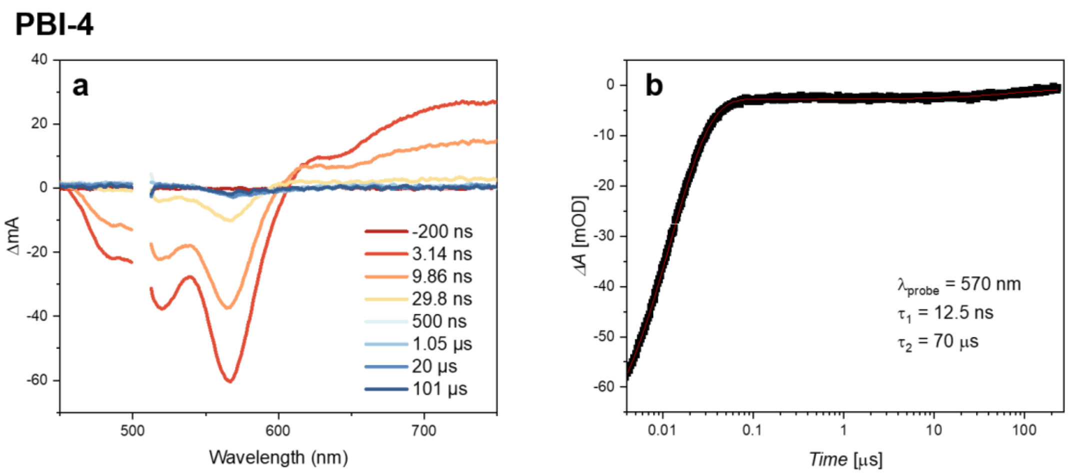

Supplementary Figure 37. Optical characterization by time-resolved spectroscopy. a ns-Transient absorption results of **PBI-4** in Tol using an excitation pump of 520 nm. **b** Temporal traces of **PBI-4** in Tol at 570 nm. The decay lines were fitted by a biexponential fit.

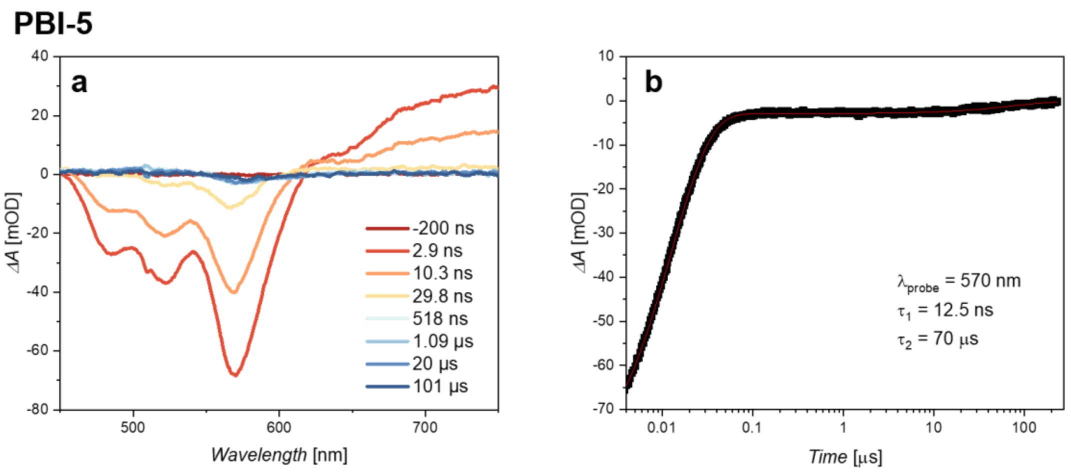

Supplementary Figure 38. Optical characterization by time-resolved spectroscopy. a ns-Transient absorption results of **PBI-5** in Tol using an excitation pump of 520 nm. **b** Temporal traces of **PBI-5** in Tol at 570 nm. The decay lines were fitted by a biexponential fit.

PBI-6

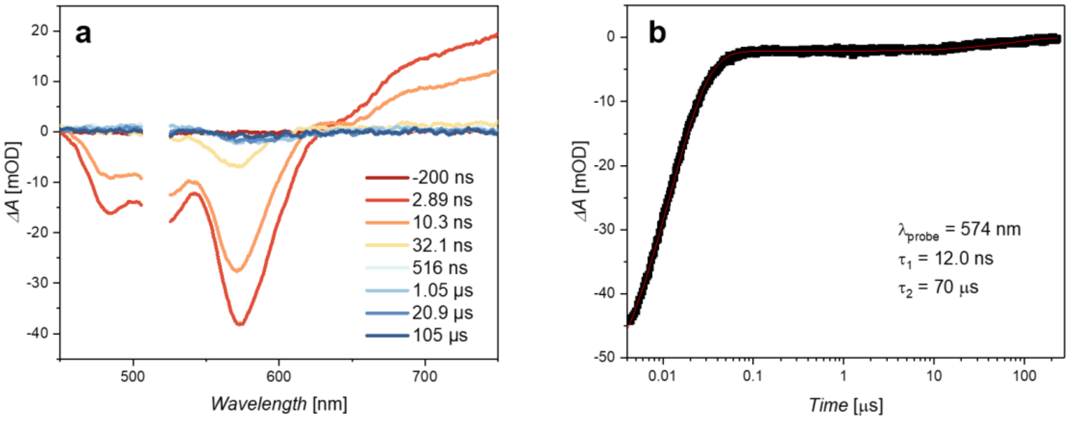

Supplementary Figure 39. Optical characterization by time-resolved spectroscopy. a ns-Transient absorption results of **PBI-6** in Tol using an excitation pump of 520 nm. **b** Temporal traces of **PBI-6** in Tol at 574 nm. The decay lines were fitted by a biexponential fit.

PBI-10

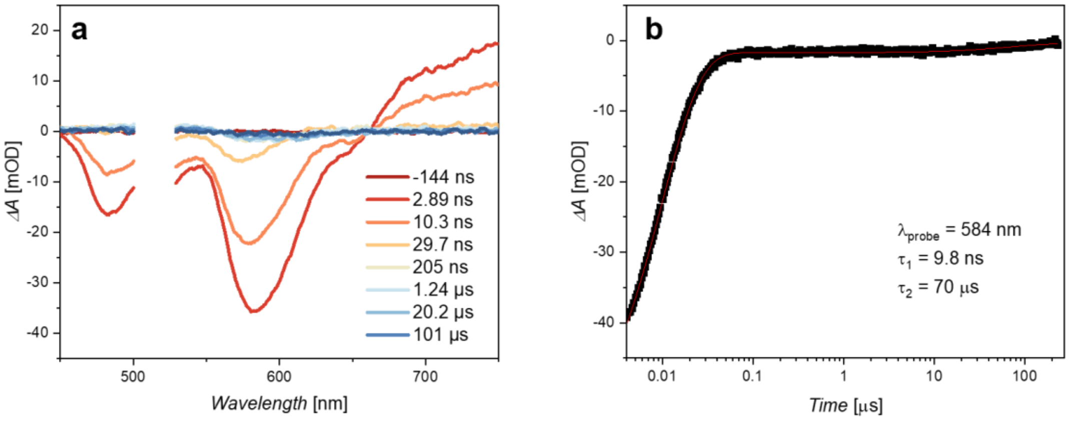

Supplementary Figure 40. Optical characterization by time-resolved spectroscopy. a ns-Transient absorption results of **PBI-10** in Tol using an excitation pump of 520 nm. **b** Temporal traces of **PBI-10** in Tol at 584 nm. The decay lines were fitted by a biexponential fit.

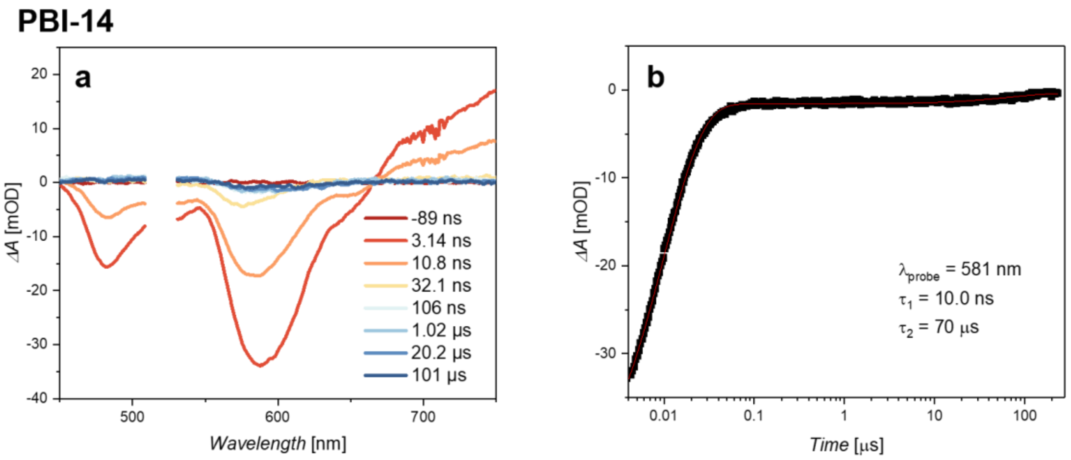

Supplementary Figure 41. Optical characterization by time-resolved spectroscopy. a ns-Transient absorption results of **PBI-14** in Tol using an excitation pump of 520 nm. **b** Temporal traces of **PBI-14** in Tol at 581 nm. The decay lines were fitted by a biexponential fit.

Relative Free Triplet Yield Determination

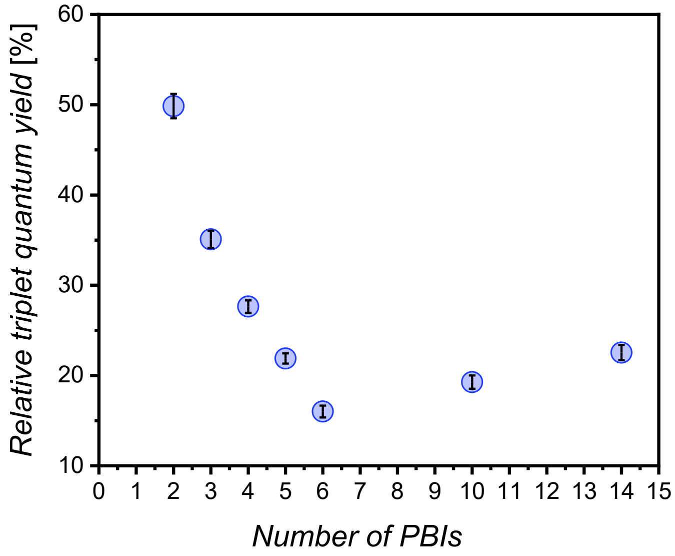

Supplementary Figure 42. Relative free triplet yield determination. Relative free triplet quantum yields for **PBI-N** (N = 2 to 14) obtained by singlet oxygen generation method. The error bar is one ± standard deviation. Error bars represent the uncertainty estimated from line fitting shown in Supplementary Figure 44 and do not reflect statistical variation across independent measurements.

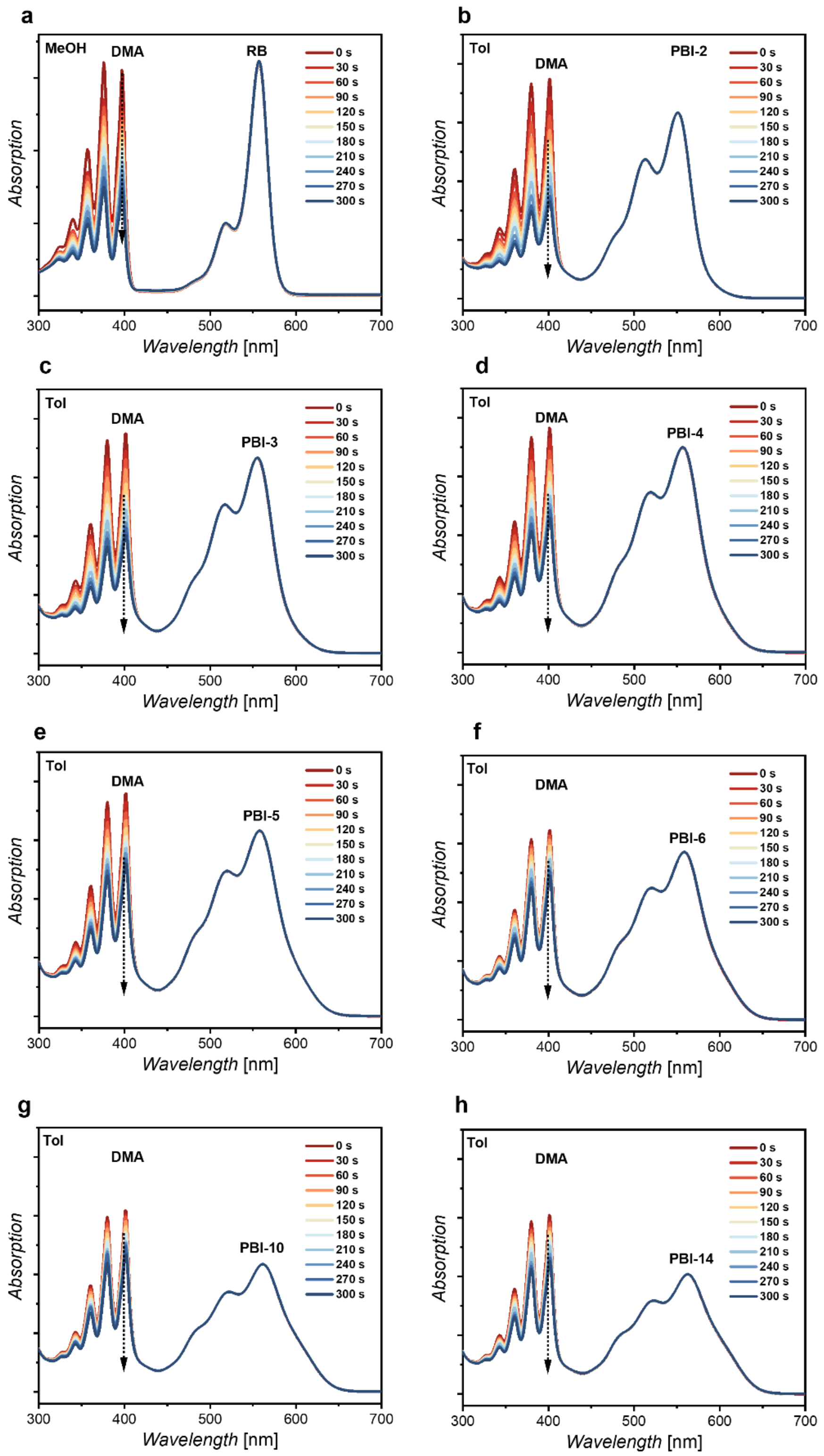

Supplementary Figure 43. Relative free triplet yield determination. Time-dependent absorption spectra for DMA as singlet oxygen probe in air-saturated toluene (MeOH for RB) containing **a** RB and **b-h PBI-N** (N = 2 to 14) as a photo-sensitizer with photoexcitation at 520 nm. The decrease in DMA absorbance is due to oxidized decomposition by singlet oxygen. We note that the absorption spectra for RB and PBI-N show no change during light irradiation.[32,33]

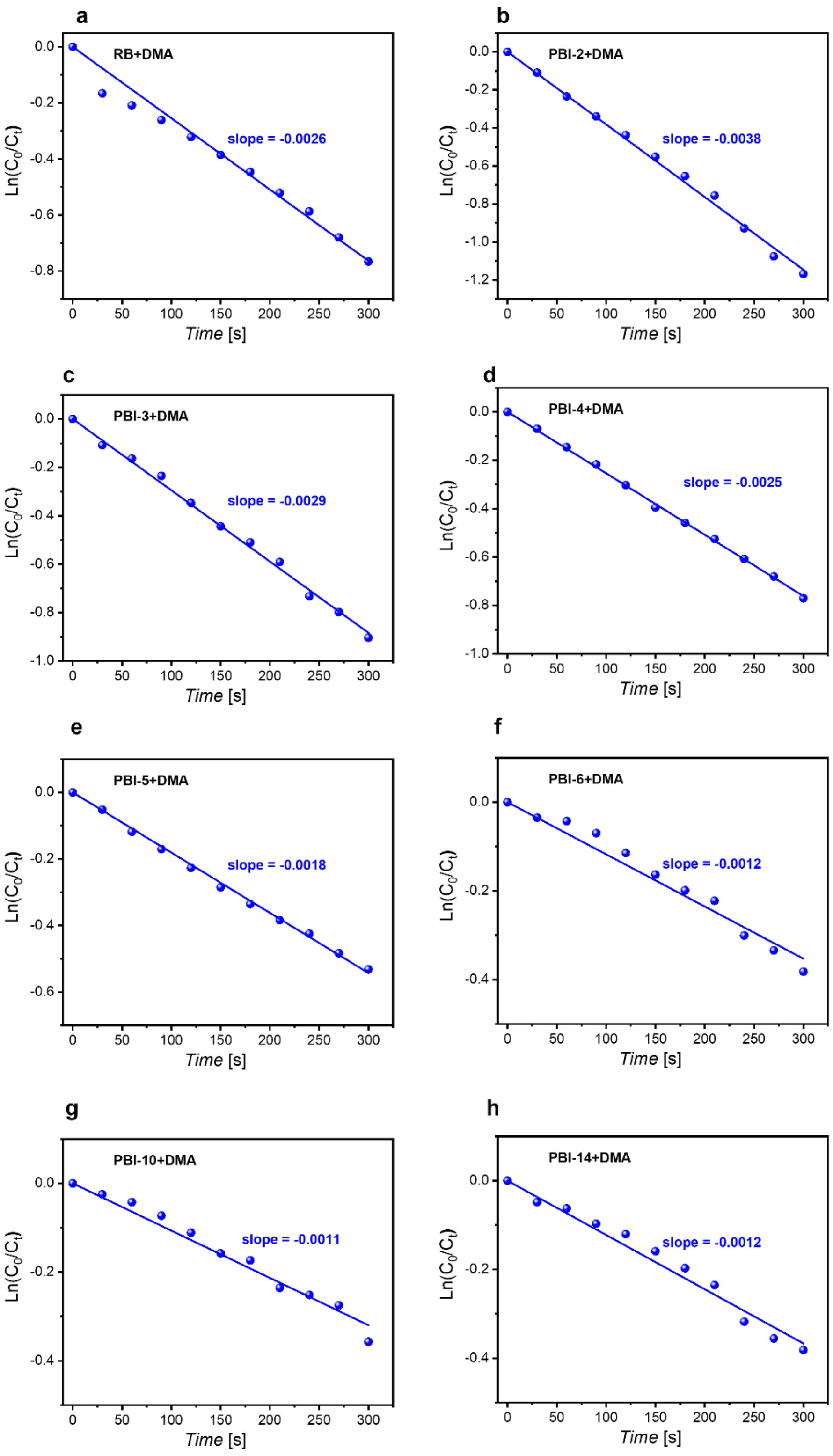

Supplementary Figure 44. Relative free triplet yield determination. The decay of DMA containing **a** RB and **b-h PBI-N** (N = 2 to 14) obtained from Supplementary Figure 43. The temporal traces were fitted by linear function.

fs-Time-resolved Fluorescence Spectroscopy

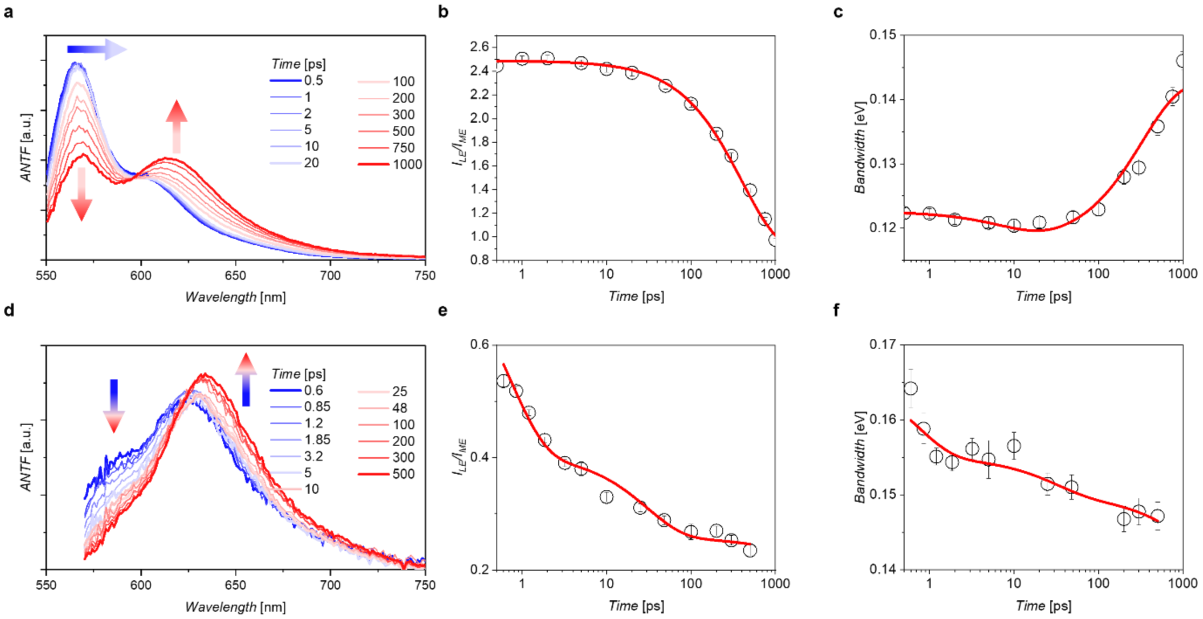

Supplementary Figure 45. fs-Transient fluorescence (TF) results. a-c, fs-TF results of **PBI-2** in Tol. The area-normalized transient fluorescence (ANTF) spectra (**a**). The relative peak ratio between LE and ME bands (**b**) and bandwidth (**c**) as a function of time. The relative peak ratio and bandwidth were estimated by fitting Gaussian functions. The error bar indicates ± one standard. **d-f**, fs-TF results of **PBI-5** in Tol. The area-normalized transient fluorescence (ANTF) spectra (**d**). The relative peak ratio between LE and ME bands (**e**) and bandwidth (**f**) as a function of time. The relative peak ratio and bandwidth were estimated by fitting Gaussian functions. The error bar indicates ± one standard deviation. Error bars indicate the uncertainty estimated from Gaussian fitting and do not represent statistical variation.

The higher-energy peak at 575 nm and the lower-energy peak at 625 nm exhibit clearly different lifetimes (~5 ns vs. ~8 ns; see Fig. 3b in the main text), confirming that they arise from distinct electronic states rather than vibronic replicas of a single state.

Although the steady-state emission spectrum superficially resembles a vibronic progression, the fs-TF data clearly distinguish the two emissive features. For **PBI-2**, the fs-TF results reveal a small redshift (~9 ps) associated with structural relaxation, followed by a slower intensity redistribution (~330 ps) between the LE (575 nm) and ME (625 nm) peaks, confirming the evolution into the multiexciton (ME) state (Supplementary Figure 45).

In contrast, in the elongated stack (**PBI-5**), the fs-TF spectra evolve on both sub-picosecond and ~10 ps timescales, accompanied by a progressive narrowing of the emission bandwidth. This behavior is markedly different from that of excimer emission, which typically dominates in H-type aggregates as studied in earlier work.[34,35] Instead, it is consistent with efficient LE–CT mixing at the Franck–Condon geometry, followed by rapid generation of the ME state.

Due to experimental challenges including stability issues observed for stacks larger than the hexamer, reliable data could so far only be obtained up to the pentamer.

ns-Time-resolved Fluorescence Spectroscopy

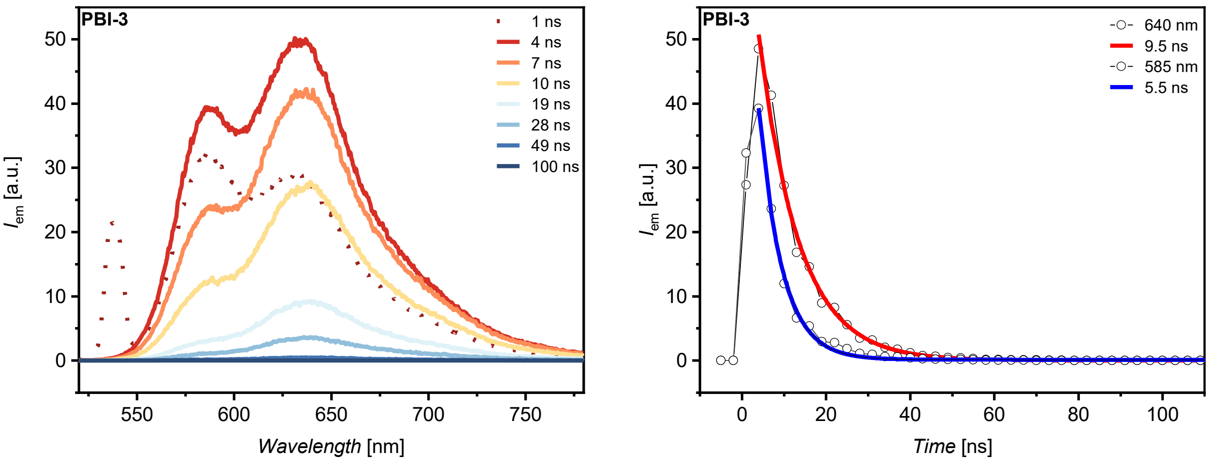

Supplementary Figure 46. Optical characterization by time-resolved spectroscopy. The ns-TF fluorescence spectra (left) and temporal traces (right) of **PBI-3** in Tol. The decay lines were fitted by a single exponential fit. The spectra at 1 ns are within instrumental response function (~ 3 ns).

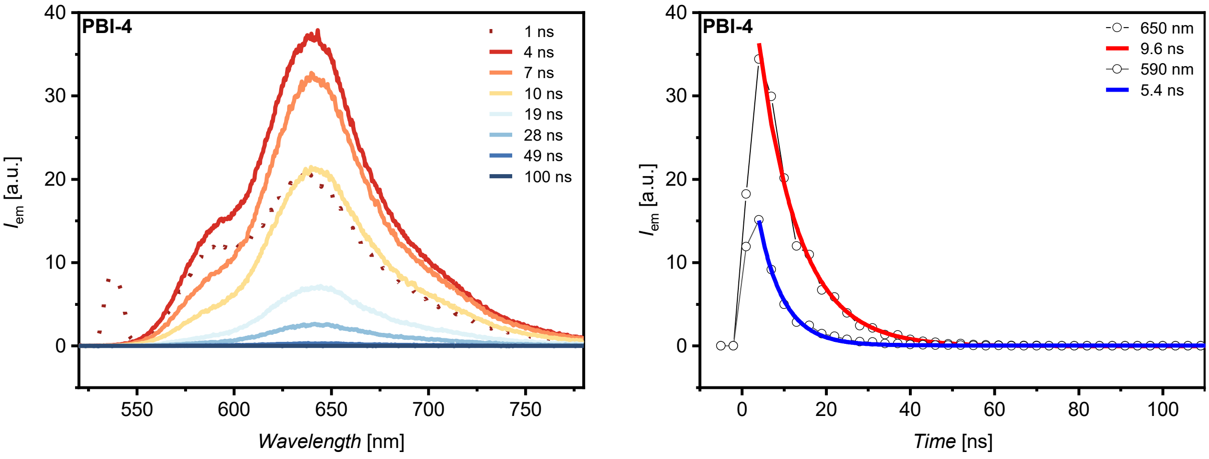

Supplementary Figure 47. Optical characterization by time-resolved spectroscopy. The ns-TF fluorescence spectra (left) and temporal traces (right) of **PBI-4** in Tol. The decay lines were fitted by a single exponential fit. The spectra at 1 ns are within instrumental response function (~ 3 ns).

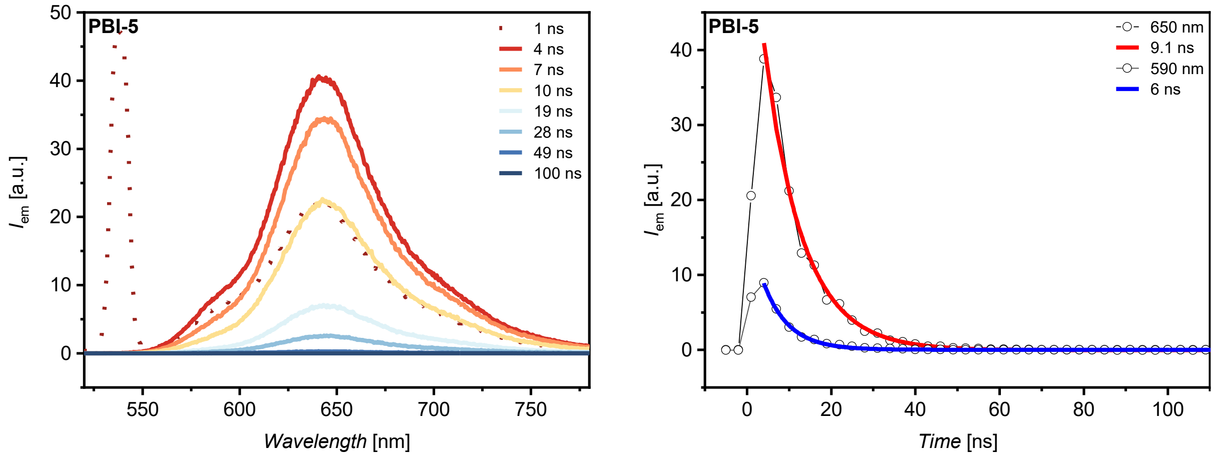

Supplementary Figure 48. Optical characterization by time-resolved spectroscopy. The ns-TF fluorescence spectra (left) and temporal traces (right) of **PBI-5** in Tol. The decay lines were fitted by a single exponential fit. The spectra at 1 ns are within instrumental response function (~ 3 ns).

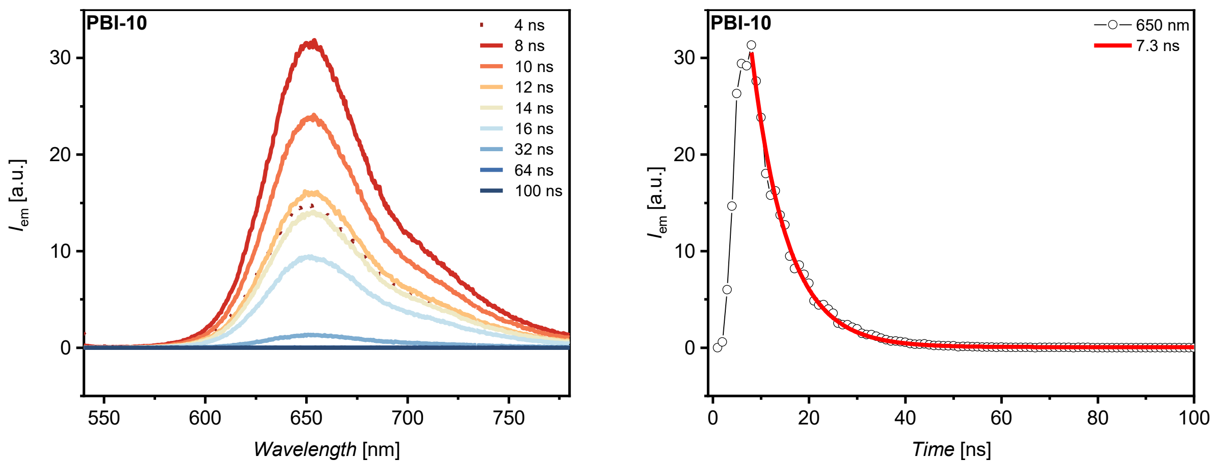

Supplementary Figure 49. Optical characterization by time-resolved spectroscopy. The ns-TF fluorescence spectra (left) and temporal traces (right) of **PBI-10** in Tol. The decay lines were fitted by a single exponential fit. The spectra at 1 ns are within instrumental response function (~ 3 ns).

7. NMR Spectroscopy

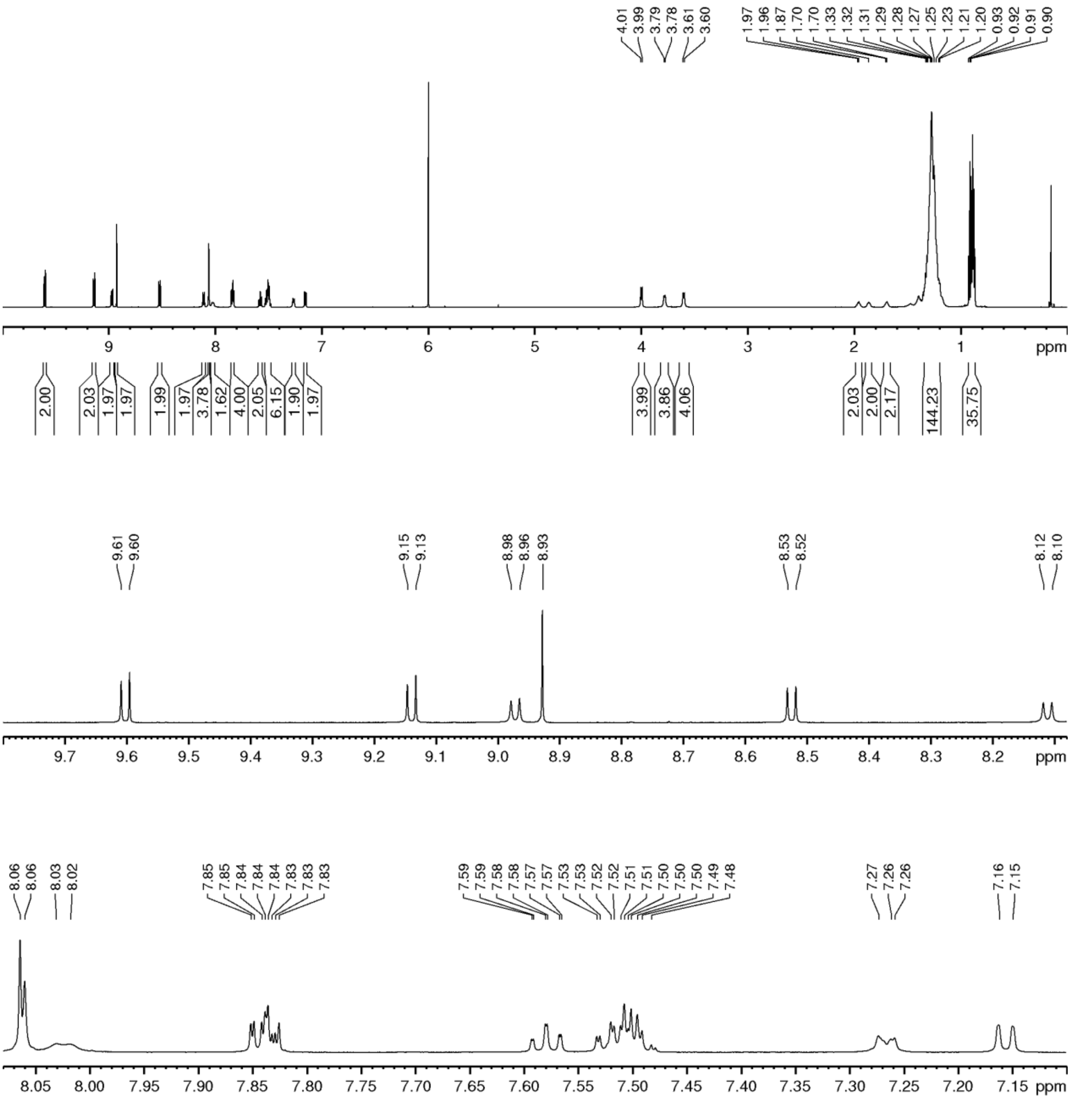

Supplementary Figure 50. ^{1}H NMR spectrum (600 MHz, 384 K, TCE-d_2) of **PBI-Center3** (for structures, see Supplementary Figure 1).

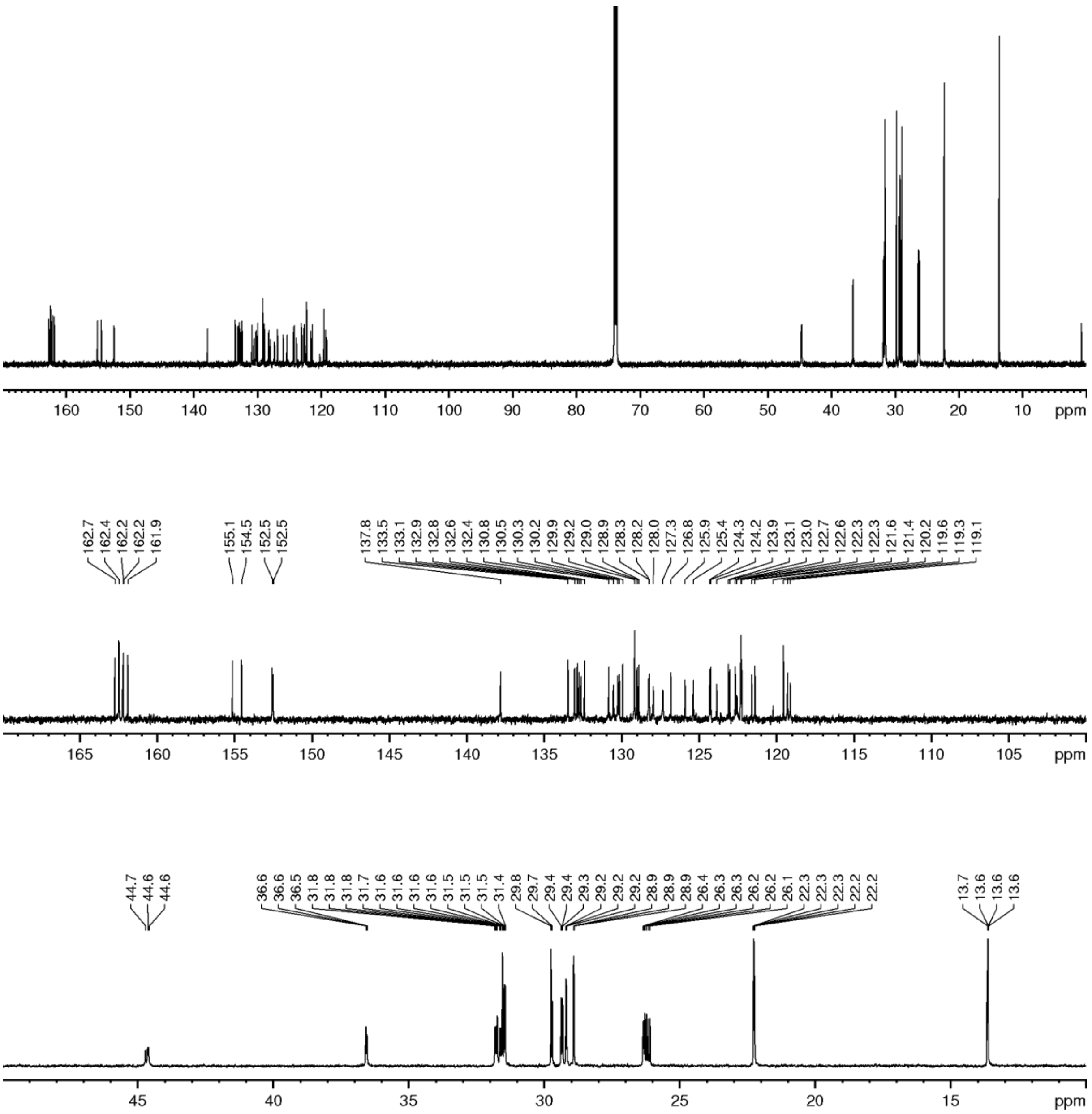

Supplementary Figure 51. ^{13}C NMR spectrum (151 MHz, 384 K, TCE-d_2) of **PBI-Center3** (for structures, see Supplementary Figure 1).

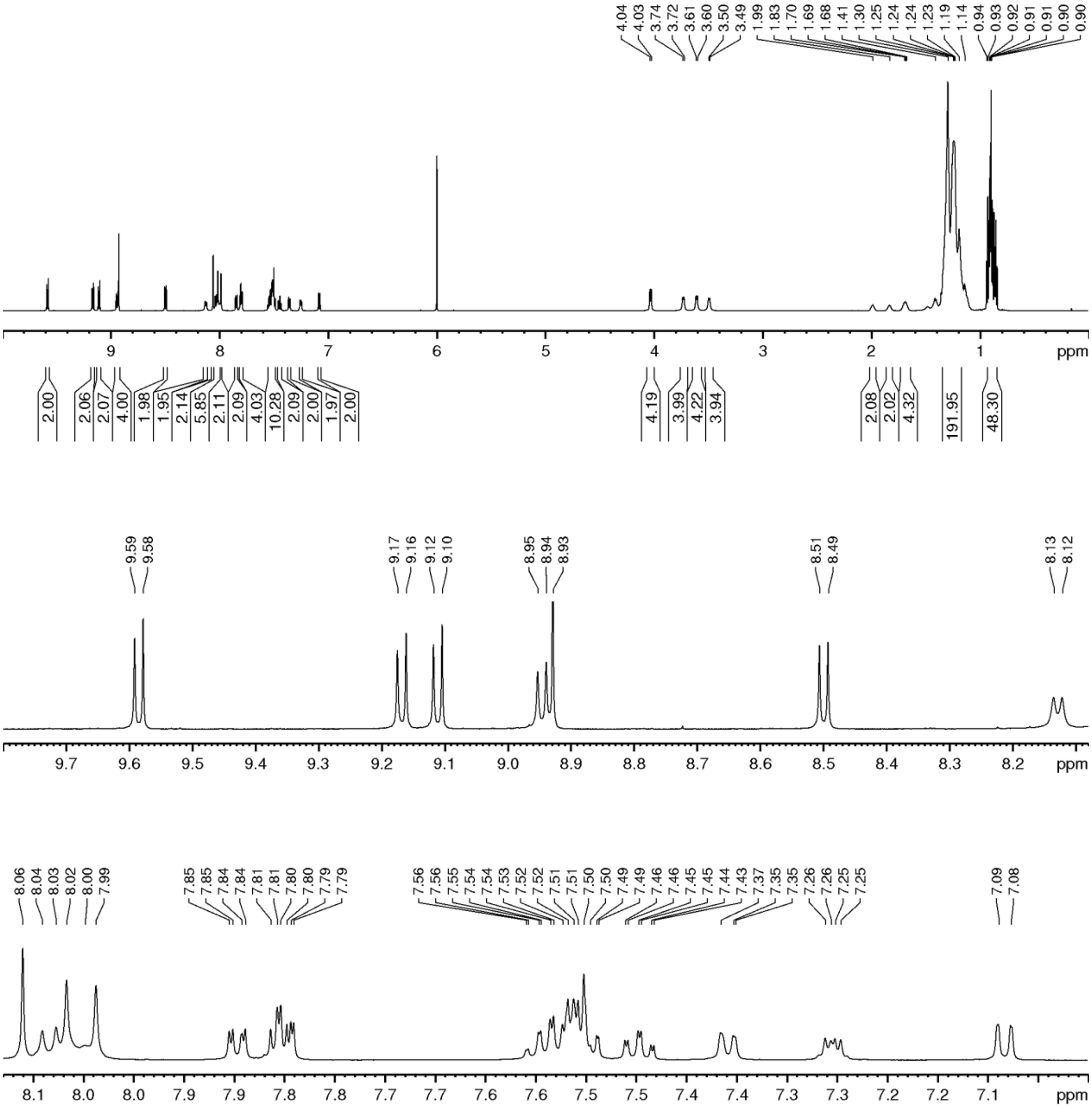

Supplementary Figure 52. ^{1}H NMR spectrum (600 MHz, 384 K, TCE-d_2) of **PBI-Center4** (for structures, see Supplementary Figure 1).

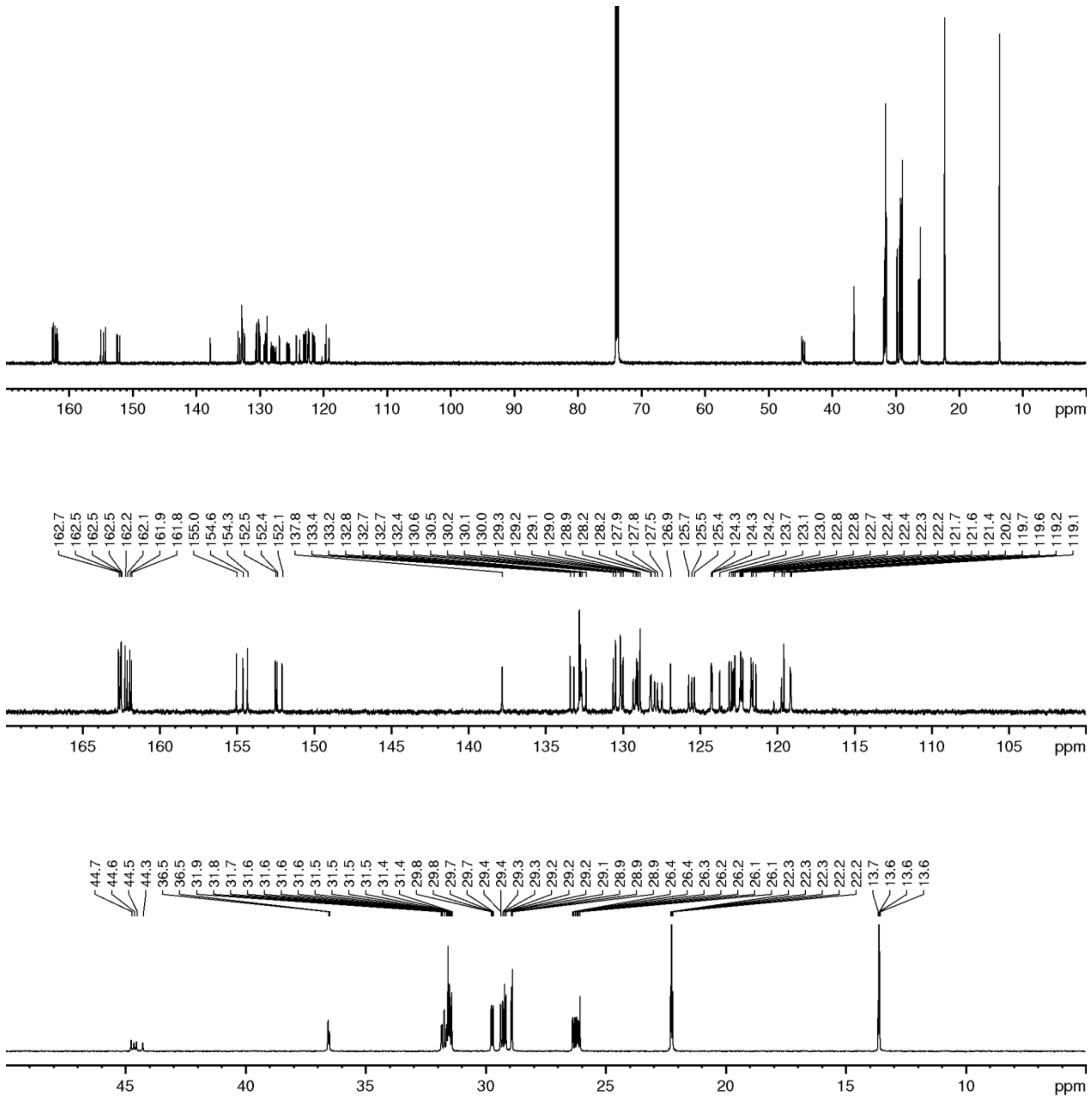

Supplementary Figure 53. ^{13}C NMR spectrum (151 MHz, 384 K, TCE-d_2) of **PBI-Center4** (for structures, see Supplementary Figure 1).

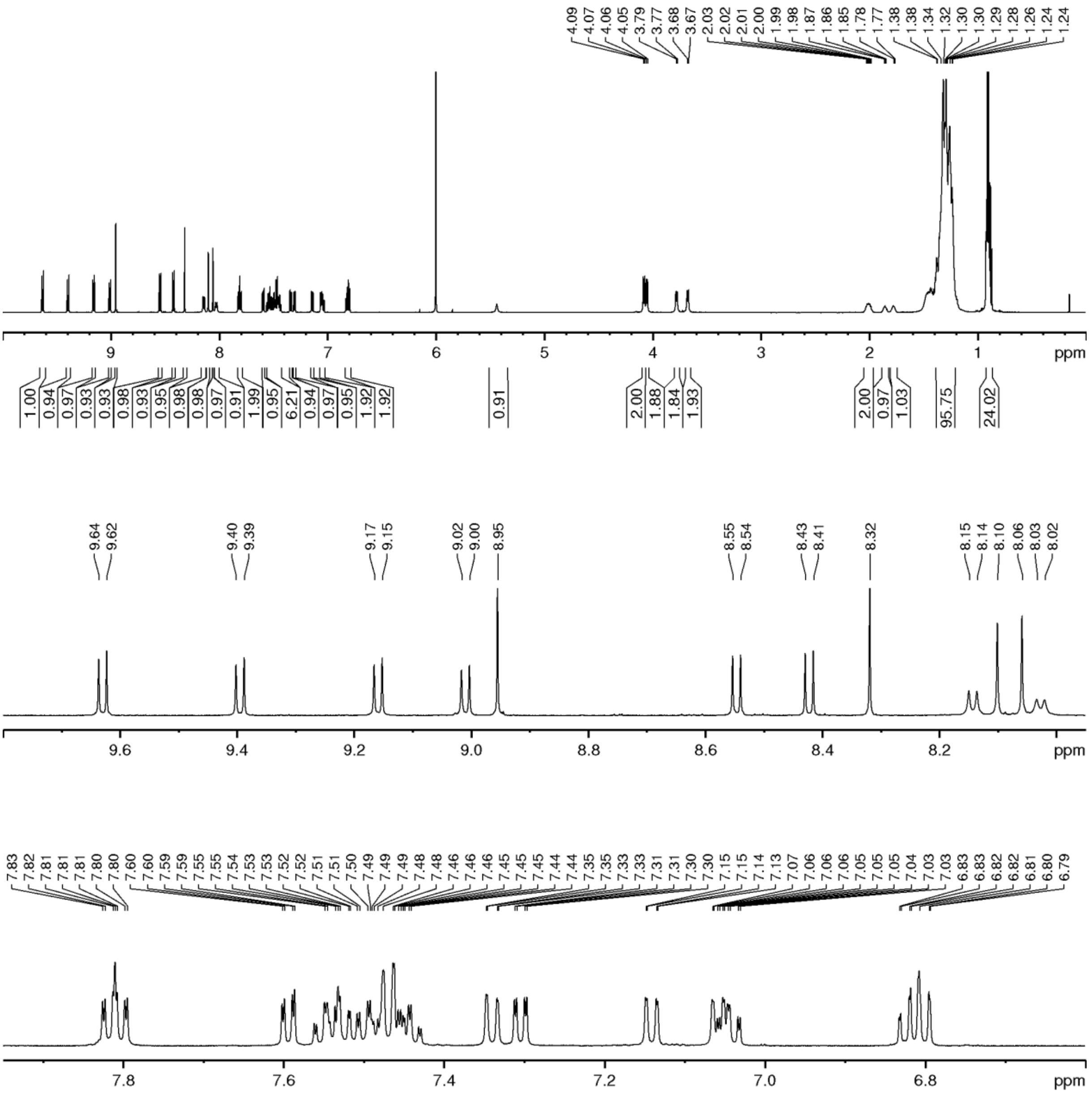

Supplementary Figure 54. ^{1}H NMR spectrum (600 MHz, 384 K, TCE-d_2) of the 2,2´-biphenol-bromo-substituted dimer **2** (for structures, see Supplementary Figure 1).

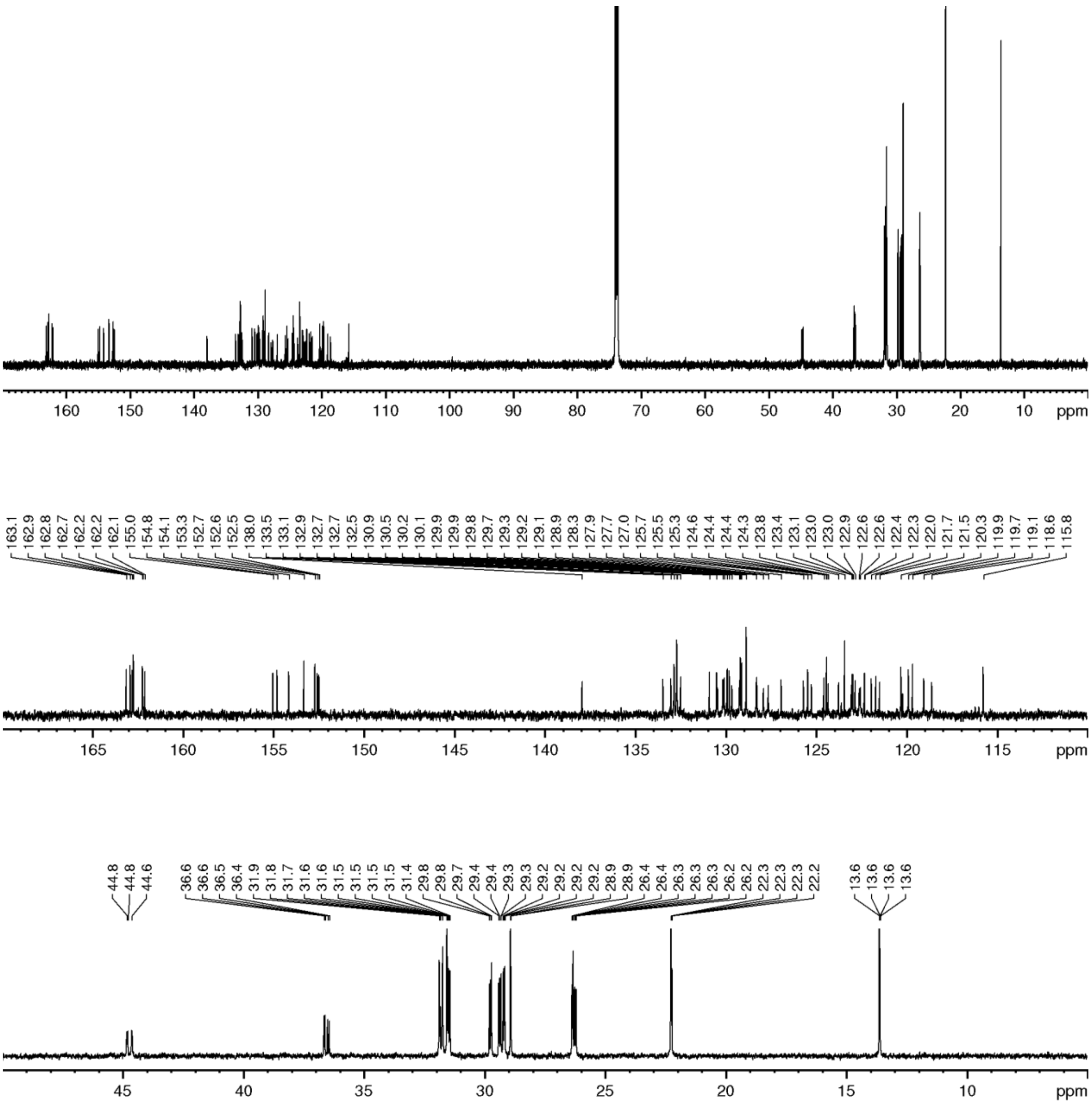

Supplementary Figure 55. ^{13}C NMR spectrum (151 MHz, 384 K, TCE-d_2) of the 2,2´-biphenol-bromo-substituted dimer **2** (for structures, see Supplementary Figure 1).

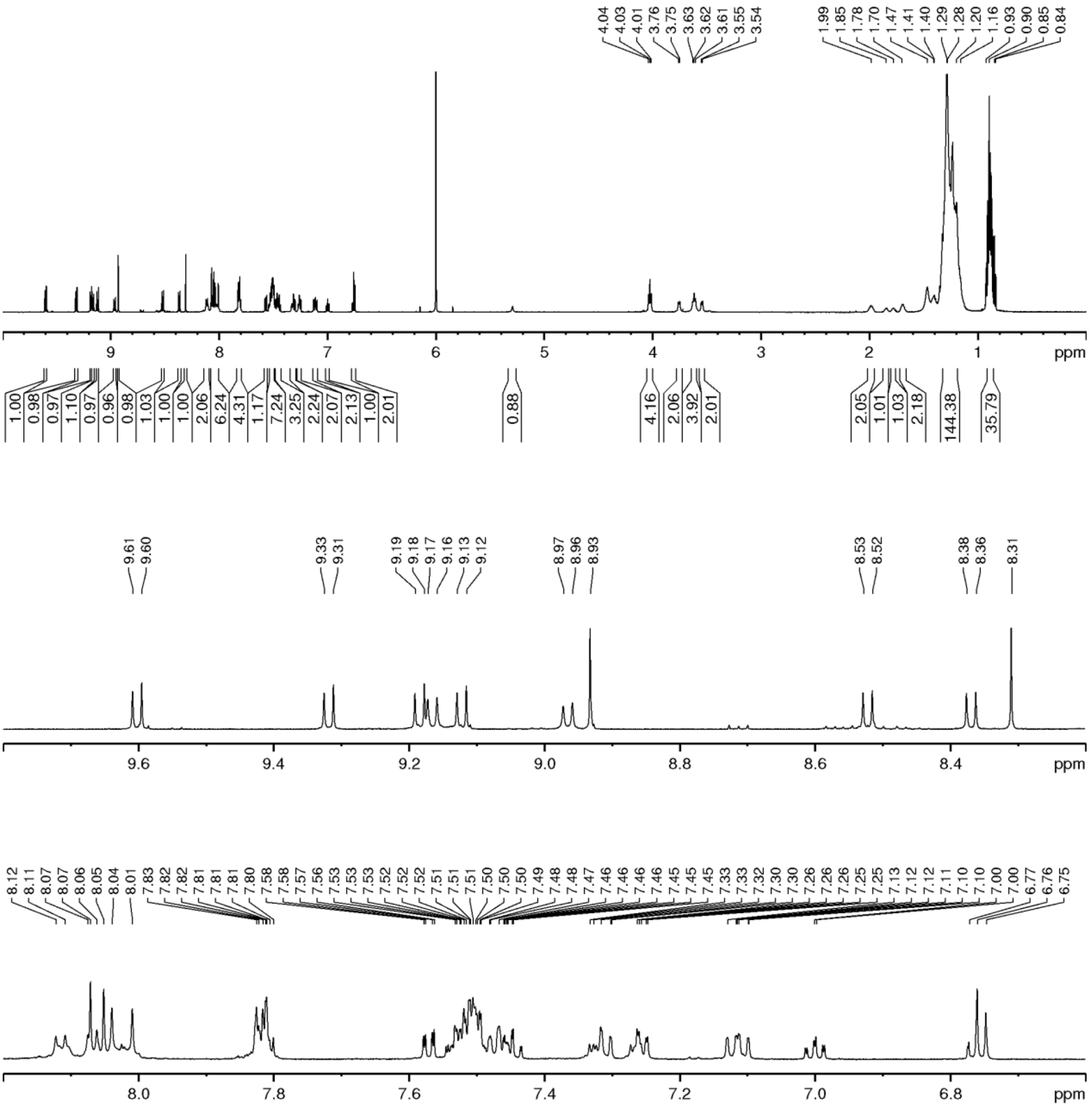

Supplementary Figure 56. ^{1}H NMR spectrum (600 MHz, 384 K, TCE-d_2) of the 2,2´-biphenol-bromo-substituted trimer **3** (for structures, see Supplementary Figure 1).

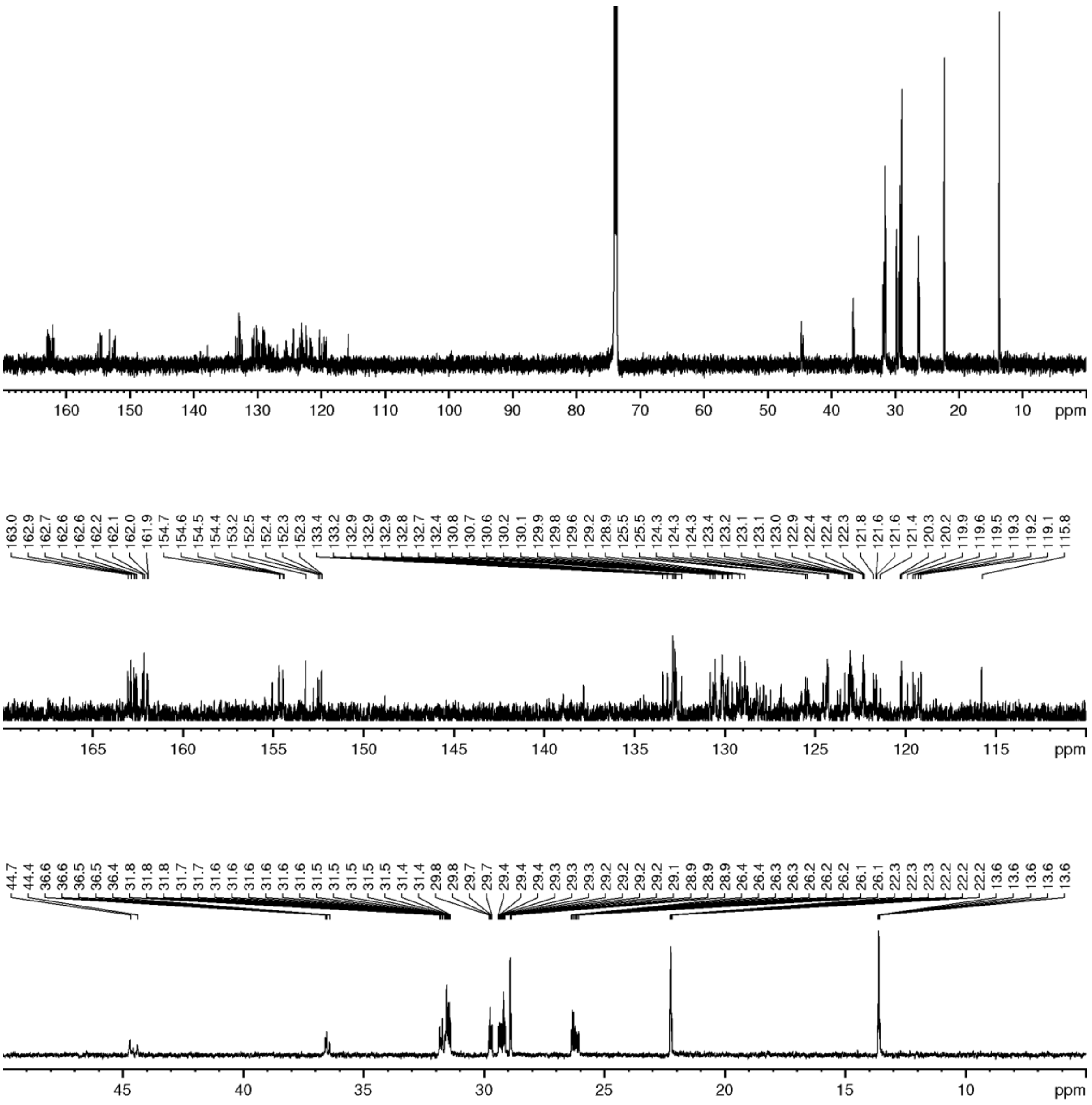

Supplementary Figure 57. ^{13}C NMR spectrum (151 MHz, 384 K, TCE-d_2) of the 2,2´-biphenol-bromo-substituted trimer **3** (for structures, see Supplementary Figure 1).

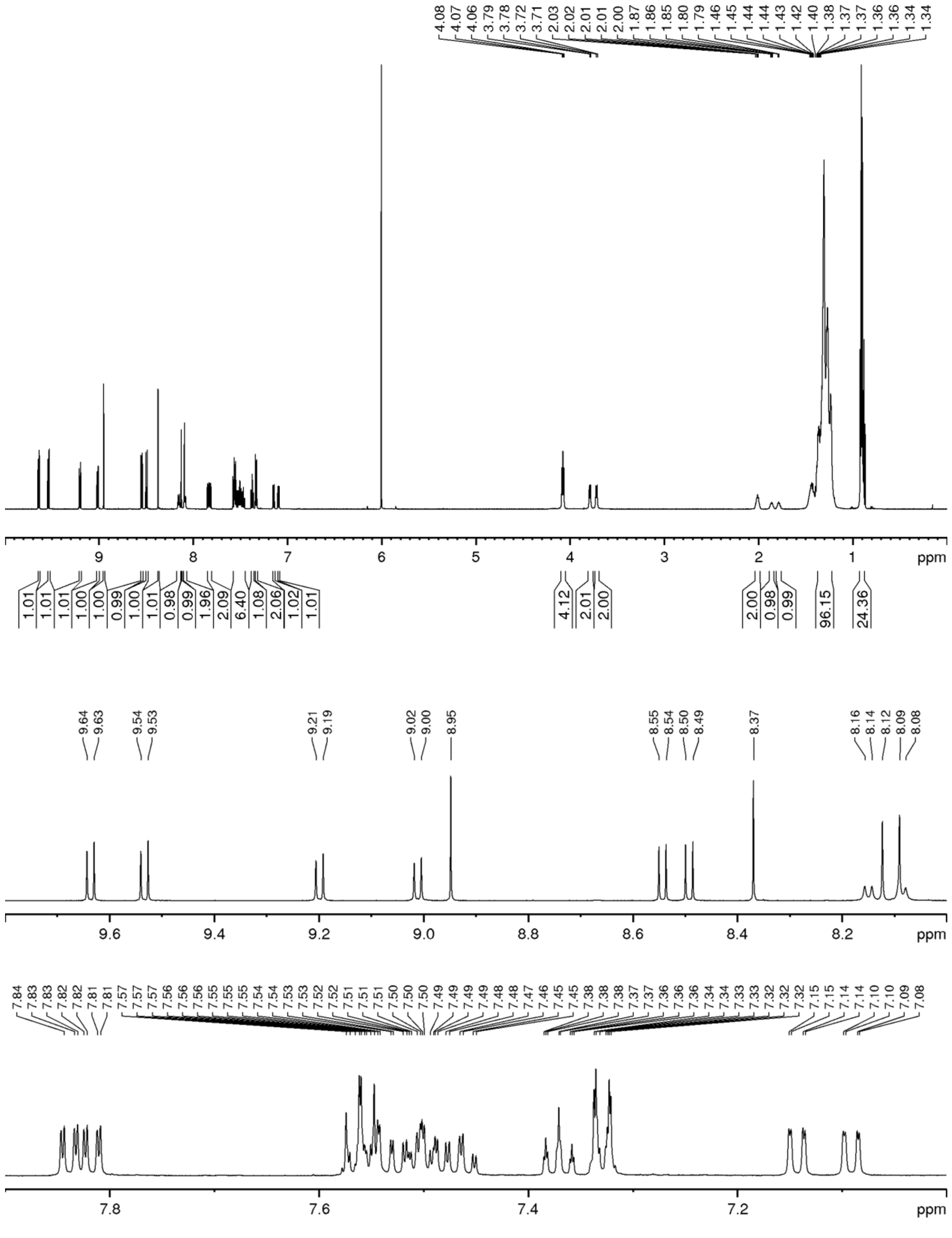

Supplementary Figure 58. ^{1}H NMR spectrum (600 MHz, 384 K, TCE-d_2) of the bromo-phenoxy-substituted dimer **5** (for structures, see Supplementary Figure 2).

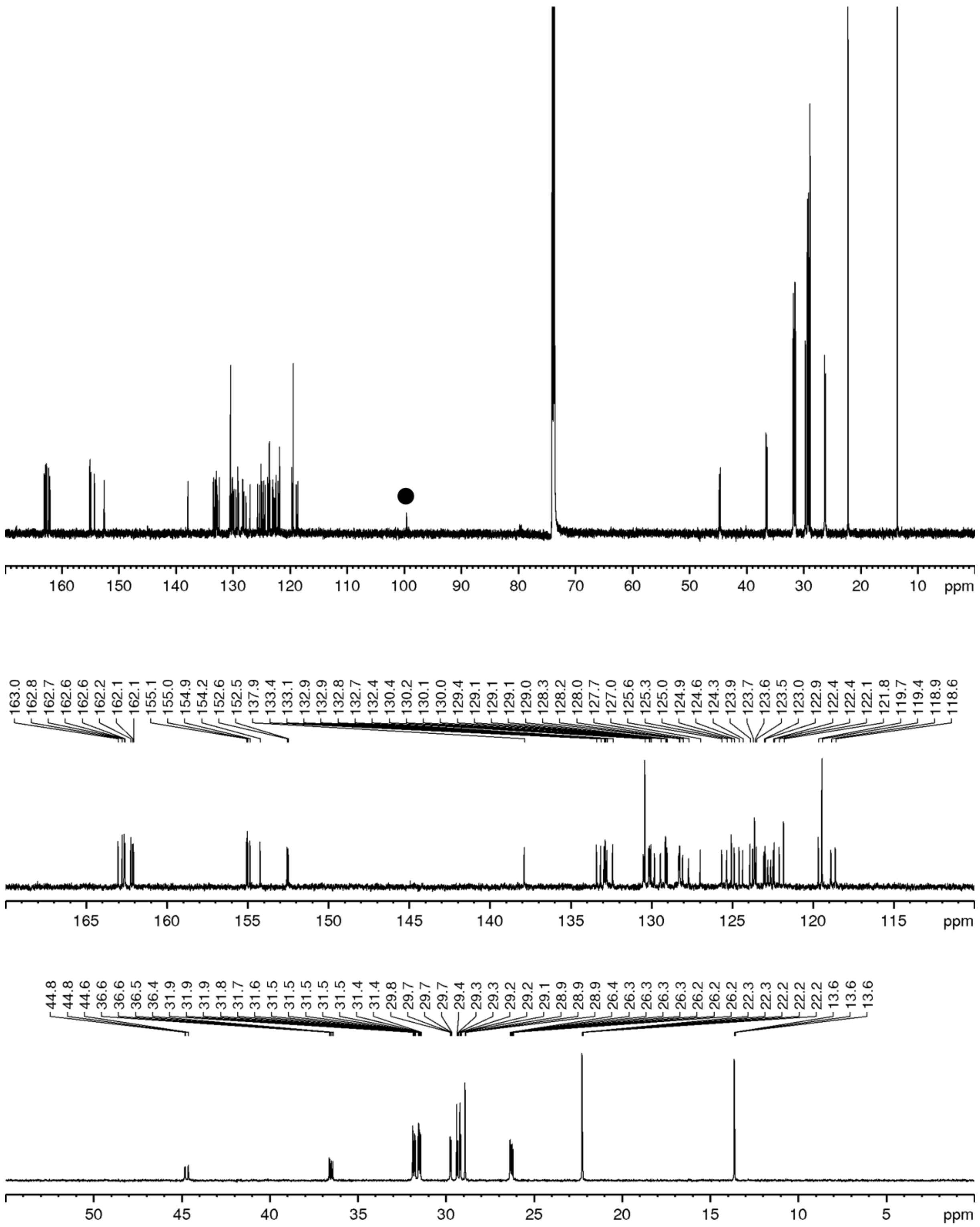

Supplementary Figure 59. ^{13}C NMR spectrum (151 MHz, 384 K, TCE-d_2) of the bromo-phenoxy-substituted dimer **5** (for structures, see Supplementary Figure 2). Residual solvent is marked as follows: tetrachloromethane (●) present in deuterated 1,1,2,2-tetrachloroethane.[36]

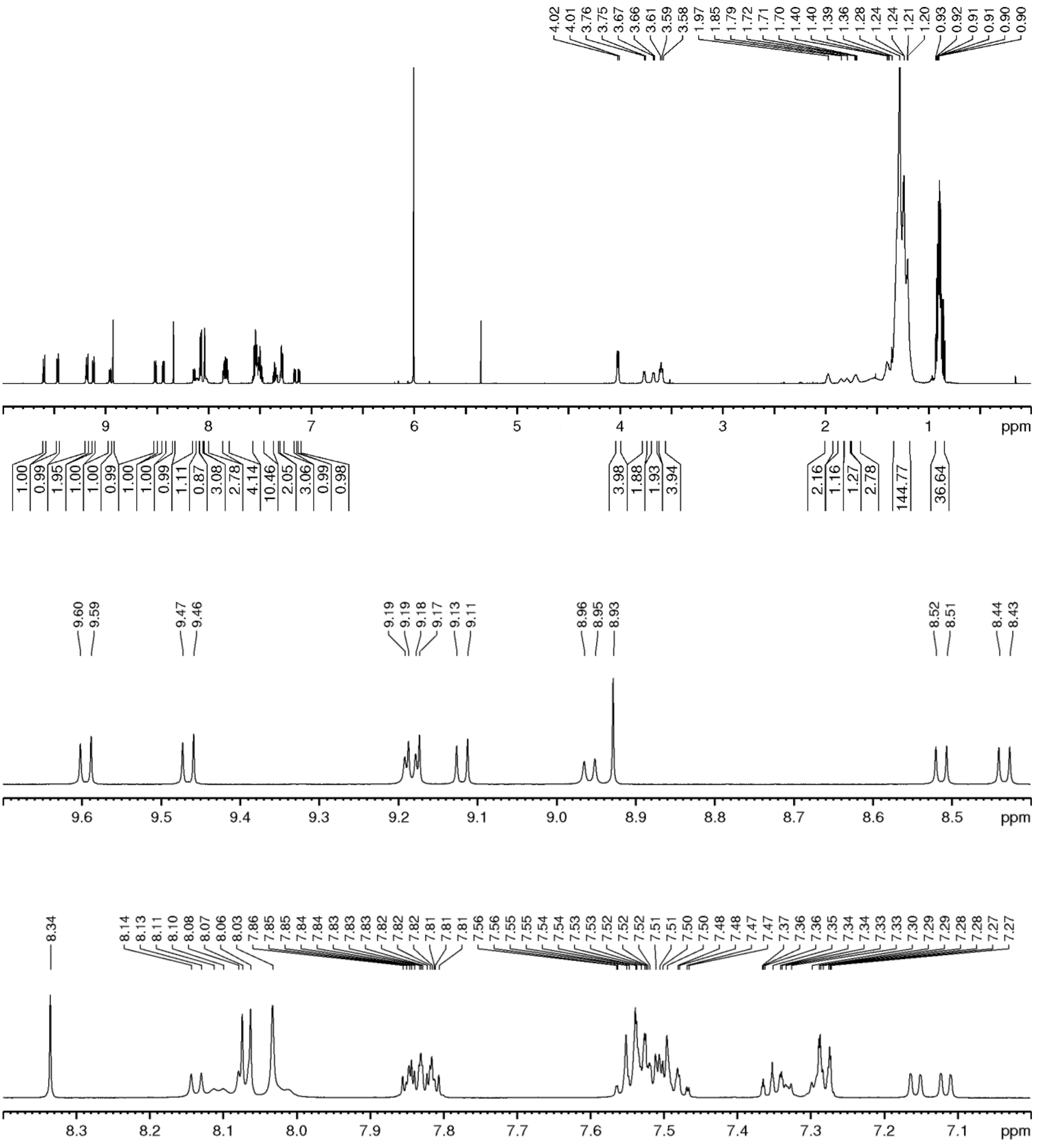

Supplementary Figure 60. ^{1}H NMR spectrum (600 MHz, 384 K, TCE-d_2) of the bromo-phenoxy-substituted trimer **6** (for structures, see Supplementary Figure 2).

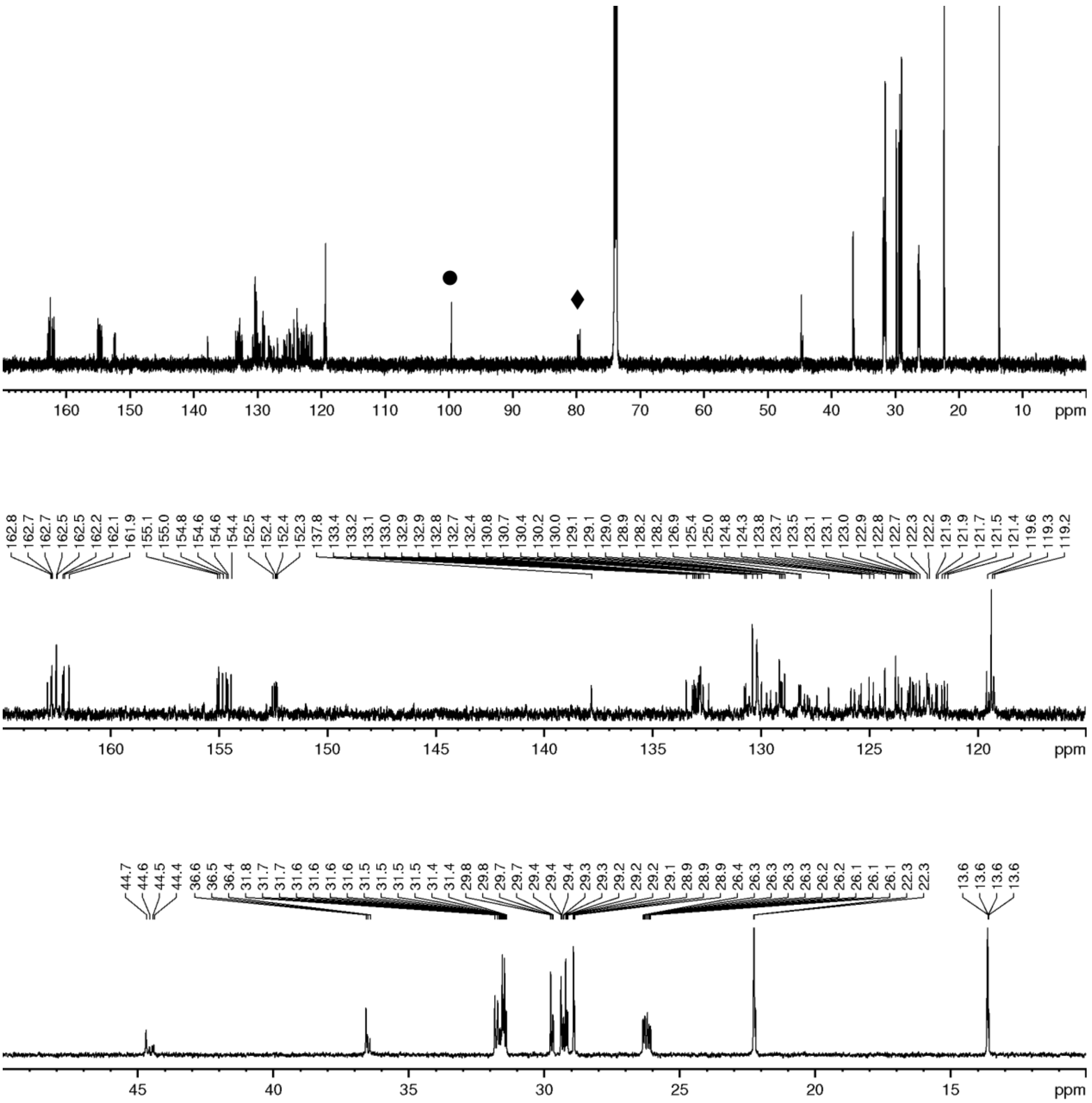

Supplementary Figure 61. ^{13}C NMR spectrum (151 MHz, 384 K, TCE-d_2) of the bromo-phenoxy-substituted trimer **6** (for structures, see Supplementary Figure 2). Residual solvent is marked as follows: chloroform (♦) and tetrachloromethane (●) present in deuterated 1,1,2,2-tetrachloroethane.[36]

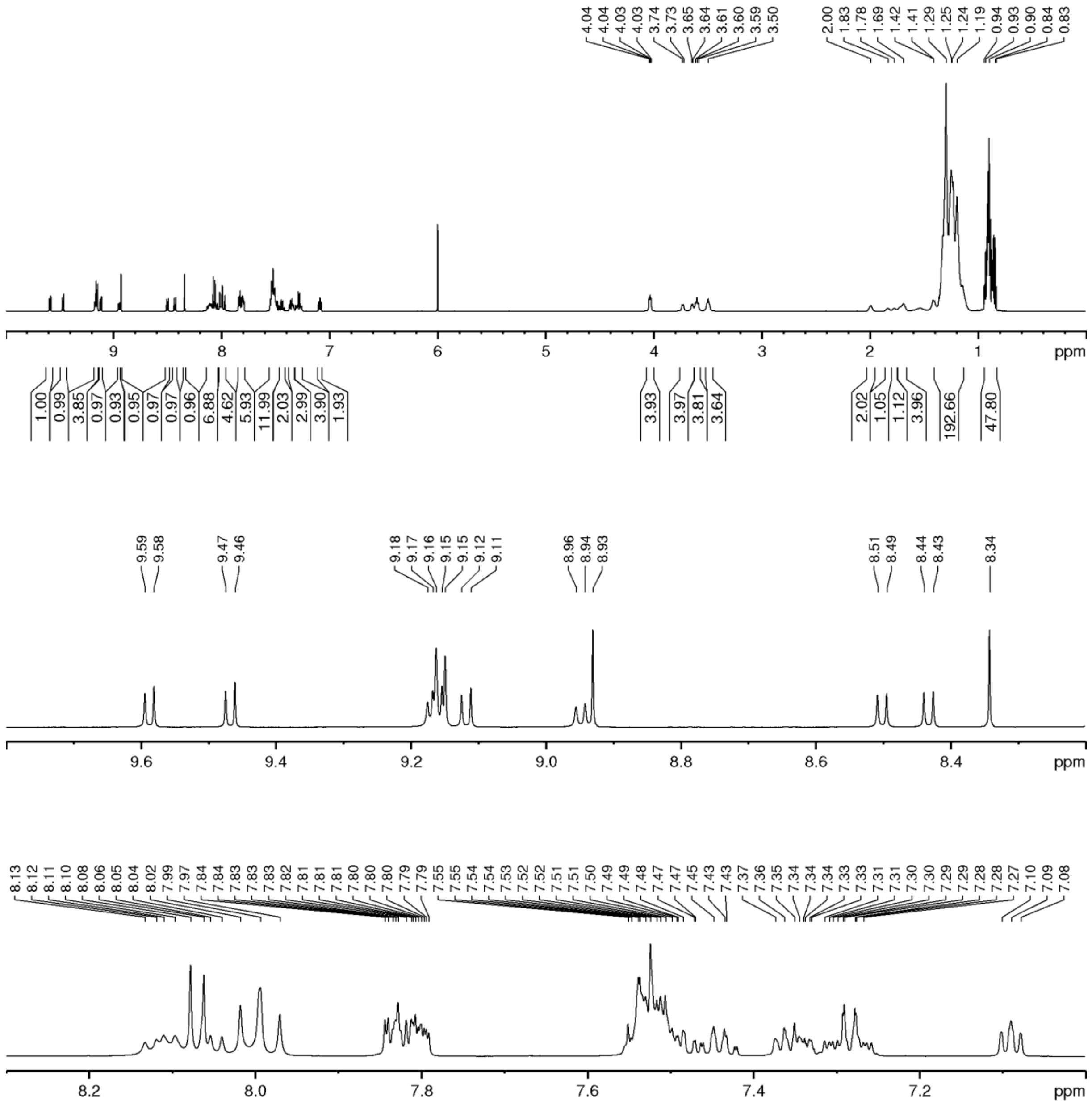

Supplementary Figure 62. ^{1}H NMR spectrum (600 MHz, 384 K, TCE-d_2) of the bromo-phenoxy-substituted tetramer **7** (for structures, see Supplementary Figure 2).

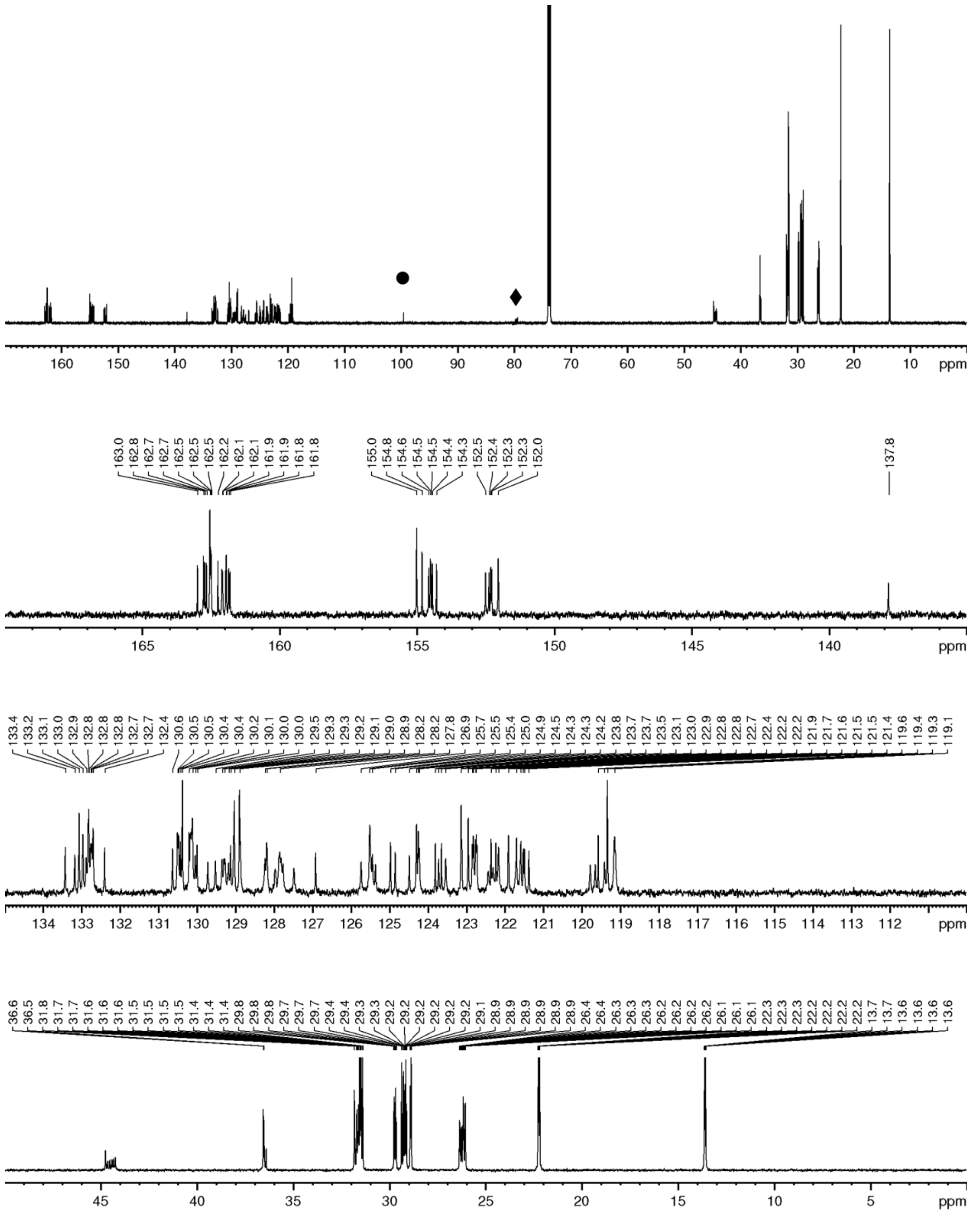

Supplementary Figure 63. ^{13}C NMR spectrum (151 MHz, 384 K, TCE-d_2) of the bromo-phenoxy-substituted tetramer **7** (for structures, see Supplementary Figure 2). Residual solvent is marked as follows: chloroform (♦) and tetrachloromethane (●) present in deuterated 1,1,2,2-tetrachloroethane.[36]

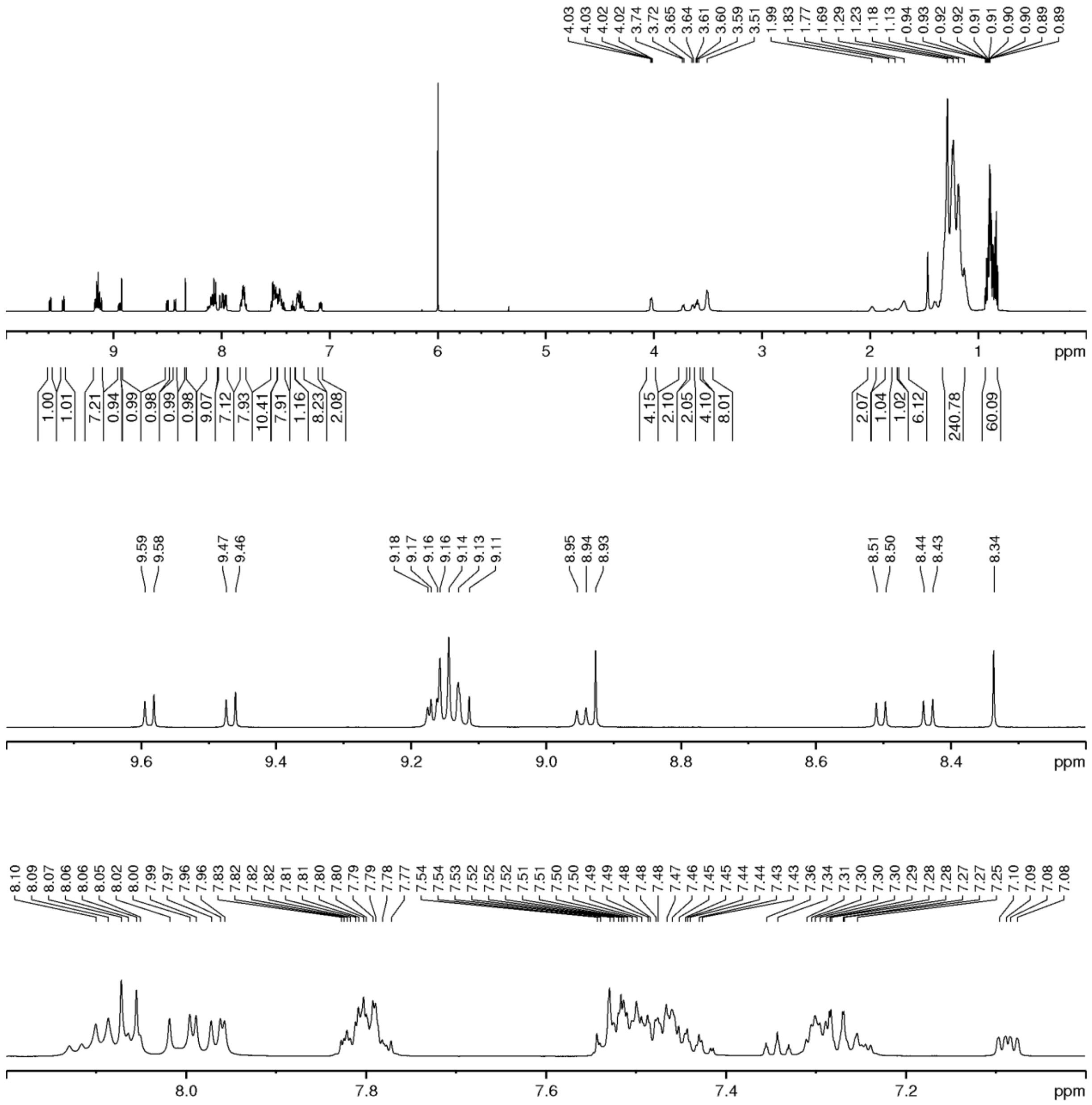

Supplementary Figure 64. ^{1}H NMR spectrum (600 MHz, 384 K, TCE-d_2) of the bromo-phenoxy-substituted pentamer **8** (for structures, see Supplementary Figure 2).

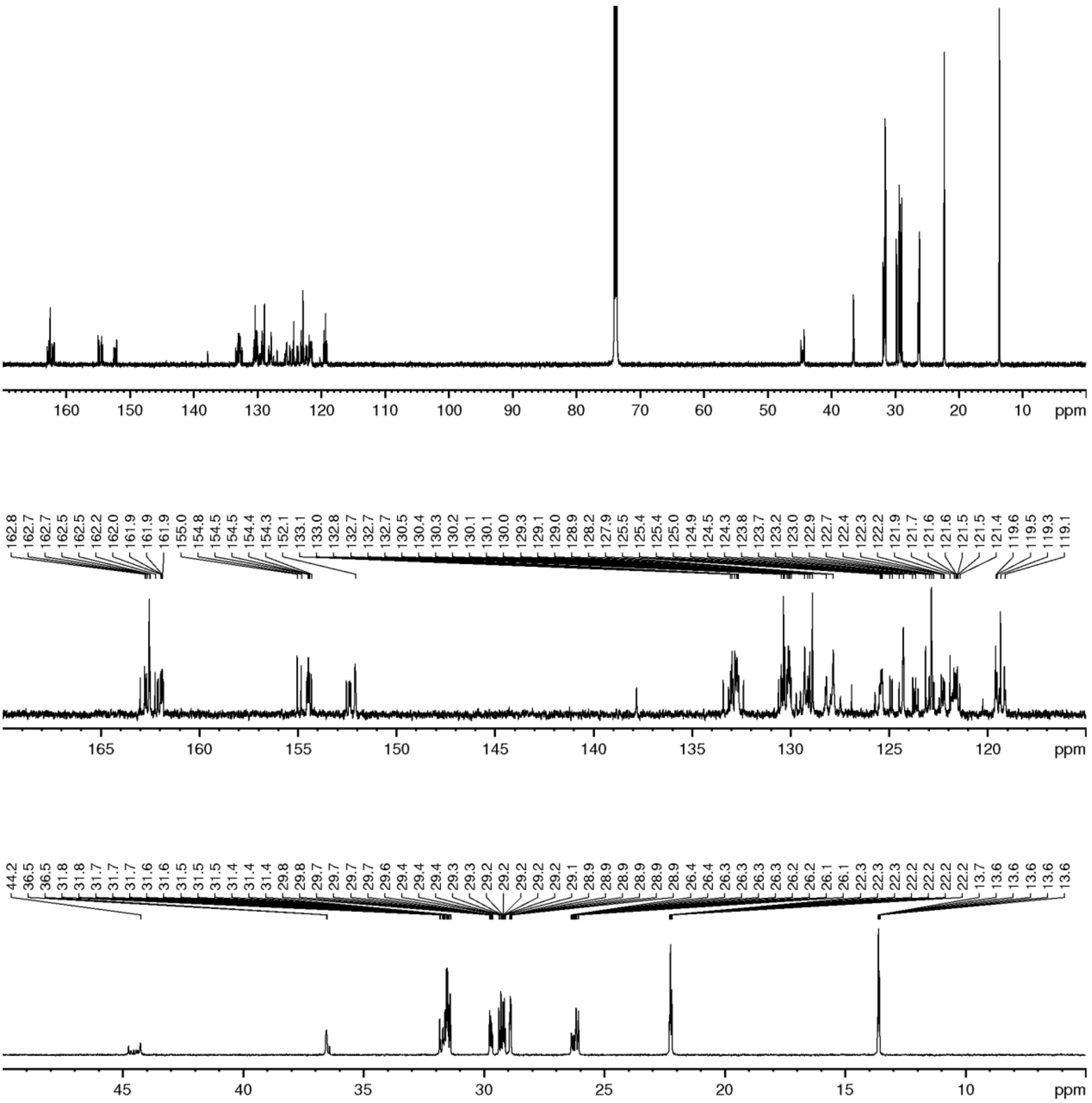

Supplementary Figure 65. ^{13}C NMR spectrum (151 MHz, 384 K, TCE-d_2) of the bromo-phenoxy-substituted pentamer **8** (for structures, see Supplementary Figure 2).

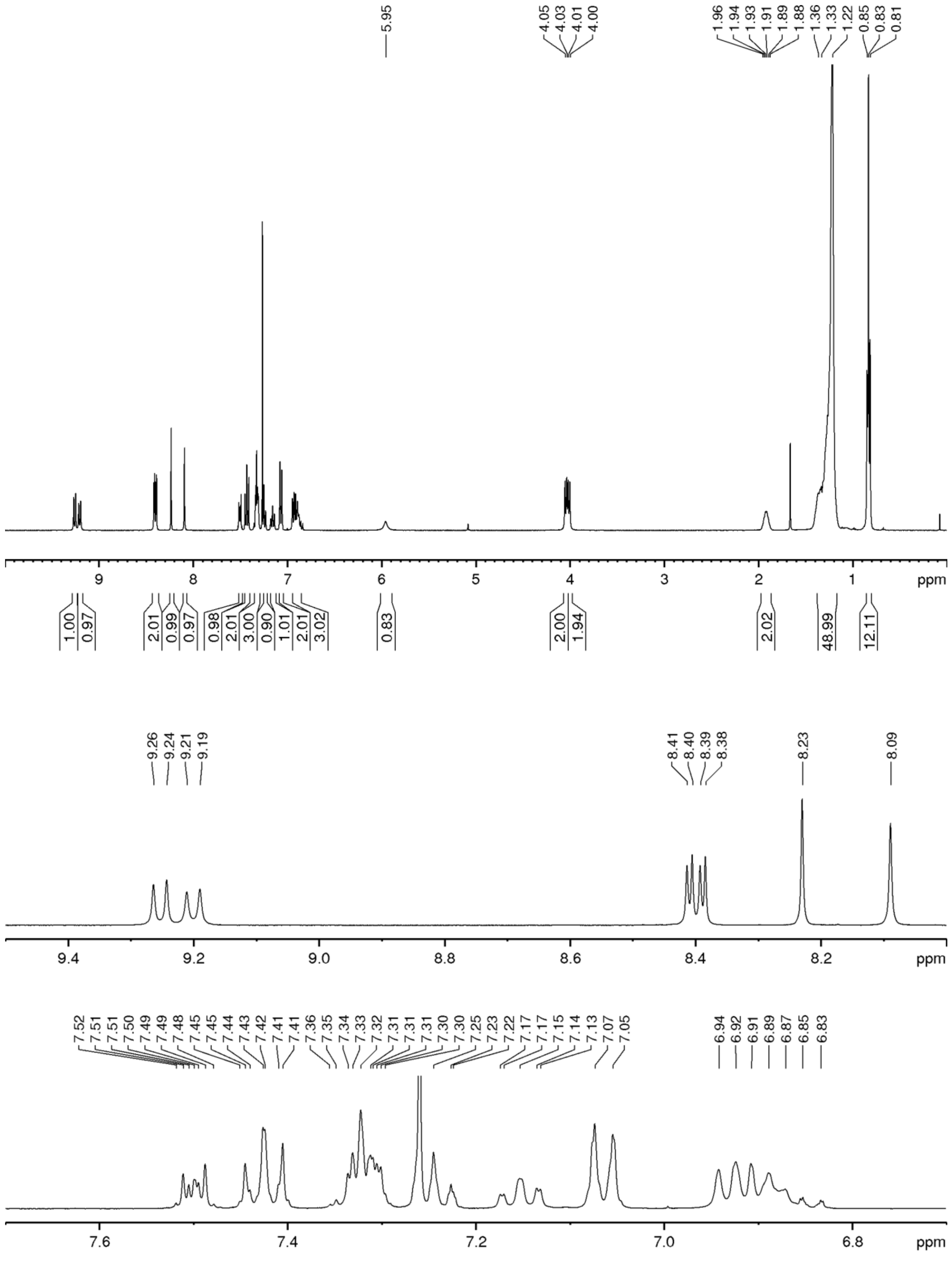

Supplementary Figure 66. ^{1}H NMR spectrum (400 MHz, 295 K, $CDCl_3$) of the monomeric **PBI-Cap1** (for structures, see Supplementary Figure 1).

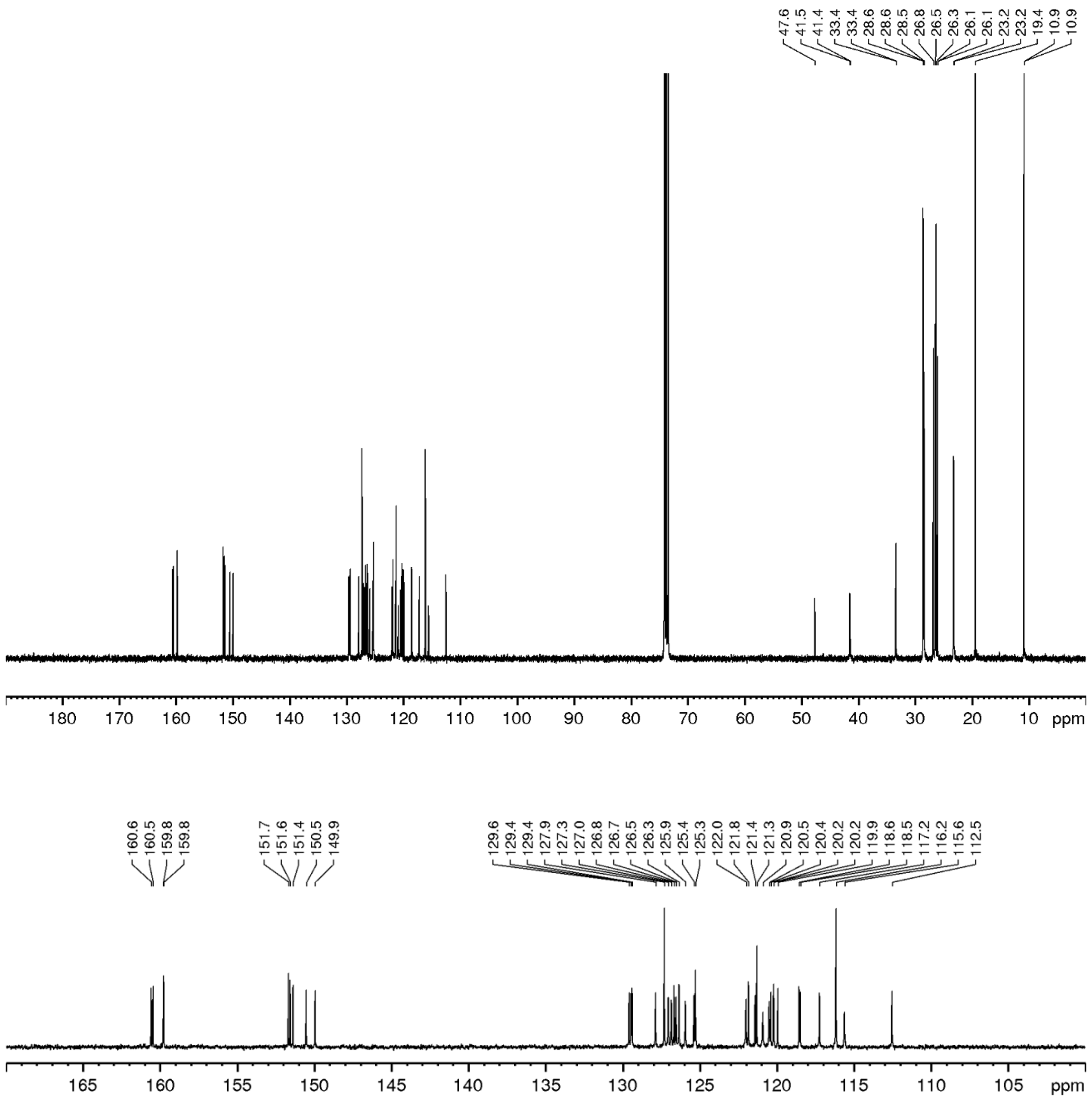

Supplementary Figure 67. ^{13}C NMR spectrum (101 MHz, 295 K, $CDCl_3$) of the monomeric **PBI-Cap1** (for structures, see Supplementary Figure 1).

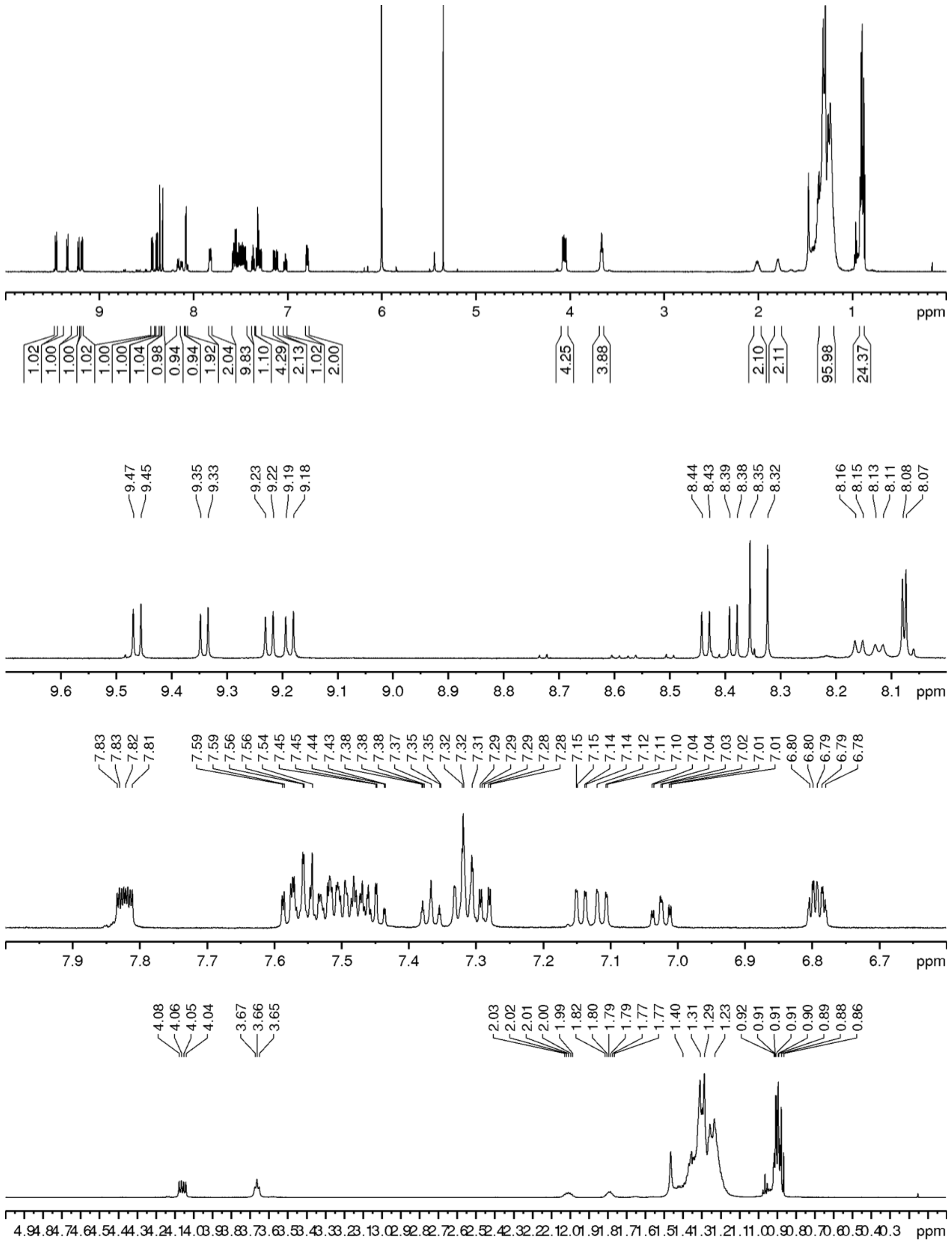

Supplementary Figure 68. ^{1}H NMR spectrum (600 MHz, 384 K, TCE-d_2) of the 2,2´-biphenol-phenoxy-substitued **PBI-Cap2** (for structures, see Supplementary Figure 2).

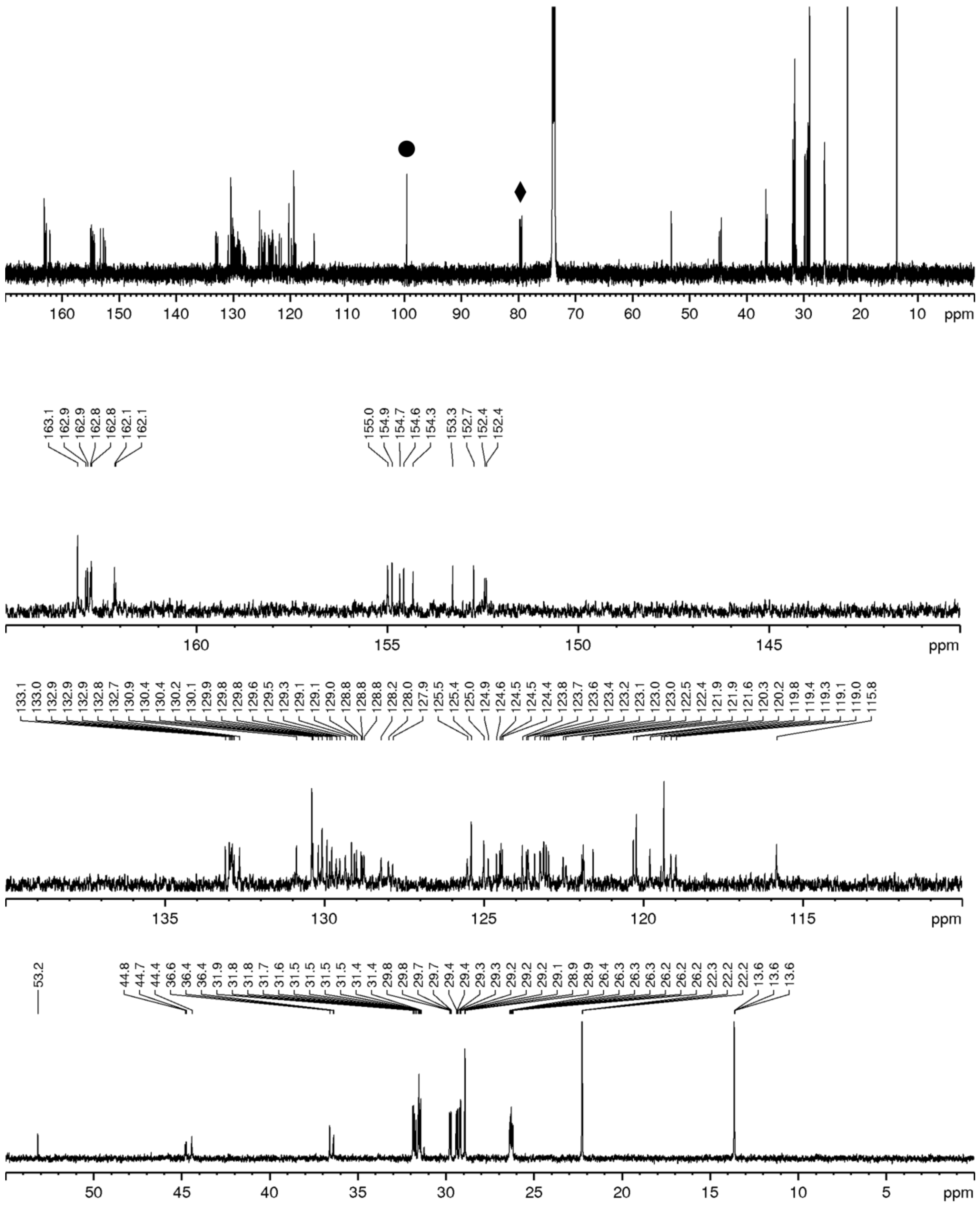

Supplementary Figure 69. ^{13}C NMR spectrum (151 MHz, 384 K, TCE-d_2) of the 2,2´-biphenol-phenoxy-substitued **PBI-Cap2** (for structures, see Supplementary Figure 2). Residual solvents marked as follows: chloroform (♦) and tetrachloromethane (●) present in deuterated 1,1,2,2-tetrachloroethane.[36]

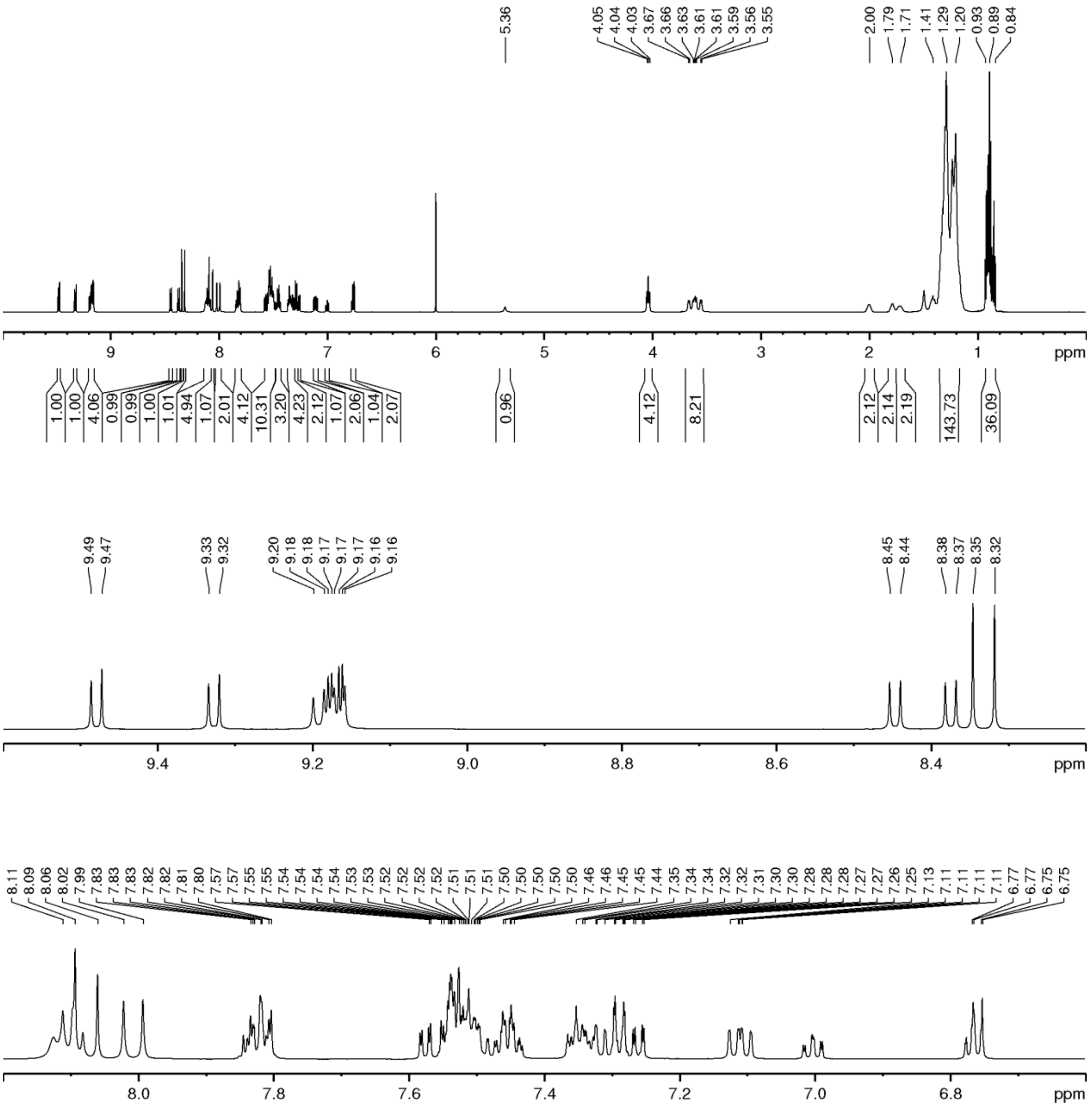

Supplementary Figure 70. ^{1}H NMR spectrum (600 MHz, 384 K, TCE-d_2) of the 2,2´-biphenol-phenoxy-substitued **PBI-Cap3** (for structures, see Supplementary Figure 2).

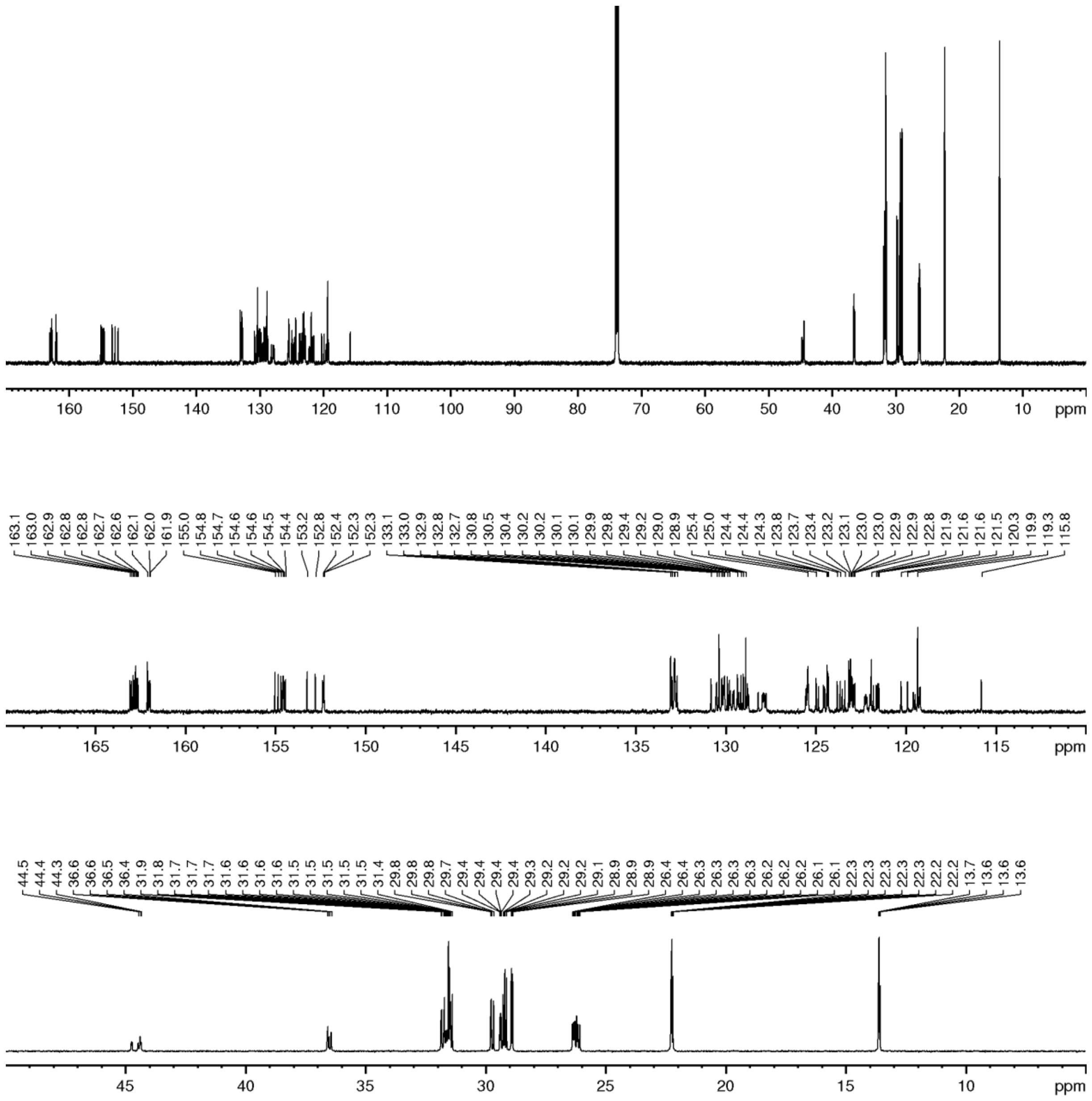

Supplementary Figure 71. ^{13}C NMR spectrum (151 MHz, 384 K, TCE-d_2) of the 2,2´-biphenol-phenoxy-substitued **PBI-Cap3** (for structures, see Supplementary Figure 2).

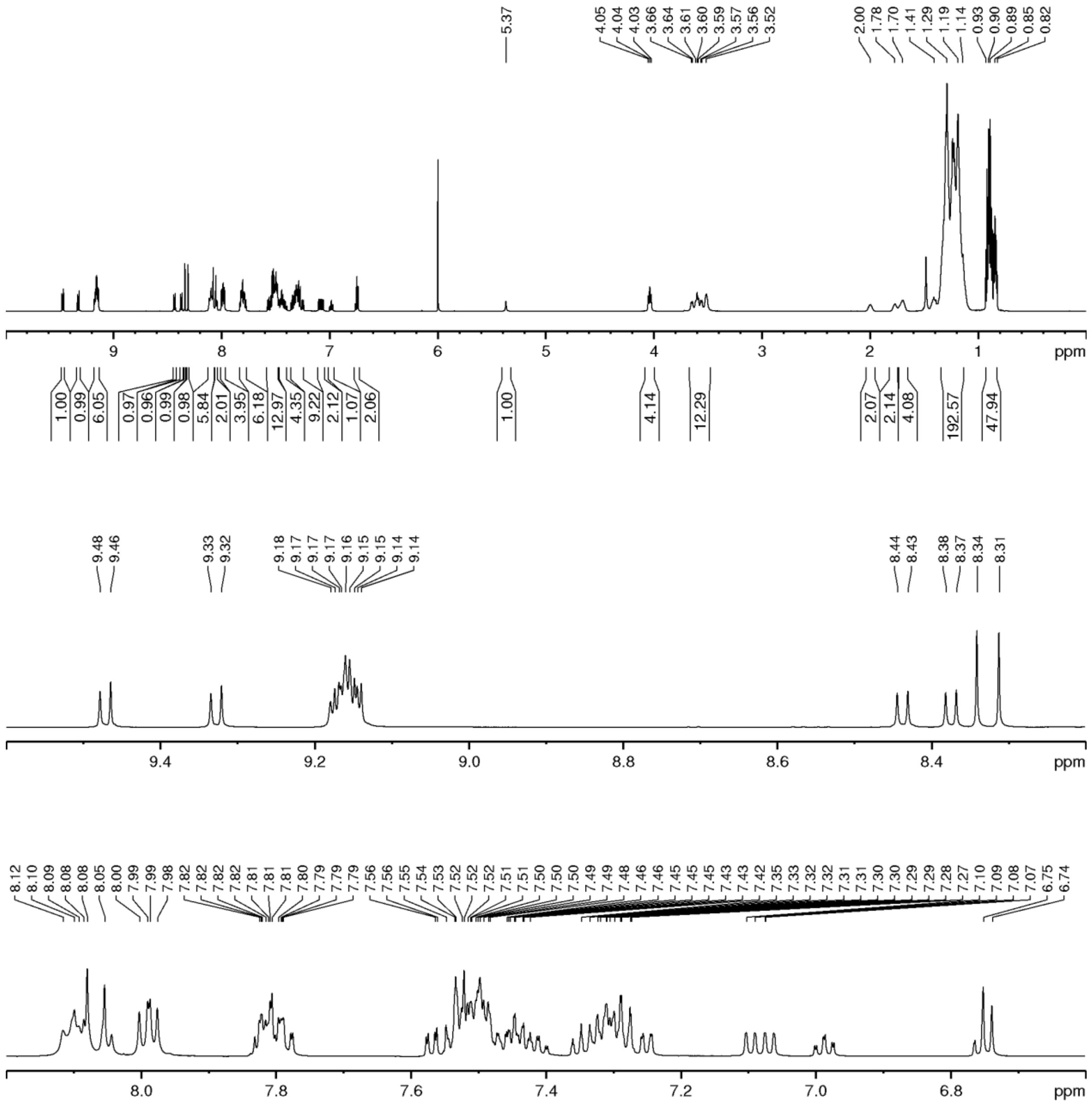

Supplementary Figure 72. ^{1}H NMR spectrum (600 MHz, 384 K, TCE-d_2) of the 2,2´-biphenol-phenoxy-substitued **PBI-Cap4** (for structures, see Supplementary Figure 2).

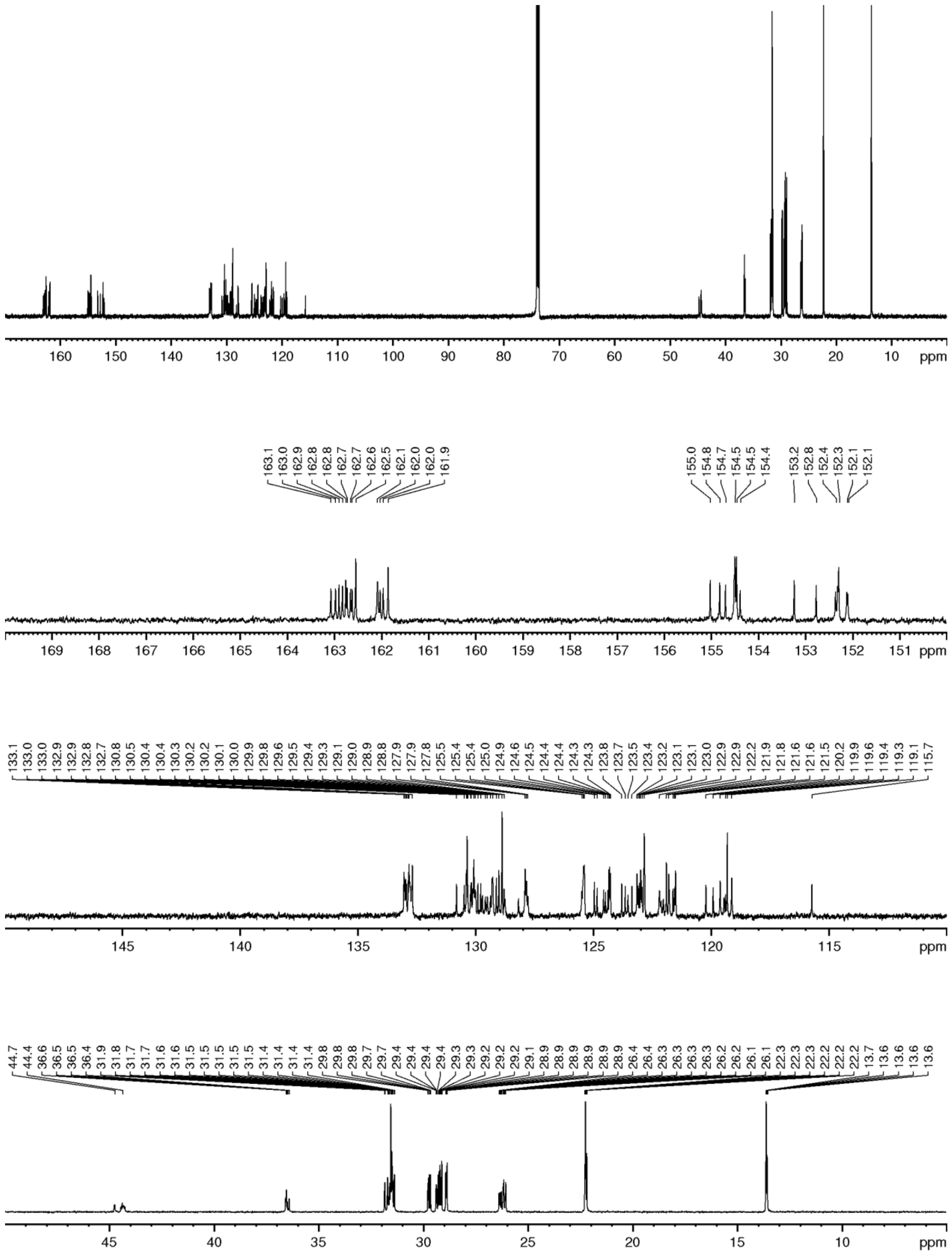

Supplementary Figure 73. ^{13}C NMR spectrum (151 MHz, 384 K, TCE-d_2) of the 2,2´-biphenol-phenoxy-substitued **PBI-Cap4** (for structures, see Supplementary Figure 2).

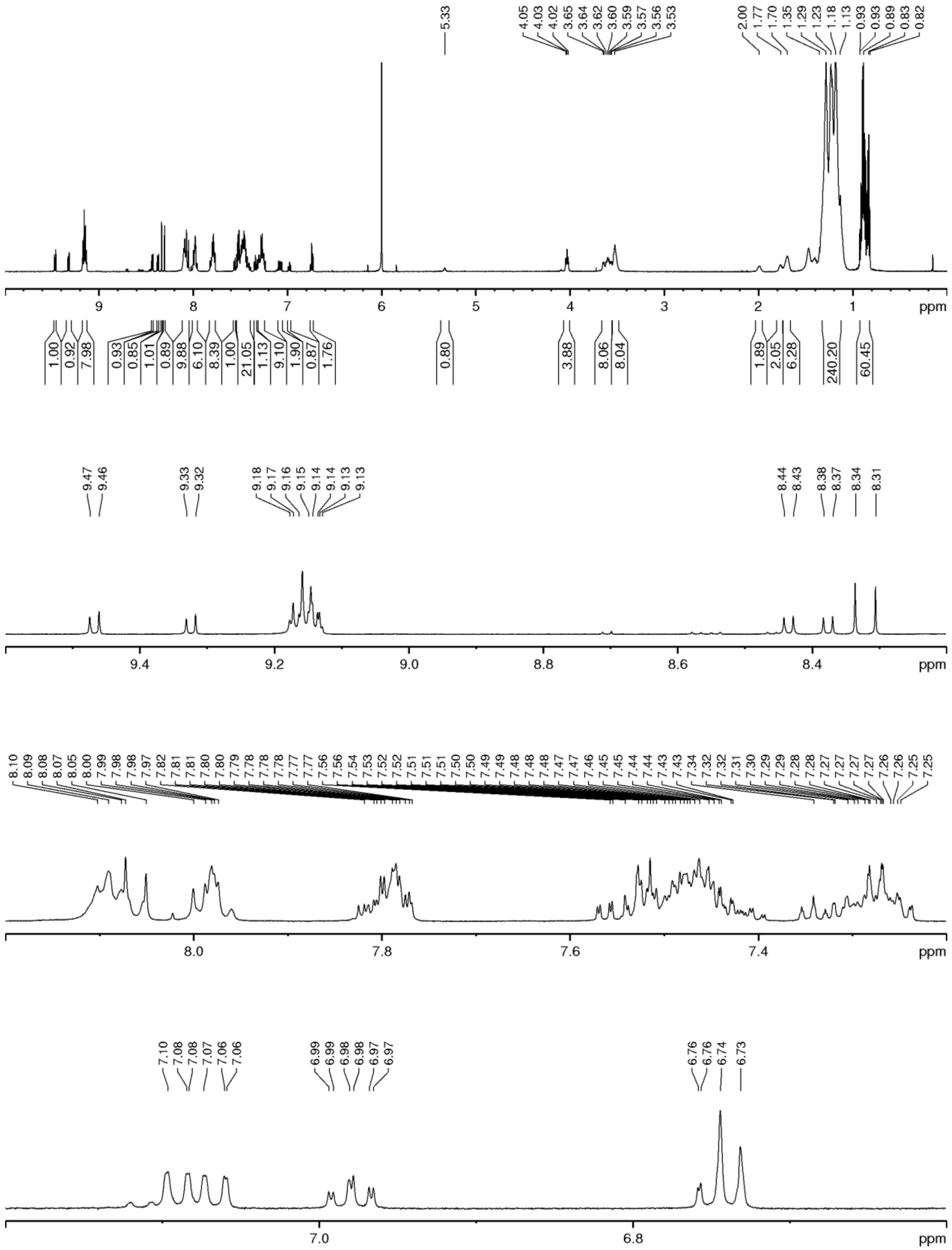

Supplementary Figure 74. ^{1}H NMR spectrum (600 MHz, 384 K, TCE-d_2) of the 2,2´-biphenol-phenoxy-substitued **PBI-Cap5** (for structures, see Supplementary Figure 2).

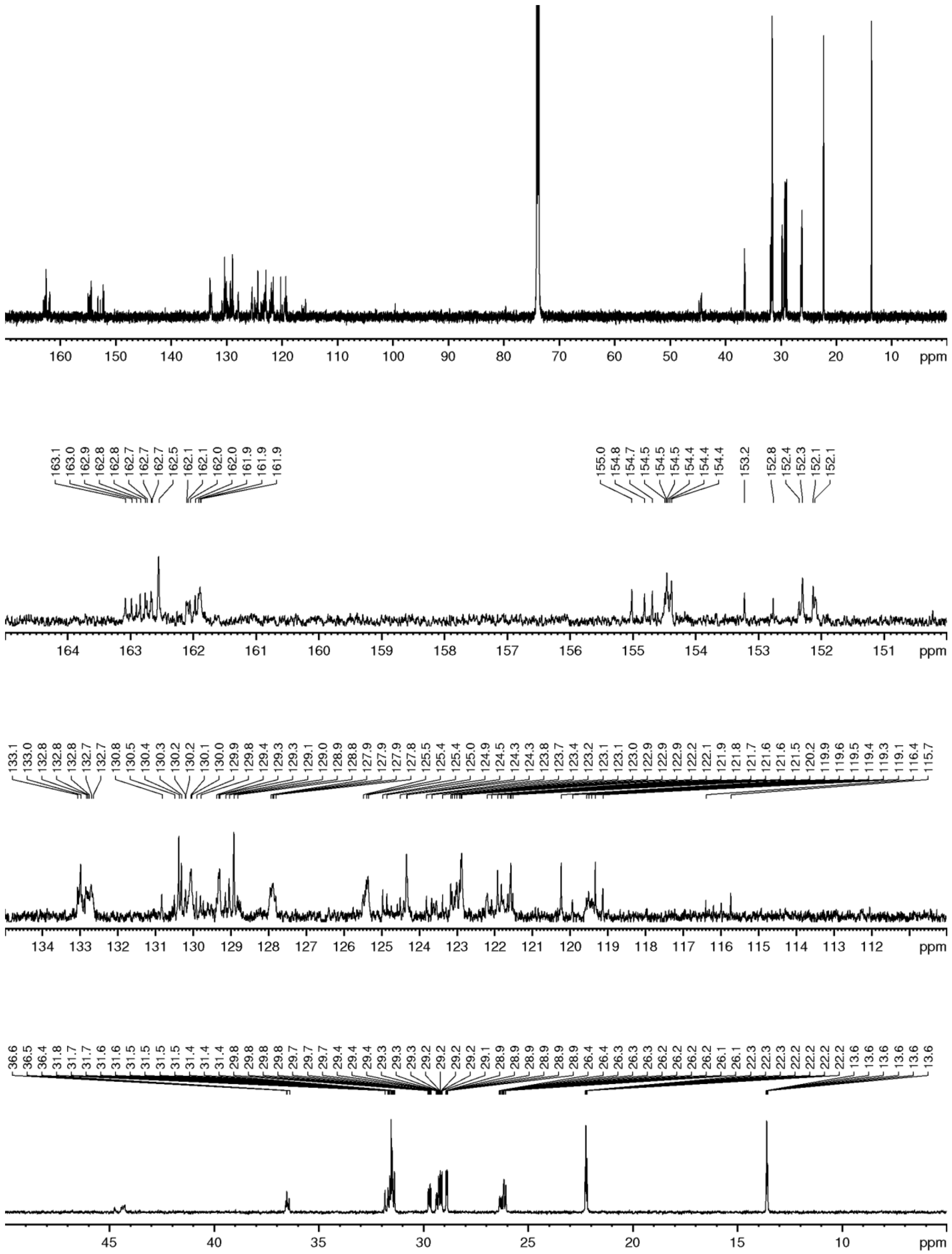

Supplementary Figure 75. ^{13}C NMR spectrum (151 MHz, 384 K, TCE-d_2) of the 2,2´-biphenol-phenoxy-substitued **PBI-Cap5** (for structures, see Supplementary Figure 2).

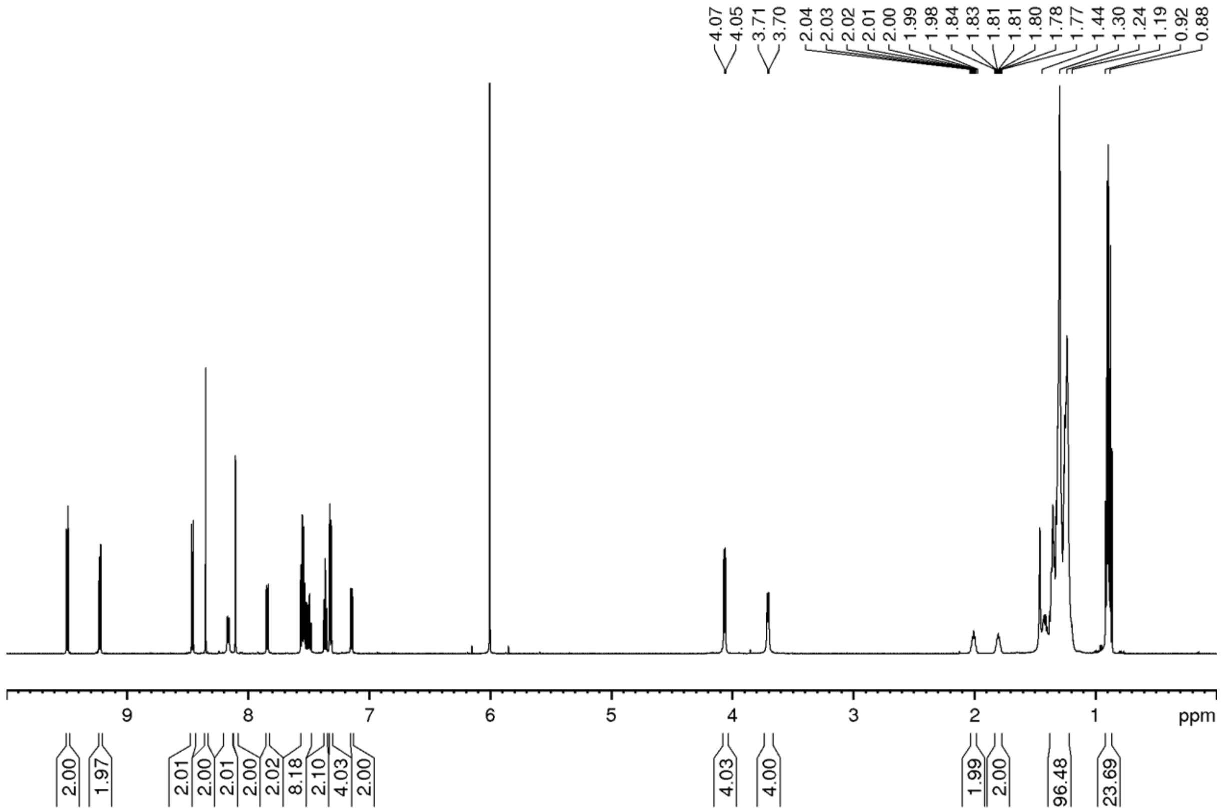

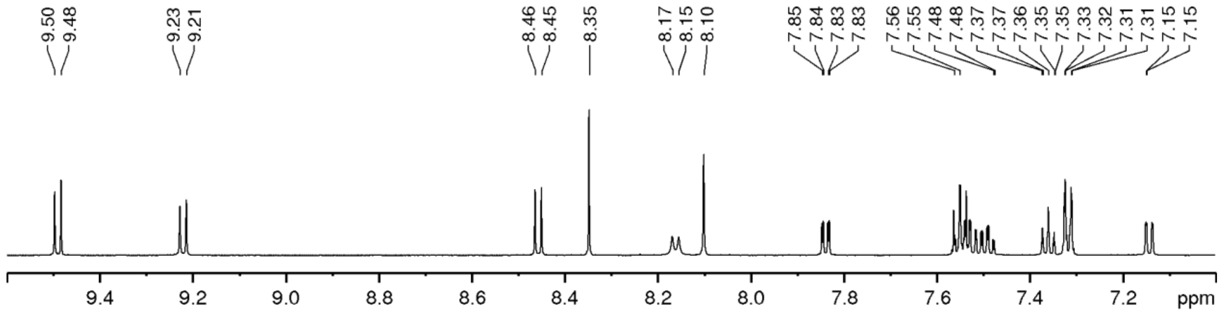

Supplementary Figure 76. ^{1}H NMR spectrum (600 MHz, 384 K, TCE-d_2) of **PBI-2** (for structures, see Supplementary Figure 1).

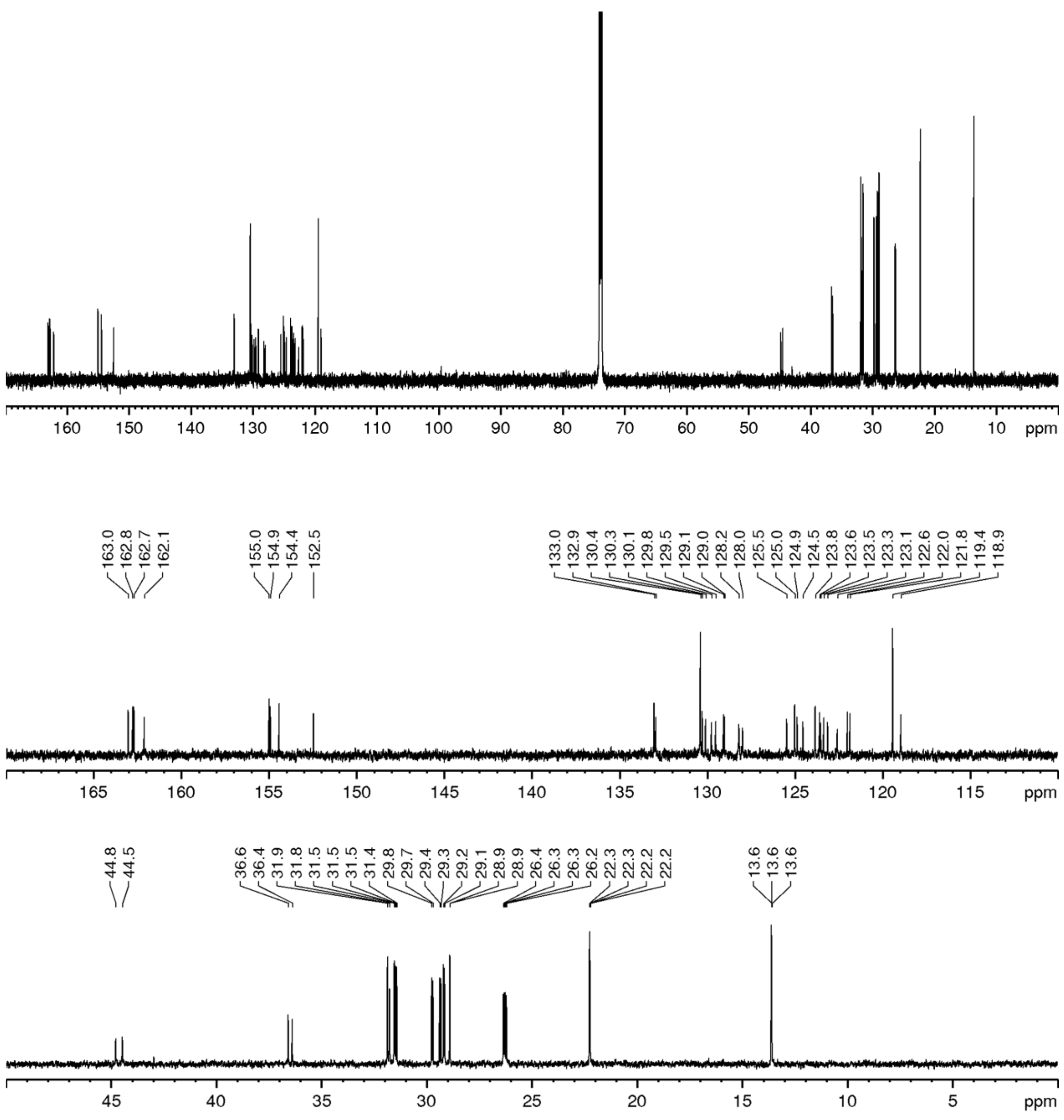

Supplementary Figure 77. ^{13}C NMR spectrum (151 MHz, 384 K, TCE-d_2) of **PBI-2** (for structures, see Supplementary Figure 1).

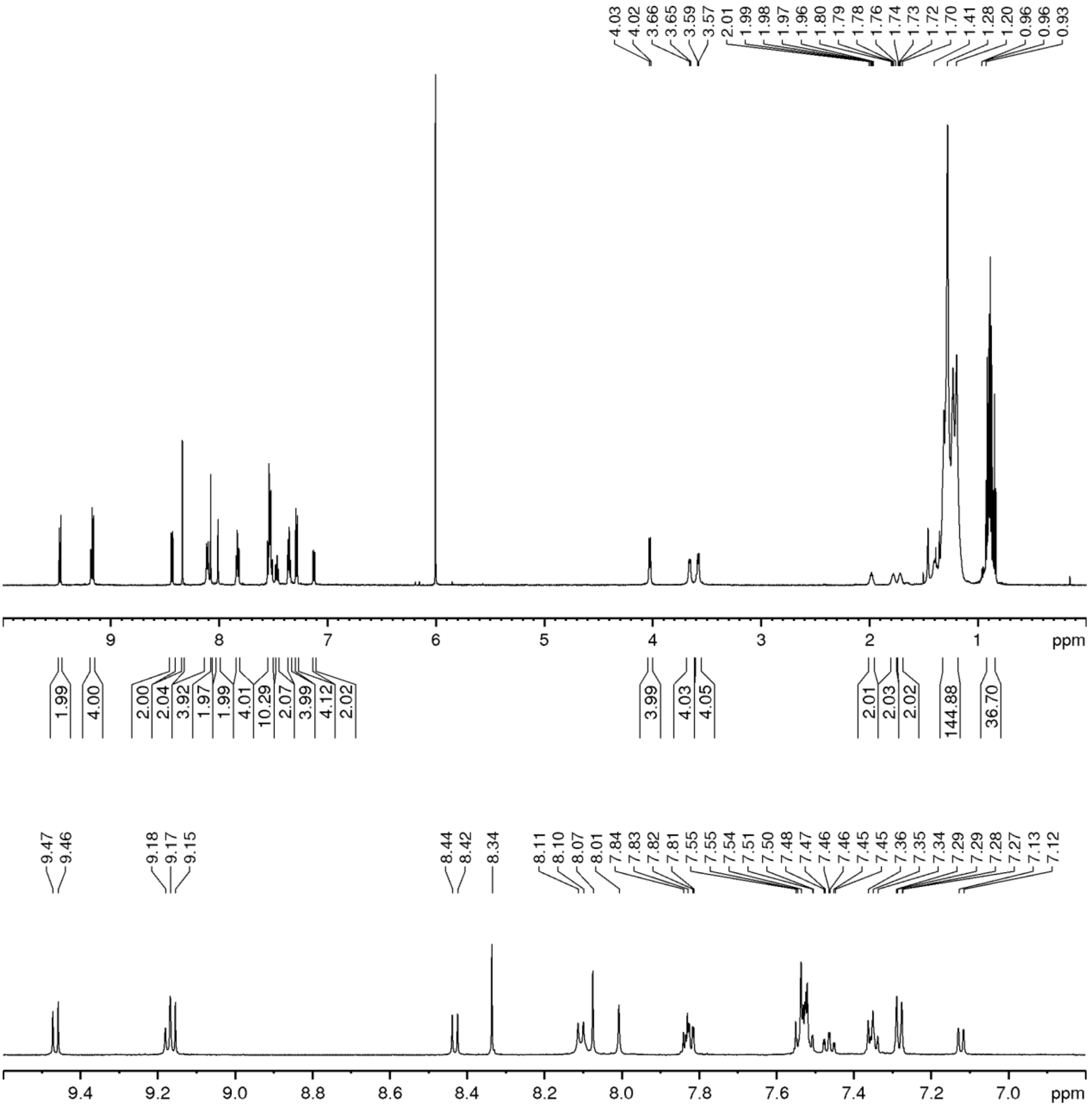

Supplementary Figure 78. ^{1}H NMR spectrum (600 MHz, 384 K, TCE-d_2) of **PBI-3** (for structures, see Supplementary Figure 3).

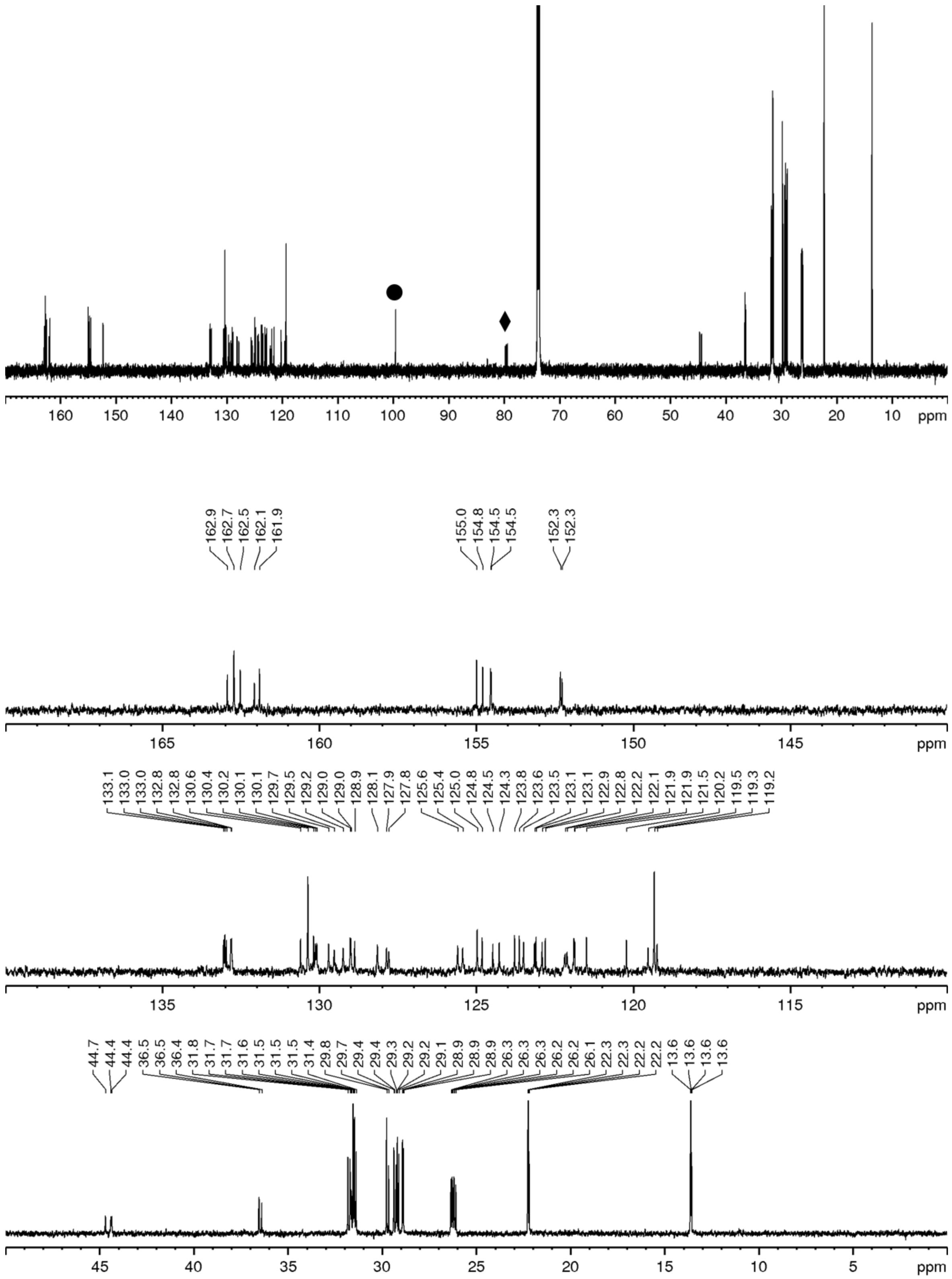

Supplementary Figure 79. ^{13}C NMR spectrum (151 MHz, 384 K, TCE-d_2) of **PBI-3** (for structures, see Supplementary Figure 3). Residual signals are marked as follows: chloroform (♦) and tetrachloromethane (●) present in deuterated 1,1,2,2-tetrachloroethane.[36]

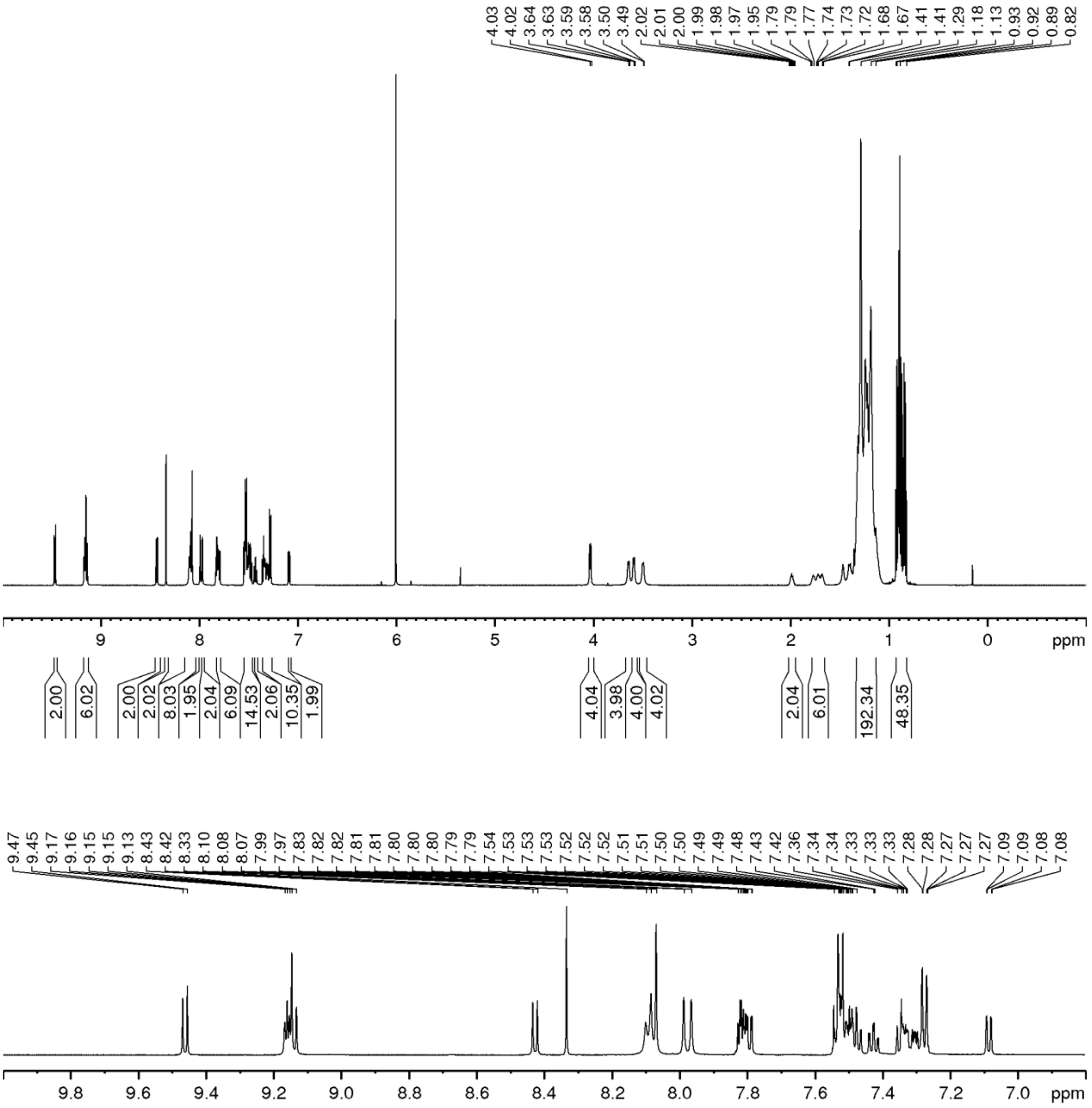

Supplementary Figure 80. ^{1}H NMR spectrum (600 MHz, 384 K, TCE-d_2) of **PBI-4** (for structures, see Supplementary Figure 3).

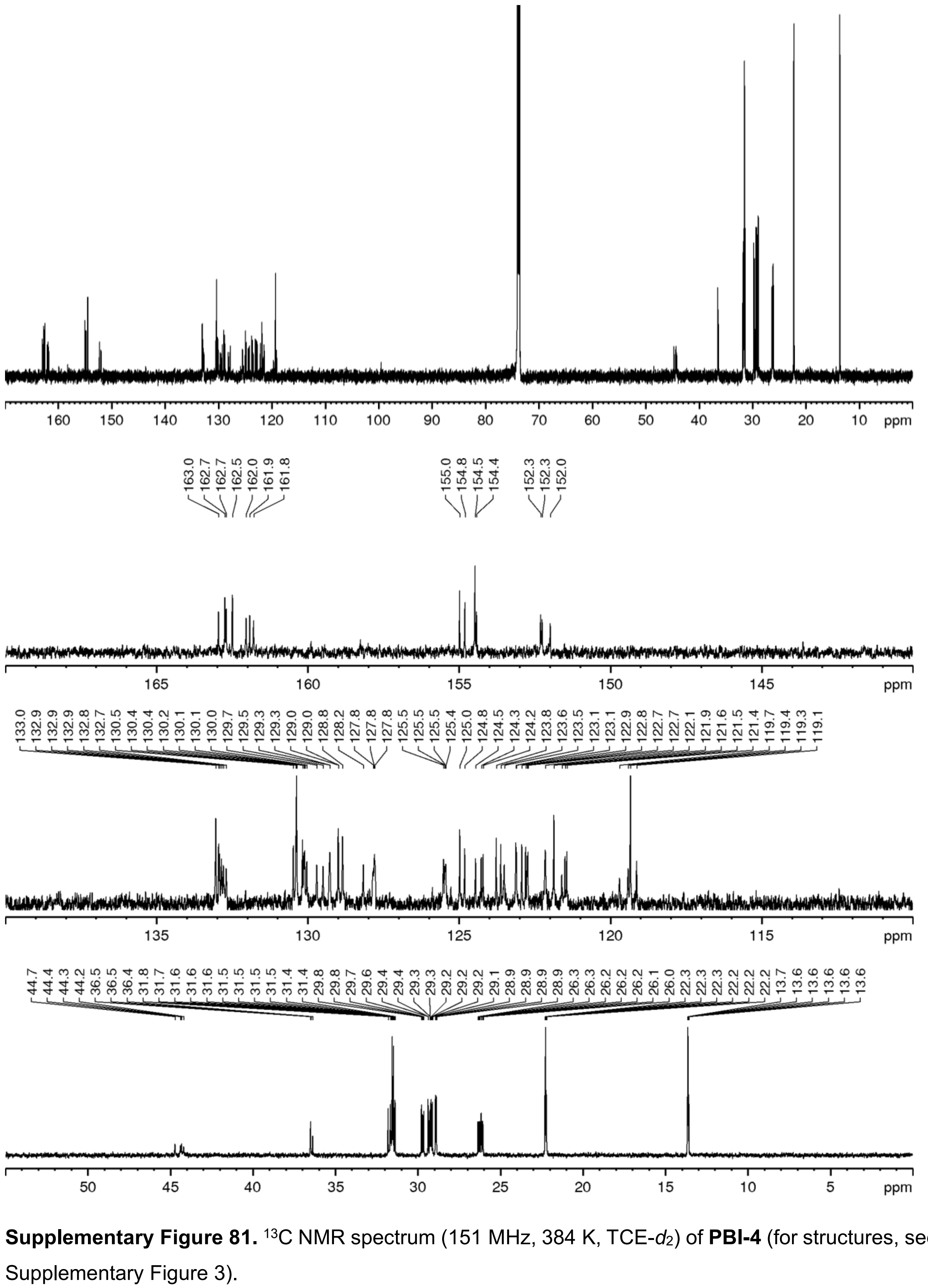

Supplementary Figure 81. ^{13}C NMR spectrum (151 MHz, 384 K, TCE-d_2) of **PBI-4** (for structures, see Supplementary Figure 3).

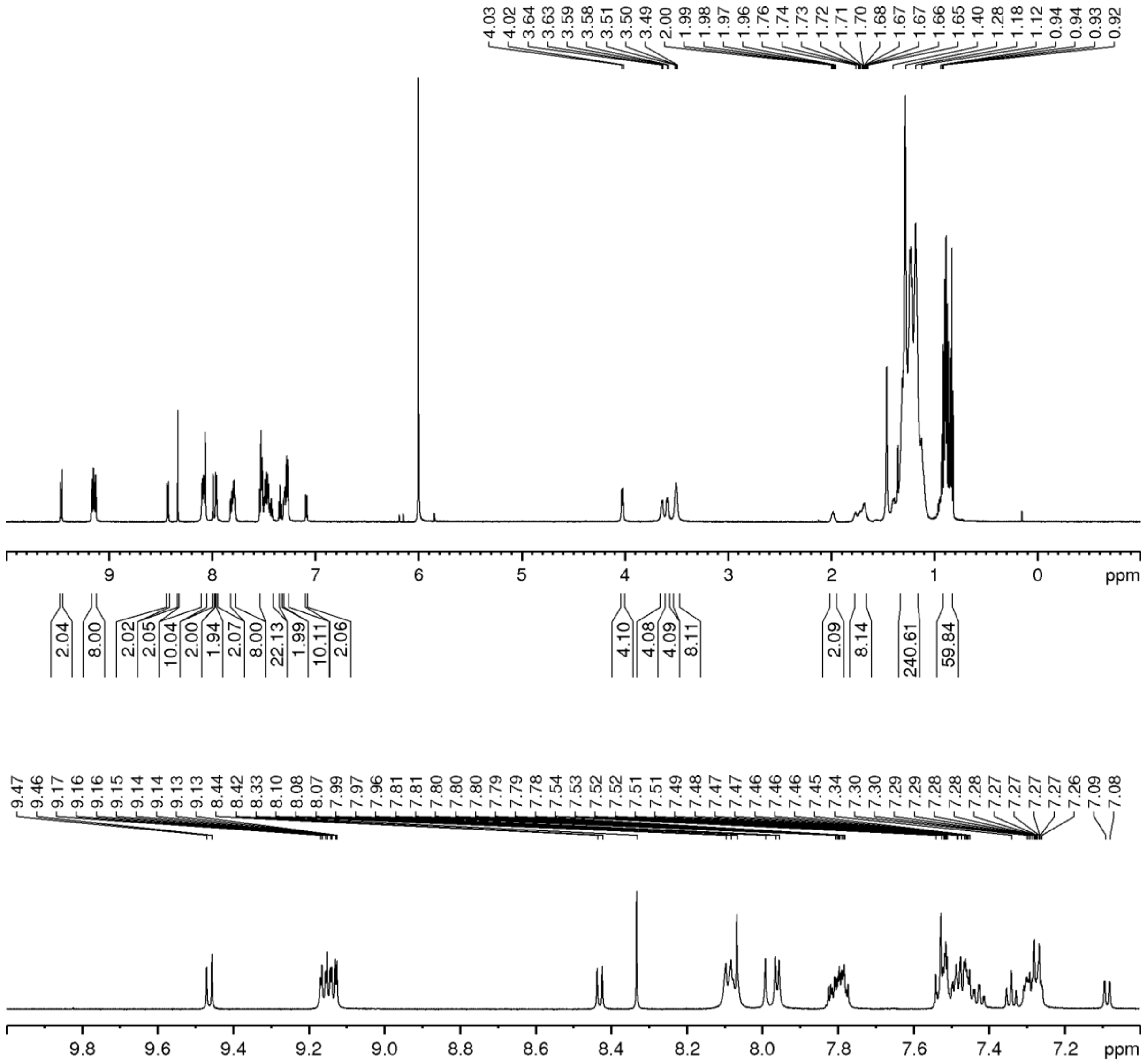

Supplementary Figure 82. ^{1}H NMR spectrum (600 MHz, 384 K, TCE-d_2) of **PBI-5** (for structures, see Supplementary Figure 3).

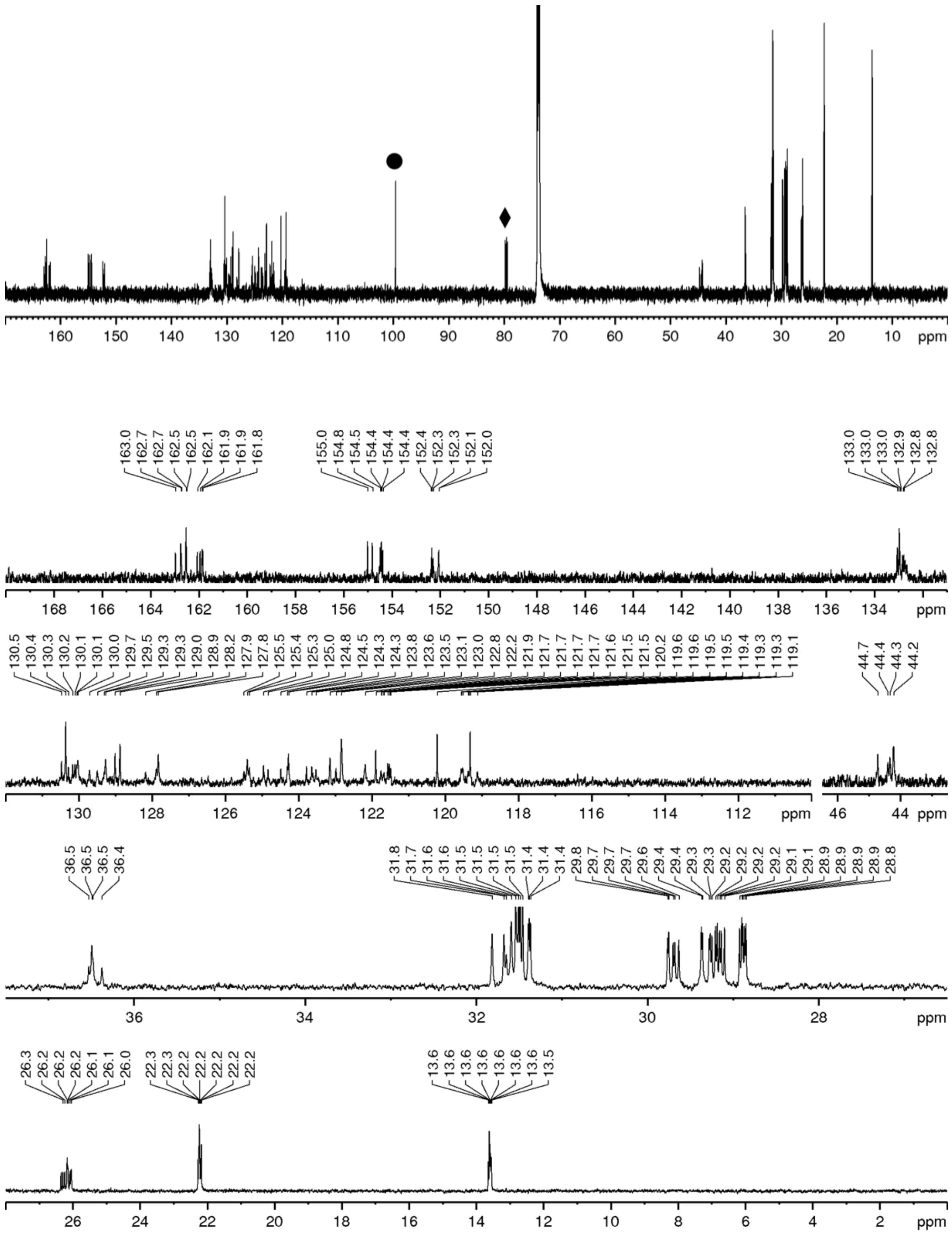

Supplementary Figure 83. ^{13}C NMR spectrum (151 MHz, 384 K, TCE-d_2) of **PBI-5** (for structures, see Supplementary Figure 3). Residual signals are marked as follows: chloroform (♦) and tetrachloromethane (●) present in deuterated 1,1,2,2-tetrachloroethane.[36]

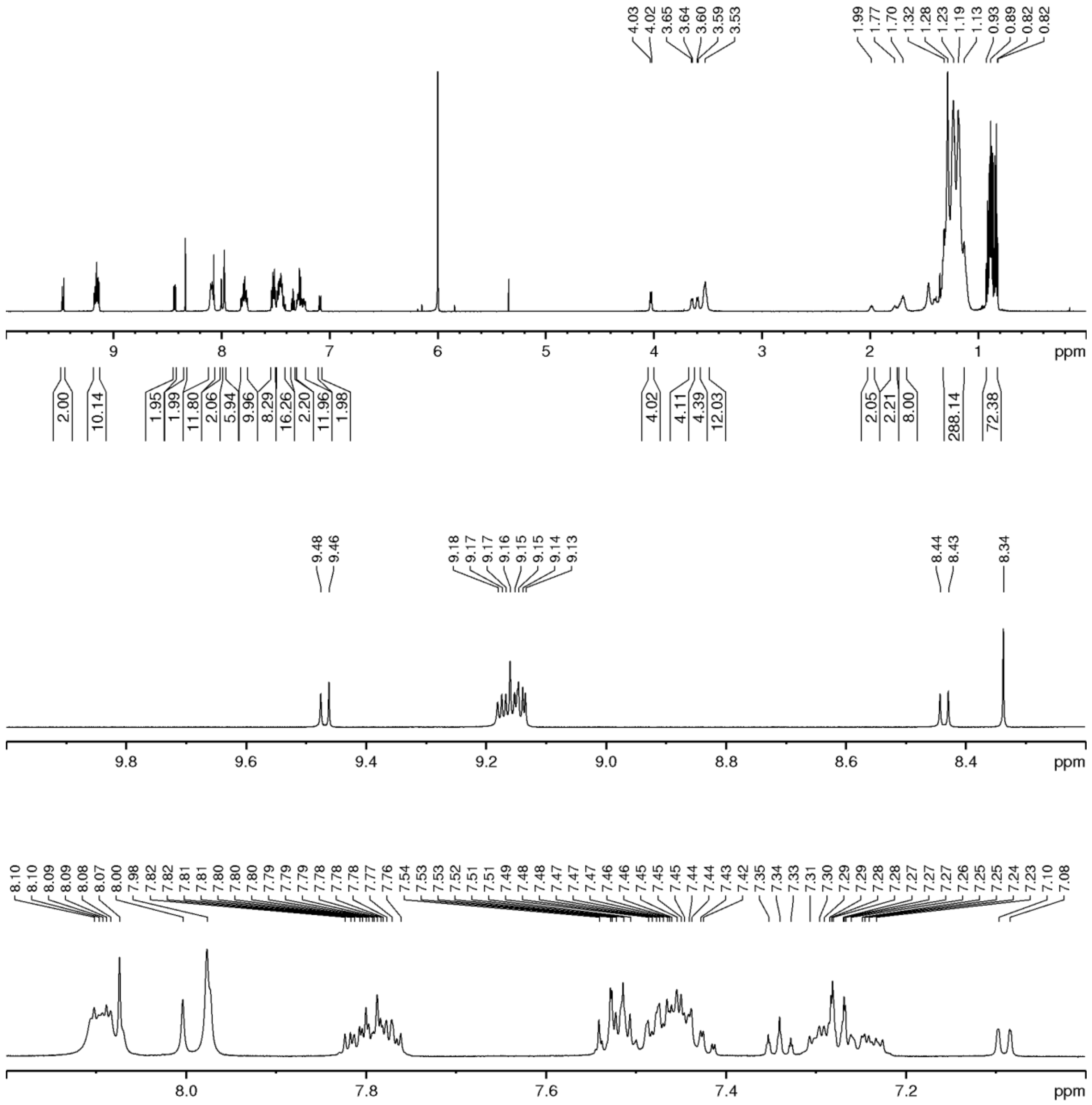

Supplementary Figure 84. ^{1}H NMR spectrum (600 MHz, 384 K, TCE-d_2) of **PBI-6** (for structures, see Supplementary Figure 4).

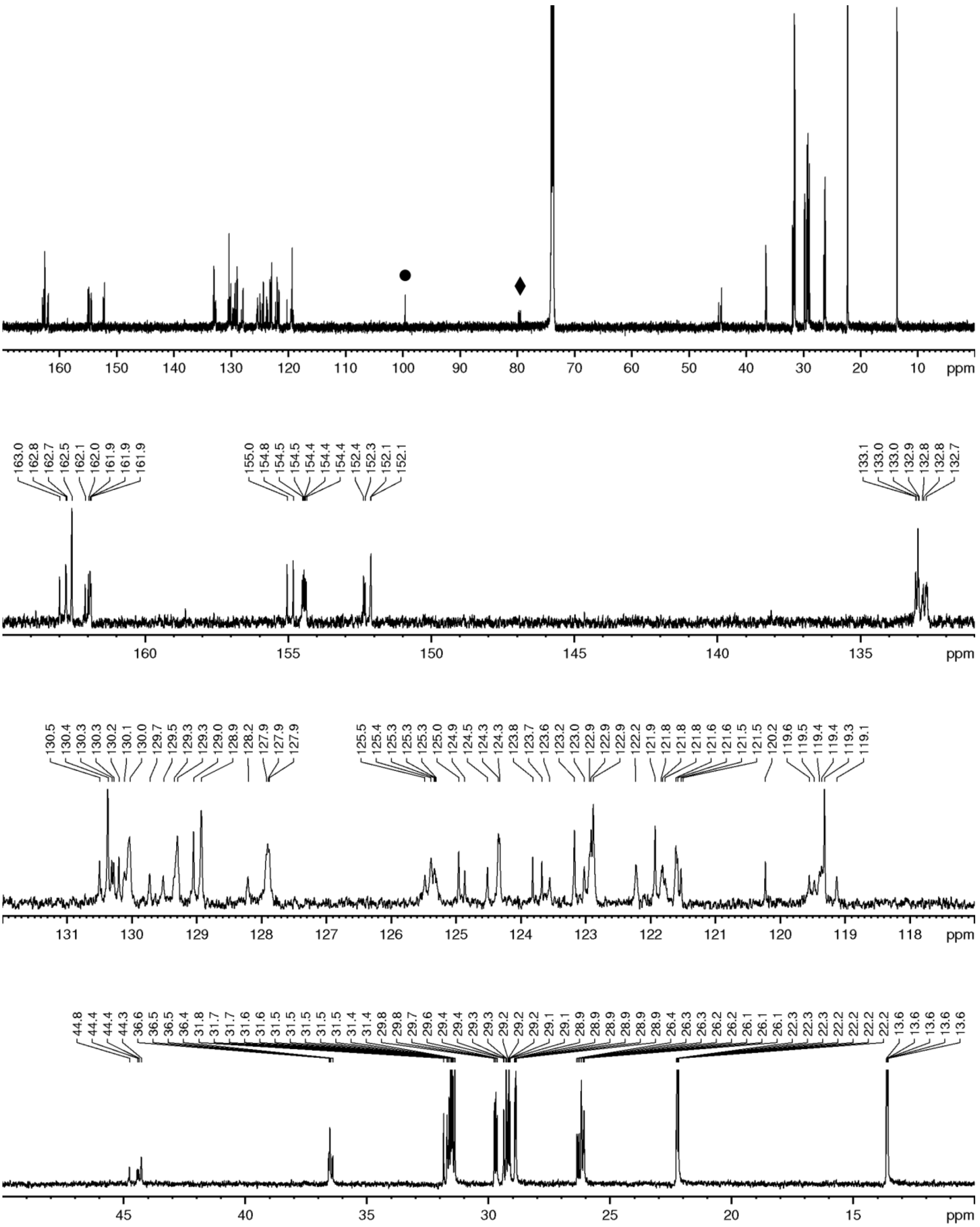

Supplementary Figure 85. ^{13}C NMR spectrum (151 MHz, 384 K, TCE-d_2) of **PBI-6** (for structures, see Supplementary Figure 4). Residual signals are marked as follows: chloroform (♦) and tetrachloromethane (●) present in deuterated 1,1,2,2-tetrachloroethane.[36]

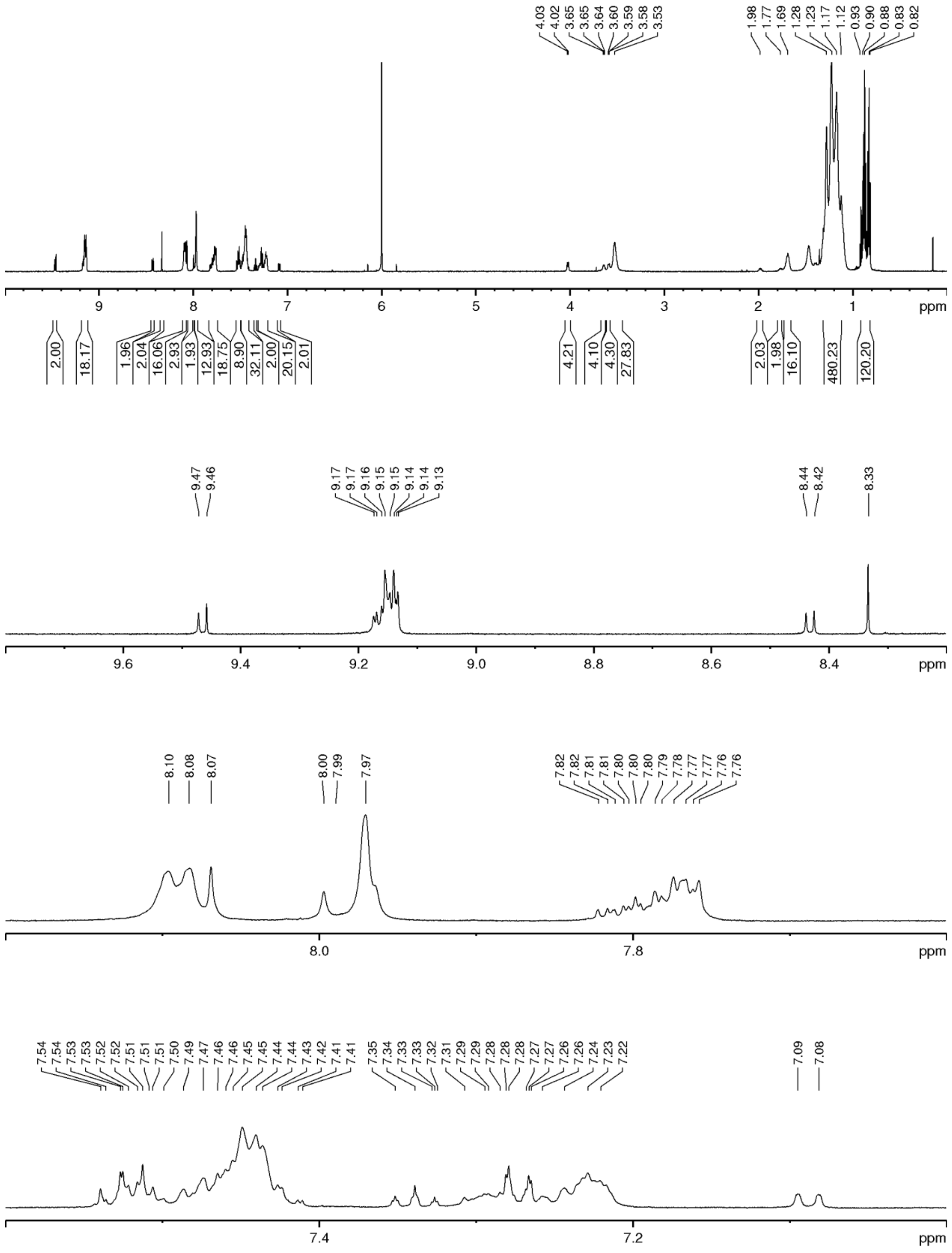

Supplementary Figure 86. ^{1}H NMR spectrum (600 MHz, 384 K, TCE-d_2) of **PBI-10** (for structures, see Supplementary Figure 4).

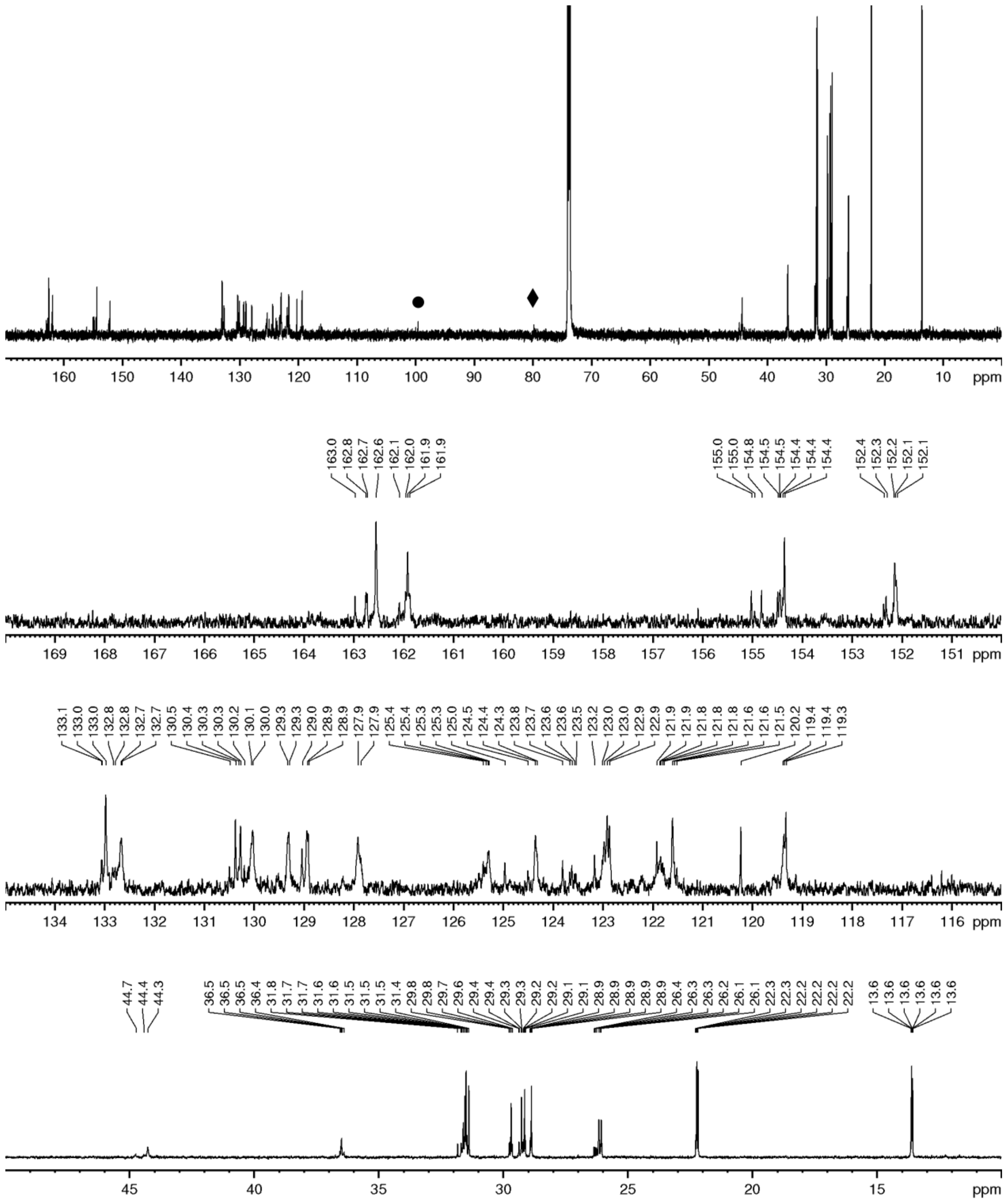

Supplementary Figure 87. ^{13}C NMR spectrum (151 MHz, 384 K, TCE-d_2) of **PBI-10** (for structures, see Supplementary Figure 4). Residual signals are marked as follows: chloroform (♦) and tetrachloromethane (●) present in deuterated 1,1,2,2-tetrachloroethane.[36]

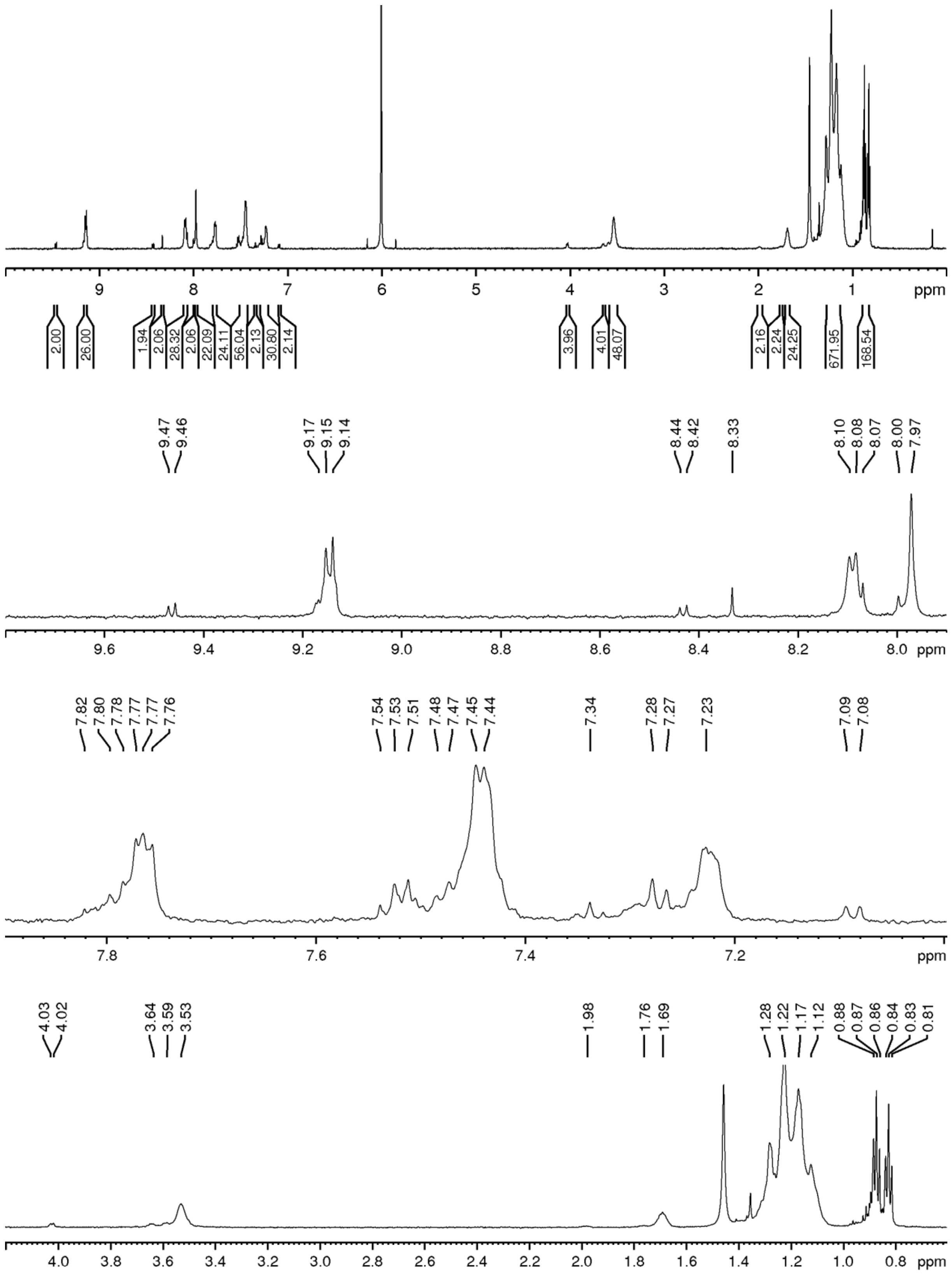

Supplementary Figure 88. ^{1}H NMR spectrum (600 MHz, 384 K, TCE-d_2) of **PBI-14** (for structures, see Supplementary Figure 4).

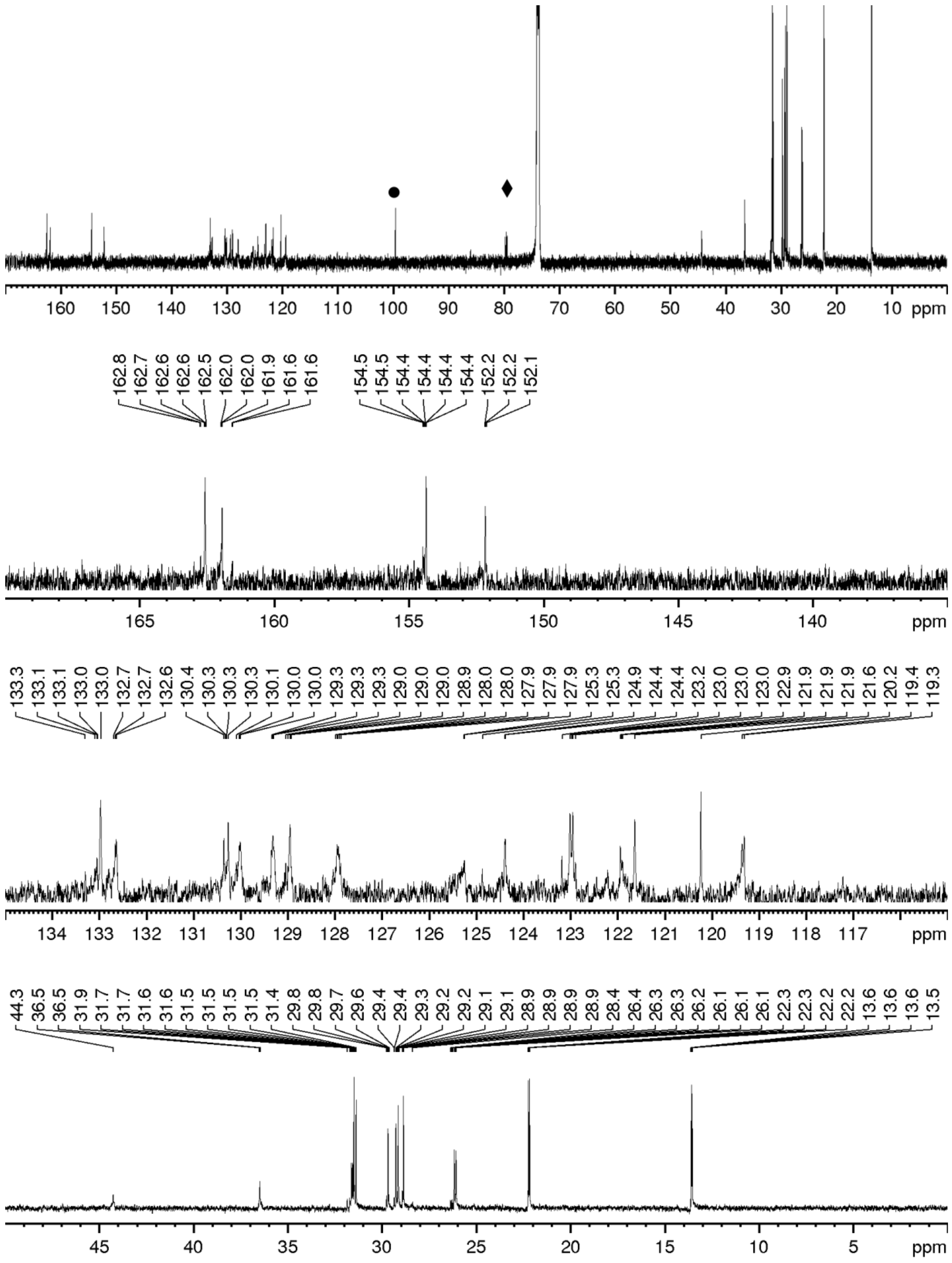

Supplementary Figure 89. ^{13}C NMR spectrum (151 MHz, 384 K, TCE-d_2) of **PBI-14** (for structures, see Supplementary Figure 4). Residual signals are marked as follows: chloroform (♦) and tetrachloromethane (●) present in deuterated 1,1,2,2-tetrachloroethane.[36]

8. Mass Spectrometry

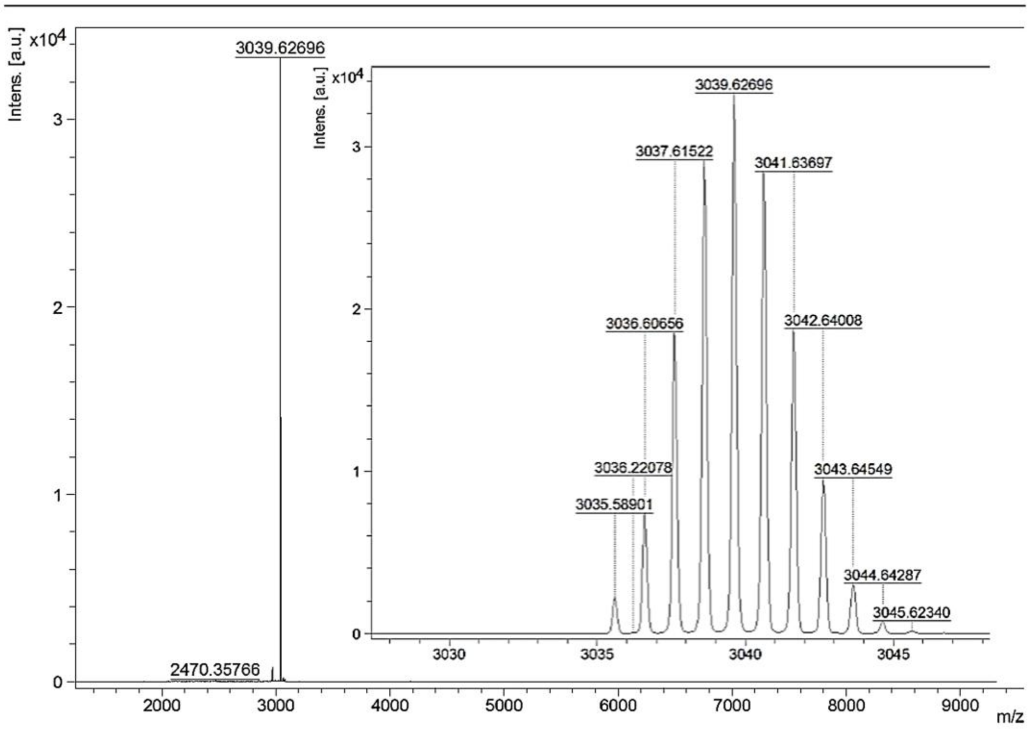

Supplementary Figure 90. HRMS (MALDI-TOF, positive mode, DCTB in chloroform) spectrum of **PBI-Center3**.

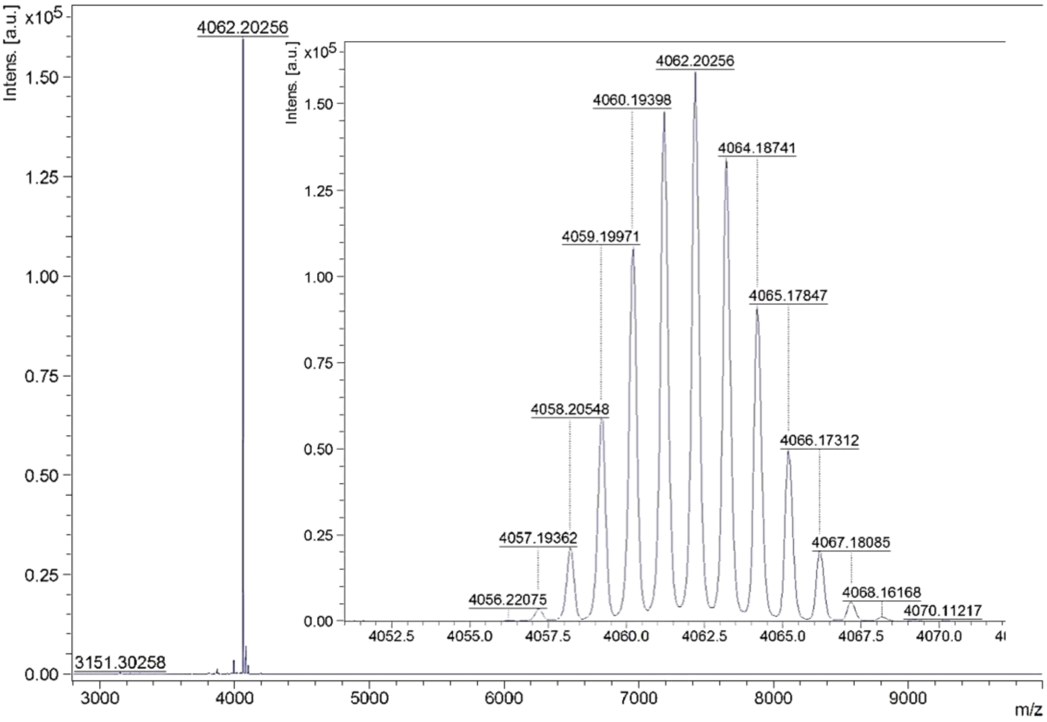

Supplementary Figure 91. HRMS (MALDI-TOF, positive mode, DCTB in chloroform) spectrum of **PBI-Center4**.

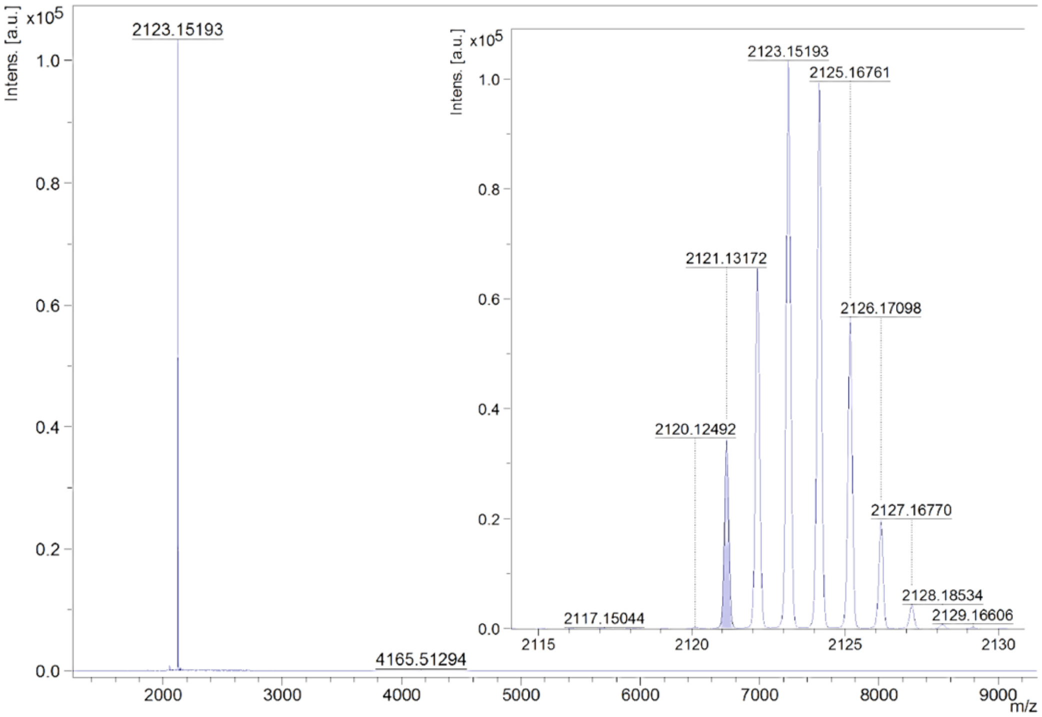

Supplementary Figure 92. HRMS (MALDI-TOF, positive mode, DCTB in chloroform) spectrum of the 2,2´-biphenol-bromo-substitued dimer **2**.

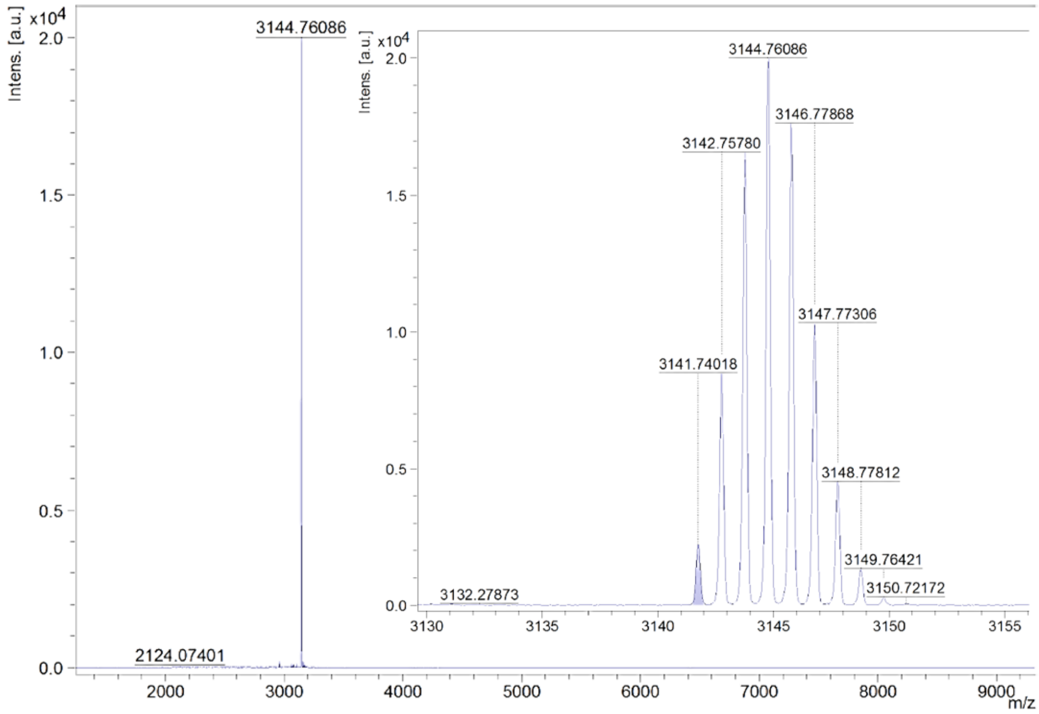

Supplementary Figure 93. HRMS (MALDI-TOF, positive mode, DCTB in chloroform) spectrum of 2,2´-biphenol-bromo-substitued trimer **3**.

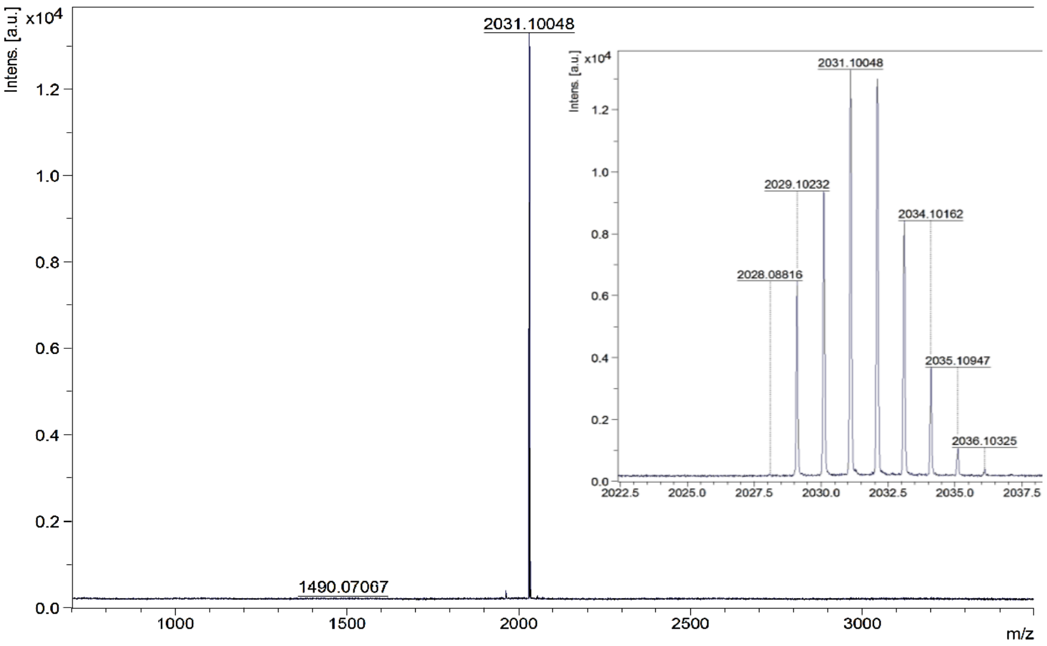

Supplementary Figure 94. HRMS (MALDI-TOF, positive mode, DCTB in chloroform) spectrum of the bromo-phenoxy-substituted dimer **5**.

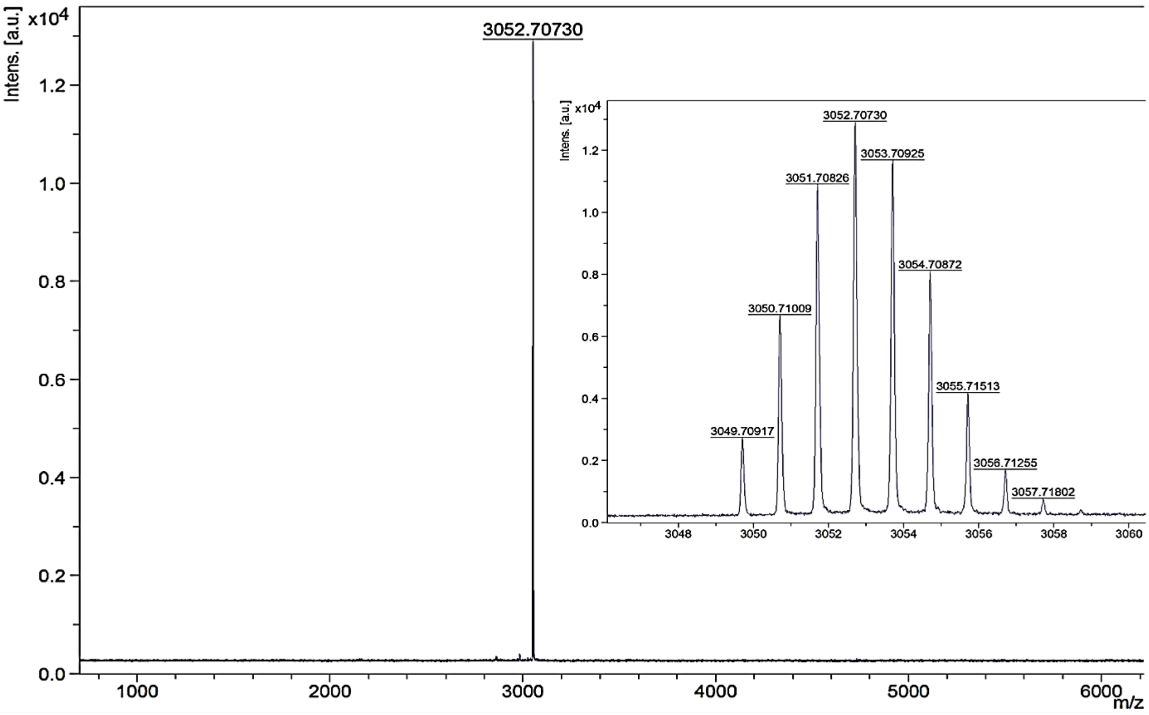

Supplementary Figure 95. HRMS (MALDI-TOF, positive mode, DCTB in chloroform) spectrum of the bromo-phenoxy-substituted trimer **6**.

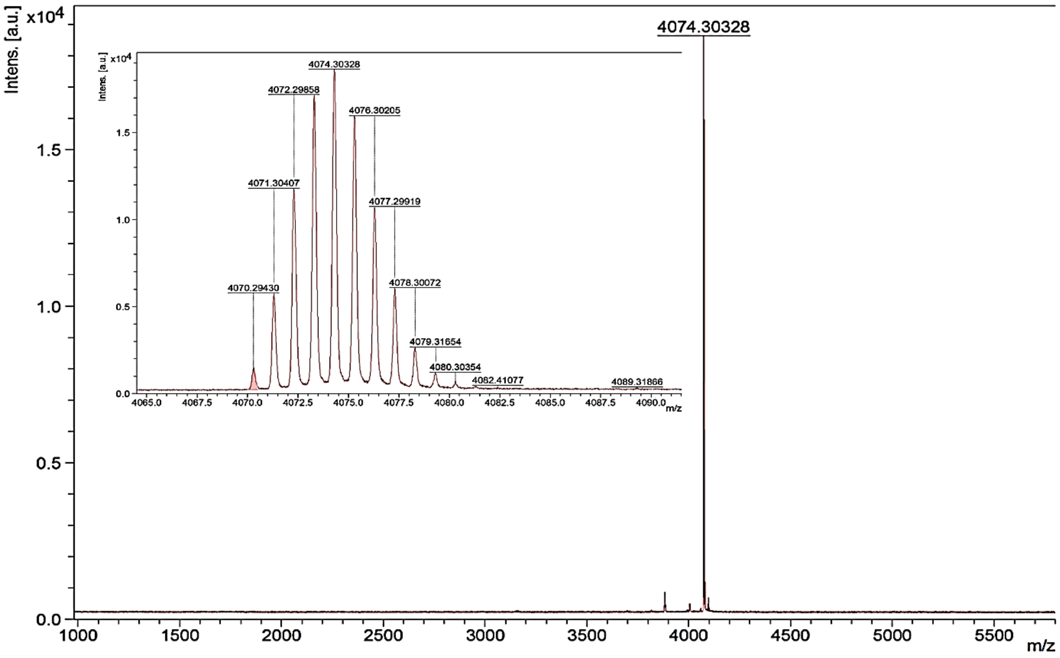

Supplementary Figure 96. HRMS (MALDI-TOF, positive mode, DCTB in chloroform) spectrum of the bromo-phenoxy-substituted tetramer **7**.

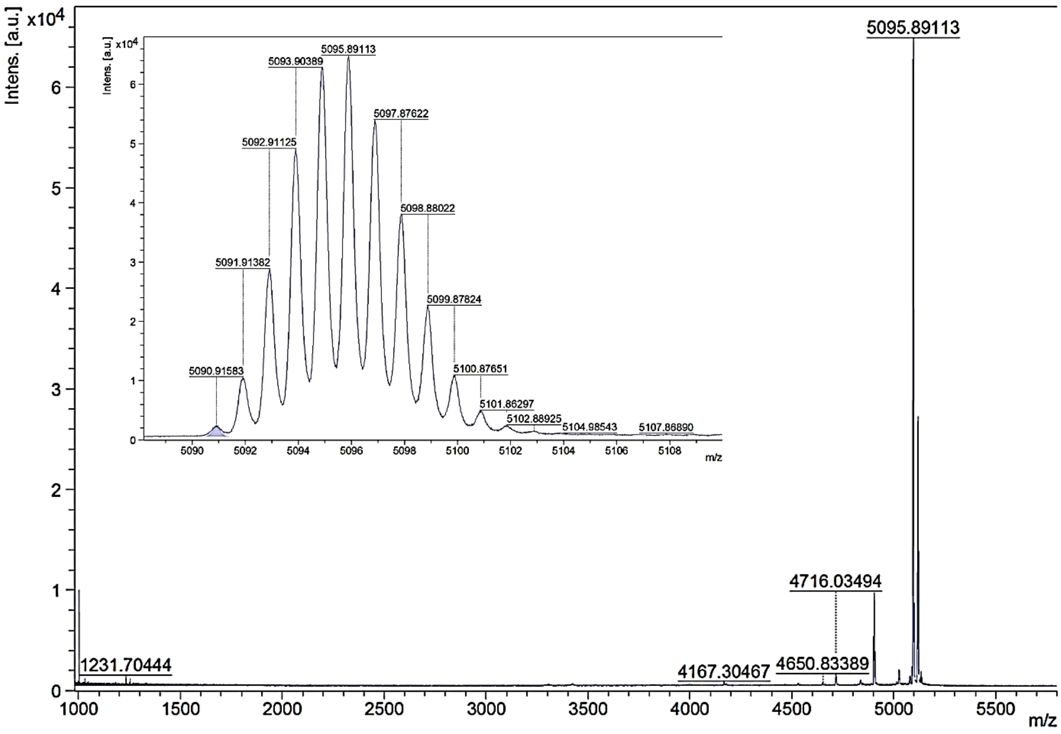

Supplementary Figure 97. HRMS (MALDI-TOF, positive mode, DCTB in chloroform) spectrum of the bromo-phenoxy-substituted pentamer **8**.

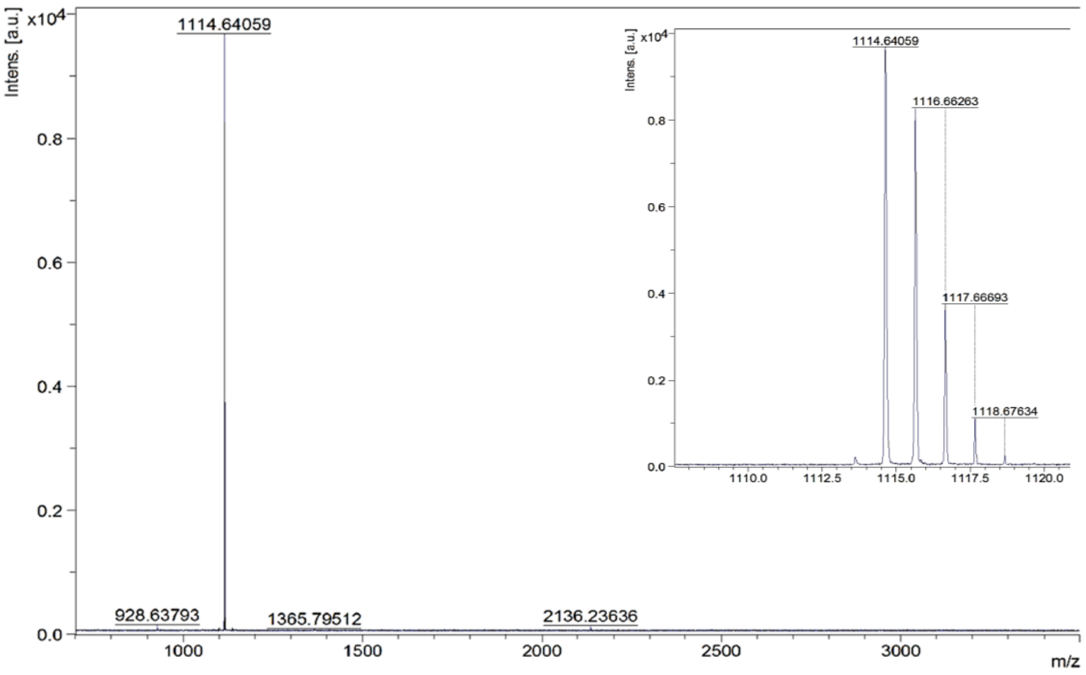

Supplementary Figure 98. HRMS (MALDI-TOF, positive mode, DCTB in chloroform) spectrum of the monomeric **PBI-Cap1**.

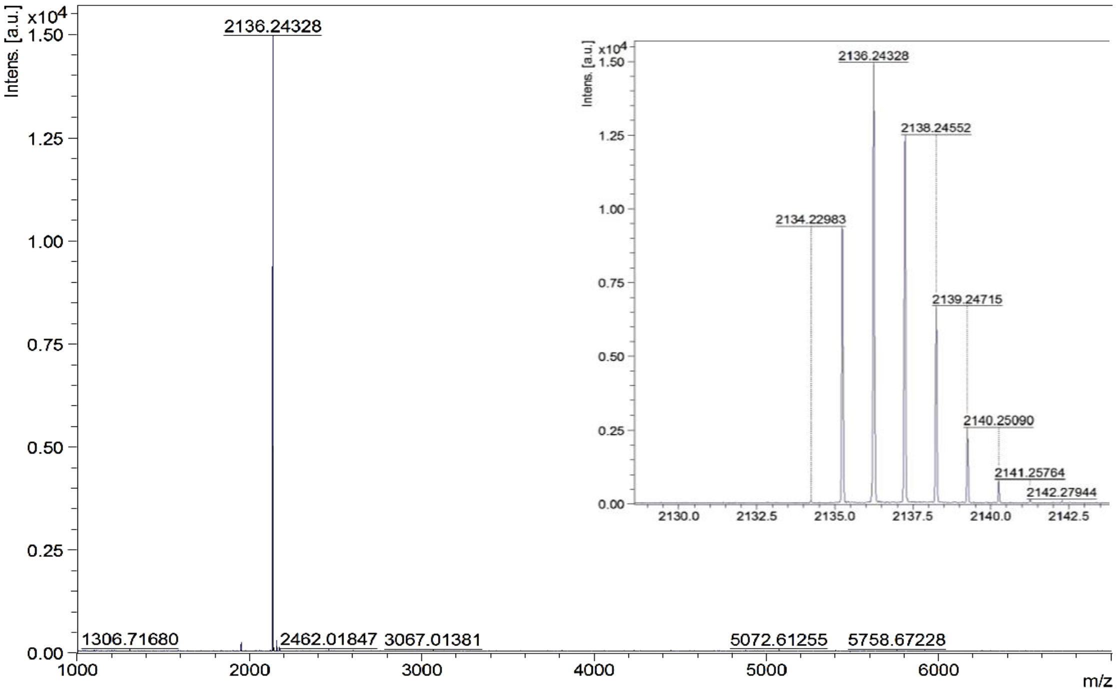

Supplementary Figure 99. HRMS (MALDI-TOF, positive mode, DCTB in chloroform) spectrum of the monomeric **PBI-Cap2**.

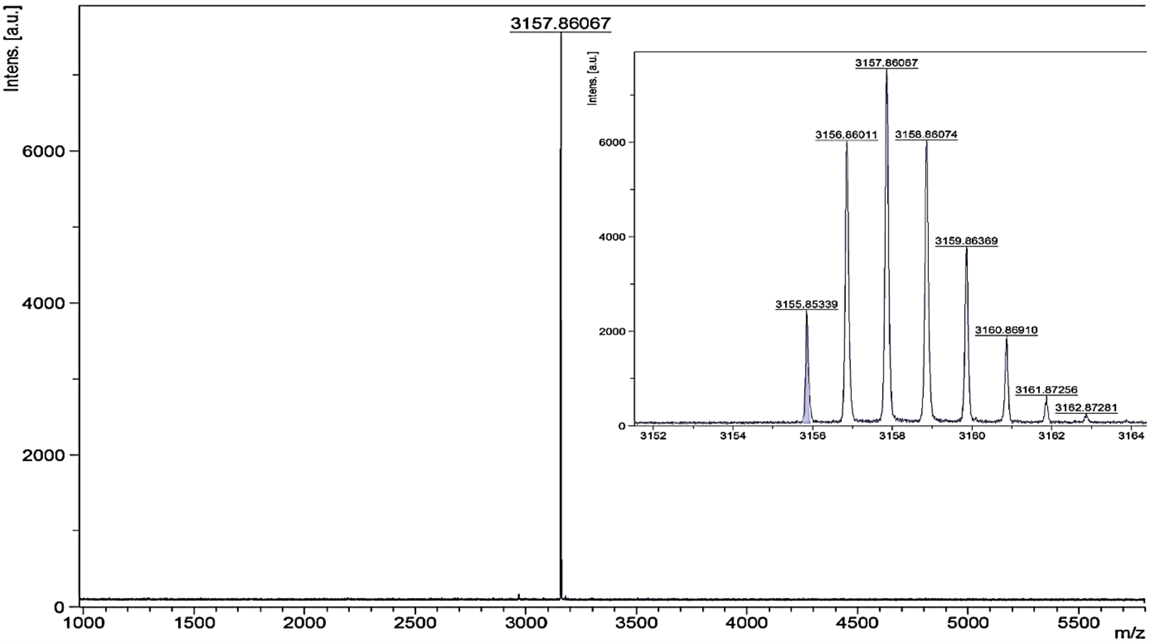

Supplementary Figure 100. HRMS (MALDI-TOF, positive mode, DCTB in chloroform) spectrum of the monomeric **PBI-Cap3**.

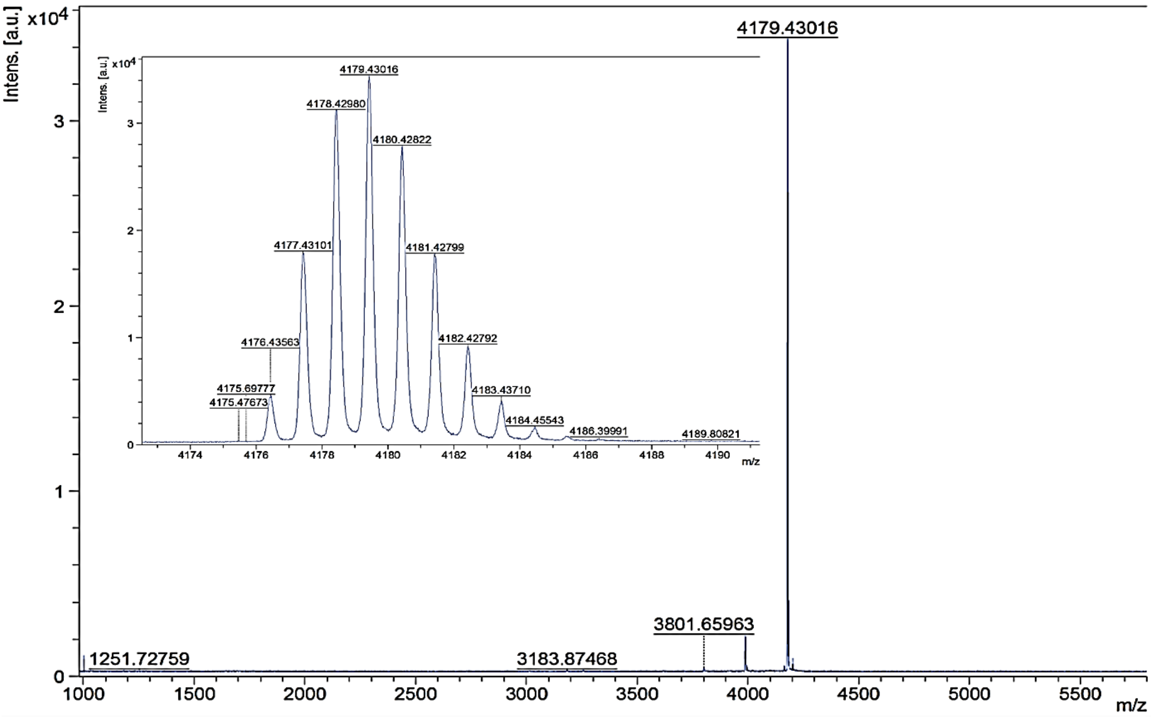

Supplementary Figure 101. HRMS (MALDI-TOF, positive mode, DCTB in chloroform) spectrum of the monomeric **PBI-Cap4**.

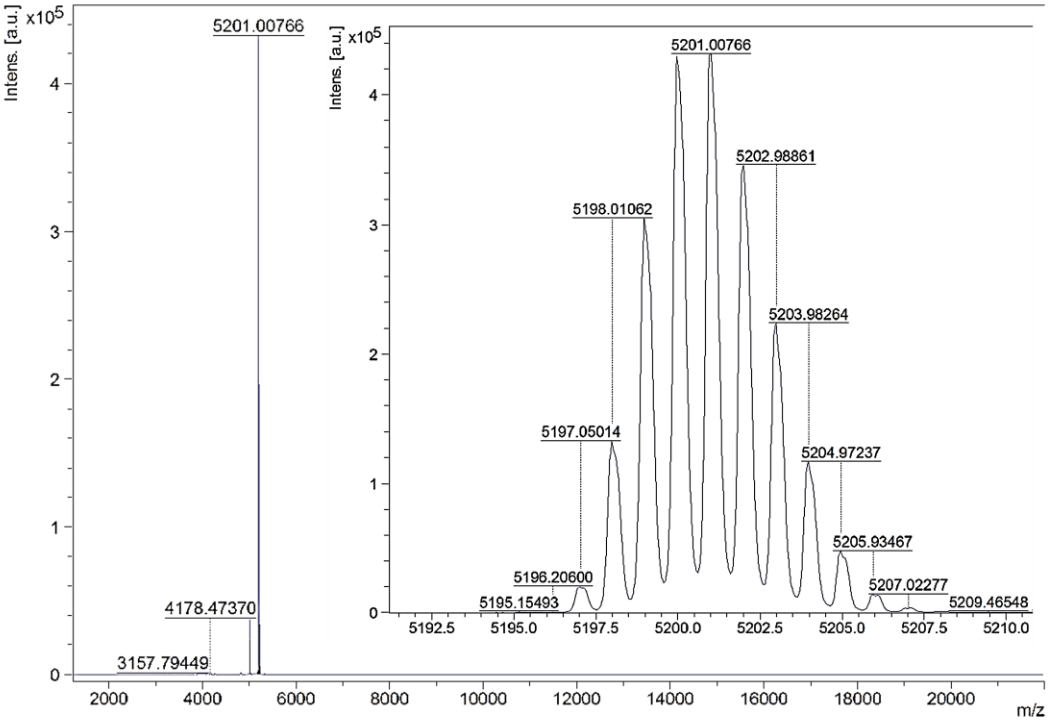

Supplementary Figure 102. HRMS (MALDI-TOF, positive mode, DCTB in chloroform) spectrum of the monomeric **PBI-Cap5**.

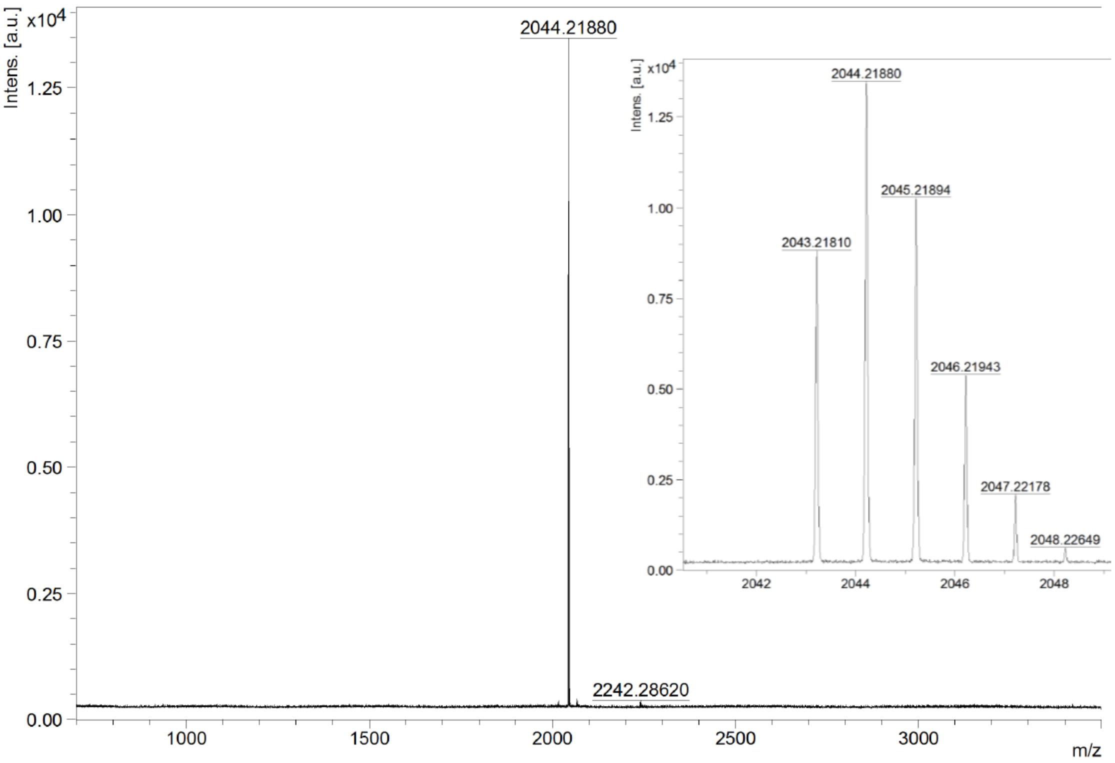

Supplementary Figure 103. HRMS (MALDI-TOF, positive mode, DCTB in chloroform) spectrum of **PBI-2**.

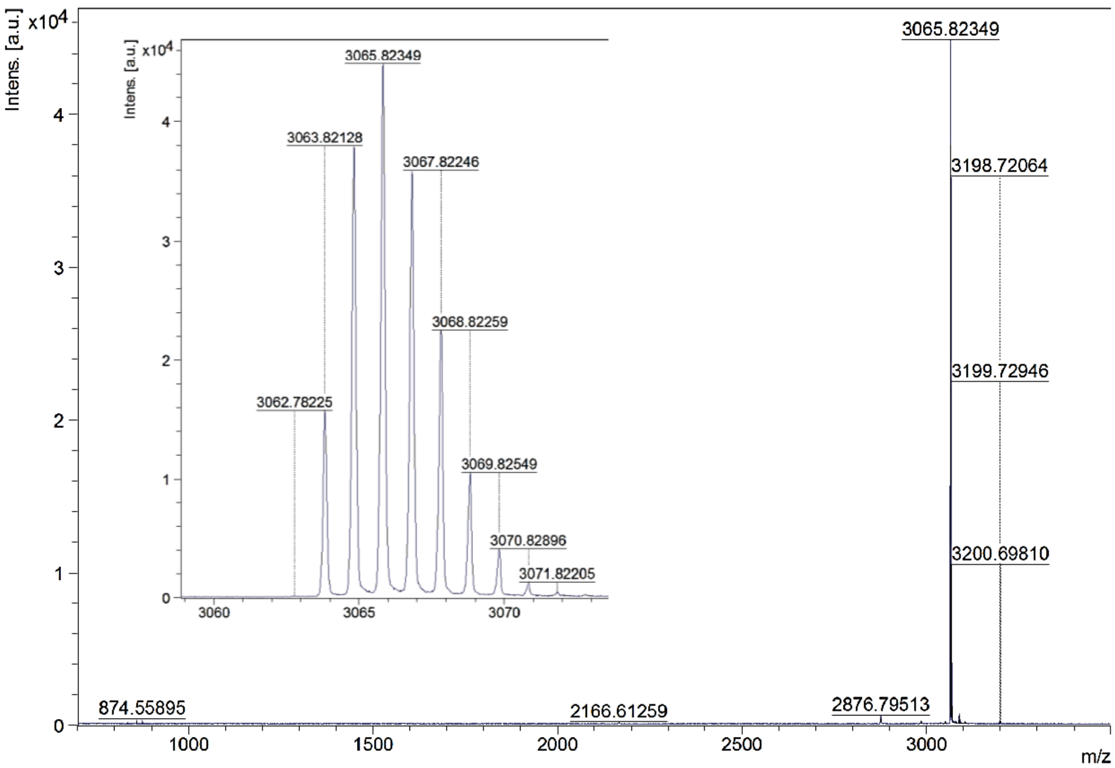

Supplementary Figure 104. HRMS (MALDI-TOF, positive mode, DCTB in chloroform) spectrum of **PBI-3.**

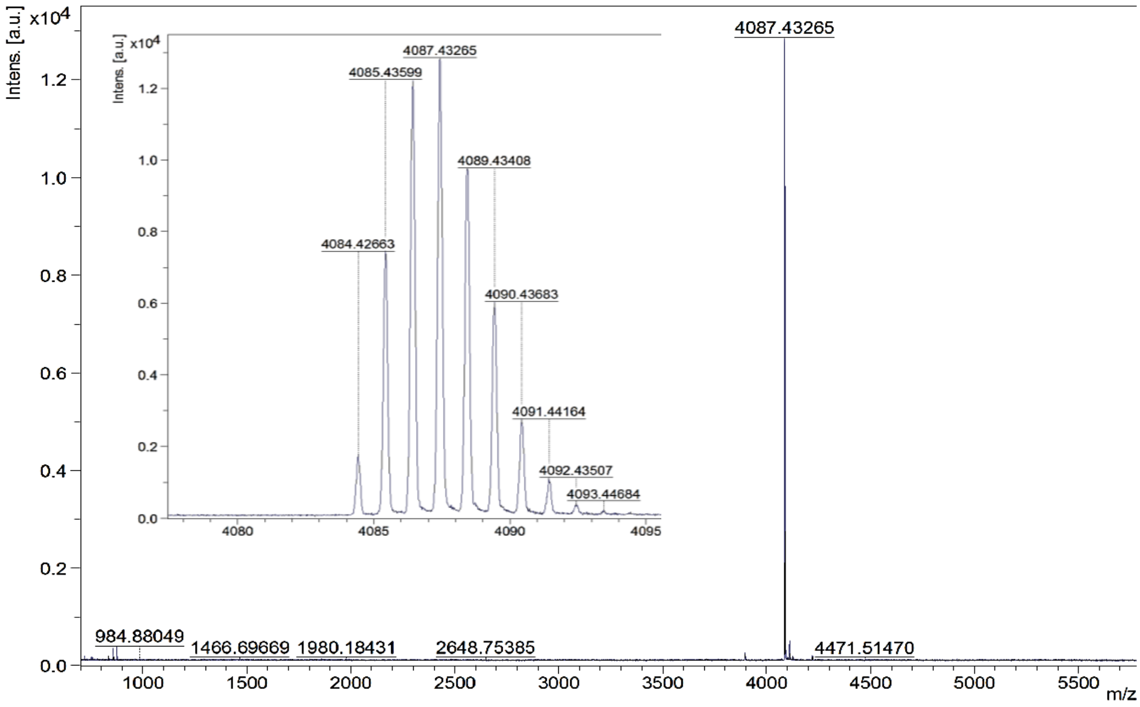

Supplementary Figure 105. HRMS (MALDI-TOF, positive mode, DCTB in chloroform) spectrum of **PBI-4**.

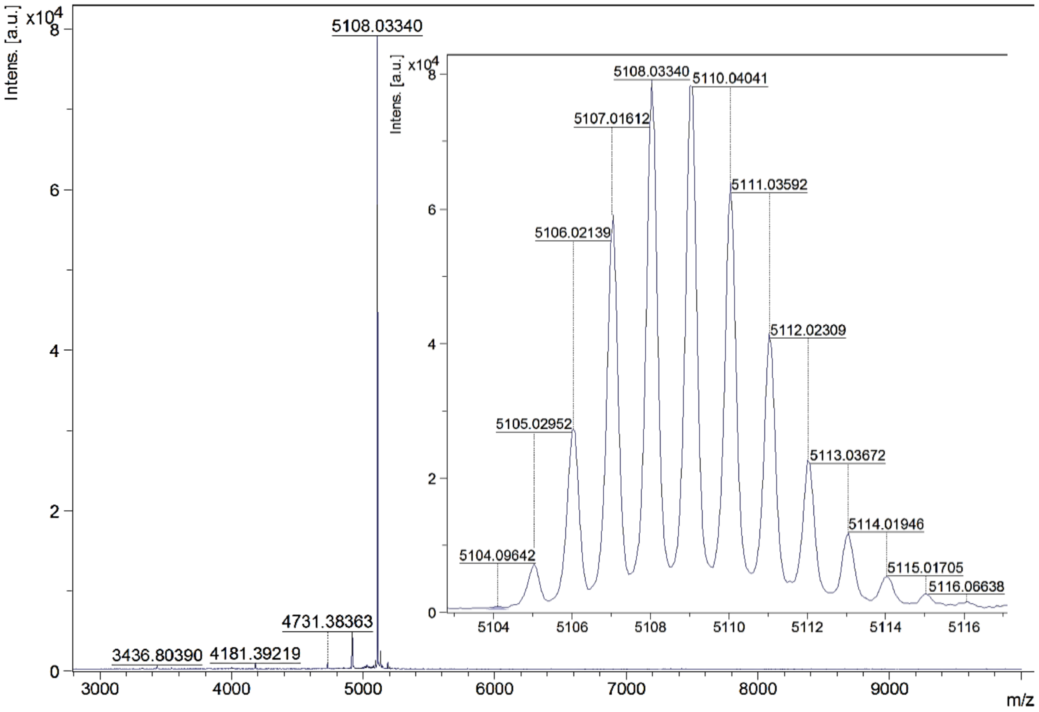

Supplementary Figure 106. HRMS (MALDI-TOF, positive mode, DCTB in chloroform) spectrum of **PBI-5**.

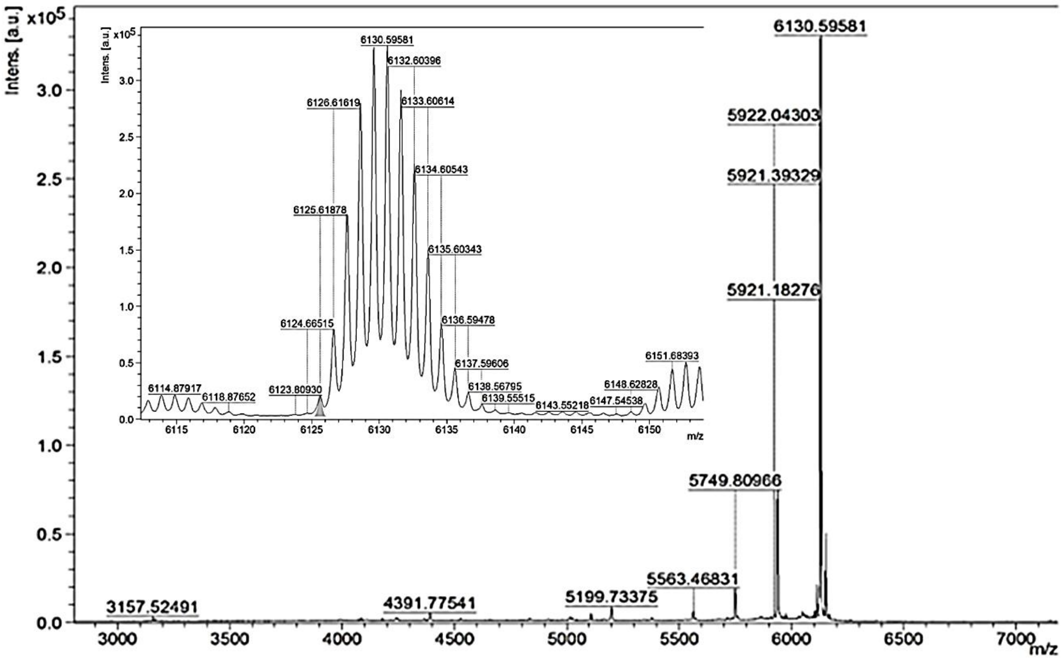

Supplementary Figure 107. HRMS (MALDI-TOF, positive mode, DCTB in chloroform) spectrum of **PBI-6**.

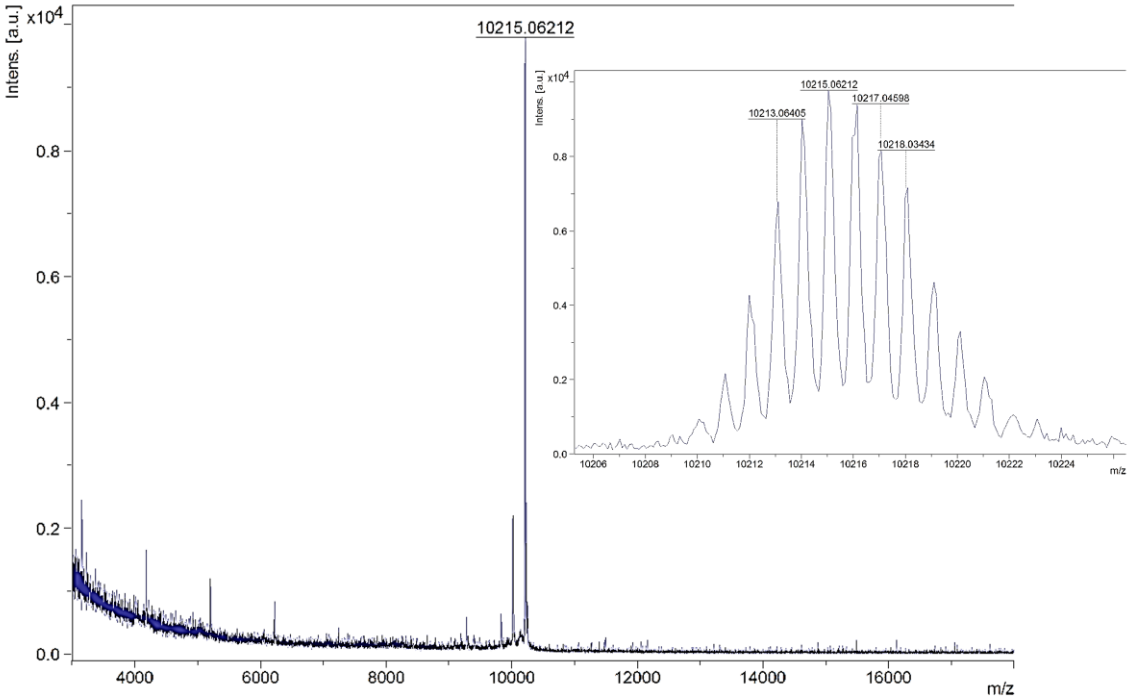

Supplementary Figure 108. HRMS (MALDI-TOF, positive mode, DCTB in chloroform) spectrum of **PBI-10**.

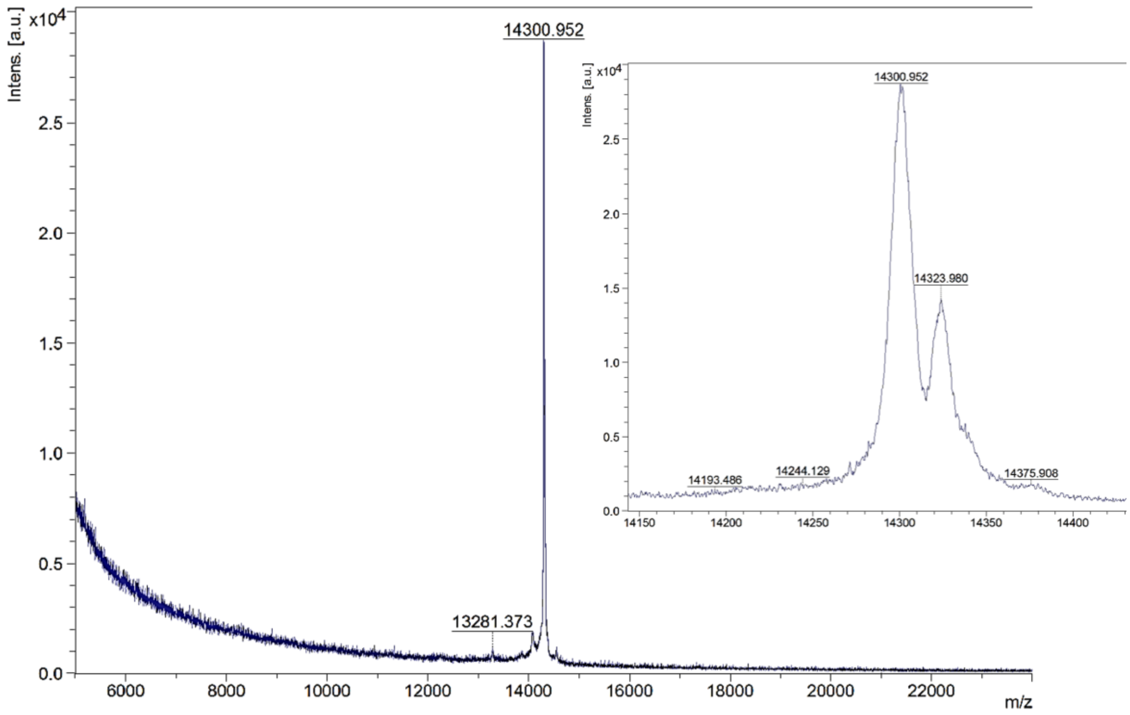

Supplementary Figure 109. HRMS (MALDI-TOF, positive mode, DCTB in chloroform) spectrum of **PBI-14**.

9. References

1. Guo, X. et al. Thieno[3,4- c]pyrrole-4,6-dione-based polymer semiconductors: Toward high-performance, air-stable organic thin-film transistors. *J. Am. Chem. Soc.* **133**, 13685–13697 (2011).

2. Zhang, S. et al. Perylene diimide copolymers with dithienothiophene and dithienopyrrole: Use in n-channel and ambipolar field-effect transistors. *J. Polym. Sci. Part A Polym. Chem.* **51**, 1550–1558 (2013).

3. Ernst, L., Song, H., Kim, D. & Würthner, F. Photoinduced stepwise charge hopping in π-stacked perylene bisimide donor–bridge–acceptor arrays. *Nat. Chem.* (2025).

4. Prasad, T. E. V. et al. Densities and viscosities of binary mixtures of m-cresol with some chlorohydrocarbons. *J. Solution Chem.* **34**, 1263–1272 (2005).

5. Baragi, J. G. et al. Density, viscosity, refractive index, and speed of sound for binary mixtures of anisole with 2-chloroethanol, 1,4-dioxane, tetrachloroethylene, tetrachloroethane, DMF, DMSO, and diethyl oxalate at (298.15, 303.15, and 308.15) K. *J. Chem. Eng. Data* **50**, 910–916 (2005).

6. Gerecke, M., Bierhance, G., Gutmann, M., Ernsting, N. P. & Rosspeintner, A. Femtosecond broadband fluorescence upconversion spectroscopy: Spectral coverage versus efficiency. *Rev. Sci. Instrum.* **87**, 53115 (2016).

7. Sajadi, M., Quick, M. & Ernsting, N. P. Femtosecond broadband fluorescence spectroscopy by down- and up-conversion in β-barium borate crystals. *Appl. Phys. Lett.* **103**, 173514 (2013).

8. Zhao, L., Luis Pérez Lustres, J., Farztdinov, V. & Ernsting, N. P. Femtosecond fluorescence spectroscopy by upconversion with tilted gate pulses. *Phys. Chem. Chem. Phys.* **7**, 1716–1725 (2005).

9. Schanz, R., Kovalenko, S. A., Kharlanov, V. & Ernsting, N. P. Broad-band fluorescence upconversion for femtosecond spectroscopy. *Appl. Phys. Lett.* **79**, 566–568 (2001).

10. Zhang, X.-X. *et al.* Femtosecond broadband fluorescence upconversion spectroscopy: Improved setup and photometric correction. *Rev. Sci. Instrum.* **82**, 063108 (2011).

11. Koti, A. S. R. & Periasamy, N. Application of time resolved area normalized emission spectroscopy to multicomponent systems. *J. Chem. Phys.* **115**, 7094–7099 (2001).

12. Frisch, M. J. et al. Gaussian 16, Revision C.01, Gaussian, Inc., Wallingford CT (2016).

13. Chai, J. D. et al. Long-range corrected hybrid density functionals with damped atom–atom dispersion corrections. *Phys. Chem. Chem. Phys.* **10**, 6615–6620 (2008).

14. Weigend, F. et al. Balanced basis sets of split valence, triple zeta valence and quadruple zeta valence quality for H to Rn: Design and assessment of accuracy. *Phys. Chem. Chem. Phys.* **7**, 3297–3305 (2005).

15. Edward, J. T. Molecular volumes and the Stokes–Einstein equation. *J. Chem. Educ.* **47**, 261 (1970).

16. Martin, R. L. Natural transition orbitals. *J. Chem. Phys.* **118**, 4775–4777 (2003).

17. Hong, Y. et al. Steering the multiexciton generation in slip-stacked perylene dye array via exciton coupling. *Nat. Commun.* **13**, 4488 (2022).

18. Kaufmann, C., Bialas, D., Stolte, M. & Würthner, F. Discrete π-Stacks of Perylene Bisimide Dyes within Folda-Dimers: Insight into Long- and Short-Range Exciton Coupling. *J. Am. Chem. Soc.* **140**, 9986–9995 (2018).

19. Chako, N. Q. Absorption of light in organic compounds. *J. Chem. Phys.* **2**, 644–653 (1934).

20. Lu, T. et al. Multiwfn: A multifunctional wavefunction analyzer. *J. Comput. Chem.* **33**, 580–592 (2012).

21. Chang, J. C. Monopole effects on electronic excitation interactions between large molecules. I. Application to energy transfer in chlorophylls. *J. Chem. Phys.* **67**, 3901–3909 (1977).

22. Kenny, E. P. et al. Benchmarking calculations of excitonic couplings between bacteriochlorophylls. *J. Phys. Chem. B* **120**, 25–32 (2016).

23. te Velde, G. et al. Chemistry with ADF. *J. Comput. Chem.* **22**, 931–967 (2001).

24. Fonseca Guerra, C. et al. Towards an order-N DFT method. *Theor. Chem. Acc.* **99**, 391–403 (1998).

25. Perdew, J. P. et al. Generalized gradient approximation for the exchange-correlation hole of a many-electron system. *Phys. Rev. B* **54**, 16533–16539 (1996).

26. Barbieri, P. L. et al. Gaussian basis sets of triple and quadruple zeta valence quality for correlated wave functions. *Mol. Phys.* **104**, 2945–2954 (2006).

27. Valeev, E. F. et al. Effect of electronic polarization on charge-transport parameters in molecular organic semiconductors. *J. Am. Chem. Soc.* **128**, 9882–9886 (2006).

28. Hestand, N. J. et al. Molecular aggregate photophysics beyond the Kasha model: Novel design principles for organic materials. *Acc. Chem. Res.* **50**, 341–350 (2017).

29. Scholes, G. D. et al. Electronic interactions and interchromophore excitation transfer. *J. Phys. Chem. A* **98**, 4580–4590 (1994).

30. Austin, A. et al. Enhanced Davydov splitting in crystals of a perylene diimide derivative. *J. Phys. Chem. Lett.* **8**, 1118–1123 (2017).

31. Lin, C. et al. Accelerating symmetry-breaking charge separation in a perylenediimide trimer through a vibronically coherent dimer intermediate. *Nat. Chem.* **14**, 786–793 (2022).

32. Zhang, X.-F., Yang, X. & Xu, B. PET-based bisBODIPY photosensitizers for highly efficient excited triplet state and singlet oxygen generation: tuning photosensitizing ability by dihedral angles. *Phys. Chem. Chem. Phys.* **19**, 24792–24804 (2017).

33. Mondal, S., Jethwa, R. B., Pant, B., Hauschild, R. & Freunberger, S. A. Singlet oxygen formation in non-aqueous oxygen redox chemistry: direct spectroscopic evidence for formation pathways and reliability of chemical probes. *Faraday Discuss*. **248**, 175–189 (2024).

34. Sung, J., Kim, P., Fimmel, B., Würthner, F. & Kim, D. Direct observation of ultrafast coherent exciton dynamics in helical π-stacks of self-assembled perylene bisimides. *Nat. Commun.* **6**, 8646 (2015).

35. Kaufmann, C., Kim, W., Nowak-Król, A., Hong, Y., Kim, D. & Würthner, F. Ultrafast Exciton Delocalization, Localization, and Excimer Formation Dynamics in a Highly Defined Perylene Bisimide Quadruple π-Stack. *J. Am. Chem. Soc.* **140**, 4253–4258 (2018).

36. Rühe, J. et al. Perylene bisimide cyclophanes: Structure–property relationships upon variation of the cavity size. *Org. Mater.* **2**, 149–158 (2020).
